# CNVcaller: highly efficient and widely applicable software for detecting copy number variations in large populations

**DOI:** 10.1093/gigascience/gix115

**Published:** 2017-12-04

**Authors:** Xihong Wang, Zhuqing Zheng, Yudong Cai, Ting Chen, Chao Li, Weiwei Fu, Yu Jiang

**Affiliations:** College of Animal Science and Technology, Northwest A&F University, Yangling, Shaanxi 712100, China

**Keywords:** copy number variation, next-generation sequencing, read depth, population genetics, absolute copy number

## Abstract

**Background:**

The increasing amount of sequencing data available for a wide variety of species can be theoretically used for detecting copy number variations (CNVs) at the population level. However, the growing sample sizes and the divergent complexity of nonhuman genomes challenge the efficiency and robustness of current human-oriented CNV detection methods.

**Results:**

Here, we present CNVcaller, a read-depth method for discovering CNVs in population sequencing data. The computational speed of CNVcaller was 1–2 orders of magnitude faster than CNVnator and Genome STRiP for complex genomes with thousands of unmapped scaffolds. CNV detection of 232 goats required only 1.4 days on a single compute node. Additionally, the Mendelian consistency of sheep trios indicated that CNVcaller mitigated the influence of high proportions of gaps and misassembled duplications in the nonhuman reference genome assembly. Furthermore, multiple evaluations using real sheep and human data indicated that CNVcaller achieved the best accuracy and sensitivity for detecting duplications.

**Conclusions:**

The fast generalized detection algorithms included in CNVcaller overcome prior computational barriers for detecting CNVs in large-scale sequencing data with complex genomic structures. Therefore, CNVcaller promotes population genetic analyses of functional CNVs in more species.

Copy number variations (CNVs) are defined as duplications or deletions of genomic segments that range in size from 50 base pairs (bp) to megabase pairs (Mb) and vary among individuals or species [1]. As a prevalent and important source of genetic diversity, more than 50 000 CNVs have been detected in the human genome, accounting for 10% of the entire genome [2]. CNVs regulate gene expression via both gene dosage and position effects, and they have larger expression-altering effect sizes than single nucleotide polymorphisms (SNPs) and indels [3]. In the human genome, CNVs are important genetic components of numerous diseases [4, 5] and a primary driving force of evolution [6]. Furthermore, CNVs are associated with different phenotypes and functions in animals and plants [7–11].

With the dramatic increase in sequencing capacity and the accompanying decrease in sequencing cost, whole-genome sequencing data are becoming the main source of CNV detection. The large-scale population sequencing data also provide an unprecedented opportunity to discover the functional CNVs using genome-wide association studies (GWAS) and evolutionary analysis [11, 12]. To study the polymorphism among individuals, the overlapping CNVs need to be merged into unified regions, namely CNV regions (CNVRs) [13]. As merging CNVs identified in each individual is inconvenient for large populations, some methods use multiple samples as input, then output the CNVRs directly [14]. More importantly, the population-based methods can improve detection by building statistical models, such as Poisson distribution and the Gaussian Mixture Model [15, 16].

As the amount of data increases, the computational efficiency is becoming a rate-limiting factor in CNV analysis. In addition, most CNV detection algorithms are based on mapping the sequencing reads back to the reference genome. For example, the methods of read-pair (RP) and split-read (SR) deduce the breakpoint of CNVs from the discordant alignments [17–21]; the method of read depth (RD) refers to the depth of coverage in a genomic region that can be calculated from the number of aligned reads [14]. A duplicated or deleted region should have a higher or lower RD than expected [22–24]. However, the nonmodel genome assemblies are riddled with many gaps, unplaced scaffolds, and misassembled segmental duplications (SDs) [25–28]. For example, 97% of highly similar tandem duplications in the Btau4.1 cattle genome assembly actually correspond to a single copy [29]. Therefore, more robust signal detection and noise reduction algorithms are required for detecting CNVRs from nonmodel species.

In this study, we introduce a super-fast generalized method, CNVcaller, for analysing CNV sequencing data in large populations (CNVcaller, RRID:SRC_015752). Based on the RD algorithm, this software applies robust signal detection and noise deduction methods to increase the computational efficiency in complex genomes. We applied CNVcaller to population sequencing data of humans, livestock, and crops to demonstrate its utility and benchmarked it against the widely used CNV detectors.

## Materials and Methods

### Input data

CNVcaller requires alignment files in BAM format as the main input. The following data/samples were included in the validation. (1) Human. Thirty human BAM files from the 1000 Genomes Project (1000GP) Phase 3 [30], including 27 normal (∼×12) and 3 deeply sequenced samples (∼×50) and 30 BAM files (∼×20) for 10 families from the Genomes of Netherlands (GoNL) project [31]. (2) Sheep. Seventy FASTQ files were downloaded from the NCBI BioProject PRJNA160933 (∼×10). Three Tan sheep trios (∼×19, including a total of 8 individuals; 1 ewe was the mother of 2 trios) were from unpublished data. (3) Goat. A total of 103 FASTQ files were acquired from NCBI [32–35], and the remaining 129 were generated by ourselves (∼×12, unpublished data). (4) Plant. Two maize [36] and 2 soybean [11] FASTQ files (each species containing 1 ∼×5 and 1 ∼×10 sample) were downloaded from NCBI. Details of the downloaded files are provided in Supplementary Table S1.

The FASTQ files were aligned to their respective reference assemblies using the Burrows-Wheeler Aligner (BWA) 0.7.13 (BWA, RRID:SCR_010910) [37]. The versions of the reference genomes included human GRCh37, maize B73 RefGen_v3, soybean Glycine_max_v2.0, sheep OAR_v3.1, and goat ARS1. After alignment, the polymerase chain reaction (PCR) duplications were marked using Picard 2.1 [38], and realignment was performed by GATK v3.5 (GATK, RRID:SCR_001876) [39]. The reads with a 0×504 flag (indicating unmapped, secondary mapped, or PCR duplication) were removed.

### Individual RD processing

#### RD estimation

**Figure 1:**
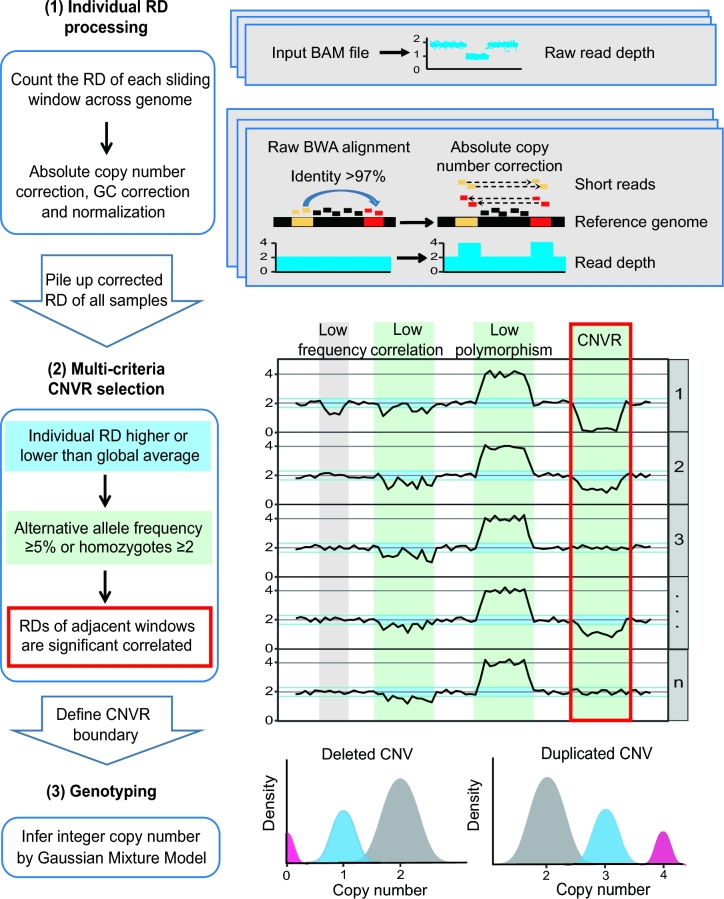
CNVcaller algorithm flowchart (left) and the key algorithms of each step (right). (1) Individual RD processing. In the absolute copy number correction, the RDs of highly similar windows were added together to deduce the absolute copy number. (2) Multicriteria CNVR selection. The curves show the copy numbers in a specific region for multiple samples. The blue transverse boxes mark the windows with a significant distinguishing copy number from the average (individual criterion). The green vertical boxes indicate that these regions meet the frequency conditions, and the red frame indicates that the RDs between the 2 adjacent windows are significantly correlated (population criteria). The fourth bar from the left, satisfying all the above conditions, is selected as the CNVR. (3) Genotyping: The copy numbers in each CNVR are clustered by a Gaussian Mixture Model to distinguish the normal, heterozygous, and homozygous samples.

The pipeline of CNVcaller is shown in Fig. 1. To calculate the RD signal, we first divided the reference genome into overlapping sliding windows, which were used for all samples. Windows with >50% gaps were excluded from the database and further computation. Then, the BAM file for each individual was parsed out using SAMtools v1.3 (SAMTOOLS, RRID:SCR_002105) [39], and the RD signal was calculated for each window as the number of placed reads with centres within the window boundaries. Considering the uncontrollable effect of gap ratios from different genome assemblies, all of the end reads located in the window were independently added to the RD of this window, regardless of whether the read was from single-end mapping or paired mapping. The window size was an important parameter for RD methods. CNVcaller uses half of the window size as the step size. The optimal window size is 800 bp (with a 400-bp overlap) for ×5–10 coverage human and livestock sequencing data (Supplementary Figure S1). The recommended window sizes are inversely related with coverage, and thus, ∼400-bp windows correspond to ×20 coverage, and ∼200-bp windows correspond to ×50 coverage.

#### Absolute copy number correction

To perform absolute copy number correction, windows with >97% sequence similarity were linked together to form a duplicated window record file. This file was generated by splitting the reference genome into nonoverlapping windows and aligning the windows onto the reference genome using the precise aligner BLAT v. 36×1 [40]. Windows with more than 20 hits were excluded to remove the low-complexity regions. The record files for humans, livestock, and main crops can be downloaded from the CNVcaller website [41]. Based on the duplicated window record file, the raw RDs located on similar windows were summed to generate the absolute RD for all high-similarity windows:

\begin{equation*}RD_{absolute}^i = \sum\limits_{j = 1}^t {RD_{raw}^{ij}} \end{equation*}

where *i* is the index of the window to be corrected, *t* is the total number of the high-similarity windows, $RD_{raw}^{ij}$ is the raw RD of the window that is similar to the *i*th window (including the *i*th window itself), which is counted directly from the BWA alignment, and $RD_{absolute}^i$ is the corrected RD of the *i*th window, which can be used to deduce the absolute copy number.

#### GC correction

Considering that the population sequencing data may come from different platforms, the RD of each individual sample was counted and corrected. Because the resequencing samples may show various GC content distributions, the GC bias was corrected individually, similar to the method used in CNVnator (CNVnator, RRID:SCR_010821) [23]:

\begin{equation*}RD_{corrected}^i = \frac{{{{\overline {RD} }_{40}}}}{{{{\overline {RD} }_{gc}}}}RD_{absolute}^i\end{equation*}

where *i* is the window index, $RD_{absolute}^i$ is the RD after absolute copy number correction, $RD_{corrected}^i$ is the final corrected RD for the window, ${\overline {RD} _{40}}$ is the mean RD of windows with 40% GC as a standard, and ${\overline {RD} _{gc}}$ is the mean RD over all windows that have the same GC content as the *i*th window.

#### RD normalization

Because the samples have different sequencing depths, the corrected RD must be normalized to a single standard before population-level CNV detection. Assuming that the majority of the genome has normal copy numbers, the corrected RDs were divided by the global median RD for normalization to 1:

\begin{equation*}RD_{normalized}^i = \frac{{RD_{corrected}^i}}{{{{\overline {RD} }_{global}}}}\end{equation*}

where ${\overline {RD} _{global}}$ is the median of the $\overline {RD} _{corrected}^i$ of all windows.

#### RD corrections for sex chromosomes

Most mammalian and avian genomes show an XX/XY-type or ZZ/ZW-type sex-determining system. Their homogametic sex chromosomes (X or Z) constitute 5–10% of the total genome and show half the RD of the autosomes in XY or ZW individuals. Therefore, intensive correction for X and Z chromosomes is needed. The RD of the X or Z chromosome (the particular name provided by the user) is used to determine the sex of a particular individual. If the median RD of this chromosome is <×0.6 the median RD of the autosome, the individual is considered an XY or ZW type, and the RDs of this chromosome are doubled before normalization. Otherwise, nothing is performed for individuals determined to be XX or ZZ type.

#### Parallel processing of individual RDs

The CNVcaller processes the BAM file of each individual separately in the first step, and therefore, parallel computations can be performed to reduce the total running time. All BAM files are equally distributed into N groups, and each group contains M files. The max N is the total available processing cores, and M is the total number of BAM files/N. For example, the 232 goat BAM files were processed on a node with 32 processing cores and 124 GB of RAM. We distributed the 232 files into 20 groups, and each group contained 12 BAM files. The shell command for 1 group is as follows:

#!/bin/sh

for i in {1..M}

do bash Individual.Process.sh -b $i.bam -h $i -d dup -s sex_chromosome

done

After corrections and normalization, the comparable RDs of each sample are aggregated into an ∼100-MB intermediate file and output, thus preventing repeated calculations for the same individual in different populations.

### CNVR detection by multiple criteria

#### Individual candidate CNV window definition

The individual candidate CNV windows are defined using 2 criteria: (1) The normalized RD must be significantly higher or lower than the normalized mean RD (deletions < 1–2 * STDEV; duplications > 1 + 2 * STDEV). (2) Considering that the normalized RD of heterozygous deletions and duplications should be approximately 0.5 and 1.5, respectively, an empirical standard for the normalized RD (deletions < 0.65; duplications > 1.35) also must be achieved. For some strictly self-bred species, such as soybean and wheat, this empirical standard should be raised to 0.25 or 1.75 for the normalized RD of the homozygous deletions or duplications, respectively.

#### Population-level candidate CNV window definition

All the individual RD files are arranged according to the universal window index into a 2-dimensional population RD file. Each line of this file is the multisample RDs of a particular window, from which the candidate CNV windows are selected. The user can retain all the windows with at least 1 individual that shows heterozygous deletion or duplication. However, we recommend removing low-frequency windows in large populations with low sequencing coverage because of increased random mistakes. By default, windows with an allele frequency ≥0.05 or at least 2 homozygous duplicated/deleted individuals are selected for further validation. Then, Pearson's product-moment correlation coefficients of the multisample RDs are calculated between 2 adjacent nonoverlapping windows. If the Pearson's correlation index is significant at the *P* = 0.01 level by the Student *t* test, the 2 windows are merged into 1 call.

#### CNV region definition

The initial calls are selected if more than 4 sequential overlapping windows are defined as population-level candidate windows. Regarding noise tolerance, a maximum of 1 unselected window out of 4 continuous candidate windows is allowed; however, their RD is not calculated in the RD of CNVR. As CNVRs can be separated by gaps or poorly assembled regions, the adjacent initial calls are merged if their RDs are highly correlated. The default parameters are as follows: the distance between the 2 initial calls is less than 20% of their combined length, and the Pearson's correlation index of the 2 CNVRs is significant at the 0 = 0.01 level.

### CNVR genotyping

After merging the candidate CNV windows into a CNVR, the RDs of all samples in each CNVR are clustered, and the integer copy number of each individual is calculated, which represents the genotyping step as used in SNP detection. The copy number of a specific sample is initially estimated as 2 times the median RD of all candidate windows in a given region. Then, the copy numbers of all samples of a CNVR are decomposed into several Gaussian distributions. The expectation maximization (EM) algorithm is used to estimate the model parameters, and the effective number of components is inferred by the Dirichlet Process. To ensure the quality of the genotyping, the silhouette coefficient is calculated for each CNVR. The Python package scikit-learn v0.19.0 [42] is used to implement the above algorithms. This genotyping step can be performed in sequence or in parallel, and the parameter “nproc” is used to control the number of processes. The genotyping of 232 goats took 17.49 minutes and 488 MB of memory on 1 node with 2 processors. The final output is a VCF file, which can be analysed by SNP-based population genetic software.

### Performance evaluation

#### Competing methods

Most of the validations were based on the 30 human BAM files from 1000GP Phase 3 data, and only the autosomes were included unless otherwise noted. The performance of CNVcaller was compared with 2 pipelines, including CNVnator_v0.3.3 (CNVnator, RRID:SCR_010821) [23], which is widely used for CNVR detection in animal populations, and Genome STRiP (included in svtoolkit_2.00.1696) [16], which is the state-of-the-art CNV detector generated by 1000GP. The recommended parameters and quality controls were used. For Genome STRiP, both the deletion and CNV pipelines were utilized. The unplaced scaffolds were excluded, and the whole genome was separated by chromosomes as recommended. The standard screening procedures were applied to select the passing sites and to remove duplicate calls. For CNVnator, a 400-bp window was used, as recommended. The gap regions and calls with *P* values of less than 0.01 were removed, and the q0 filter was used to remove any predictions with q0 <0.5 (reads with multiple mapping locations), as recommended. The individual CNVs of all samples were merged into the population CNVRs based on the following arbitrary standards: 2 calls with >50% reciprocal overlap or 1 call with >90% coverage by another call [23, 43]. Then, the CNVRs were genotyped using the built-in function in CNVnator.

#### Sensitivity validation

Sensitivity was defined as the number of CNVs that existed in both the CNV predictions and the high-confidence CNVR database (>50% reciprocal intersection) divided by the total number of CNVs in the database. Calls with ≤2500 bp and an allele frequency <5% and sex chromosomes were removed from this study. Two previously published high-confidence CNVR databases, including the same samples from the test data, were used. One was the 1000GP CNVR map [44], which included 26 tested samples, and the other was array comparative genomic hybridization (aCGH)–based CNVR database [1], which included 10 tested samples. The CNVRs of the specific samples were extracted from the database and were then screened by the same length and frequency as the detected CNVRs (length > 2500 bp and alternative allele frequency ≥ 0.05). The intersected length of the predicted CNVRs and the high-confidence CNVR database was calculated using BEDTools v2.25 (BEDTools, RRID:SCR_006646) [45].

#### Accuracy validation

The intensity rank-sum (IRS) test (included in the svtoolkit_2.00.1696) was performed as in previous studies [16], based on the Affymetrix SNP 6.0 array intensity data of 26 test samples. Meanwhile, the genotyping accuracy was benchmarked against the aCGH CNVR database [1]. The predicted CNVs were subject to validation if the predicted regions had a >90% reciprocal intersection with 1 CNVR in the database. Only if the predicted genotyping was in exact agreement with the aCGH database was this genotype defined as correct. Also, the Mendelian inconsistencies were calculated from the deleted and biallelic duplicated CNVRs (maximum copy number ≤ 4) in the Dutch families and sheep trios.

#### Sheep genotyping validation by CNVplex assay

A total of 73 sheep, including Merino, Texel, Mongolia, and Tibetan sheep, were used for genotyping validation. Genomic DNA was extracted from peripheral blood using a QIAamp DNA blood mini kit (Qiagen, Germany). For each sheep, whole-genome sequencing (∼×10) was performed, and the CNVRs were detected by CNVcaller, as described above. The copy numbers of high-variant CNVRs were validated by CNVplex (Genesky Biotechnologies Inc., Shanghai, China), which is based on double ligation and multiplex fluorescence PCR [44]. The sizes of the PCR fragments and target loci sequences used in each reaction are listed in Supplementary Table S3.

### Absolute copy number validation

#### Detecting X-origin scaffolds

Unplaced scaffolds with high sequence similarity to the X chromosome were regarded as X-origin scaffolds. All the scaffolds of OAR v3.1 were mapped to the X chromosome of the sheep reference genome OAR v4.0, the goat reference genome ARS1 and the cattle reference genome UMD 3.1 using BLASR [46]. If the best hit of a scaffold had >50% coverage with >90% identity and >3-kb length, this scaffold was defined as a putative X-origin scaffold. In theory, all of these scaffolds were expected to be detected as high-frequency CNVRs because the RDs of the unplaced scaffolds were not corrected by sex. The detection and genotyping accuracy in the SD region were estimated using the sex information from 133 sheep.

#### mrsFAST alignment

The paired-end reads with multiple hits indicated by the “XA” tag in the BWA alignment were selected for realignment using mrsFAST_v3.3.10 [47], as previously described [48]. Longer reads were trimmed to 40 bp to reduce the read length heterogeneity prior to sequence alignment. After alignment, the reads with more than 20 hits were excluded to remove the low-complexity regions.

#### Simulations of the SD

The putative SDs were modified from a randomly selected 50-Mb single-copy region of Chr1 from the sheep reference (OAR v3.1). One hundred nonoverlapping regions of 5000 bp were randomly selected and artificially inserted as tandem duplications into the 50-Mb source sequence. The modified sequence with known SDs was used as the reference genome in the following study. In these putative SD regions, 2–6 copies were randomly assigned to 100 individuals, and all other regions are treated as normal copy regions. The wgsim [49] read simulator was used to sample the paired-end reads with default parameters. The coverage of the normal regions was set to ×20.

## Results and Discussion

### Computational cost in complex genomes from large population-based studies

**Figure 2:**
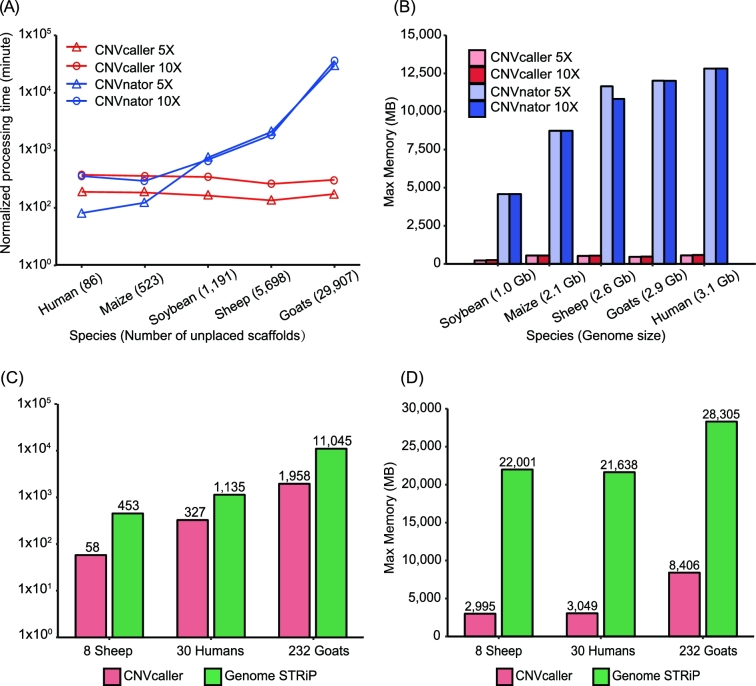
Computational performance of CNVcaller, CNVnator, and Genome STRiP. All the programs were executed on a single node with 2 2.40-GHz Intel Xeon E5–2620 v3 processors. (A, B) Log plots of the processing time (A) and the max memory (B) for 1 individual. The numbers of unplaced scaffolds of the reference genome are indicated in brackets. The processing time was normalized by the genome size and sequencing coverage to simulate a 3-Gb genome with ×5 or ×10 sequencing coverage. (C, D) Log plots of the total running time (C) and the max memory (D) of the population CNVR detection. The test cohorts are as follows: 8 sheep, 30 humans, and 232 goats with ×19, ×16, and ×12 average sequencing coverage, respectively. In Genome STRiP, the unplaced scaffolds were excluded.

As the computational cost is one of the greatest challenges for large populations, the computational efficiency of CNVcaller was evaluated on the real sequencing data of the different genomes. The individual RD processing step was compared with CNVnator, which detects CNVs individually and has been used in yak, chicken, and fish populations [43, 50, 51]. The processing time of CNVcaller was linearly related to the genome size and sequencing coverage: 20–40 minutes for a 3-Gb genome with ×10 coverage (Supplementary Table S3). However, the processing time of CNVnator exponentially increased with scaffold number, which was the only time-consuming index when the scaffold number exceeded 1000 (Fig. 2A). Consequently, CNVcaller achieved a 145-fold increase in speed over CNVnator for goat CNV detection. Notably, the goat reference genome ARS1, which contains 29 907 scaffolds, was newly assembled by single-molecule sequencing [35]. The robustness of CNVcaller reduces the quality restrictions of the reference genome, which promotes CNV research in species with a draft assembly at the scaffold level. This feature also enables comprehensive variation discoveries based on pan-genomes, which reveal numerous functionally important genes not localized on a single reference genome [52–54].

The memory requirement of CNVcaller is mainly related to the genome size: only approximately 500 MB of memory for a mammalian genome, which is less than 1/20th of the memory required by CNVnator (Fig. 2B). Therefore, in multisample CNV detection, the individual RD processing step can be run in parallel on 1 node to further reduce the running time. The population-level performance of CNVcaller was evaluated and benchmarked against Genome STRiP, which also detects CNVRs at the population level and is a main contributor of the 1000GP. After removing the unplaced scaffolds, CNVcaller was still 3.5–7.8 times faster than Genome STRiP (Fig. 2C), with a 70%∼86% reduced memory requirement (Fig. 2D). For 232 goats with a mean coverage of ×12, CNVcaller can complete CNV detection in 1.4 days using a single node. The high efficiency of CNVcaller can facilitate CNV detection in large populations.

### Absolute copy number correction in putative SDs of the sheep genome

**Figure 3:**
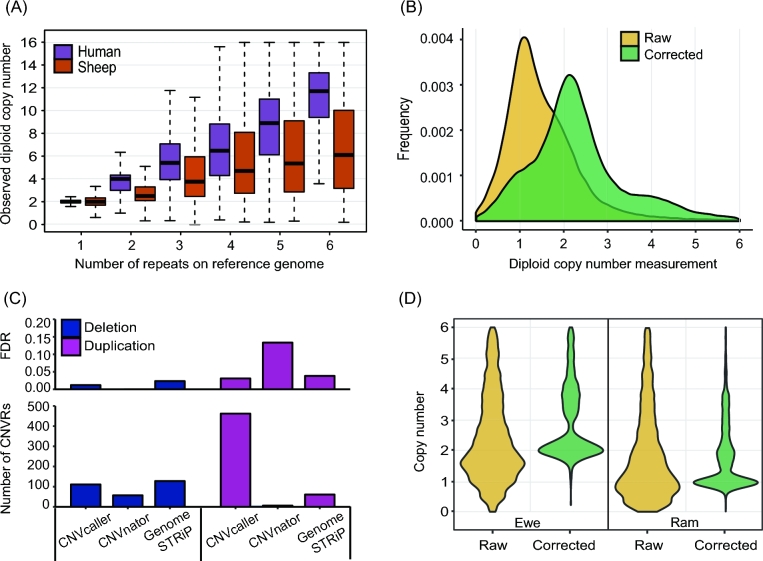
Absolute copy number correction in the sheep genome. (A) The copy numbers of all the windows with no more than 6 repeats were plotted against the repeat numbers in the reference genome. Compared with humans, the sheep sample had much lower copy numbers in the putative duplicated regions than expected. (B) The distribution of copy numbers of the putative 2-copy regions in the sheep genome before and after absolute copy number correction. After correction, the main peak of the copy number shifted to 2 (normal diploid copy number). The smaller peaks at 4, after correction, indicated the 20% real SDs. (C) The number and FDR (Mendelian inconsistency) of detected CNVRs residing in the SD regions. The sheep SD regions include the regions longer than 2 kb with >97% identity. The CNVRs residing in the SD regions were defined if more than 50% of a given CNVR overlapped with the SD regions. (D) The raw and corrected copy numbers of all the X-linked scaffolds of 133 sheep.

Previous studies have shown that a high proportion of SDs in animal genomes are misassembled single-copy regions [27, 29]. Therefore, we detected the ratios of false SDs on the human (hg19) and sheep (OAR v3.1) reference genome assemblies by the sequencing the copy number of a human (NA12878) and a Tan sheep sample (Fig. 3A). If the SDs were correctly assembled, the sequencing diploid copy number should be twice the copy number of the SDs. For example, the average sequencing copy number of the 2-copy SDs was 4 in NA12878. However, the corresponding sequencing copy number in sheep was only 2.4. These results indicated that most 2-copy SDs of hg19 were truly duplicated in NA12878, while approximately 80% of the 2-copy SDs in OAR v3.1 were single-copy regions in the Tan sheep sample. Thus, the SDs in the sheep genome were called “putative SDs” before validation.

CNV detection can be confounded by the presence of false SDs. Due to the random placement of multiple mapped reads, the RD signal in these regions is effectively smeared over all copies; thus, the raw copy number is underestimated. For example, in the putative 2-copy SDs, the main peak of the copy numbers was 1, the same as heterozygous deletions (Fig. 3B). CNVcaller incorporates absolute copy number correction by simply adding the RD of the putative SDs to deduce the absolute copy number independent of the copy number of the genome assembly (Fig. 1). This target can also be achieved using mrsFAST; however, more than 10 core hours were required to realign the multihit reads by mrsFAST for a mammalian genome with ×10 sequencing coverage. The equivalent result was achieved by CNVcaller within only 0.06 core hours (Supplementary Figure S2).

In the simulated sheep sequencing data, this correction deduced the correct genotyping in SD regions (Supplementary Figure S3) and reduced the STDEV within each genotyping (Supplementary Table S4). In the real individual sheep data, the corrected putative 2-copy SDs clearly fell in to 2 categories: normal copy (the major peak, with a diploid copy number of 2) and the true duplicated regions (the minor peak, with a diploid copy number of 4) (Fig. 3B). The accuracy of the CNVRs in putative SDs was validated by the Mendelian inconsistency of 3 Tan sheep trios. CNVcaller detected more duplications in the putative SDs, with only 3% Mendelian inconsistency (Fig. 3C).

The sensitivity of sheep CNVRs was estimated indirectly due to the lack of a validated database. Based on our integrated analysis (see the Methods section), there were 138 sheep X chromosome–origin scaffolds that were not anchored onto chromosomes of OAR v3.1. Therefore, all of these scaffolds should be detected as CNVs because the rams had half the copy numbers of the ewes. As a result, CNVcaller detected 101 of these 138 X-origin scaffolds, with a sensitivity of 73%. In contrast, CNVnator and Genome STRiP did not report these regions. Furthermore, the copy numbers of single-copy and duplicated X-origin scaffolds were centred at integers, namely 1 and 2 in rams and doubled in ewes, whereas the peaks of the raw copy numbers were ambiguous (Fig. 3D). Further examination of the duplicated regions showed that this result was caused by splitting the raw RDs among the putative SDs (Supplementary Figure S4).

### Performance evaluations on sheep data

To evaluate the robustness and false discovery rate (FDR) in sheep, we used CNVcaller, CNVnator, and Genome STRiP to detect CNVRs from 3 sheep trios. CNVnator detected less than 1/10th the number of CNVRs of the other methods, with only 260 CNVRs reported. One main reason was that assembly gaps without reads were detected as homozygous deletions by CNVnator in the initial calls. Thus, more than 90% of the initial calls were removed in the recommended gap filtering step because the sheep reference genome OAR v3.1 has ∼125 000 gaps. By comparison, the human reference genome hg19 has only 354 gaps. To address the gap problem in nonhuman genomes, CNVcaller removed the sliding windows with gaps at the first step, and adjacent CNVRs were merged into 1 call if their RDs were ultimately highly correlated. These optimizations avoided the artefacts caused by assembly errors and retained the adjacent CNVRs as well.

**Figure 4:**
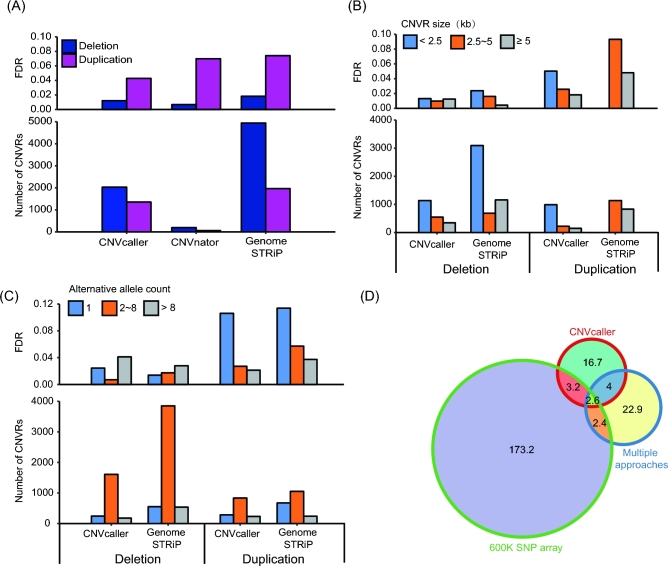
Performance evaluations on the sheep data. (A) CNVR number and FDR (Mendelian inconsistency) of 3 sheep trios. (B) The number of calls and FDR partitioned by CNV length. (C) The number of calls and FDR partitioned by allele frequency. The frequency was shown by the alternative allele number. (D) The length of overlapping CNVRs (in Mb) detected by CNVcaller and 2 other large-scale sheep studies with different approaches and platforms.

The accuracy was evaluated by the Mendelian inconsistency of all the CNVRs on autosomes against the length and alternative allele frequency (Fig. 4). CNVcaller achieved higher accuracy than Genome STRiP in both deletion (1% vs 2%) and duplication (4% vs 7%) (Fig. 4A). Whereas Genome STRiP had greater capability to detected short (<2.5 kb) deletions (Fig. 4B), indicating that the RP methods integrated in Genome STRiP performed well on small deletions. Concerning the alternative allele frequency, both methods showed an increased FDR in rare duplications (Fig. 4C). However, CNVcaller is primarily used to detect CNVRs related to economic traits in livestock and crops. In these studies, the target CNVRs usually have a high frequency after long-duration breeding selection.

To investigate the reproducibility of CNVcaller, the CNVRs identified by CNVcaller from 133 sheep of 44 worldwide breeds were compared with 2 other recently released large-scale sheep CNVR data sets. One data set was derived from allied breeds using multiple platforms, including aCGH, SNP chip, and whole-genome sequencing [55], and the other data set was based on 3 Chinese sheep breeds using a 600-K SNP array [56]. The samples and platforms had major influences on the results; therefore, the overall intersection ratio was low. However, CNVcaller covered 51% of the cross-validated regions of the other data set (Fig. 4D).

**Figure 5:**
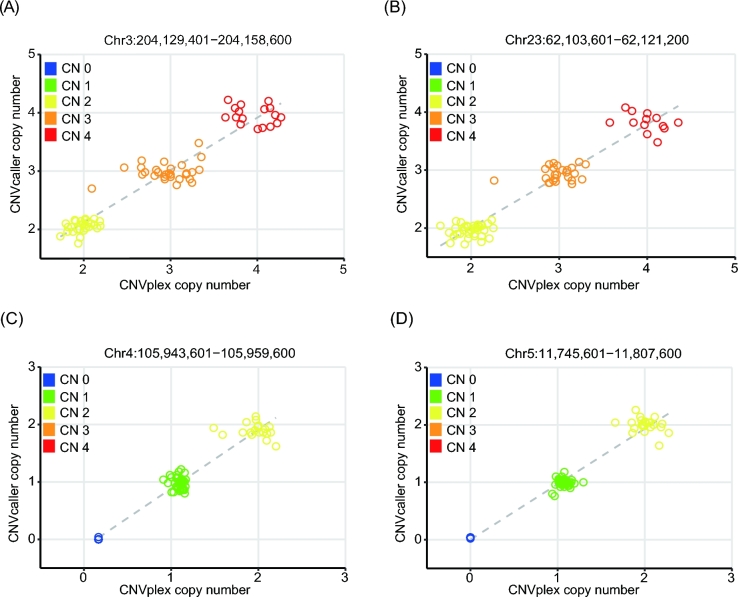
Evaluation of the sheep CNV genotypes by CNVplex. Two duplicated (A, B) and 2 deleted (C, D) CNVRs with a high variation frequency were typed in CNVplex using 73 sheep samples. The copy number genotypes predicted by CNVcaller from the sequencing data were plotted against the CNVplex measurements of the same animal.

The genotyping accuracy of 73 sheep was validated by a recently developed molecular biology technique, CNVplex. This method reports the copy number of a genomic sequence based on the multiplex ligation-dependent probe amplification (MLPA) method [44]. When the copy numbers of sequencing data predicted by CNVcaller and CNVplex were compared, the Pearson’s product–moment correlation coefficients were greater than 0.95, and the genotyping concordance was 98% (Fig. 5).

### Performance evaluations on 1000 Genomes Project data

**Figure 6:**
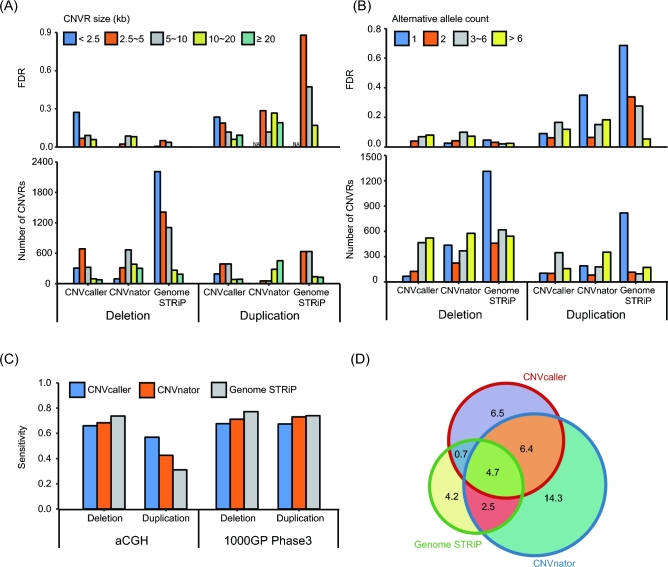
Performance evaluations on the 1000GP data. (A, B) The number of calls (A) and the IRS FDR (B) partitioned by CNV length. All the calls on the autosomes were included. (C, D) The number of calls (C) and the IRS FDR (D) partitioned by allele frequency. To eliminate the huge FDR diversity of the short CNVs, the effect of the allele frequency was evaluated using the >2500 bp calls. (C) The sensitivity (the proportion of high-confidence CNV database overlapped by the predicted CNVs) of the 3 methods. For the highly variable FDRs, the sensitivity estimation removed the calls of less than 2500 bp or that had an alternative allele frequency of less than 5%. (D) Comparison of CNVR results identified by CNVcaller, CNVnator, and Genome STRiP based on the same 30 BAM files from the 1000GP Phase 3. The length of overlapping CNVRs was indicated in Mb.

Although CNVcaller was mainly designed for complex genomes, the performance was also evaluated on 30 human BAM files from 1000GP. The SNP array data and high-confidence CNVR databases were only available in human data, which can be used to evaluate the accuracy for population level (IRS test) and sensitivity. CNVcaller demonstrated the highest overall accuracy for detecting duplications and performed consistently across the length and frequency categories, whereas Genome STRiP and CNVnator had high FDRs on the short or singleton duplications (Fig. 6A, B). Genome STRiP showed the greatest ability to detect deletions, indicating the advantage of combining RD and RP methods for deletion detection. The genotyping accuracy of the human data set was further benchmarked against the high-confidence aCGH array-based database. The discordance rates of CNVcaller, CNVnator, and Genome STRiP were 2.6%, 5.5%, and 2.2%, respectively. This genotyping accuracy ranking was the same with the Mendelian consistency of the 10 Dutch trios (Supplementary Figure S5).

The sensitivity was estimated as the proportion of the high-confidence CNVR database that overlapped with the predicted CNVRs. Two previously published high-confidence databases that include our test samples are the aCGH-based CNVR database [1] and the 1000GP CNVR map [2]. For the aCGH database, CNVcaller demonstrated the highest sensitivity (57%) in duplications, whereas Genome STRiP achieved the highest sensitivity (74%) in deletions (Fig. 6C). Both Genome STRiP and CNVnator were the core contributors to the 1000GP CNV maps. However, the sensitivity rates of CNVcaller were 68% and 67% for deletions and duplications according to this database, only 4–10% lower than Genome STRiP and CNVnator.

The 3 methods had a high degree of intersection with each other. The numbers of overlapping (>50%) calls were 429 (CNVcaller vs CNVnator), 502 (CNVcaller vs Genome STRiP), and 513 (CNVnator vs Genome STRiP). CNVcaller covered 40% of the CNVRs detected by CNVnator, 45% of the CNVRs detected by Genome STRiP, and 65% of their intersecting CNVRs by length (Fig. 6D).

## Conclusion

CNVcaller was designed to detect CNVRs from large-scale resequencing data from all types of genomes. The generalized detection and correction algorithms employed in CNVcaller greatly increase the computational efficiency of analysing complex genomes. The validation performed using sheep data showed that the absolute copy number correction increased the detection efficiency of the misassembled SDs, greatly reduced the running time, and deduced more reasonable copy numbers. Both the evaluations using sheep and human data indicated that CNVcaller achieved the best accuracy and sensitivity for detecting duplications. Therefore, this rapid and reliable population-level CNV detection method can promote the discovery of the missing heritability of complex traits and the accurate determination of causative mutations in more species.

## Availability and requirements

Project name: CNVcaller

RRID: SCR_015752

Project home page: http://animal.nwsuaf.edu.cn/software

https://github.com/JiangYuLab/CNVcaller

Operating system(s): platform independent

Programming language: Perl, Python

Other requirements: SAMtools 1.3 (using htslib 1.3), scikit-learn v0.19.0

License: GNU General Public License, version 3.0 (GPL-3.0)

## Availability of data

Snapshots of the supporting code and materials are hosted in the *GigaScience* repository, *Giga*DB [58].

## Additional files

Additional file: Figure S1: Number of CNVRs (A) and corresponding IRS FDR (B) were plotted against window size; 30 human BAM files of 1000GP Phase 3 were used as input.

Additional file: Figure S2: The haploid copy number distributions of a sheep sample at *ASIP* and *MOGAT* gene loci. The haploid copy number from top to bottom was counted from: raw BWA alignment, mrsFAST alignment, BWA alignment corrected by CNVcaller. The signal of each window was normalized to 1 using the global mean read depth of the sequencing data. The gray regions indicate the gaps in the reference genome.

Additional file: Figure S3: The absolute copy number of SD regions deduced by CNVcaller against the simulated copy number: 2, 3, 4, 5, 6. The red points indicate the mean values.

Additional file: Figure S4: The raw and corrected copy number of 36 duplicated X-origin scaffolds grouped by the number of repeats on reference genome. Noteworthy, the raw copy numbers were split among the putative SDs.

Additional file: Figure S5: FDR via the rate of Mendelian inconsistency and the number of detected CNVRs in the 10 Dutch families using CNVcaller, CNVnator, and Genome STRiP.

Additional file: Table S1: Validation sequencing data information.

Additional file: Table S2: PCR fragments sizes and target loci sequences in CNVplex.

Additional file: Table S3: Individual processing time and memory footprint of genomes with different genome sizes and unplaced scaffold numbers.

Additional file: Table S4: RD and STDEV of the simulated data before and after absolute copy number correction.

Additional file: Table S5: Detailed information of the CNVRs detected from 1000GP Phase 3 data.

### Abbreviations

aCGH: array comparative genomic hybridization; CNV: copy number variation; CNVR: copy number variation region; EM: expectation maximization; FDR: false discovery rate; Gb: giga base; GWAS: genome-wide association study; IRS: intensity rank-sum; Mb: megabase pairs; PCR: ploymerase chain reaction; RD: read depth; RP: read pair; SD: segmental duplication; SNP: single nucleotide polymorphism; SR: split read.

## Conflict of interest

The authors declare that they have no competing interests.

## Author contributions

W.X.H. and J.Y. designed the software; Z.Z.Q. and C.T. wrote the code; W.X.H. and Z.Z.Q. improved the pipeline structures; Z.Z.Q. and C.Y.D. tested the software prototype; L.C. and F.W.W. contributed to the data organization; and W.X.H. and J.Y. drafted the manuscript. All authors read and approved the final manuscript.

## Supplementary Material

GIGA-D-17-00119_Original_Submission.pdf

GIGA-D-17-00119_Revision_1.pdf

GIGA-D-17-00119_Revision_2.pdf

Response_to_Reviewer_Comments_Original_Submission.pdf

Response_to_Reviewer_Comments_Revision_1.pdf

Reviewer_1_Report_(Original_Submission).pdf

Reviewer_2_Report_(Original_Submission).pdf

Supplemental material
