# Supplementary material for: CNVcaller: highly efficient and widely applicable software for detecting copy number variations in large populations
Source: Gigascience. 2017 Dec 4;6(12):1–12. doi: 10.1093/gigascience/gix115 (PMC5751039; doi:10.1093/gigascience/gix115)
Supplement: GIGA-D-17-00119_Original_Submission.pdf [file gix115_giga-d-17-00119_original_submission.pdf]

GigaScience

CNVcaller: High efficient and Widely Applicable Software for Detecting Copy Number Variations in large Populations

--Manuscript Draft--

**Manuscript Number:**	GIGA-D-17-00119
**Full Title:**	CNVcaller: High efficient and Widely Applicable Software for Detecting Copy Number Variations in large Populations
**Article Type:**	Research
**Funding Information:**	National Natural Science Foundation of China (31572381) — Prof. Yu Jiang<br>National Thousand Youth Talents Plan — Prof. Yu Jiang
**Abstract:**	Background: The increasing sequencing data of a wide variety of species offers an opportunity for copy number variation (CNV) detection at population level. However, the growing sample size and the divergent complexity of non-human genomes challenge the efficiency and robustness of the current human-oriented CNV detection methods.<br>Result: Here we present CNVcaller, a read depth based method for CNVs discovering of the population sequencing data. By the statistics-based signal detection and population-level noise reduction algorithms, the detection for 232 goats with complicated genome assembly takes only 1.4 days on a single compute node. Besides, the false segmental duplications in reference genome assemblies can be mitigated by a simplified absolute copy number correction, which consumes only a few minutes and increases the sensitivity in CNV enriched regions. Multiple validations showed that CNVcaller achieved increased total sensitivity, high genotyping accuracy and low false discovery rate in human, livestock and crop populations.<br>Conclusion: The fast and general detection algorithms of CNVcaller overcome prior computational barriers for detecting CNV from large scale sequencing data with complicated genome structure. These advantages will promote the population genetic analysis of functional CNVs of more species.
**Corresponding Author:**	Yu Jiang, Ph.D<br>Northwest Agriculture and Forestry University<br>Yangling, Shaanxi CHINA
**Corresponding Author Secondary Information:**	
**Corresponding Author's Institution:**	Northwest Agriculture and Forestry University
**Corresponding Author's Secondary Institution:**	
**First Author:**	Xihong Wang
**First Author Secondary Information:**	
**Order of Authors:**	Xihong Wang
	Zhuqing Zheng
	Yudong Cai
	Ting Chen
	Chao Li
	Weiwei Fu
	Yu Jiang
**Order of Authors Secondary Information:**	
**Opposed Reviewers:**	

Powered by Editorial Manager® and ProduXion Manager® from Aries Systems Corporation

Additional Information:	
**Question**	**Response**
Are you submitting this manuscript to a special series or article collection?	No
**Experimental design and statistics**<br><br>Full details of the experimental design and statistical methods used should be given in the Methods section, as detailed in our Minimum Standards Reporting Checklist. Information essential to interpreting the data presented should be made available in the figure legends.<br><br>Have you included all the information requested in your manuscript?	Yes
**Resources**<br><br>A description of all resources used, including antibodies, cell lines, animals and software tools, with enough information to allow them to be uniquely identified, should be included in the Methods section. Authors are strongly encouraged to cite Research Resource Identifiers (RRIDs) for antibodies, model organisms and tools, where possible.<br><br>Have you included the information requested as detailed in our Minimum Standards Reporting Checklist?	Yes
**Availability of data and materials**<br><br>All datasets and code on which the conclusions of the paper rely must be either included in your submission or deposited in publicly available repositories (where available and ethically appropriate), referencing such data using a unique identifier in the references and in the "Availability of Data and Materials" section of your manuscript.<br><br>Have you have met the above requirement as detailed in our Minimum Standards Reporting Checklist?	Yes

Powered by Editorial Manager® and ProduXion Manager® from Aries Systems Corporation

Manuscript Click here to download Manuscript CNVcaller main.docx

CNVcaller: High Efficient and Widely Applicable Software for Detecting Copy Number Variations in large Populations

Xihong Wang [1†], Zhuqing Zheng[1†], Yudong Cai[1], Ting Chen[1], Chao Li[1], Weiwei Fu[1], Yu Jiang[1]*

1 College of Animal Science and Technology, Northwest A&F University, Yangling, Shaanxi, China

† These authors contributed equally to this work.

*Correspondence should be addressed to Yu Jiang (yu.jiang@nwafu.edu.cn).

Abstract

Background: The increasing sequencing data of a wide variety of species offers an opportunity for copy number variation (CNV) detection at population level. However, the growing sample size and the divergent complexity of non-human genomes challenge the efficiency and robustness of the current human-oriented CNV detection methods.

Result: Here we present CNVcaller, a read depth based method for CNV discovering of the population sequencing data. By the statistics-based signal detection and population-level noise reduction algorithms, the detection for 232 goats with complicated genome assembly takes only 1.4 days on a single compute node. Besides, the false segmental duplications in reference genome assemblies can be mitigated by a simplified absolute copy number correction, which consumes only a few minutes and increases the sensitivity in CNV enriched regions. Multiple validations showed that CNVcaller achieved increased total sensitivity, high genotyping accuracy and low false discovery rate in human, livestock and crop populations.

Conclusion: The fast and general detection algorithms of CNVcaller overcome prior

computational barriers for detecting CNVs from large scale sequencing data with complicated genome structure. These advantages will promote the population genetic analysis of functional CNVs of more species.

Keywords

copy number variation (CNV), next-generation sequencing (NGS), population genetics, segmental duplication, absolute copy number.

Introduction

Copy number variants (CNVs) are the prevalent and important source of genetic diversity [1], which are highly correlated with diseases [2, 3], evolutions [4] and other phenotypes [5-8] for all kinds of species. Over the development for decades, the large-scale sequencing projects have provided us with enormous amount of data across the tree of life. The geometric growing sample size enables the population genetics variant association studies using the CNV regions (CNVRs) integrated from multi-sample CNVs [9, 10]. However, the increasing data size aggravates computational burden and challenges the efficiency of the current CNV detectors. In addition, the complicated genome structure of many non-human species demands more robust signal detection and noise reduction algorithms.

Currently, several strategies are used to for CNV detecting of whole genome sequencing data: read-pair/split-read [11-14], local assembly [15-17] and read-depth (RD) [18-20]. Although employing multiple methods in one dataset can increase the total sensitivity [21], the efficiency and convenience would consequently become the subsequent concern. With the increasing release

of large-scale sequencing data, the population genetic information is applied to improve the detecting accuracy [22]. A typical strategy is to simultaneously scan the genomes of multiple samples, then decompose all signals into true variations and noises by priori distributions. Genome STRiP [23] is shown to be one of the best population-level CNV detectors in 1000 Genome Project [24].

Current human-oriented CNV detectors leave some uncertain points for application to the other species. Firstly, gaps and unplaced scaffolds are riddled with reference genome assemblies of most non-model organism [25, 26], leading to the increase of the abnormal mapping and the false positive rate of the read-pair/split-read algorithms. In comparison, the RD algorithm deduces copy number from the number of reads aligned to of a particular region, which can efficiently screen out noises by statistical hypothesis [18, 27]. In the RD based methods, CNVnator [19] which provides multi-sample genotyping function was used in yak, chicken and fish cohorts [28-30]. Secondly, the alternative alleles lead to high-proportioned erroneous segmental duplications (SDs) for the animal reference genomes [31, 32]. Therefore, intensive filtering of the duplicated regions on the reference genome is recommended by many CNV detectors. However, the SDs enrich CNVs 10 times than other area of the genome [4] and contribute to the evolutionary adaptive traits [33]. A more precise solution is deducing the absolute copy number from mrsFAST alignment which reports multiple hits of a single read [34]. However, precise realignment always requires enormous time, especially for the crop plant genomes which frequently contain large duplicated regions. Therefore, this strategy was hired in very few non-human CNV researches [35].

In this study, we introduce a super-fast and generally applicable method, CNVcaller, for

CNV discovering sequencing data of large populations. This software is based on the RD algorithm, and implies robust signal detection and noise deduction methods to increase the computational efficiency in all kinds of genomes. We applied it to the population sequencing data of human, livestock and crop to demonstrate the utility and benchmarked against the widely used and best practice CNV detectors.

Materials and Methods

Input data

The main input of CNVcaller are the alignment files in BAM format. The following data/samples were included in the validation: 30 BAM files of human from the 1000 Genome Project Phase 3 [36], including 27 normal (~ 12 X) and three deeply sequenced samples (~ 50 X); 30 BAM files of 10 families from the Genomes of Netherlands (GoNL) project [37] (~ 20 X); 70 FASTQ files of domestic sheep samples (~ 10 X) from the NCBI BioProject: PRJNA160933; two maize [38] and two soybeans [8] FASTQ files (each species contain one ~5 X and one ~10 X sample). An additional table showed the downloaded files in detail **(Supplementary Table1)**.

Another 63 sheep (~10 X) and 232 goats (~12 X) data were sequenced using pair-end libraries on the Illumina HiSeq 4000 platform. The FASTQ files were aligned to their respective reference assemblies using BWA 0.7.13 to generate BAM files [35]. The version of reference genomes are: human GRCh37, maize B73 RefGen_v3, soybean Glycine_max_v2.0, sheep OAR_v3.1 and goat ARS1. The GATK v3.5 [39] pre-processing workflow is used to produce analysis-ready BAM files. After alignment, PCR duplications were marked by Picard 2.1 and the

realignment was performed by GATK. The reads with 0x504 flag (indicating unmapped, secondary mapped or PCR duplication) were removed.

Individual RD processing

RD Estimation. The reference genome is segmented into overlapping sliding windows. For 5-10 X sequencing data, 800 bp windows with a 400 bp overlap is recommended. The sliding windows with gaps are excluded from the computation. The windows are indexed to form a reference database which will be used in all samples. The BAM file of each individual is parsed out using SAMtools v1.3 [40]. The raw RD signal is calculated for each window as the number of placed reads with centers within window boundaries. This step consumes less than 500 Mb max memory for one BAM file, so parallel submitting is recommended.

Absolute copy number correction. The standard mapping only aligns one sequencing read to one best position of the genome. For the regions with more than one assembled copy in reference genome, the reads will be split among the copies. Therefore, the deduced copy numbers are dependent to the segment number in reference genome, which is called relative copy number. CNVcaller implies a simple correction to deduce the copy number independent to the reference genome, which was called absolute copy number.

To perform the absolute copy number correction, the windows with >97% sequence similarity are linked together to form a duplicated window record file before correction. This file can be generated by splitting the reference genome into non-overlapping windows and aligning them onto the reference genome using the precise aligner, e.g. BLAT v. 36X1 [41]. The windows with

more than 20 hits are excluded to remove the low complexity regions. The record files of human, livestock and main crops can be downloaded from the CNVcaller website.

Based on the duplicated window record file, the raw RD located on similar windows are added together to generate the absolute RD for all the high similarity windows.

$$RD_{absolute}^{i} = \sum_{j=1}^{t} RD_{raw}^{ij}$$

Where i is the index of the window to be corrected, t is the total number of the high similarity windows. RD_{raw}^{ij} is the raw RD of the window similar with the i-th window (including the i-th window itself), which is counted directly from the BWA alignment, and $RD_{absolute}^{i}$ is the corrected RD of the i-th window which can be used to deduce the absolute copy number.

GC correction and normalization. Since the resequencing samples may show various GC content distribution, the GC bias is corrected individually basically as CNVnator [19] except using the RD of the windows with 40% GC as standard:

$$RD_{corrected}^{i} = \frac{\overline{RD}_{40}}{\overline{RD}_{gc}} RD_{absolute}^{i}$$

Where i is the window index, $RD_{absolute}^{i}$ is the RD after absolute copy number correction, $RD_{corrected}^{i}$ is final corrected RD for the window, $\overline{RD}_{40}$ is the mean RD of windows with 40% percent GC as standard, and $\overline{RD}_{gc}$ is the mean RD over all windows that have the same GC content with the i-th window.

Assuming that the majority part of the genome is in normal copy number, the corrected RDs are divided by the global median RD to normalized to one. For the sex chromosomes, if the median RD of the homogametic sex chromosomes (X or Z) is about half of the median RD of autosome,

the RDs on the X or Z chromosome are doubled before normalization.

CNVR detection by multiple criteria

Individual candidate CNV window definition. The individual candidate CNV windows are defined using two criteria: (1) The normalized RD is significantly higher or lower than the normalized mean RD (deletions < 1 – 2 * STDEV; duplications > 1 + 2 * STDEV). (2) Considering the normalized RD of heterozygous deletions and duplications should be around 0.5 and 1.5 respectively, an empirical standard for the normalized RD (deletions < 0.65; duplications > 1.35) also need to be achieved. For some strictly self-bred species, such as soybean and wheat, this empirical standard should be raised to 0.25 or 1.75 for the normalized RD of the homozygous deletions or duplications respectively.

Population-level candidate CNV window definition. All individual RD files are piled up by the universal window index to a two-dimensional population RD file. The window showing high frequency of individually candidate CNV (allele frequency >0.05) or have at least three homozygous duplicated/deleted individuals in large population are selected. Then Pearson's product–moment correlation coefficients of the multi-sample RDs are calculated between the two adjacent non-overlapping windows. Only the windows with significant correlation ($P<0.01$ by T test) are selected and merged into one call.

CNV region definition. Initial calls are selected if more than four sequential 800 bp overlapped

windows (total length >=2,000 bp) are defined as the population-level candidate windows. To tolerant noises, at most one unselected window out of four continuous candidate windows is allowed to exist. Then the two adjacent initial calls are further merged if their copy numbers are highly correlated and the distance between them is less than a certain percent of their own length.

CNVR Genotyping

The copy number of a specific sample is initially estimated by two times the median RD of all the candidate windows in this region. The deleted and biallelic duplicated CNVRs (average copy number <= 4) will be clustered by a constrained mixture Gaussian model embedded in CNVcaller. This model presets the average copy number of homozygous deletion, heterozygous deletion, normal, heterozygous deletion and homozygous deletion at zero to four respectively. For multiallelic CNVRs (average copy number >4) we provide a clustering process by unsupervised mixture Gaussian model (calling R package mclust 5.2 [42]). In a population, the calls with the same copy number in all samples are defined as SDs while the polymorphic calls are defined as CNVRs. The output CNVR genotyping file is analyzable by the population genetic algorithms.

Performance evaluation

Competing methods. Most validations were based on the 30 human BAM files from the 1000 Genome Project Phase 3 unless otherwise noted. The performance of CNVcaller was compared with two pipelines: CNVnator_v0.3.3 [19] which was well-used in animal population CNVR

detection and Genome STRiP (included in svtoolkit_2.00.1696) [23] which was the state-of-the-art human population CNV detector. The recommended parameters and QC filters were used. For Genome STRiP, both the deletion and CNV pipelines were performed. The unplaced scaffolds were removed from the reference genome and the whole genome was separated by chromosome as recommended. The standard screens were applied to select passing sites and remove duplicated calls. For CNVnator, the gap regions and calls with p values less than 0.01 were removed. We also used the q0 filter to remove any predictions with q0 <0.5 (reads with multiple mapping locations) as recommend. The individual CNVs of all sample were merged in to the population CNVRs by the arbitrary standards: two calls have >50% reciprocal overlapping with each other or >90% of on one call is covered by another call. Then the CNVRs were genotyped by the built-in function of CNVnator. Because the three software have different limitations in CNV detection, only the CNVRs on autosomes with >2,000 bp length and allele frequency >=0.05 were used in the following validation.

Sensitivity validation. Sensitivity was calculated as the proportion of high-confident CNVR database overlapped by predicted CNVRs. Two previously published database including the same samples in the test data were used. One is the 1000 Genome Project CNVR map [24] included 26 tested samples, the other is the array comparative genomic hybridization, (aCGH) based CNVR database [43] included 10 tested samples. The CNVRs of the specific samples were extracted from the database then screened by the same length and frequency as detected CNVRs (length >2,000 bp and alternative allele frequency >=0.05). The intersected length of the predicted CNVRs and the high-confident CNVR database were calculated by the bedtools v2.25.0 [44].

Accuracy validation using human database. The intensity rank-sum (IRS) test (included in the svtoolkit_2.00.1696) was performed based on the intensity data of the Affymetrix SNP 6.0 array including 26 test samples. SD regions were removed as [23] because the probe design does not cover the high similarity regions. The genotyping accuracy were calculated based on the aCGH CNVR database [43]. For a detected CNVR has >90% overlap with the database, the predicted copy number showed exact agreement with the integer genotyping from aCGH database were defined as correct. The Mendelian inconsistencies were calculated from the deleted and biallelic duplicated CNVRs (average copy number < 4) in the Dutch families and sheep trios.

Sheep genotyping validation by CNVplex assay. A total of 73 sheep including Merino, Texel, Mongolia and Tibetan sheep were used for genotyping validation. Genomic DNA was extracted from the peripheral blood using the QIAamp DNA blood mini kit (Qiagen, Germany). ~10 X resequencing was performed for each sheep and the CNVRs were detected by CNVcaller as described above. The predicted CNVRs with high variation frequency were selected for the validation. The copy numbers were validated by CNVplex® (Genesky Biotechnologies Inc., Shanghai, China), which is based on double ligation and multiplex fluorescence PCR [45]. The probes were designed to target the candidate windows of the target CNVR. The sizes of the PCR fragments and target loci sequences in each reaction are listed in **Supplementary Table 2.** Amplified probes were detected as fluorescent signals and peak areas were compared and normalized to determine the dosage of each target.

Absolute copy number validation

Putative X-linked scaffolds. All the scaffolds of OAR v3.1 were mapped to the X chromosome of sheep reference genome OAR v4.0, goat reference genome ARS1, and cattle reference genome UMD 3.1 using BLASR [46]. If the best hit of a scaffold had a coverage >50% with >90% identity and >3 Kb length, this scaffold was defined as the putative X-linked scaffold.

mrsFAST alignment. The pair-end reads with multiple hits indicated by the XA tag in BWA alignment were selected to realign by mrsFAST_v3.3.10 [34]. The mrsFAST alignment was performed basically as previously described [47]. Longer reads were trimmed into 40 bp to reduce the read length heterogeneity prior to sequence alignments. After alignment, the reads with more than 20 mapped hits were excluded to remove the low complexity regions.

Results

Overview of CNVcaller algorithm

CNVcaller pipeline includes three main steps **(Figure 1)**. First, considering the population sequencing data may come from different platforms, the RD of each sample is counted and corrected individually. An original absolute copy number correction is used to modify the standard read alignments generated by BWA software to multi-hit alignments, as similar to mrsFAST format **(Supplementary Figure 1)**. This correction takes only 0.06 core-hour for a mammalian genome with 10 X sequencing coverage, while 10 core-hours are needed for remapping the reads by mrsFAST. After corrections and normalization, the comparable RDs of each sample is

concentrated to a ~100 Mb intermediate file and output. This design avoids repeat calculation of a same individual in different populations, and save much time since the individual step consumes more than 80% of the total running time.

In the second CNVR detection step, the RD files of all samples are piled up into a two-dimensional population RD file. Multi-criteria are implied to remove the high-proportional noise caused by low sequencing quality or assembly bias. Individually, the RD of the candidate CNV window should significantly deviates from average. The piled-up candidate windows should also meet two population-level criteria: CNV allele frequency > 5% and the multi-sample RDs of adjacent windows are significantly correlated **(Figure 1)**. Compared with intensified individual RD screening, the multi-criteria filtering preserves heterozygous CNVs with half RD value of the homozygous CNVs.

After merging the candidate CNV windows into a CNVR, the RDs of all samples in each CNVR are clustered by the mixture Gaussian model and deducing the integer copy number of each individual. This step is called genotyping as used in SNP detection. The final output is compatible with most SNP based population genetic algorithm.

Computational cost

The robustness of CNVcaller was validated by the real sequencing data of different genomes. The individual RD processing step of CNVcaller was compared against CNVnator, which also detects CNVs individually. The processing time of CNVcaller was linear related to the genome size and sequencing coverage: 20-40 minutes for a 3 Gb genome with 10 X coverage **(Supplementary Table 3)**. However, the processing time of CNVnator rose exponentially with the scaffold number,

which became the only index of time consuming when the scaffold number exceeds one thousand **(Figure 2A)**. Consequently, CNVcaller achieved 145 fold speed increasing over CNVnator for goat CNV detection. Noteworthy, the goat reference genome ARS1 which contains nearly 30 thousand scaffolds was newly assembled by single-molecule sequencing platform [48].

The memory requirement of CNVcaller is extremely low and mainly related to the genome size: only about 500 Mb for a mammalian genome, which was less than one twentieth of CNVnator **(Figure 2B)**. Therefore, in multi-sample CNV detection, more than 20 missions of the individual RD processing step can be run in parallel on one node to further increase the population CNVR detection efficiency. The population-level performance of CNVcaller was evaluated and benchmarked by Genome STRiP which also detects CNVRs at population level. After removing the unplaced scaffolds, CNVcaller was still 3.5-7.8 times faster than Genome STRiP **(Figure 2C)**, with 70% ~86% reduction in memory requirement **(Figure 2D)**. The CNV detection of 232 goats with mean 12 X coverage can be completed in 1.4 days by CNVcaller on one node.

Sensitivity and accuracy

A total of 1,058 CNVRs with a total length of 24.5 Mb were detected by CNVcaller from a 30 human cohort, 20% longer than CNVnator, and twice of Genome STRiP. CNVcaller covered 43% of the CNVRs detected by CNVnator, 65% of Genome STRiP and 76% of their intersection **(Figure 3A)**, indicating CNVcaller has higher sensitivity for the cross validated CNVRs. We also compared the CNVRs identified by CNVcaller from worldwide 133 sheep of 44 breeds with the other two recently released large scale sheep CNVR datasets. One is derived from a small pedigrees using multiple platforms including aCGH, SNP chip and whole genome sequence [49],

the other is based on three Chinese sheep breeds and 600K SNP array [50]. Although based on different technologies and breeds, CNVcaller still covers 51% of their intersection **(Figure 3B)**.

The 1000 Genome Project samples with experimental validated CNV database was mainly used to evaluate the sensitivity and accuracy of CNVcaller and other two methods. The sensitivity was estimated as the proportion of high-confident CNV database overlapped by predicted CNVs **(Table 1)**. Based on the aCGH database [43], the sensitivity of CNVcaller was 13%-18% higher than the other two methods. Even based on the 1000 Genome Project CNV maps which was constructed by multiple methods including the Genome STRiP and CNVnator [24], CNVcaller still achieved higher sensitivity than other software.

False discovery rate (FDR) on human genome was estimated by multiple strict sample specific methods **(Table 1)**. (1) IRS test based on the intensity data of the SNP array; (2) the integer copy numbers in aCGH database; (3) the Mendelian inconsistencies from 10 Dutch families. For the three CNV detectors, Genome STRiP achieved the best accuracy (0.8% - 3.9%) in all the human based validations, and a little higher Mendelian inconsistencies (5.2%) in the three sheep trios. CNVcaller had median FDR (2.8% - 5.4%) in human validations, however, it achieved the best accuracy (2.4% Mendelian inconsistencies) in the three sheep trios, indicating its superiority in non-human genomes. To evaluate the genotyping accuracy, we turned to a recently developed molecular biology technique, CNVplex, which counts the copy number of a genomic sequence based on the multiplex ligation-dependent probe amplification (MLPA) method [45]. When we compared the copy numbers predicted by CNVcaller from sequencing data and the CNVplex result, the Pearson's product–moment correlation coefficients were higher than 0.95, and the integer genotype concordance was 98% **(Figure 4)**.

The absolute copy number correction in duplicated region of the reference genome

Compared with human, the sheep sample had much lower copy numbers in putative duplicated regions than expected **(Figure 5A)**, indicating the sequencing reads were split among the false duplications as previous reported on other animal genome assemblies [31]. This bias leaded the raw copy number distribution of the putative two-copy segment duplications peak at one **(Figure 5B)**, thus most of these windows were likely to be mistaken for heterozygote deletions. After absolute copy number correction **(Figure 1)**, the copy number distribution was more reasonable: the main peak shift to around two (normal biallelic copy number), and the smaller peaks around one and four indicated the detectable heterozygous deletions and duplications respectively **(Figure 5B)**. Because this correction preserves the complicated regions and the multi-hit reads, CNVcaller increased the sensitivity of SD region about six time than the other two methods. Moreover, it is more reasonable for most CNVs in SD regions were genotyped as duplications instead of deletions **(Figure 5C)**.

The detection and genotyping accuracy in SD region were further estimated by the sex information of 133 sheep. We first defined 138 unplaced scaffolds with high sequence similarity to X chromosome as X-linked scaffolds. In theory, all these scaffolds were expected to be detected as high frequency CNVRs because the RDs of unplaced scaffolds were not corrected by sex. CNVcaller detected 101 out of the 138 X-linked scaffolds with a sensitivity of 73%, while CNVnator did not catch any of these regions. The corrected copy number of these scaffold centralized at one and two in rams and doubled in ewes indicating the unique and duplicated X-

linked regions. However, the peaks of raw copy numbers were ambiguous and not at integer **(Figure 5D)**. Further examination of the duplicated regions showed the higher divergence was caused by splitting of raw RDs among the mistaken-assembly segments **(Supplementary Figure 2)**.

Discussion

CNVcaller was designed to detect the CNVRs from large scale resequencing data of all kinds of genomes. It takes full consideration of the complexity of genome, and implies several general applicable signal detection and noise deduction algorithms to increase the computational efficiency. The detection of 232 individuals can be complete on one compute note within two days, and this speed up does not compromise the accuracy. Meanwhile, validated through multiple rigorous assessment, CNVcaller increased the sensitivity by 13%-18% with a low FDR in human and non-human species.

The statistics-based detection algorithm of CNVcaller assumes a true CNV signal can be interrupted with high ratio of noise, while the segmentation algorithms assume that the RD signal is basically piecewise constant in the genome sequence. The formal model is universally applicable to high quality as well as fragmented genomes. Therefore, the speed of CNVcaller is still fast with thousands of scaffolds, which is common in non-human reference genome assemblies. The robustness of CNVcaller reduces the restrictions of the reference genome, which will promote CNV research of the species with only scaffold level reference genome assemblies. More importantly, this feature enables the comprehensive variation discoveries using multiple

assemblies or pan-genomes. Defined as the entire set of genes possessed by all members of a particular species, pan-genomes reveal numerous functional important genes unplaced on one single reference genome [50-52]. However, its complexity and diversity hinder the application of almost all CNV detectors. Up on our unpublished data, CNVcaller is efficient and friendly to detect the present/absent variations for pan-genomes.

Another optimization of CNVcaller is the simplified absolute copy number correction. Although absolute copy number can reduce the bias of misassembled duplications in non-human genomes, conventional solution requires mapping the sequencing reads of each individual by time-consuming precise aligner. CNVcaller simplifies the calculation by generating a duplicated window record file through one precise alignment of the reference genome (the duplicated window record file of the latest reference genome of human, livestock and main crops can be downloaded from website), then the standard alignment of all samples can be corrected with high speed. In this way, the misassembled duplications can be mitigated with a great reduction of running time. Validation of sheep genomes showed this correction multiplied the detection efficiency of SD regions, and deduced the more reasonable copy numbers.

Several limitations still exist in CNVcaller pipeline. First, population level criteria are used to screen out the low frequency and uncorrelated windows. Therefore, CNVcaller is not suitable for detection of rare CNVs, however, the influence is much less on GWAS which also has low power to capture rare functional variants [51]. Besides, the RD algorithm has disadvantage in short CNV detection and breakpoint definition. The visual examination for the specific interval using IGV [52] or combined with other CNV detection methods can improve the results. In the latter situation, CNVcaller can provide high-confidence RD information as solid prior for the read

pair/split read pipeline [53].

In summary, CNVcaller offers a fast and robust pipeline to detect CNVRs from population-scale resequencing data. The high computational efficiency reduces the hardware requirements and facilitates the CNVR detection of large populations. The general applicable detection and correction algorithms have greatly increased the sensitivity in non-model species and enabled the CNV detection for a wide range of species. The rapid and reliable population-level CNV detection will promote the discovery of the missing heritability of complex traits and accurately determination of the causative mutations for more species.

Availability and requirements

Project name: CNVcaller

Project home page: http://animal.nwsuaf.edu.cn/software

https://github.com/JiangYuLab/CNVcaller

Operating system(s): platform independent

Programming language: Perl, C++

Other requirements: Samtools 1.3 (using htslib 1.3)

License: GNU General Public License, version 3.0 (GPL-3.0)

Conflict of interest

The authors declare that they have no competing interests

Author contributions

WXH and JY designed this software; ZZQ and CT wrote the code; WXH and ZZQ improved the pipeline structures; ZZQ and CYD tested the software prototype; LC and FWW contributed to the data organization; WXH and JY drafted the manuscript. All authors read and approved the final manuscript.

Acknowledgements

This work is supported by grants from National Natural Science Foundation of China (31572381), and the National Thousand Youth Talents Plan. We thank the International Sheep Genomics Consortium (ISGC) for access to the unpublished sheep sequencing data provided under the Toronto guidelines for data users. We thank for the Genomes of Netherlands (GoNL) project for the human family data.

References

1. Chiang C, Scott AJ, Davis JR, Tsang EK, Li X, Kim Y, et al. The impact of structural variation on human gene expression. Nature genetics. 2017.
2. Stefansson H, Meyer-Lindenberg A, Steinberg S, Magnusdottir B, Morgen K, Arnarsdottir S, et al. CNVs conferring risk of autism or schizophrenia affect cognition in controls. Nature. 2014;505 7483:361-6.
3. Khurana E, Fu Y, Colonna V, Mu XJ, Kang HM, Lappalainen T, et al.

Integrative annotation of variants from 1092 humans: application to cancer genomics. Science. 2013;342 6154:1235587.

4. Sudmant PH, Mallick S, Nelson BJ, Hormozdiari F, Krumm N, Huddleston J, et al. Global diversity, population stratification, and selection of human copy-number variation. Science. 2015;349 6253:aab3761.

5. Norris BJ and Whan VA. A gene duplication affecting expression of the ovine ASIP gene is responsible for white and black sheep. Genome research. 2008;18 8:1282-93.

6. Wright D, Boije H, Meadows JR, Bed'Hom B, Gourichon D, Vieaud A, et al. Copy number variation in intron 1 of SOX5 causes the Pea-comb phenotype in chickens. PLoS Genet. 2009;5 6:e1000512.

7. Seo B-Y, Park E-W, Ahn S-J, Lee S-H, Kim J-H, Im H-T, et al. An accurate method for quantifying and analyzing copy number variation in porcine KIT by an oligonucleotide ligation assay. BMC genetics. 2007;8 1:81.

8. Zhou Z, Jiang Y, Wang Z, Gou Z, Lyu J, Li W, et al. Resequencing 302 wild and cultivated accessions identifies genes related to domestication and improvement in soybean. Nature biotechnology. 2015;33 4:408-14.

9. Craddock N, Hurles ME, Cardin N, Pearson RD, Plagnol V, Robson S, et al. Genome-wide association study of CNVs in 16,000 cases of eight common diseases and 3,000 shared controls. Nature. 2010;464 7289:713-20. doi:10.1038/nature08979.

10. Xu L, Cole JB, Bickhart DM, Hou Y, Song J, VanRaden PM, et al. Genome wide CNV analysis reveals additional variants associated with milk production traits in

Holsteins. BMC genomics. 2014;15:683. doi:10.1186/1471-2164-15-683.

11. Chen K, Wallis JW, McLellan MD, Larson DE, Kalicki JM, Pohl CS, et al. BreakDancer: an algorithm for high-resolution mapping of genomic structural variation. Nat Meth. 2009;6 9:677-81.

12. Hormozdiari F, Hajirasouliha I, Dao P, Hach F, Yorukoglu D, Alkan C, et al. Next-generation VariationHunter: combinatorial algorithms for transposon insertion discovery. Bioinformatics. 2010;26 12:i350-i7. doi:10.1093/bioinformatics/btq216.

13. Ye K, Schulz MH, Long Q, Apweiler R and Ning Z. Pindel: a pattern growth approach to detect break points of large deletions and medium sized insertions from paired-end short reads. Bioinformatics. 2009;25 21:2865-71. doi:10.1093/bioinformatics/btp394.

14. Rausch T, Zichner T, Schlattl A, Stütz AM, Benes V and Korbel JO. DELLY: structural variant discovery by integrated paired-end and split-read analysis. Bioinformatics. 2012;28 18:i333-i9.

15. Chen K, Chen L, Fan X, Wallis J, Ding L and Weinstock G. TIGRA: A targeted iterative graph routing assembler for breakpoint assembly. Genome research. 2014;24 2:310-7. doi:10.1101/gr.162883.113.

16. Zerbino DR and Birney E. Velvet: Algorithms for de novo short read assembly using de Bruijn graphs. Genome research. 2008;18 5:821-9. doi:10.1101/gr.074492.107.

17. Zhuang J and Weng Z. Local sequence assembly reveals a high-resolution profile of somatic structural variations in 97 cancer genomes. Nucleic Acids Res.

2015;43 17:8146-56. doi:10.1093/nar/gkv831.

18. Yoon S, Xuan Z, Makarov V, Ye K and Sebat J. Sensitive and accurate detection of copy number variants using read depth of coverage. Genome research. 2009;19 9:1586-92.

19. Abyzov A, Urban AE, Snyder M and Gerstein M. CNVnator: an approach to discover, genotype, and characterize typical and atypical CNVs from family and population genome sequencing. Genome research. 2011;21 6:974-84.

20. Szatkiewicz JP, Wang W, Sullivan PF, Wang W and Sun W. Improving detection of copy-number variation by simultaneous bias correction and read-depth segmentation. Nucleic acids research. 2013;41 3:1519-32.

21. Wang M, Tu L, Lin M, Lin Z, Wang P, Yang Q, et al. Asymmetric subgenome selection and cis-regulatory divergence during cotton domestication. Nature genetics. 2017.

22. Klambauer G, Schwarzbauer K, Mayr A, Clevert D-A, Mitterecker A, Bodenhofer U, et al. cn. MOPS: mixture of Poissons for discovering copy number variations in next-generation sequencing data with a low false discovery rate. Nucleic acids research. 2012:gks003.

23. Handsaker RE, Van Doren V, Berman JR, Genovese G, Kashin S, Boettger LM, et al. Large multiallelic copy number variations in humans. Nature genetics. 2015;47 3:296-303.

24. Sudmant PH, Rausch T, Gardner EJ, Handsaker RE, Abyzov A, Huddleston J, et al. An integrated map of structural variation in 2,504 human genomes. Nature.

2015;526 7571:75-81.

25. Claros MG, Bautista R, Guerrero-Fernández D, Benzerki H, Seoane P and Fernández-Pozo N. Why assembling plant genome sequences is so challenging. Biology. 2012;1 2:439-59.

26. Warr A, Hume D, Archibald AL, Deeb N and Watson M. Identification of low-confidence regions in the pig reference genome (Sscrofa10. 2). Frontiers in genetics. 2015;6:338.

27. Xie C and Tammi MT. CNV-seq, a new method to detect copy number variation using high-throughput sequencing. BMC bioinformatics. 2009;10 1:80.

28. Zhang X, Wang K, Wang L, Yang Y, Ni Z, Xie X, et al. Genome-wide patterns of copy number variation in the Chinese yak genome. BMC genomics. 2016;17 1:1.

29. Chain FJ, Feulner PG, Panchal M, Eizaguirre C, Samonte IE, Kalbe M, et al. Extensive copy-number variation of young genes across stickleback populations. PLoS Genet. 2014;10 12:e1004830. doi:10.1371/journal.pgen.1004830.

30. Yi G, Qu L, Liu J, Yan Y, Xu G and Yang N. Genome-wide patterns of copy number variation in the diversified chicken genomes using next-generation sequencing. BMC genomics. 2014;15:962. doi:10.1186/1471-2164-15-962.

31. Kelley DR and Salzberg SL. Detection and correction of false segmental duplications caused by genome mis-assembly. Genome biology. 2010;11 3:1.

32. Zimin AV, Kelley DR, Roberts M, Marçais G, Salzberg SL and Yorke JA. Mis-Assembled “Segmental Duplications” in Two Versions of the <italic>Bos taurus</italic> Genome. PLoS One. 2012;7 8:e42680.

doi:10.1371/journal.pone.0042680.
33. Dennis MY, Harshman L, Nelson BJ, Penn O, Cantsilieris S, Huddleston J, et al. The evolution and population diversity of human-specific segmental duplications. Nature Ecology & Evolution. 2017;1:0069.
34. Hach F, Hormozdiari F, Alkan C, Hormozdiari F, Birol I, Eichler EE, et al. mrsFAST: a cache-oblivious algorithm for short-read mapping. Nature methods. 2010;7 8:576-7.
35. Liu GE, Hou Y, Zhu B, Cardone MF, Jiang L, Cellamare A, et al. Analysis of copy number variations among diverse cattle breeds. Genome research. 2010;20 5:693-703.
36. Zarrei M, MacDonald JR, Merico D and Scherer SW. A copy number variation map of the human genome. Nature Reviews Genetics. 2015.
37. Consortium GotN. Whole-genome sequence variation, population structure and demographic history of the Dutch population. Nature genetics. 2014;46 8:818-25.
38. Diez CM, Meca E, Tenaillon MI and Gaut BS. Three groups of transposable elements with contrasting copy number dynamics and host responses in the maize (Zea mays ssp. mays) genome. PLoS Genet. 2014;10 4:e1004298.
39. McKenna A, Hanna M, Banks E, Sivachenko A, Cibulskis K, Kernytsky A, et al. The Genome Analysis Toolkit: a MapReduce framework for analyzing next-generation DNA sequencing data. Genome Res. 2010;20 9:1297-303. doi:10.1101/gr.107524.110.
40. Li H, Handsaker B, Wysoker A, Fennell T, Ruan J, Homer N, et al. The

Sequence Alignment/Map format and SAMtools. Bioinformatics. 2009;25 16:2078-9. doi:10.1093/bioinformatics/btp352.

41. Kent WJ. BLAT—the BLAST-like alignment tool. Genome research. 2002;12 4:656-64.

42. Scrucca L, Fop M, Murphy TB and Raftery AE. mclust 5: Clustering, classification and density estimation using gaussian finite mixture models. The R Journal. 2016;8 1:289.

43. Conrad DF, Pinto D, Redon R, Feuk L, Gokcumen O, Zhang Y, et al. Origins and functional impact of copy number variation in the human genome. Nature. 2010;464 7289:704-12.

44. Quinlan AR and Hall IM. BEDTools: a flexible suite of utilities for comparing genomic features. Bioinformatics. 2010;26 6:841-2.

45. Zhang X, Xu Y, Liu D, Geng J, Chen S, Jiang Z, et al. A modified multiplex ligation-dependent probe amplification method for the detection of 22q11. 2 copy number variations in patients with congenital heart disease. BMC genomics. 2015;16 1:364.

46. Chaisson MJ and Tesler G. Mapping single molecule sequencing reads using basic local alignment with successive refinement (BLASR): application and theory. BMC bioinformatics. 2012;13 1:238.

47. Bickhart DM, Hou Y, Schroeder SG, Alkan C, Cardone MF, Matukumalli LK, et al. Copy number variation of individual cattle genomes using next-generation sequencing. Genome research. 2012;22 4:778-90.

48. Bickhart DM, Rosen BD, Koren S, Sayre BL, Hastie AR, Chan S, et al. Single-molecule sequencing and chromatin conformation capture enable de novo reference assembly of the domestic goat genome. Nature Genetics. 2017;49 4:643-50.

49. Jenkins GM, Goddard ME, Black MA, Brauning R, Auvray B, Dodds KG, et al. Copy number variants in the sheep genome detected using multiple approaches. BMC genomics. 2016;17 1:1.

50. Zhu C, Fan H, Yuan Z, Hu S, Ma X, Xuan J, et al. Genome-wide detection of CNVs in Chinese indigenous sheep with different types of tails using ovine high-density 600K SNP arrays. Scientific reports. 2016;6.

51. Robinson MR, Wray NR and Visscher PM. Explaining additional genetic variation in complex traits. Trends in Genetics. 2014;30 4:124-32.

52. Thorvaldsdóttir H, Robinson JT and Mesirov JP. Integrative Genomics Viewer (IGV): high-performance genomics data visualization and exploration. Briefings in bioinformatics. 2013;14 2:178-92.

53. Layer RM, Chiang C, Quinlan AR and Hall IM. LUMPY: a probabilistic framework for structural variant discovery. Genome biology. 2014;15 6:R84.

Figure Legends

Figure 1 CNVcaller algorithm flowchart (left) and the key algorithms of each step (right). (1) Individually RD processing. In the absolute copy number correction, the RDs of high similar windows are added together to deduce the absolute copy number. (2) Multi-criteria CNVR selection. Curves show copy numbers in a specific region for multiple samples. Blue transverse

boxes mark the windows with significantly distinguish copy number from the average (individual criterion). Green vertical boxes indicate regions with the CNV allele frequency >5% in a specific region, and red frame indicates the RDs between two adjacent windows are significantly correlated (population criteria). Only the region with continuous high CNV allele frequency and high correlation (the forth bar from the left) are selected as the CNVRs. (3) Genotyping: The copy numbers in each CNVR are clustered by mixture Gaussian model to distinguish the normal, heterozygous and homozygous samples.

Figure 2 Computational performance of CNVcaller, CNVnator and Genome STRiP. All the programs were executed on one node with two 2.40-GHz Intel Xeon E5-2620 v3 processors. (A, B) Log plots of processing time (A) and max memory (B) for one individual. The numbers of unplaced scaffolds of the reference genome are indicated in brackets. The processing time was normalized by genome size and sequencing coverage to simulate a 3 Gb genome with 5 X or 10 X sequencing coverage. (C, D) Log plots of total running time (C) and max memory (D) of population CNVR detection. The test cohorts are: 8 sheep, 30 humans and 232 goats with 19 X, 16 X and 12 X average sequencing coverage respectively. In Genome STRiP running, the unplaced scaffolds were removed from the reference genome.

Figure 3 Overlap of the CNVRs detected by CNVcaller and other approaches/platforms (A) Intersection of the CNVRs detected by CNVcaller, CNVnator and Genome STRiP based on the same 30 human data. (B) Intersection of the CNVRs detected by CNVcaller and two other large scale sheep CNVR studies.

Figure 4 Evaluation of CNV genotypes by CNVplex. Two duplicated (A, B) and two deleted (C, D) CNVRs with high variation frequency were typed in CNVplex in 73 sheep samples. The copy number genotypes predicted by CNVcaller from sequencing data were plotted against the measurements from CNVplex of the same animal.

Figure 5 Absolute copy number correction in sheep genome. (A) The copy numbers of all windows with no more than six repeats were plotted against the repeat numbers in reference genome. (B) Distribution of copy numbers of two-copy loci in sheep genome before and after absolute copy number correction. (C) The detected CNVRs resided in SD regions. The sheep SD regions include the regions longer than 2 Kb with >97% identity. The CNVRs resided in SD regions were defined if more than 50% of this CNVR was overlapped with the SD regions. (D) The raw and corrected copy numbers of all X-linked scaffolds of 133 sheep.

Table 1. Sensitivity and FDR of CNVcaller, CNVnator and Genome STRiP.

Methods	Estimated sensitivity		Estimated FDR			
	aCGH	1000 GP	IRS	CGH Genotype	Mendelian error Human*	Mendelian error Sheep*
CNVcaller	45.4%	56.1%	4.1%	5.4%	2.8%	2.4%
CNVnator	32.6%	51.7%	11.4%	5.4%	5.5%	3.7%
Genome STRiP	27.2%	50.4%	3.9%	2.2%	0.8%	5.2%

*The Mendelian errors in human and sheep were calculated based on 10 Dutch families and three sheep trios respectively. Other evaluations were based on 30 human BAM files downloaded from 1000 genome project.

Figure1

Click here to download Figure Figure1.pdf

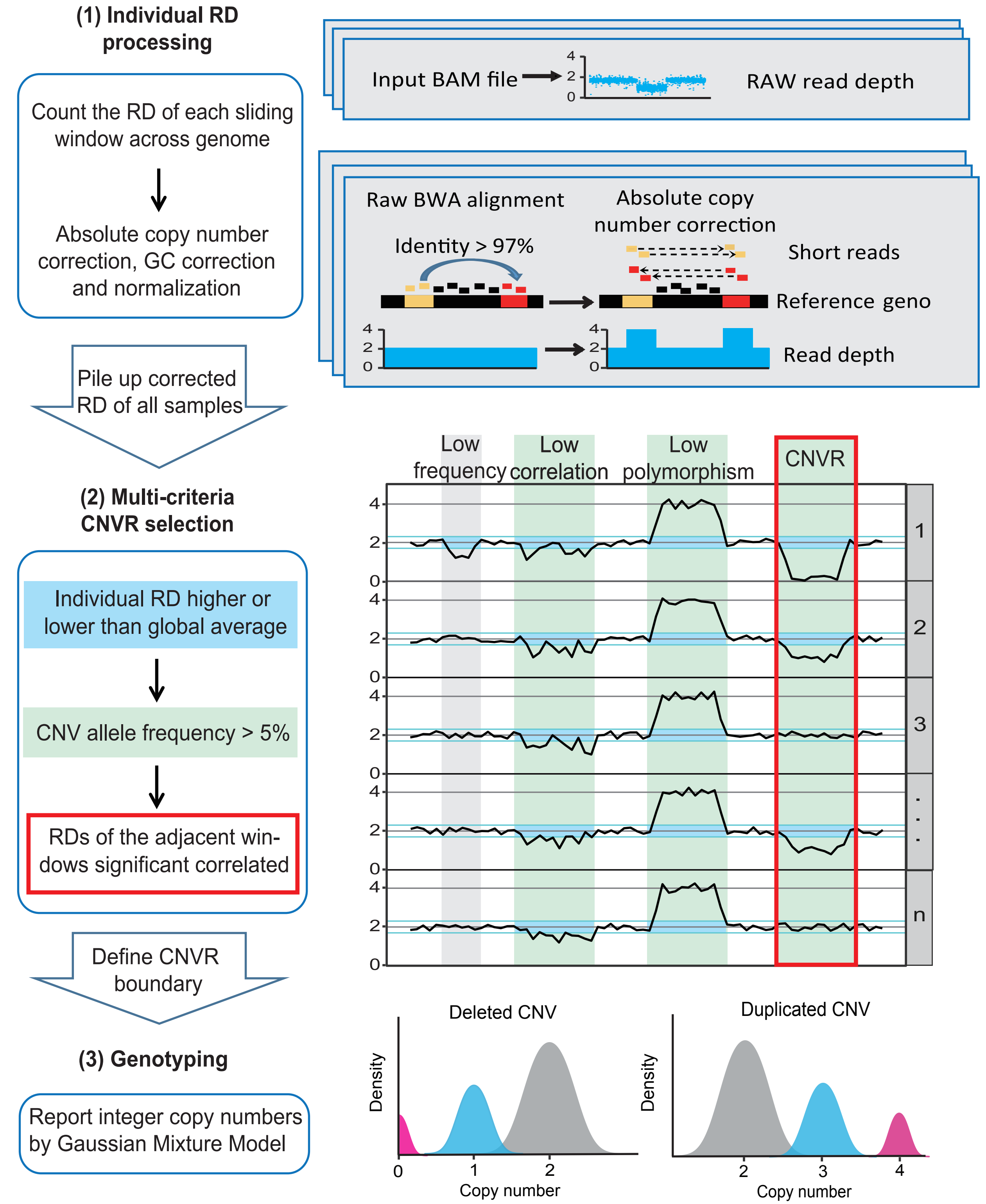

Figure2

Click here to download Figure Figure2.pdf

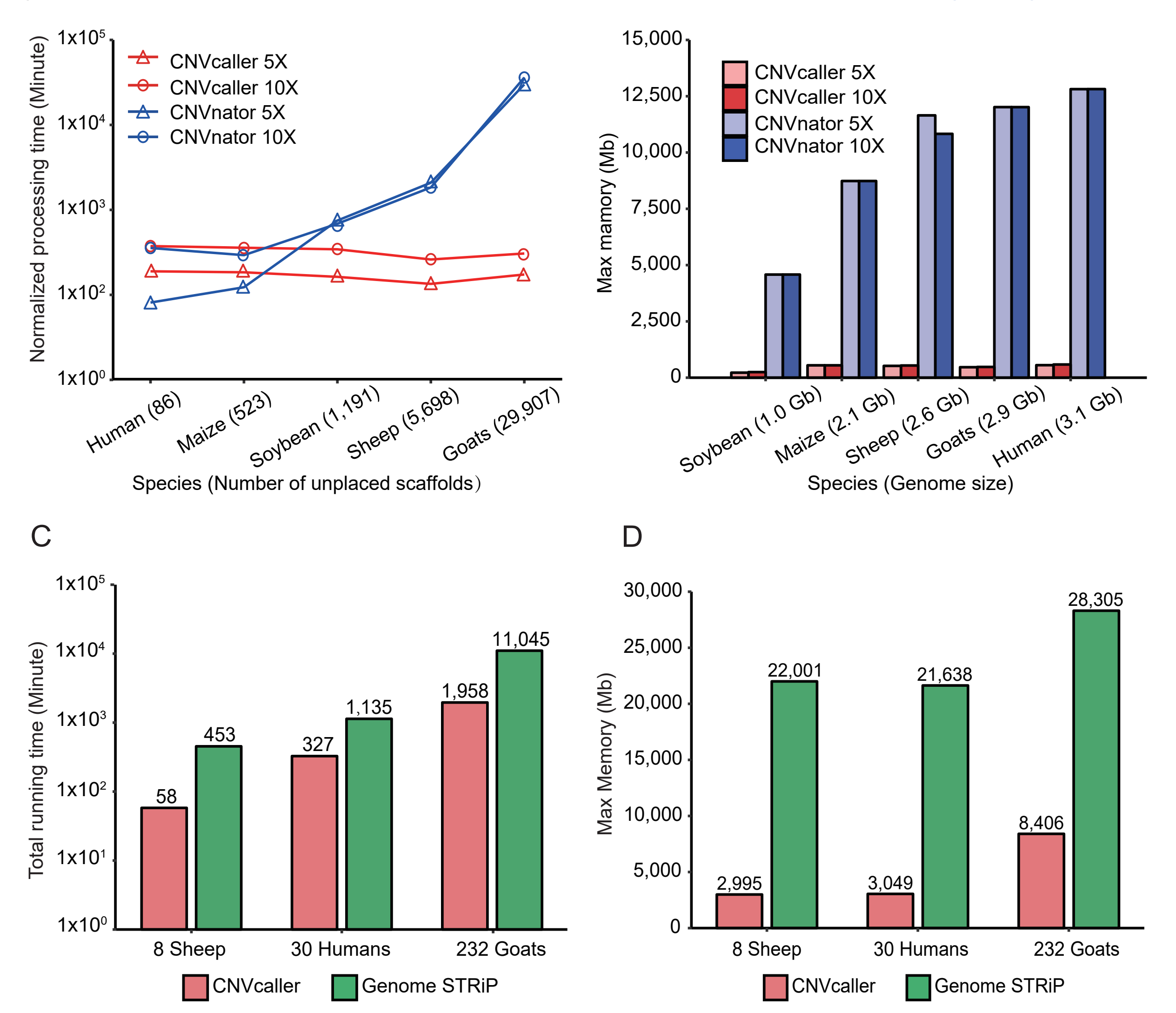

Figure3

Click here to download Figure Figure3.pdf

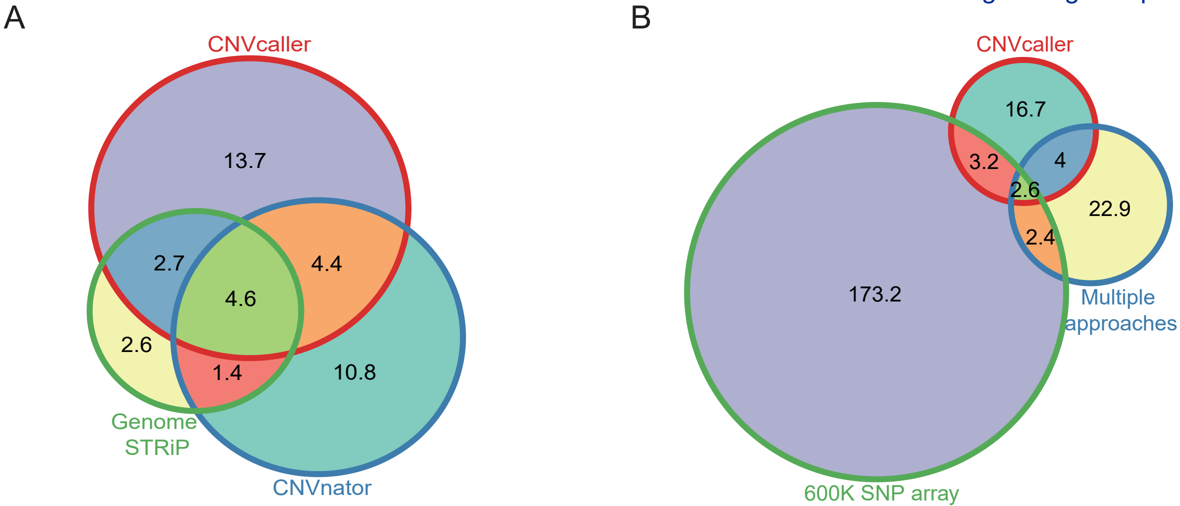

Figure4

Click here to download Figure Figure4.pdf

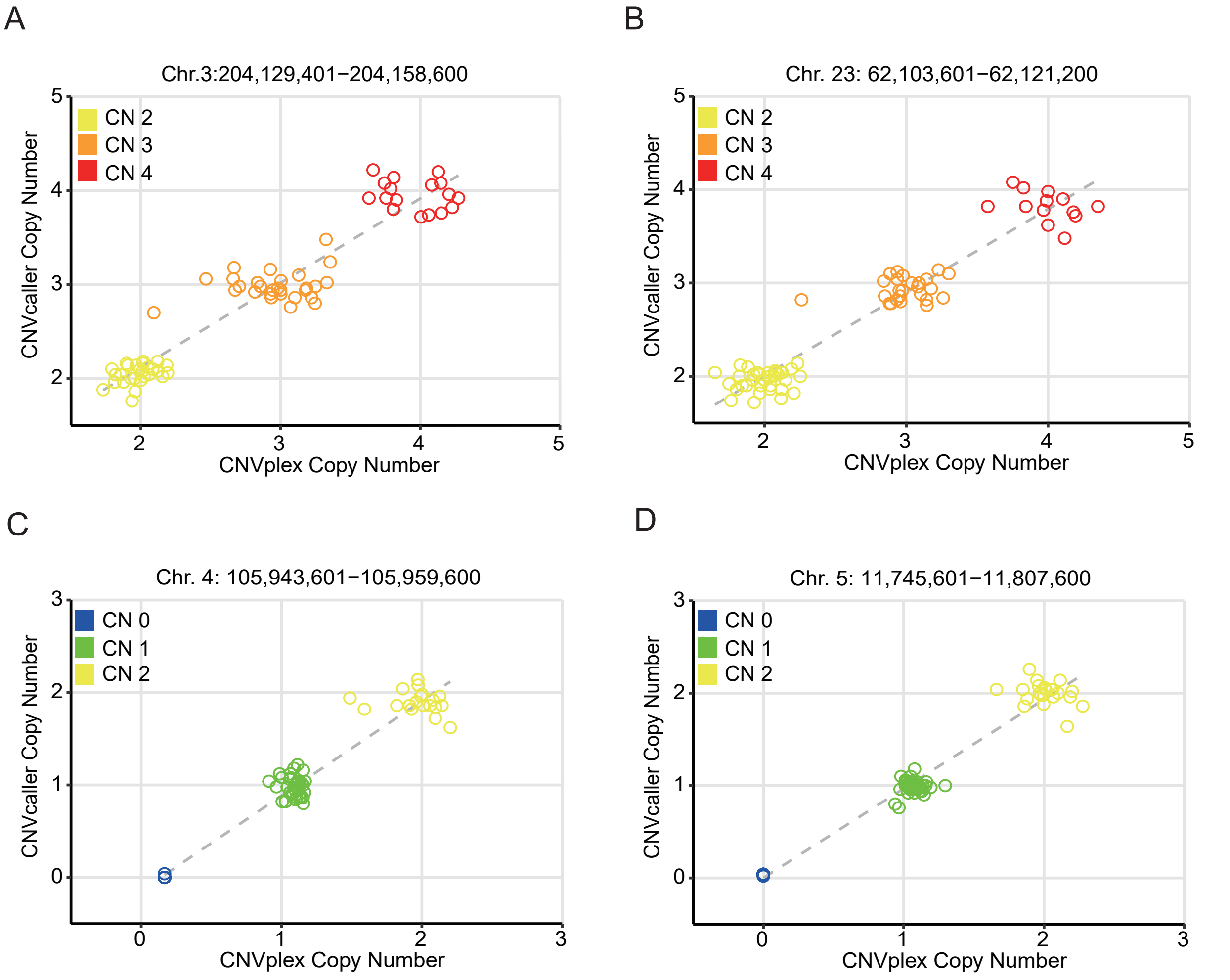

Figure5

Click here to download Figure Figure5.pdf

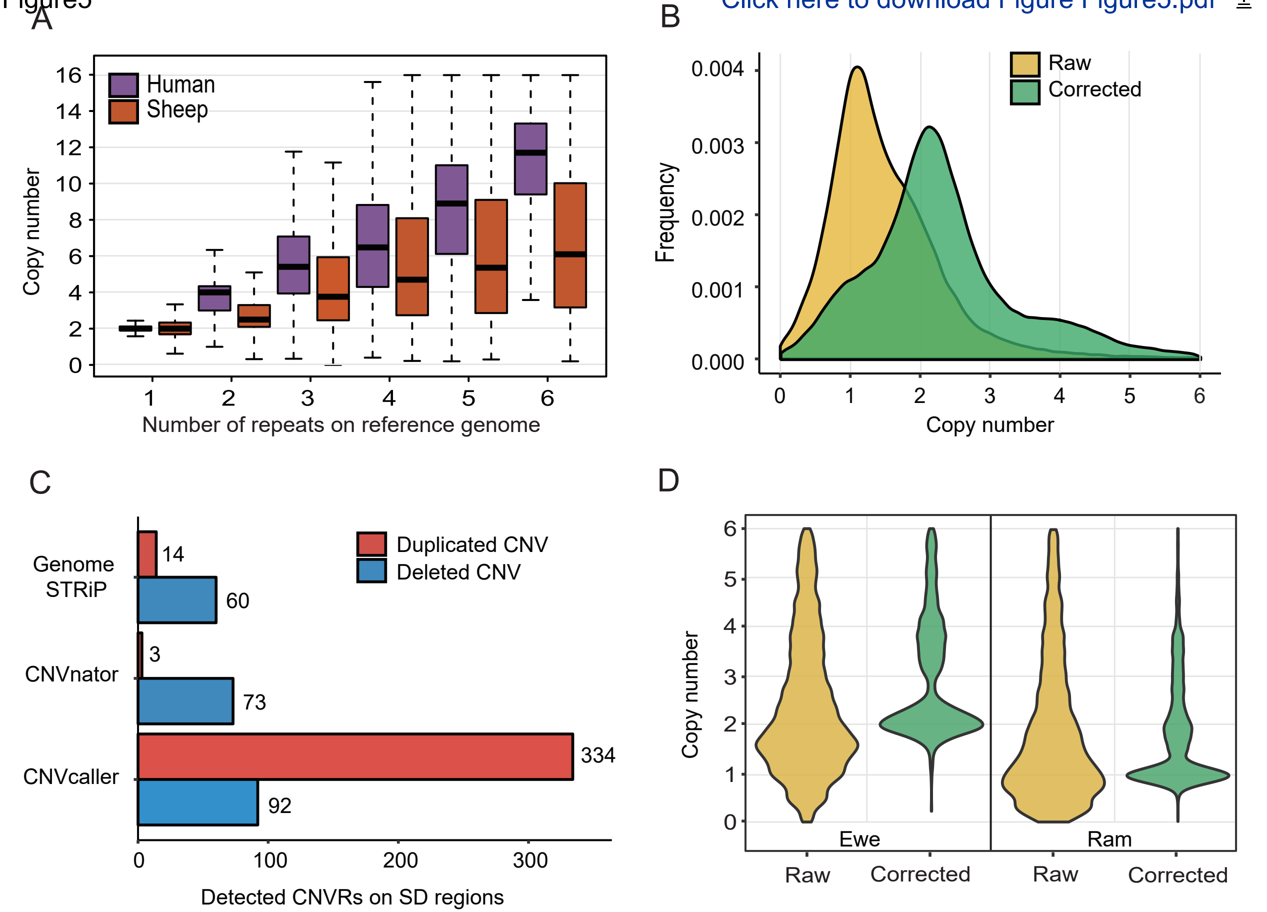

Click here to access/download
Supplementary Material
Supplementary Materials.docx
