# Supplementary material for: CNVcaller: highly efficient and widely applicable software for detecting copy number variations in large populations
Source: Gigascience. 2017 Dec 4;6(12):1–12. doi: 10.1093/gigascience/gix115 (PMC5751039; doi:10.1093/gigascience/gix115)
Supplement: GIGA-D-17-00119_Revision_1.pdf [file gix115_giga-d-17-00119_revision_1.pdf]

GigaScience

CNVcaller: Highly Efficient and Widely Applicable Software for Detecting Copy Number Variations in Large Populations

--Manuscript Draft--

**Manuscript Number:**	GIGA-D-17-00119R1
**Full Title:**	CNVcaller: Highly Efficient and Widely Applicable Software for Detecting Copy Number Variations in Large Populations
**Article Type:**	Technical Note
**Funding Information:**	National Natural Science Foundation of China (31572381) — Prof. Yu Jiang<br>National Thousand Youth Talents Plan — Prof. Yu Jiang
**Abstract:**	Background:<br>The increasing sequencing data of a wide variety of species can be used for copy number variation (CNV) detection at the population level in theory. However, the growing sample size and the divergent complexity of non-human genomes challenges the efficiency and robustness of the current human-oriented CNV detection methods.<br><br>Result:<br>Here, we present CNVcaller, a read depth based method for CNV discovery in population sequencing data. The speed was 1-2 magnitudes higher than CNVnator and Genome STRiP in complex genomes with many unmapped scaffolds. The detection for 232 goats takes only 1.4 days on a single computing node. The Mendelian consistence test of sheep trios indicated that CNVcaller mitigated the influence of high-proportioned gaps and mis-assembled duplications in the non-human reference genome assembly. Furthermore, the validation of both sheep and human samples showed CNVcaller achieved the best accuracy and sensitivity in duplications compared to other methods.<br><br>Conclusion:<br>The fast and general detection algorithms of CNVcaller overcome prior computational barriers for detecting CNVs from large scale sequencing data with complicated genome structures. These advantages promote the population genetic analysis of functional CNVs in more species.
**Corresponding Author:**	Yu Jiang, Ph.D<br>Northwest Agriculture and Forestry University<br>Yangling, Shaanxi CHINA
**Corresponding Author Secondary Information:**	
**Corresponding Author's Institution:**	Northwest Agriculture and Forestry University
**Corresponding Author's Secondary Institution:**	
**First Author:**	Xihong Wang
**First Author Secondary Information:**	
**Order of Authors:**	Xihong Wang<br>Zhuqing Zheng<br>Yudong Cai<br>Ting Chen<br>Chao Li<br>Weiwei Fu

Powered by Editorial Manager® and ProduXion Manager® from Aries Systems Corporation

	Yu Jiang
**Order of Authors Secondary Information:**	
**Response to Reviewers:**	GIGA-D-17-00119<br>CNVcaller: Highly Efficient and Widely Applicable Software for Detecting Copy Number Variations in Large Populations<br>Xihong Wang; Zhuqing Zheng; Yudong Cai; Ting Chen; Chao Li; Weiwei Fu; Yu Jiang<br>GigaScience<br><br>Dear Dr. Edmunds<br><br>Thank you very much for handling our manuscript "CNVcaller: Highly Efficient and Widely Applicable Software for Detecting Copy Number Variations in Large Populations " (GIGA-D-17-00119). We appreciate all the comments from the reviewers, which helped us improve our manuscript. We have now revised the manuscript according to the reviewers' comments and your instructions.<br><br>We addressed the comments and questions of the reviewers as explained below, the reviewers' text has been included and our responses are in colored italics. Revised text is indicated by quotation marks. Because several new figures have been added, we attach a list of the current figures and tables corresponding to those from last version so that the changes can easily be tracked.<br><br>Upon the suggestions of the reviewers, we modified the manuscript as follows:<br><br>1.The newly released version of CNVcaller updated the genotyping method. The python package, scikit-learn v0.19.0, was used to decompose the reported copy numbers into several Gaussian distributions. Therefore, the accuracy of the CNVcaller in the new version was increased.<br><br>2.Since the reviewers required to evaluate the effects of the length and allele frequency of the discovered CNVRs. Two result sections have been added to analyze the number and FDR of the CNVRs detected by the three methods against the length and allele frequency. One section including Figure 4 was based on the sheep data, the other section including Figure 6 was based on the human data.<br><br>3.To answer the reviewer's question about the difference of the FDR in deletions and duplications, their FDR was evaluated respectively in the result sections.<br><br>4.The high-proportion mis-assembled segmental duplications in non-human assemblies caused misunderstanding of the reviewer. The section has been extensively redrafted, as analyzing both real and simulated data.<br><br>5.The previous discussion sections has been merged to the result sections to reduce the length of the manuscript. The first part of the previous results has been moved to the method sections as suggested by the reviewer.<br><br>6.The language has been professionally edited by an English editing service agency, American Journal Experts (AJE). (Because the first version is inadequate, we are waiting for the second version.)<br><br>Thank you again for all of your assistance.<br>Sincerely yours,<br>Yu Jiang, and other coauthors<br><br><br>************************<br>REVIEWS<br>************************<br>Reviewer #1:<br><br>The authors developed a new CNV caller pipeline which they called CNVcaller geared towards improved speed compared to existing CNV callers and improved accuracy for high complexity genomes. I commend the authors on their efforts to introduce

improved algorithms and pipelines for an inherently difficult procedure, namely CNV calling. My comments are mostly suggestions for improvement as followss. Note, comments of the form (4:5 for example represent page 4, line 5).
Thanks for your positive comments and encouragement. We have substantially revised the manuscript upon your suggestions.

There are several grammatical errors which make the paper somewhat confusing. I would strongly recommend further extensive English editing.
We apologize for these mistakes. The manuscript has been professionally edited by an English editing service agency, American Journal Experts (AJE) .

My main criticism of the analysis is one that I have seen repeatedly of most other CNV calling publications, and that is there is no sensitivity analysis.
We are sorry for the ambiguous of the sensitivity tests, which were stuffed in previous Table1. In the revised manuscript, new figures and tests have been added. On general, CNVcaller demonstrated 57%-67% sensitivity for duplications, and 66%-68% for deletions in human data. The sensitivity in sheep data was ~73%.
The detailed descriptions were as follows:
"The sensitivity of human data was estimated as the proportion of the high-confident CNVR database that overlapped by the predicted CNVRs. Two previously published high-confident databases, including the particular samples, were the aCGH-based CNVR database and the 1000GP CNVR map. For the highly variable FDR, the sensitivity estimation removed the calls that were <=2,500 bp and had an alternative allele frequency <5%. For the aCGH database, CNVcaller demonstrated the highest sensitivity (57%) for duplication, 14% and 26% higher than Genome STRiP and CNVnator. Whereas Genome STRiP achieved the highest sensitivity (74%) in deletions, 8% and 2% higher than CNVcaller and CNVnator (Figure 6C). For the 1000 GP CNV maps, even though both Genome STRiP and CNVnator were the core methods of creating the library, the sensitivity of CNVcaller were 68% and 67% for deletions and duplications, only 4%-10% lower than Genome STRiP and CNVnator."
"Because the lack of validated sheep CNVR database, the sensitivity was validated indirectly. Based on our integrated analysis (see method), there are 138 sheep X chromosome origin scaffolds, which were not anchored onto chromosomes of OAR v3.1. Therefore, all of these scaffolds should be detected as CNV because the rams had half copy numbers of ewes. As a result, CNVcaller detected 101 out of these 138 X-origin scaffolds, with a sensitivity of 73%. Furthermore, the corrected copy numbers of these scaffolds were centralized at integer (Figure 3D), whereas the peaks of the raw copy numbers were ambiguous because of splitting the raw RDs among the putative SDs (Supplementary Figure 4). In contrast, CNVnator and Genome STRiP could not report these unmapped CNVRs."

The authors here also suggest various parameters throughout their paper for performing CNV calling, but there is no analysis of how the results change if these parameters are adjusted, i.e. no analysis of how robust your algorithm is to changes in the parameters.
Thank you for your suggestion. The FDR against the window size and allele frequency have been added in Supplementary Figure 1 and Figure 6B.
The following description was added in methods.
"The window size is an important parameter for the RD methods. CNVcaller uses half of the window size as step size. The optimal window size is 800 bp for 5-10X coverage human and livestock sequencing data (Supplementary Figure 1). The recommended scales roughly inversely coverage, resulting in 400 bp windows for 20X coverage and 200 bp windows for 50X coverage."

As another example, Hong et al 27503473 has demonstrated that the biggest variability in calling CNVs is in terms of the CNV size. I suspect that the same can be said of CNVcaller. Please comment on what sizes of CNVs does CNV caller do well or poorly on.
Figure 4 and Figure 6 have been added to evaluate the effect of the length and frequency in sheep and human data. On general, the performance of CNVcaller was good for deletions and duplications >2.5 Kb, however poorly on < 2.5 kb.
The detailed comparisons in the manuscript are as follows:
"The detected CNVRs of CNVcaller and Genome STRiP were further analyzed against the length and alternative allele frequency (Figure 4B). CNVcaller performed better in

duplication detection, it can detect duplications <2.5 Kb, and the Mendelian inconsistence of longer calls were lower than Genome STRiP (3% versus 9% for 2.5Kb ~ 5Kb calls; 2% versus 5% for > 5Kb calls). On the other hand, Genome STRiP detected 1,958 more < 2.5Kb deletions than CNVcaller. One possible reason was Genome STRiP integrating RP methods which have higher capability in detecting shorter deletions. In terms of the frequency, because the detected samples were three trios, most CNVRs were medium frequency (6%-50%). The rare duplications tended to have a higher FDR than the median and high frequency calls (Figure 4C)."
"First, CNVcaller demonstrated the highest overall accuracy for detecting duplications, and the FDR of CNVcaller are relative consistent across duplication length and frequency categories. Whereas the short or singleton duplications of other two methods have high FDR. Second, 43% duplications detected by CNVnator were >20 kb. This was not due to the merged individual CNV to the CNVR, because the average size of individual calls was 3-4 times larger than the other methods. Third, Genome STRiP also showed the highest capability for detecting deletions, especially the short and rare ones, indicating the advantage of combining RD and RP methods in deletion."

2:32 "the prevalent.." is a gross exaggeration. I think you mean "a prevalent".
Corrected as suggested.

2:35 I don't think you mean geometric. I did not comment on other grammatical/English errors as there were too many to list individually. I would highly recommend getting help with the English in this paper.
We apologize for these mistakes. The manuscript has been professionally edited by an English editing service agency, American Journal Experts (AJE).

3:53 "RD" is not defined.
We apologize for the missing. This description has been added to the introduction as follows.
"Read-depth (RD) means the depth of the coverage or the genomic region that can be calculated by the number of reads aligned [16]."

6:120 Give a brief description of how CNVnator handles GC bias. Also why 40% for the GC bias? Shouldn't this parameter be dependent on the organism of interest?
We apologize for not clearly describing the procedure. In general, the mean RD of windows with 40% percent GC is only used as the temporary standard in the GC correction step. It will be lost in the following normalization step: the GC corrected RDs of each window are divided by the global median RDs. Because the denominator is calculated from the RDs already corrected by the 40% GC windows, this parameter will be lost and is not necessarily dependent on the organism of interest.
The CG correction of CNVnator was the combination of the correction and normalization steps of CNVcaller. The equation is as follow:

Where i is bin index, is raw RD signal for a bin, is corrected RD signal for the bin, is average RD signal over all bins, and is the average RD signal over all bins with the same GC content as in the bin.

The commentary on certain genomes not being as complete as others is important. I suspect though that if a large percentage of the samples show a CNV in a genome that is newer or not as complete, then this observation may be more likely indicative of a problem with the reference. Can you comment?
If the detected CNVR has variation in population, which means the read depths can be separated into two or more normal distributions, this call is probably true even with high frequency. On the contrary, if all of the individuals show the same abnormal read depth, it suggests the reference individual is indeed different from the sampling population or have assembly problems.

7:145 I am not convinced Pearson's correlation is appropriate. Your data is likely to have outliers and non-normal data. A non-parametric test of correlation like Spearman's correlation (Kendall-Tau is likely too computational intensive), or performing correlation after 5 or 10% trimming may be more appropriate.
We tried to replace the Pearson's correlation with Spearman's correlation in the 30 BAM files from 1000 Genome Project data. However, after replacement the FDR doubled while the length of each calls reduced to half. A possible reason was the

Spearman's correlation was calculated by sorting of the read depth. So, the diverged copy numbers of deletion or duplication individuals contribution no more than the subtle random mistakes of the normal individuals. In the low frequency CNVRs, the Spearman's correlation index was mainly contributed by the random mistakes of the normal copy individuals.
The trimming is also not recommended for similar reason. In the low frequency CNVRs, the individuals with abnormal copy number will be trimmed as outliers.

cn.MOPS (Klambauer et al, PMID: 22302147) uses a mixture of Poissons as opposed to Gaussian Mixture Models for CNV detection. I suspect the mixture of Poissions will be superior to Gaussian Mixture Models when the read depths are low, and Gausssian mixtures may be more appropriate when read depths are high. How difficult is it to replace the Gaussian mixtures with Poisson mixtures and compare the performance? I feel that this analysis would be informative and potentially improve your algorithm.
Thank you for your suggestion. However, it is not easy to replace the distribution because the RDs after GC correction and normalization are not integer so they can not be directly treated as Poisson distributions. Basically, CNV caller recommended a proper window size to make the standard variation less than 30% of the mean RD, which will not fit the Poisson distribution for RDs. Besides, we used the RDs of 232 goats with 10X coverage to test the fitness of Gaussian distribution using omnibus test (packages). As a result, 88% windows accepted the null hypothesis at P=0.01 level. So, we believe the Gaussian Mixture Models was acceptable for the 10 X data.

The term "CNVR" is critical for understanding the algorithm, and requires more explanation of the term.
We apologies for missing this important concept. The explanation has been added to the introduction as follows.
“To compare the copy number of a particular region across the samples, the shared CNVs among individuals are needed, so the unified CNV regions (CNVRs) were merged from the individual CNVs.”
It would be helpful to include some further discussion on where you see that CNVcaller works better or worse than existing CNV calling software.
Figure 2 showed the speed of CNVcaller was one to two orders of magnitudes higher than the other methods. Figure 4 and Figure 6 have been added to evaluate the effect of the length and frequency in sheep and human data. On general, the performance of CNVcaller was better for all sizes of duplications, however poorly on deletions < 2.5 kb.

9:180. The "arbitrary standards" require a citation.
Two citations were added.
1.Chain FJ, Feulner PG, Panchal M, Eizaguirre C, Samonte IE, Kalbe M, et al. Extensive copy-number variation of young genes across stickleback populations. PLoS genetics. 2014;10 12:e1004830.
2.Abyzov A, Urban AE, Snyder M and Gerstein M. CNVnator: an approach to discover, genotype, and characterize typical and atypical CNVs from family and population genome sequencing. Genome research. 2011;21 6:974-84.

Minor comment: Since speed seems to be a major selling point of the software, more details about running the software on a compute cluster or running algorithms in parallel in the documentation would be helpful.
A new part of “Parallel submission of individual RD processing” has been added to the methods with the principle and command as follows.
“CNVcaller processes the BAM file of each individual separately in the first step, so parallel submissions can be used to save the total running time. All the BAM files should be equally distributed in to N groups, and each group contains M files. The max N = the available processing cores. M = the total number of BAM files/ N. For example, the 232 goat BAM files were processed on a node with 32 processing cores and 128 GB of RAM. We distributed the 232 files into 20 groups, and each group contained 12 BAM files. The shell command for one group likes following:

```
#!/bin/sh
for i in {1..M}
 do bash Individual.Process.sh -b $i.bam -h $i -d dup -s sex_chromosome
done
```

After corrections and normalization, the comparable RDs of each sample are concentrated to an ~100 MB intermediate file and output. This design avoids the

	repeated calculation of the same individual in different populations."<br><br>************************<br>Reviewer #2:<br><br>The proposed method "CNVcaller" enables the efficient discovery and genotyping of CNVs in large populations. One of the main benefits of the method is that it can handle draft genome assemblies with thousands of scaffolds. The computational benchmarks proof that the method is fast and memory efficient but the evaluation of the accuracy of the method is less convincing. Some details of the method remain vague and hinder an objective evaluation. Detailed comments of how to improve the manuscript are below:<br>Thank you for your affirmation. We are sorry for the ambiguous of the accuracy test, and have substantially revised the manuscript upon your suggestions. In the revised manuscript, the performance evaluation in previous Table 1 was described more detail and classify by the species in Figure 4 and Figure 6.<br><br>Comment 1 - The primary application of CNVcaller is the detection of CNVs in large populations. Population variant call sets are dominated by rare variants of rather small size. For instance, less than 20% of the 1000 Genomes structural variants have a population allele frequency >5% and almost 50% of the SVs are <2kbp in size despite the rather low coverage (~7x). CNVcaller is currently restricted to large CNVs (>2kbp) and common variants (>5% allele frequency), which is a major limitation for population genomic studies.<br>In the revised version, all detected calls were included in the IRS test. In fact, all three methods can report some short and rare CNVRs. However, the short and rare duplications made by Genome STRiP and CNVnator had extremely high FDR. So, we excluded these results from the previous version of manuscript as 1000 GP.<br>The newly added Figure 6 showed the shortest duplication reported by CNVnator and Genome STRiP was 2.8 kb and 2.5 kb, and the IRS FDR of 2.5-5kb calls are 29% and 88%, respectively. The FDR of >2.5kb singletons was 35% and 69% for CNVnator and Genome STRiP, respectively. These uncertain calls were also removed by the phase 3 extended SV release of 1000GP. After extra quality controls, the number of duplications in the released database are only 1/7 of deletions, and the median size was 36 kb, 17 times longer than deletions. Therefore, improving the accuracy of duplications on this foundation is meaningful for enrich the CNV database. The main improvement of CNVcaller is the accuracy of duplications. The FDR of 2.5 kb – 5 kb was reduced to 19%, and the >2.5kb singletons was reduced to 9%. However, the FDR were still higher than the longer and higher frequency calls.<br>Besides, the current main usage of CNVcaller is to detect the CNVRs related to economy traits in livestock and crops. In these populations, the target CNVs usually have a medium or high frequency after long time artificial selection. We believe the high-confident medium to high frequency reported by CNVcaller can contribute to the functional and breeding study of non-human studies.<br><br>The sensitivity increase of CNVcaller for the subset of common and large CNVs seems to be driven by an increased number of detected CNVs in SD regions (Figure 5C). SNP arrays have a low SNP density in SD regions and in the present Manuscript array SNP probes in SD regions have been removed entirely. The reported IRS FDR is therefore heavily biased against CNVs in SD regions and it thus seems mandatory to me to proof that this sensitivity increase for SD-associated CNVs is not leading to an inflated FDR.<br>Thanks for your suggestions. Figure 5C (New Figure 3C) was updated to show both the number and the Mendelian inconsistence of the detected CNVs in SDs. The inconsistence rate of the calls in SD regions made by CNVcaller was about 3%. The copy numbers of unique and SDs were also indirectly validated by the X-origin scaffolds of a 133-sheep population. In both validation dataset, one main reason for acceptable FDR in SDs was most SDs in sheep reference genome assembly is actually mis-assembled unique region.<br>The detailed description are as follows:<br>"In the real dataset, CNVcaller detected more duplications in the SDs of the sheep genome with only 3% Mendelian inconsistence (Figure 3C, Supplementary Figure 3). Because the lack of validated sheep CNVR database, the sensitivity was validated indirectly. Based on our integrated analysis (see method), there are 138 sheep X chromosome origin scaffolds, which were not anchored onto chromosomes of OAR

v3.1. Therefore, all of these scaffolds should be detected as CNV because the rams had half copy numbers of ewes. As a result, CNVcaller detected 101 out of these 138 X-origin scaffolds, with a sensitivity of 73%. Furthermore, the corrected copy numbers of these scaffolds were centralized at integer (Figure 3D), whereas the peaks of the raw copy numbers were ambiguous because of splitting the raw RDs among the putative SDs (Supplementary Figure 4). In contrast, CNVnator and Genome STRiP could not report these unmapped CNVRs.”

The Manuscript lacks a Figure that shows the size and allele frequency distribution of the discovered CNVs in comparison to Genome STRiP and CNVnator. An estimate of breakpoint accuracy of CNVcaller would also be valuable.
Thanks for your suggestion. Figure 4 and Figure 6 have been added to evaluate the effect of the length and frequency in sheep and human data. On general, the performance of CNVcaller was better for all sizes of duplications, however poorly on deletions < 2.5 kb.
The breakpoint accuracy is an innate disadvantage of RD methods. Because the detailed situation within a window is not calculated. And the window size cannot be too small for the medium or low coverage sequencing data. We recommend the user to combine the read pair or split read methods to improve the breakpoint accuracy.
The detailed comparations in the manuscript are as follows:
“The detected CNVRs of CNVcaller and Genome STRiP were further analyzed against the length and alternative allele frequency (Figure 4B). CNVcaller performed better in duplication detection, it can detect duplications <2.5 Kb, and the Mendelian inconsistence of longer calls were lower than Genome STRiP (3% versus 9% for 2.5Kb ~ 5Kb calls; 2% versus 5% for > 5Kb calls). On the other hand, Genome STRiP detected 1958 more < 2.5Kb deletions than CNVnator. One possible reason was Genome STRiP integrating RP methods which have higher capability in detecting shorter deletions. In terms of the frequency, because the detected samples were three trios, most CNVRs were medium frequency (6%-50%). The rare duplications tended to have a higher FDR than the median and high frequency calls (Figure 4C).”
“First, CNVcaller demonstrated the highest overall accuracy for detecting duplications, and the FDR of CNVcaller are relative consistent across duplication length and frequency categories. Whereas the short or singleton duplications of other two methods have high FDR. Second, 43% duplications detected by CNVnator were >20 kb. This was not due to the merged individual CNV to the CNVR, because the average size of individual calls was 3-4 times larger than the other methods. Third, Genome STRiP also showed the highest capability for detecting deletions, especially the short and rare ones, indicating the advantage of combining RD and RP methods in deletion. Besides directly combination of the two methods into one piece of software, another option was using high-confidence RD results generated CNVcaller as the prior to improve the accuracy of the read pair/split read pipeline.”

The Manuscript mentions mrsFAST for absolute copy number validation. I could not find any formal comparison of predicted copy-number by mrsFAST and CNVcaller but maybe I missed this?
Supplementary Figure 2 (Previous Supplementary Figure 1) showed the copy number calculated from mrsFAST and CNVcaller was similar. However, mrsFAST needed to realign all the multi-hit reads in BWA alignments, leading to significantly increased computational time. For example, mrsFAST needed 10 hours for a 3G genome with 10X sequencing data, whereas, CNVcaller only needed 4 minutes.
- Please add to Table 1 the number of CNV sites that could be assessed by the IRS method and what proportion of each call set could be evaluated using IRS. I also believe the IRS method reports p-values separately for deletions, duplications and multi-allelic CNVs. Was there any difference among these for CNVcaller?
The detailed information of 1000GP calls including the required information has been added to Supplementary Table 5. Overall, 28%, 30% and 60% CNVRs of CNVcaller, CNVnator and Genome STRiP covered at least one probe of Affymetrix SNP 6.0 array, therefore can be assessed by IRS test. One main reason for the diverged testable proportion was only 4% of Genome STRiP calls were overlap with SDs which have seldom probes, whereas the 34% CNVcaller calls and 28%CNVnator calls were overlap with SDs.
Two extra genome-wide evaluations can provide supplemental proofs. The Mendelian inconsistence of 10 Dutch family was added in Supplementary Figure 5, which can test both unique and SD regions. The inconsistence rate of CNVcaller, CNVnator and

Powered by Editorial Manager® and ProduXion Manager® from Aries Systems Corporation

Genome STRiP was 1.5%, 4.4%, and 0.4%. This accuracy ranking was consistent with the genotyping discordance compared with the aCGH database, which were 2.6%, 5.5% and 2.2% for CNVcaller, CNVnator and Genome STRiP respectively.

- Some details of the method are vaguely specified and some Figures lack clarity and units.
Page 6, line 129: "... if the median RD of the homogametic sex chromosomes is about half of the median RD of autosome..."
Modified as follows:
“Most mammalian and avian genomes show XX/XY-type or ZZ/ZW-type sex-determining system. Their homogametic sex chromosomes (X or Z) constitute 5%-10% of the total genome, and show half RD of the autosomes in XY or ZW individuals. Therefore, insensitive corrections are needed. The name of the homogametic sex chromosome is required as a parameter. If the median RD of this chromosome is <0.6X of the median RD of the autosome, this individual is determined as the XY or ZW type, and the RDs of this chromosome are doubled before normalization. Otherwise, this individual is determined as XX or ZZ type, and no sex correction will be done.”

Page 8, line 154: "... and the distance between them is less than a certain percent of their own length."
Modified as follows: “The distance between the two initial calls is less than 20% of their combined length.”

Page 5, line 91: "The reference genome is segmented into overlapping sliding windows." What window size and overlap was used for high-coverage genomes?
The following description was added in methods.
“The window size is an important parameter for the RD methods. CNVcaller uses half of the window size as step size. The optimal window size is 800 bp (with a 400 bp overlap) for 5-10X coverage human and livestock sequencing data (Supplementary Figure 1). The recommended scales roughly inversely coverage, resulting in 400 bp windows for 20X coverage and 200 bp windows for 50X coverage.”

Page 5, line 95: "The raw RD signal is calculated for each window as the number of placed reads with centers within window boundaries." Does this imply that for paired-end data both reads are counted?
Yes, considering the uncontrollable effect of gap ratios from different genome assemblies, all of the end reads located in the window are independently added to the RD of this window, regardless of the read is from single end mapping or paired mapping.

Page 8, line 154: "Then the two adjacent initial calls are further merged if their copy numbers are highly correlated". What threshold was used?
Modified as follows: “CNVRs can be separated by gaps or poorly assembled regions, therefore, the adjacent initial calls are merged if their RDs are highly correlated. The default parameters are: the distance between the two initial calls is less than 20% of their combined length, and the Person’s correlation index of the two CNVRs is significant at P = 0.01 level.”

Figure 3A: CNVcaller 13.7. What is the unit? Are these 13,700 CNVs?
The unit of this figure was Mbp, because the intersection of the three methods was hard to define in number with different boundaries, so they are evaluated in length. CNVcaller covered 40% of the CNVRs detected by CNVnator, 45% of Genome STRiP and 65% of their intersection, in length.

Minor:
- I could not find a reference to the 232 goat sequencing data? Is this data publicly available?
Among the 232 goat whole genome sequencing data, 103 were acquired from NCBI (reference paper see below), and the accession numbers are provided in Supplementary Table 1. The remaining 129 samples without accession number were generated by ourselves.
Badr Benjelloun FJA, Streeter I, Boyer F, Coissac E, Stucki S, et al. (2015) Characterizing neutral genomic diversity and selection signatures in indigenous

	populations of Moroccan goats (Capra hircus) using WGS data. Frontiers in genetics 6.<br>Dong Y, Zhang X, Xie M, Arefnezhad B, Wang Z, et al. (2015) Reference genome of wild goat (capra aegagrus) and sequencing of goat breeds provide insight into genic basis of goat domestication. BMC genomics 16: 431.<br>Dong Y, Xie M, Jiang Y, Xiao N, Du X, et al. (2013) Sequencing and automated whole-genome optical mapping of the genome of a domestic goat (Capra hircus). Nature biotechnology 31: 135-141.<br>Bickhart DM, Rosen BD, Koren S, Sayre BL, Hastie AR, et al. (2017) Single-molecule sequencing and chromatin conformation capture enable de novo reference assembly of the domestic goat genome. Nature Genetics 49: 643-650.<br>Wang XL, Liu J, Niu YY, Li Y, Zhou SW, et al. Low incidence of SNVs and indels in trio genomes of Cas9-mediated multiplex edited sheep. BMC Genomics. Under review.<br><br>- The first Results section "Overview of CNVcaller algorithm" seems better suited for the Methods part.<br>Modified as suggested.<br><br>- Is the Mendelian consistency higher for the high-coverage trio: NA12878, her father (NA12891) and her mother (NA12892)?<br>Yes. Upon the high coverage data of all three members of the trio (NA12891, NA12892 and NA12878 are all 50 X), the inconsistent rate was 2.4%. Upon the high coverage parents (50 X NA12891 and NA12892) and low coverage child (5.3 X NA12878), the inconsistent rate was 6.1%. So, the increased sequencing depth can help to reduce the number of false positives.<br><br>- I believe the claim that read-pair/split-read algorithms are less powerful on draft assemblies of non-model organisms compared to read-depth methods is potentially true but the Manuscript lacks a proof for this or a citation that supports this claim.<br>Thank you for your agreement. This problem was found in our previous reference genome assembly projects for both sheep and goats. However, we did not report this result in the section of CNV/SD detection. So, we removed this comment from this manuscript. However, we found the following citations may help to support this claim:<br>All of these algorithms including read-pair/split-read (RP/SR) and read-depth rely on mapping sequencing reads back to reference genome. However, for many non-model organisms, the reference genome likely contains many errors, which mainly arose from repeat collapse and expansion; and rearrangement and inversion [1] . These mis-assembly sequences and the repetitive regions of the genome can result in many pair-end reads have multiple good mappings, thus it is difficult for RP/SR to uniquely identify the true CNVs boundaries[2]. However, based on read depth by considering all possible map locations for a read can address this problem[3].<br>1. Phillippy AM, Schatz MC, Pop M (2008) Genome assembly forensics: finding the elusive mis-assembly. Genome biology 9: R55.<br>2. He D, Hormozdiari F, Furlotte N, Eskin E (2011) Efficient algorithms for tandem copy number variation reconstruction in repeat-rich regions. Bioinformatics 27: 1513-1520.<br>3. Alkan C, Kidd JM, Marques-Bonet T, Aksay G, Antonacci F, et al. (2009) Personalized copy number and segmental duplication maps using next-generation sequencing. Nature genetics 41: 1061-1067.<br><br>- It is not clear from the Manuscript if CNVcaller reports copy-number likelihoods based on the Gaussian mixture model. Please clarify.<br>Thank you for you suggest. CNVcaller reports the silhouette coefficients of the copy numbers instead of the Gaussian mixture model likelihoods as quality control. Because we found silhouette coefficients has greater correlation with IRS test result than likelihoods.<br><br>- Figure 5A: Why is the absolute copy-number correction different for Human and Sheep?<br>We are sorry for not clearly interpreting the high-proportioned mis-assembled segmental duplications in non-human assemblies. This part was modified as follows: “Previous studies showed high proportion of SDs in animal genomes are mis-assembled single copy regions. So, we validated the copy numbers on human (hg19) and sheep (OAR v3.1) reference genome assembly by the sequencing copy number of a human (NA12878) and a Tan sheep sample (Figure 6A). If the SDs were correctly assembled, the sequencing diploid copy number should be two times of the copy

Powered by Editorial Manager® and ProduXion Manager® from Aries Systems Corporation

	number of SDs. For example, the average sequencing copy number of the two-copy SDs was four in NA12878. However, the corresponding sequencing copy number of sheep was only 2.4. These results indicated most two-copy SDs of hg19 were truly duplicated in NA12878 while approximately 80% of the two-copy SDs in OAR v3.1 were unique regions in the Tan sheep sample. So, the SDs in sheep genome were called "putative SDs" before validation."<br><br>- There is quite a few typing and grammatical errors. For instance:<br>*Figure 2B: Max mamory<br>*Supplementary Table 3: Memery<br>*Page 3, line 53: ...the number of reads aligned to of a particular region.<br>*Page 8, line 160: This model presets the average copy number of homozygous deletion, heterozygous deletion, normal, heterozygous deletion (duplication!), homozygous deletion (duplication!) at zero to four respectively.<br>We are sorry for these mistakes. We have proofread the revised manuscript and used professional English language editing to minimize the grammatical errors.<br><br>**********************************<br>Checklist of the updated tables and figures<br><br>Current versionLast version<br>Fig. 3Fig. 5<br>Fig. 4A-CTable 1 and newly added<br>Fig. 4DFig. 3B<br>Fig. 5Fig. 4<br>Fig. 6A-CTable 1 and newly added<br>Fig. 6DFig. 3A<br>Supplementary Fig. 1Newly added<br>Supplementary Fig. 2Supplementary Fig. 1<br>Supplementary Fig. 3Newly added<br>Supplementary Fig. 4Supplementary Fig. 2<br>Supplementary Fig. 5Table 1 and newly added<br>Supplementary Table 4Newly added<br>Supplementary Table 5Newly added
**Additional Information:**	
**Question**	**Response**
Are you submitting this manuscript to a special series or article collection?	No
**Experimental design and statistics**<br><br>Full details of the experimental design and statistical methods used should be given in the Methods section, as detailed in our Minimum Standards Reporting Checklist. Information essential to interpreting the data presented should be made available in the figure legends.<br><br>Have you included all the information requested in your manuscript?	Yes
**Resources**	Yes

Powered by Editorial Manager® and ProduXion Manager® from Aries Systems Corporation

A description of all resources used, including antibodies, cell lines, animals and software tools, with enough information to allow them to be uniquely identified, should be included in the Methods section. Authors are strongly encouraged to cite Research Resource Identifiers (RRIDs) for antibodies, model organisms and tools, where possible.<br><br>Have you included the information requested as detailed in our Minimum Standards Reporting Checklist?	
**Availability of data and materials**<br><br>All datasets and code on which the conclusions of the paper rely must be either included in your submission or deposited in publicly available repositories (where available and ethically appropriate), referencing such data using a unique identifier in the references and in the “Availability of Data and Materials” section of your manuscript.<br><br>Have you have met the above requirement as detailed in our Minimum Standards Reporting Checklist?	Yes

Manuscript Click here to download Manuscript CNVcalleredit10_6.docx

CNVcaller: Highly Efficient and Widely Applicable Software for Detecting Copy Number Variations in Large Populations

Xihong Wang [1†], Zhuqing Zheng[1†], Yudong Cai[1], Ting Chen[1], Chao Li[1], Weiwei Fu[1], Yu Jiang[1]*

1 College of Animal Science and Technology, Northwest A&F University, Yangling, Shaanxi, China

† These authors contributed equally to this work.

*Correspondence should be addressed to Yu Jiang (yu.jiang@nwafu.edu.cn).

Abstract

Background: The increasing sequencing data of a wide variety of species can be used for copy number variation (CNV) detection at the population level in theory. However, the growing sample size and the divergent complexity of non-human genomes challenges the efficiency and robustness of the current human-oriented CNV detection methods.

Result: Here, we present CNVcaller, a read depth based method for CNV discovery in population sequencing data. The speed was 1-2 magnitudes higher than CNVnator and Genome STRiP in complex genomes with many unmapped scaffolds. The detection for 232 goats takes only 1.4 days on a single computing node. The Mendelian consistence test of sheep trios indicated that CNVcaller mitigated the influence of high-proportioned gaps and mis-assembled duplications in the non-human reference genome assembly. Furthermore, the validation of both sheep and human samples showed CNVcaller achieved the best accuracy and sensitivity in duplications compared to other methods.

Conclusion: The fast and general detection algorithms of CNVcaller overcome prior computational barriers for detecting CNVs from large scale sequencing data with complicated genome structures. These advantages promote the population genetic analysis of functional CNVs in more species.

Keywords

copy number variation (CNV), next-generation sequencing (NGS), read depth (RD), population genetics, absolute copy number.

Introduction

Copy number variations (CNVs) are defined as duplications or deletions of genomic segments that range in size from 50 base pairs (bps) to megabase pairs (Mb) and vary among individuals and species [1]. As a prevalent and important source of genetic diversity, the number of CNVs detected in the human genome were over 50,000 and accounted for 10% of the whole genome[2]. CNVs can regulate the gene expression by both dosage and position effects, and have larger expression-altering effect sizes than do SNVs and indels [3]. In the human genome, CNVs are important genetic components of numerous diseases [4,5] and are a main force of evolution [6]. The CNVs in both animals and plants are also associated with economically important phenotypes and functions [7-11].

With the dramatic growth of the sequencing capacity and the accompanying drop in cost, an enormous amount of large-scale sequencing has been completed and available in public database. Currently, several strategies are used to detect CNVs using whole genome sequencing data: 1. Pead-pair (PR) and split-read (SR) methods analyze the abnormally mapping pair-end reads and the gapped alignment [12-15]. 2. Read-depth (RD) methods analyze the depth of the coverage in a genomic region that can be calculated by the number of reads aligned [16]. The RD is correlated with the copy number of the region, e.g., a duplicated region should have a higher RD than expected [17-19]. 3. The short reads local assembly methods extract the reads that mapped near the predicted breakpoint and assembles them into longer sequence contigs [20,21]. Employing multiple methods in one dataset can increase the total sensitivity [22], however, the efficiency and convenience would consequently become the subsequent concern, as the computational efficiency is one of the biggest challenges for the current CNV detectors.

The increasing sequencing sample size also enabled the discovery of functional CNVs using genome-wide association study (GWAS) in population level [11]. To compare the copy number of a particular region across the samples, the shared CNVs among individuals are needed, so the unified CNV regions (CNVRs) were merged from the individual CNVs [23]. However, detecting CNV individually then merged by arbitrary standard are low efficient and not feasible for the large cohort. Therefore, the population genetic information is applied to improve the detecting accuracy. A typical strategy is assuming the RD of a sliding window of all the samples to follow a specific distribution and imply populational merging criteria to directly report the CNVRs. A typical strategy is to simultaneously scan the genomes of multiple samples, then decomposes the variations in the RD across samples into true variations and noises by priori distributions [24,25].

In addition, the complicated genome structure of many non-human species demands more robust signal detection and noise reduction algorithms. First, the gaps and unplaced scaffolds are riddled with the reference genome assemblies of most non-model organisms [26,27], increasing the detecting errors. Second, because the heterogeneity alleles are easily misassembled into tandem duplication, a high proportion of the mis-assembled segmental duplications (SDs) exist in the non-human reference genomes [28]. For example, in the cattle genome assembly, Btau 4.1, 97% of the highly similar tandem duplications are actually single copy regions [29]. These error duplications can lead to false CNVRs discovery and genotyping.

In this study, we introduce a super-fast and generally applicable method, CNVcaller, for discovering CNV from sequencing data in large populations. This software is based on the RD algorithm and implies robust signal detection and noise deduction methods to increase the computational efficiency in complex genomes. We applied it to the population sequencing data

from humans, livestock and crops to demonstrate its utility and benchmarked it against the RD-based individual CNV detector CNVnator, which has been used in yak, chicken and fish cohorts [30-32], and the best practice population-level CNV detector Genome STRiP.

Materials and Methods

Input data

The main input of CNVcaller is the alignment files in BAM format. The following data/samples were included in the validation.30 human BAM files from the 1000 Genome Project (1000GP) Phase 3 [33], including 27 normal (~12X) and three deeply sequenced samples (~50X). 30 BAM files (~20X) from 10 families from the Genomes of Netherlands (GoNL) project [34]. 70 FASTQ files from domestic sheep samples (~10X) from the NCBI BioProject: PRJNA160933. 232 goat whole genome sequencing data, among these, 103 were acquired from NCBI [35-38], and the accession numbers are provided in **Supplementary Table 1**. The remaining 129 samples without accession number were generated by ourselves (unpublished data). Two maize [39] and two soybean [11] FASTQ files (each species contain one ~5X and one ~10X sample). Three Tan sheep trios, including 8 individuals (~19X) and 129 goat (~12X) data were from unpublished data. An additional table shows the downloaded files in detail **(Supplementary Table 1)**.

The FASTQ files were aligned to their respective reference assemblies using BWA 0.7.13 to generate BAM files [40]. The versions of the reference genomes include human GRCh37, maize B73 RefGen_v3, soybean Glycine_max_v2.0, sheep OAR_v3.1 and goat ARS1. The GATK v3.5 [41] pre-processing workflow was used to produce the analysis-ready BAM files. After alignment,

the PCR duplications were marked by Picard 2.1 (https://broadinstitute.github.io/picard), and the realignment was performed by GATK. The reads with a 0x504 flag (indicating unmapped, secondary mapped or PCR duplication) were removed.

Individual RD processing

RD Estimation. The pipeline of CNVcaller was showed in **Figure 1**. The reference genome is segmented into overlapping sliding windows. The windows are indexed to form a reference database, which is used in all samples. The sliding windows with >50% gaps are excluded from the database and further computation. The BAM file of each individual was parsed out using SAMtools v1.3 [42]. The raw RD signal is calculated for each window as the number of placed reads with centres within the window boundaries. This step consumes less than 500 MB maximum memory for one BAM file, so parallel submitting is recommended. The window size is an important parameter for the RD methods. CNVcaller uses half of the window size as step size. The optimal window size is 800 bp (with a 400 bp overlap) for 5-10X coverage human and livestock sequencing data **(Supplementary Figure 1)**. The recommended scales roughly inversely coverage, resulting in 400 bp windows for 20X coverage and 200 bp windows for 50X coverage.

Absolute copy number correction. To perform the absolute copy number correction, windows with >97% sequence similarity are linked together to form a duplicated window record file. This file is generated by splitting the reference genome into non-overlapping windows and aligning them onto the reference genome using the precise aligner BLAT v. 36x1 [43]. The windows with more than 20 hits are excluded to remove the low complexity regions. The record files of humans,

livestock and main crops can be downloaded from the CNVcaller website (http://animal.nwsuaf.edu.cn/).

Based on the duplicated window record file, the raw RDs, located on similar windows, are added together to generate the absolute RD for all the high similarity windows.

$$RD^{i}_{absolute} = \sum_{j=1}^{t} RD^{ij}_{raw}$$

where i is the index of the window to be corrected, and t is the total number of the high similarity windows. RD^{ij}_{raw} is the raw RD of the window similar with the i-th window (including the i-th window itself), which is counted directly from the BWA alignment, and $RD^{i}_{absolute}$ is the corrected RD of the i-th window, which can be used to deduce the absolute copy number.

GC correction. Considering that the population sequencing data may come from different platforms, the RD of each sample was counted and corrected individually. Since the resequencing samples may show various GC content distributions, the GC bias is corrected individually basically as CNVnator [18]:

$$RD^{i}_{corrected} = \frac{\overline{RD_{40}}}{\overline{RD_{gc}}} RD^{i}_{absolute}$$

where i is the window index, $RD^{i}_{absolute}$ is the RD after the absolute copy number correction, $RD^{i}_{corrected}$ is final corrected RD for the window, $\overline{RD_{40}}$ is the mean RD of windows with 40% percent GC as a standard, and $\overline{RD_{gc}}$ is the mean RD over all the windows that have the same GC content with the i-th window.

RD normalization. Because the samples have different sequencing depths, the corrected RD need

to be normalized to a single standard before the population-level CNV detection. Assuming that the majority of the genome is normal copy number, the corrected RDs are divided by the global median RD to normalize to one.

$$RD^{i}_{normalized} = \frac{RD^{i}_{corrected}}{\overline{RD}_{global}}$$

where the $\overline{RD}_{global}$ is the median of the $RD^{i}_{corrected}$ of all the windows.

Most mammalian and avian genomes show XX/XY-type or ZZ/ZW-type sex-determining system. Their homogametic sex chromosomes (X or Z) constitute 5%-10% of the total genome, and show half RD of the autosomes in XY or ZW individuals. Therefore, insensitive corrections are needed. The name of the homogametic sex chromosome is required as a parameter. If the median RD of this chromosome is <0.6X of the median RD of the autosome, this individual is determined as the XY or ZW type, and the RDs of this chromosome are doubled before normalization. Otherwise, this individual is determined as XX or ZZ type, and no sex correction will be done.

Parallel submission of the individual RD processing. CNVcaller processes the BAM file of each individual separately in the first step, so parallel submissions can be used to save the total running time. All the BAM files should be equally distributed in to N groups, and each group contains M files. The max N = the available processing cores. M = the total number of BAM files/ N. For example, the 232 goat BAM files were processed on a node with 32 processing cores and 124 GB of RAM. We distributed the 232 files into 20 groups, and each group contained 12 BAM files. The shell command for one group likes following:

```
#!/bin/sh
for i in 1..M
  do bash Individual.Process.sh -b $i.bam -h $i -d dup -s sex_chromosome
done
```

After corrections and normalization, the comparable RDs of each sample are concentrated to an ~100 MB intermediate file and output. This design avoids the repeated calculation of the same individual in different populations.

CNVR detection by multiple criteria

Individual candidate CNV window definition. The individual candidate CNV windows are defined using two criteria: (1) The normalized RD is significantly higher or lower than the normalized mean RD (deletions < 1 – 2 * STDEV; duplications > 1 + 2 * STDEV). (2) Considering that the normalized RD of the heterozygous deletions and duplications should be approximately 0.5 and 1.5, respectively, an empirical standard for the normalized RD (deletions < 0.65; duplications > 1.35) also needs to be achieved. For some strictly self-bred species, such as soybean and wheat, this empirical standard should be raised to 0.25 or 1.75 for the normalized RD of the homozygous deletions or duplications, respectively.

Population-level candidate CNV window definition. All the individual RD files are piled up by the universal window index to a two-dimensional population RD file. Each line of this file is the multi-sample RDs of a particular window, from which the candidate CNV windows are selected.

The user can retain all the windows with at least one individual shows heterozygotic deletion or duplication. However, we recommend removing the low frequency windows in large population with low sequencing coverage because of increased random mistakes. By default, the windows with allele frequency ≥ 0.05 or at least two homozygous duplicated/deleted individuals are selected for the further validation. Then, Pearson's product–moment correlation coefficients of the multi-sample RDs are calculated between the two adjacent non-overlapping windows. If the Person's correlation index is significant at P = 0.01 level by Student's t test. The two windows are merged into one call.

CNV region definition. The initial CNVRs are selected if more than four sequential overlapped windows are defined as the population-level candidate windows. For each individual, the medium RD of all the windows in this CNVR was defined as the RD of this CNVR. For noise tolerance, at most, one unselected window out of four continuous candidate windows is allowed to exist in the CNVR, however, their RD is not calculated in the RD of CNVR. CNVRs can be separated by gaps or poorly assembled regions, therefore, the adjacent initial calls are merged if their RDs are highly correlated. The default parameters are: the distance between the two initial calls is less than 20% of their combined length, and the Person's correlation index of the two CNVRs is significant at P = 0.01 level.

CNVR Genotyping

After merging the candidate CNV windows into a CNVR, the RDs of all samples in each CNVR

are clustered, and the integer copy number of each individual is deduced. This step is called genotyping as used in SNP detection. The copy number of a specific sample is initially estimated by two times the median RD of all the candidate windows in this region. Then, the copy numbers of all samples of a CNVR are decomposed into several Gaussian distributions. The expectation maximization (EM) algorithm is used to estimate the model parameters, and the Dirichlet Process is used to infer the effective number of components. The silhouette coefficient is calculated for each CNVR as the quality control of the genotyping. The python package scikit-learn v0.19.0 [44] is used to implement the above algorithms. This genotyping step could be sequential or parallel, and the parameter "nproc" is used to control the number of processes. The genotyping of 232 goats took 17.49 minutes and used 488 MB of memory on one node with two processors. The final output is the variant call format (VCF) file and can be analysed by SNP-based population genetic software.

Performance evaluation

Competing methods. Most of the validations were based on the 30 human BAM files from the 1000 GP Phase 3 unless otherwise noted. The performance of CNVcaller was compared with two pipelines, including CNVnator_v0.3.3 [18], which is well used in animal population CNVR detection, and Genome STRiP (included in svtoolkit_2.00.1696) [25], which is the state-of-the-art human population CNV detector. The recommended parameters and quality controls were used. For Genome STRiP, both the deletion and CNV pipelines were performed. The unplaced scaffolds were excluded, and the whole genome was separated by chromosomes as recommended. The

standard screens were applied to select the passing sites and remove the duplicated calls. For CNVnator, a 400 bp window was used as recommended. The gap regions and calls with a p value less than 0.01 were removed. We also used the q0 filter to remove any predictions with q0 < 0.5 (reads with multiple mapping locations) as recommended. The individual CNVs of all samples were merged into the population CNVRs by the following arbitrary standards: two calls have >50% reciprocal overlap with each other or >90% of one call is covered by another call [18,32]. Then, the CNVRs were genotyped by the built-in function of CNVnator.

Sensitivity validation. Sensitivity was defined by the number of CNVs called by both the CNV predictions and the high-confident CNVR database (>50% reciprocal intersection) out of the total known CNVs of the particular samples in the database. The calls with <=2,500 bp and alternative allele frequency <5% and sex chromosomes were removed from this study. Two previously published databases, including the same samples from the test data, were used. One is the 1000 GP CNVR map [2], which included 26 tested samples, and the other is the array comparative genomic hybridization (aCGH) based CNVR database [1], which included 10 tested samples. The CNVRs of the specific samples were extracted from the database and were then screened by the same length and frequency as the detected CNVRs (length >2,500 bp and alternative allele frequency ≥0.05). The intersected length of the predicted CNVRs and the high-confident CNVR database was calculated by the bedtools v2.25.0 [45].

Accuracy validation. The intensity rank-sum (IRS) test (included in the svtoolkit_2.00.1696) was performed based on the intensity data of the Affymetrix SNP 6.0 array, including 26 test samples.

The segmental duplication (SD) regions were removed [25] because the probe design did not cover the high similarity regions. The genotyping accuracy was calculated based on the aCGH CNVR database [1]. We took an intersection of the predicted regions and the aCGH database using bedtools. The predicted CNVs were considered as subject to validation if the predicted regions have a >90% reciprocal intersection with one CNVR in the database. The predicted copy number was in exact agreement with the integer genotyping from the aCGH database, which was defined as correct. The Mendelian inconsistencies were calculated from the deleted and biallelic duplicated CNVRs (maximum copy number < =4) in the Dutch families and sheep trios.

Sheep genotyping validation by CNVplex assay. A total of 73 sheep, including Merino, Texel, Mongolia and Tibetan sheep, were used for genotyping validation. Genomic DNA was extracted from the peripheral blood using the QIAamp DNA blood mini kit (Qiagen, Germany). Resequencing (~10X) was performed for each sheep, and the CNVRs were detected by CNVcaller as described above. The predicted CNVRs, with a high variation frequency, were selected for the validation. The copy numbers were validated by CNVplex® (Genesky Biotechnologies Inc., Shanghai, China), which is based on double ligation and multiplex fluorescence PCR [46]. The probes were designed to target the candidate windows of the target CNVR. The sizes of the PCR fragment and target loci sequences in each reaction are listed in **Supplementary Table 2.** The amplified probes were detected as fluorescent signals, and the peak areas were compared and normalized to determine the dosage of each target.

Absolute copy number validation

Detecting X-origin scaffolds. The unplaced scaffolds with a high sequence similarity with the X chromosome were regarded as X-origin scaffolds. All the scaffolds of OAR v3.1 were mapped to the X chromosome of the sheep reference genome OAR v4.0, the goat reference genome ARS1 and the cattle reference genome UMD 3.1 using BLASR [47]. If the best hit of a scaffold had a coverage >50% with >90% identity and >3 Kb length, these scaffolds were defined as the putative X-origin scaffolds. In theory, all these scaffolds were expected to be detected as high frequency CNVRs because the RDs of the unplaced scaffolds were not corrected by sex. The detection and genotyping accuracy in the SD region was estimated by the sex information of 133 sheep.

mrsFAST alignment. The pair-end reads with multiple hits indicated by the "XA" tag in the BWA alignment were selected for realignment by mrsFAST_v3.3.10 [48]. The mrsFAST alignment was performed basically as previously described [49]. Longer reads were trimmed to 40 bp to reduce the read length heterogeneity prior to the sequence alignment. After alignment, the reads with more than 20 mapped hits were excluded to remove the low complexity regions.

Simulations of the SD region. To evaluate the sensitivity and accuracy of the absolute copy number correction in the putative SD regions, simulations were carried out. The source sequence was derived from a randomly selected 50 Mb single copy region of chr1 from the sheep reference (OAR v3.1). To simulate the putative SD regions, we randomly extracted 100 non-overlapping regions of 5,000 bp and artificially inserted a tandem duplication into the reference genome. In

these putative SD regions, 2-6 copies were randomly assigned to 100 individuals, and all other regions are treated as normal copy regions.

The wgsim read simulator was used to sample the reads, assuming a 2% sequencing error rate, a 500 bp insert size with a standard deviation of 50 bp and a 100 bp read length under different coverage according to the copy number. The coverage of the normal regions was set to 20X. All the simulated reads were mapped to the modified source sequence using Burrows-Wheeler Aligner (BWA)[50] with the default parameters. The final BWA alignment file was used for calling CNVs by CNVcaller. The outputs about the position and copy number were compared with the ground truth.

Results and discussion

Computational cost in complex genomes from large population based studies

The robustness of CNVcaller was validated by the real sequencing data of the different genomes. The individual RD processing step of CNVcaller was compared to CNVnator, which detects CNVs individually. The processing time of CNVcaller was linearly related to the genome size and sequencing coverage: 20-40 minutes for a 3 Gb genome with 10X coverage **(Supplementary Table 3)**. However, the processing time of CNVnator rose exponentially with the scaffold number, which became the only index of time consuming when the scaffold number exceeded one thousand **(Figure 2A)**. Consequently, CNVcaller achieved a 145-fold speed increase from CNVnator for goat CNV detection. Noteworthy, the goat reference genome ARS1, which contains nearly 30 thousand scaffolds, was newly assembled by single-molecule sequencing [38].

The memory requirement of CNVcaller is extremely low and is mainly related to the genome size: only approximately 500 MB for a mammalian genome, which is less than one twentieth of CNVnator **(Figure 2B)**. Therefore, in multi-sample CNV detection, more than 20 missions of the individual RD processing step can be run in parallel on one node to further reduce the running time. The population-level performance of CNVcaller was evaluated and benchmarked by Genome STRiP, which also detects CNVRs at the population level. After removing the unplaced scaffolds, CNVcaller was still 3.5-7.8 times faster than Genome STRiP **(Figure 2C)**, with a 70% ~86% reduction in memory requirement **(Figure 2D)**. CNVcaller can complete the CNV detection of 232 goats, with a mean coverage of 12X, in 1.4 days on one node.

The high efficiency of CNVcaller facilitated the CNV detection in large populations. The robustness of CNVcaller also reduces the restrictions of the quality of reference genome, which will promote CNV research of the species with a draft assembly at the scaffold level. More importantly, this feature enables that CNVcaller is efficient and friendly to detect the present/absent variations for pan-genomes. Defined as the entire set of genes possessed by all members of a particular species, pan-genomes reveal numerous functional important genes unplaced on one single reference genome[51-53].

Absolute copy number correction in the putative SDs of sheep genome

Previous studies showed high proportion of SDs in animal genomes are mis-assembled single copy regions [28,29]. So, we validated the copy numbers on human (hg19) and sheep (OAR v3.1) reference genome assembly by the sequencing copy number of a human (NA12878) and a Tan sheep sample **(Figure 3A)**. If the SDs were correctly assembled, the sequencing diploid copy

number should be two times of the copy number of SDs. For example, the average sequencing copy number of the two-copy SDs was four in NA12878. However, the corresponding sequencing copy number of sheep was only 2.4. These results indicated most two-copy SDs of hg19 were truly duplicated in NA12878 while approximately 80% of the two-copy SDs in OAR v3.1 were unique regions in the Tan sheep sample. So, the SDs in sheep genome were called "putative SDs" before validation.

CNV detection is confounded by the presence of false SDs. Due to random placement of multiple mapped reads, the RD signal in these regions is effectively smeared over all copies therefore the copy number is under estimated. At the putative two-copy SD regions, the main peak of the copy numbers was one, same as heterozygotic deletions **(Figure 3B, yellow)**. CNVcaller implied an absolute copy number correction by simply adding the RD of the putative SDs to deduced the absolute copy numbers independent from the copy number on the genome assembly **(Figure 1)**. Based on the BWA alignments, this correction takes only 0.06 core-hour for a mammalian genome with 10X sequencing coverage. The results are similar with remapping using the multi-hit alignments mrsFAST **(Supplementary Figure 2)**. However, the principle of mrsFAST was realignment of reads. More than 10 hours were needed for the same data. After correction, the main peak of the copy numbers shifted to two at the putative two-copy SDs **(Figure 3B)**. Moreover, this peak separated more clearly with the true duplicated regions (with diploid copy number four). The simulated data also showed the absolute copy number correction can reduce the STDEV of each simulated copy number **(Supplementary Table 4)**.

In the real dataset, CNVcaller detected more duplications in the SDs of the sheep genome with only 3% Mendelian inconsistence **(Figure 3C, Supplementary Figure 3)**. Because the lack of

validated sheep CNVR database, the sensitivity was validated indirectly. Based on our integrated analysis (see method), there are 138 sheep X chromosome origin scaffolds, which were not anchored onto chromosomes of OAR v3.1. Therefore, all of these scaffolds should be detected as CNV because the rams had half copy numbers of ewes. As a result, CNVcaller detected 101 out of these 138 X-origin scaffolds, with a sensitivity of 73%. Furthermore, the corrected copy numbers of these scaffolds were centralized at integer **(Figure 3D)**, whereas the peaks of the raw copy numbers were ambiguous because of splitting the raw RDs among the putative SDs **(Supplementary Figure 4)**. In contrast, CNVnator and Genome STRiP could not report these unmapped CNVRs.

Performance evaluations on sheep data

To evaluate the robustness and FDR in sheep, we used CNVcaller, CNVnator and Genome STRiP to detect the CNVRs from three sheep trios, respectively. Only 260 CNVRs were reported by CNVnator, while 3,386 CNVRs were detected by CNVcaller. More than 90% of the initial calls of CNVnator was removed by the gap filtering step. This is not surprising because sheep reference genome OAR v3.1 has ~125,000 gaps, while the human reference genome hg19 only has 354 gaps. CNVcaller removed the sliding windows with gaps at the first step, and finally the adjacent CNVRs were merged into one call if their RDs are highly correlated. These optimizations avoided the artefacts leaded by assembly errs and retained the adjacent CNVRs as well.

The accuracy was validated by the Mendelian inconsistencies of all the CNVRs on autosomes **(Figure 4)**. CNVcaller achieved higher accuracy than Genome STRiP in both deletion (1% versus 2%) and duplication (4% versus 7%). The detected CNVRs of CNVcaller and Genome

STRiP were further analysed against the length and alternative allele frequency **(Figure 4B)**. CNVcaller performed better in duplication detection, it can detect duplications <2.5 Kb, and the Mendelian inconsistence of longer calls were lower than Genome STRiP (3% versus 9% for 2.5Kb ~ 5Kb calls; 2% versus 5% for > 5Kb calls). On the other hand, Genome STRiP detected 1958 more < 2.5Kb deletions than CNVnator. One possible reason was Genome STRiP integrating RP methods which have higher capability in detecting shorter deletions. In terms of the frequency, because the detected samples were three trios, most CNVRs were medium frequency (6%-50%). The rare duplications tended to have a higher FDR than the median and high frequency calls **(Figure 4C)**. Currently, the main use of CNVcaller is to detect the CNVRs related to economy traits in livestock and crops. In these studies, the target CNVRs usually have a high frequency after long time breeding selection.

To investigate the reproducibility of CNVcaller, the CNVRs identified by CNVcaller from 133 sheep of 44 worldwide breeds were compared with the other two recently released large-scale sheep CNVR datasets. One is derived from allied breeds using multiple platforms, including aCGH, SNP chip and whole genome sequence [54], and the other is based on three Chinese sheep breeds using 600K SNP array [55]. The samples and platforms had a big influence on the results, so the overall intersection ratio was low. However, CNVcaller covered 51% of the cross validated region of the two datasets **(Figure 4 D)**.

The genotyping accuracy of 73 sheep was validated by a recently developed molecular biology technique, CNVplex **(Figure 5)**. This method reports the copy number of a genomic sequence based on the multiplex ligation-dependent probe amplification (MLPA) method [46]. When we compared the copy number predicted by CNVcaller from sequencing data and the

CNVplex result; the Pearson's product–moment correlation coefficients were higher than 0.95, and the genotype concordance was 98%**.**

Performance evaluations on 1000 Genomes Project data

Although CNVcaller were mainly designed for complex genomes, its performance was also benchmarked on 30 human BAM files from 1000GP. Because the SNP array and the highly-confident CNVR databases are available for the population level accuracy and sensitivity evaluation. First, CNVcaller demonstrated the highest overall accuracy for detecting duplications, and the FDR of CNVcaller are relative consistent across duplication length and frequency categories **(Figure 6A, B).** Whereas the short or singleton duplications of other two methods have high FDR. Second, 43% duplications detected by CNVnator were >20 kb. This was not due to the merged individual CNV to the CNVR, because the average size of individual calls was 3-4 times larger than the other methods. Third, Genome STRiP also showed the highest capability for detecting deletions, especially the short and rare ones, indicating the advantage of combining RD and RP methods in deletion. Besides directly combination of the two methods into one piece of software, another option was using CNVcaller's high-confidence RD results as the prior to improve the accuracy of the read pair/split read pipeline [22].

The genotyping accuracy of the human data was benchmarked against the integer copy numbers of the high-confident aCGH array based database. The discordance rate of CNVcaller, CNVnator and Genome STRiP were 2.6%, 5.5% and 2.2%, respectively. This genotyping accuracy ranking was concordance with the Mendelian inconsistence of the 10 Dutch trio **(Supplementary Figure 5)**.

The sensitivity of human data was estimated as the proportion of the high-confident CNVR database that overlapped by the predicted CNVRs. Two previously published high-confident databases, including the particular samples, were the aCGH-based CNVR database [1] and the 1000GP CNVR map [2]. For the highly variable FDR, the sensitivity estimation removed the calls that were <=2,500 bp and had an alternative allele frequency <5%. For the aCGH database, CNVcaller demonstrated the highest sensitivity (57%) for duplication, 14% and 26% higher than Genome STRiP and CNVnator. Whereas Genome STRiP achieved the highest sensitivity (74%) in deletions, 8% and 2% higher than CNVcaller and CNVnator **(Figure 6C)**. For the 1000 GP CNV maps, even though both Genome STRiP and CNVnator were the core methods of creating the library, the sensitivity of CNVcaller were 68% and 67% for deletions and duplications, only 4%-10% lower than Genome STRiP and CNVnator.

With the same input data, the three methods have a high proportion of intersection with each other. The number of overlapped (>50%) calls of each of the two methods was 429 (CNVcaller vs CNVnator), 502 (CNVcaller vs Genome STRiP) and 513 (CNVnator vs Genome STRiP). Because the intersection of the three methods was hard to define in number so they are showed in length. CNVcaller covered 40% of the CNVRs detected by CNVnator, 45% of Genome STRiP and 65% of their intersection **(Figure 6D)**.

Conclusion

CNVcaller was designed to detect the CNVRs from large scale resequencing data from all types of genomes. The general applicable detection and correction algorithms have greatly increased the computational efficiency in complex genomes. The validation of the sheep genomes showed that

the absolute copy number correction multiplied the detection efficiency of the misassembled SD regions with a great reduction in the running time and deduced more reasonable copy numbers. Based on the evaluations from sheep and human studies, CNVcaller achieved the best accuracy and sensitivity for detecting duplications. This rapid and reliable population-level CNV detection promotes the discovery of the missing heritability of complex traits and the accurate determination of the causative mutations for more species.

Availability and requirements

Project name: CNVcaller

Project home page: http://animal.nwsuaf.edu.cn/software

https://github.com/JiangYuLab/CNVcaller

Operating system(s): platform independent

Programming language: Perl, C++

Other requirements: Samtools 1.3 (using htslib 1.3), scikit-learn v0.19.0

License: GNU General Public License, version 3.0 (GPL-3.0)

Conflict of interest

The authors declare that they have no competing interests

Author contributions

WXH and JY designed this software; ZZQ and CT wrote the code; WXH and ZZQ improved the pipeline structures; ZZQ and CYD tested the software prototype; LC and FWW contributed to the data organization; WXH and JY drafted the manuscript. All authors read and approved the final manuscript.

Acknowledgements

This work is supported by grants from National Natural Science Foundation of China (31572381), and the National Thousand Youth Talents Plan. We thank the International Sheep Genomics Consortium (ISGC) for access to the unpublished sheep sequencing data provided under the Toronto guidelines for data users. We thank for the NextGen Project for the goat sequencing data. We thank for the Genomes of Netherlands (GoNL) project for the human family data.

Figure Legends

Figure 1 CNVcaller algorithm flowchart (left) and the key algorithms of each step (right). (1) Individually RD processing. In the absolute copy number correction, the RDs of highly similar windows were added together to deduce the absolute copy number. (2) Multi-criteria CNVR selection. The curves show the copy numbers in a specific region for multiple samples. The blue transverse boxes mark the windows with a significantly distinguishing copy number from the average (individual criterion). The green vertical boxes indicate that these regions meet the frequency conditions, and the red frame indicates that the RDs between the two adjacent windows are significantly correlated (population criteria). The forth bar from the left, satisfying all the

above conditions, is selected as the CNVR. (3) Genotyping: The copy numbers in each CNVR are clustered by mixture Gaussian model to distinguish the normal, heterozygous and homozygous samples.

Figure 2 Computational performance of CNVcaller, CNVnator and Genome STRiP. All the programs were executed on one node with two 2.40-GHz Intel Xeon E5-2620 v3 processors. (A, B) Log plots of the processing time (A) and the max memory (B) for one individual. The numbers of unplaced scaffolds of the reference genome are indicated in brackets. The processing time was normalized by the genome size and sequencing coverage to simulate a 3 Gb genome with 5X or 10X sequencing coverage. (C, D) Log plots of the total running time (C) and the max memory (D) of the population CNVR detection. The test cohorts are as follows: 8 sheep, 30 humans and 232 goats, with 19X, 16X and 12X average sequencing coverage, respectively. In Genome STRiP, the unplaced scaffolds were excluded.

Figure 3 Absolute copy number correction in the sheep genome. (A) The RDs of a human (NA12787) and sheep was calculated by 800 bp sliding window and the sequencing copy number was deduced. The copy numbers were plotted against the copy number on the reference genome assembly of human and sheep. Compared with humans, the sheep sample had much lower copy numbers in the putative duplicated regions than expected. (B) The distribution of the copy numbers of the putative two-copy regions in the sheep genome before and after the absolute copy number correction. After correction, the main peak of the copy number shifted to two (normal diploid copy number). The smaller peaks at four, after correction, indicated the 20% real

segmental duplications. (C) The number and Mendelian FDR of CNVRs residing in the SD regions (>50% overlap with the SD regions). The sheep SD regions include the regions longer than 2 Kb with >97% identity. (D) The raw and corrected copy numbers of all the X-origin scaffolds of 133 sheep.

Figure 4 Accuracy and reproducibility of the sheep data. (A) The Mendelian inconsistency of the 3 sheep trios. The number of detected deletions and duplications and the FDR of the calls are shown by the bar plot, respectively. (B) The bar plots of the number of calls and IRS FDR partitioned by the CNV length. All calls on the autosomes were included. (C) The bar plots of the number of calls and IRS FDR partitioned by alternative allele frequency. The frequency is shown by the CNV allele number (D) Overlap of the length of the CNVRs (by Mb) detected by CNVcaller and two other large-scale sheep CNVR studies using different approaches and platforms.

Figure 5 Evaluation of the sheep CNV genotypes by CNVplex. Two duplicated (A, B) and two deleted (C, D) CNVRs with a high variation frequency were typed in CNVplex using 73 sheep samples. The copy number genotypes predicted by CNVcaller from the sequencing data were plotted against the measurements of CNVplex of the same animal.

Figure 6 Accuracy, genotyping, reproducibility and sensitivity on the 1000GP data. (A) The bar plots of the number of calls and the IRS FDR partitioned by CNV length. All the calls on the autosomes were included. (B) The bar plots of the number of calls and the IRS FDR partitioned by

alternative allele frequency. To eliminate the huge FDR diversity of the short CNVs, the effect of the allele frequency was evaluated using the >2.5 Kb calls. (C) The sensitivity (the proportion of high-confident CNV database overlapped by predicted CNVs) of the three methods. (D) Overlap of the length of the CNVRs (by Mb) detected by CNVcaller, CNVnator and Genome STRiP based on the same 30 BAM files from the 1000GP.

References

1. Conrad DF, Pinto D, Redon R, Feuk L, Gokcumen O, et al. (2010) Origins and functional impact of copy number variation in the human genome. Nature 464: 704-712.

2. Sudmant PH, Rausch T, Gardner EJ, Handsaker RE, Abyzov A, et al. (2015) An integrated map of structural variation in 2,504 human genomes. Nature 526: 75-81.

3. Chiang C, Scott AJ, Davis JR, Tsang EK, Li X, et al. (2017) The impact of structural variation on human gene expression. Nature genetics.

4. Khurana E, Fu Y, Colonna V, Mu XJ, Kang HM, et al. (2013) Integrative annotation of variants from 1092 humans: application to cancer genomics. Science 342: 1235587.

5. Stefansson H, Meyer-Lindenberg A, Steinberg S, Magnusdottir B, Morgen K, et al. (2014) CNVs conferring risk of autism or schizophrenia affect cognition in controls. Nature 505: 361-366.

6. Sudmant PH, Mallick S, Nelson BJ, Hormozdiari F, Krumm N, et al. (2015) Global diversity, population stratification, and selection of human copy-number

variation. Science 349: aab3761.

7. Norris BJ, Whan VA (2008) A gene duplication affecting expression of the ovine ASIP gene is responsible for white and black sheep. Genome research 18: 1282-1293.

8. Giuffra E, Törnsten A, Marklund S, Bongcam-Rudloff E, Chardon P, et al. (2002) A large duplication associated with dominant white color in pigs originated by homologous recombination between LINE elements flanking KIT. Mammalian Genome 13: 569-577.

9. Wright D, Boije H, Meadows JR, Bed'Hom B, Gourichon D, et al. (2009) Copy number variation in intron 1 of SOX5 causes the Pea-comb phenotype in chickens. PLoS Genet 5: e1000512.

10. Seo B-Y, Park E-W, Ahn S-J, Lee S-H, Kim J-H, et al. (2007) An accurate method for quantifying and analyzing copy number variation in porcine KIT by an oligonucleotide ligation assay. BMC genetics 8: 81.

11. Zhou Z, Jiang Y, Wang Z, Gou Z, Lyu J, et al. (2015) Resequencing 302 wild and cultivated accessions identifies genes related to domestication and improvement in soybean. Nature biotechnology 33: 408-414.

12. Chen K, Wallis JW, McLellan MD, Larson DE, Kalicki JM, et al. (2009) BreakDancer: an algorithm for high-resolution mapping of genomic structural variation. Nat Meth 6: 677-681.

13. Hormozdiari F, Hajirasouliha I, Dao P, Hach F, Yorukoglu D, et al. (2010) Next-generation VariationHunter: combinatorial algorithms for transposon insertion

discovery. Bioinformatics 26: i350-i357.

14. Ye K, Schulz MH, Long Q, Apweiler R, Ning Z (2009) Pindel: a pattern growth approach to detect break points of large deletions and medium sized insertions from paired-end short reads. Bioinformatics 25: 2865-2871.

15. Rausch T, Zichner T, Schlattl A, Stütz AM, Benes V, et al. (2012) DELLY: structural variant discovery by integrated paired-end and split-read analysis. Bioinformatics 28: i333-i339.

16. Zhao M, Wang Q, Wang Q, Jia P, Zhao Z (2013) Computational tools for copy number variation (CNV) detection using next-generation sequencing data: features and perspectives. BMC bioinformatics 14: S1.

17. Xie C, Tammi MT (2009) CNV-seq, a new method to detect copy number variation using high-throughput sequencing. BMC bioinformatics 10: 80.

18. Abyzov A, Urban AE, Snyder M, Gerstein M (2011) CNVnator: an approach to discover, genotype, and characterize typical and atypical CNVs from family and population genome sequencing. Genome research 21: 974-984.

19. Szatkiewicz JP, Wang W, Sullivan PF, Wang W, Sun W (2013) Improving detection of copy-number variation by simultaneous bias correction and read-depth segmentation. Nucleic acids research 41: 1519-1532.

20. Chen K, Chen L, Fan X, Wallis J, Ding L, et al. (2014) TIGRA: A targeted iterative graph routing assembler for breakpoint assembly. Genome research 24: 310-317.

21. Zerbino DR, Birney E (2008) Velvet: Algorithms for de novo short read

assembly using de Bruijn graphs. Genome research 18: 821-829.

22. Layer RM, Chiang C, Quinlan AR, Hall IM (2014) LUMPY: a probabilistic framework for structural variant discovery. Genome biology 15: R84.

23. Redon R, Ishikawa S, Fitch KR, Feuk L, Perry GH, et al. (2006) Global variation in copy number in the human genome. Nature 444: 444-454.

24. Klambauer G, Schwarzbauer K, Mayr A, Clevert D-A, Mitterecker A, et al. (2012) cn. MOPS: mixture of Poissons for discovering copy number variations in next-generation sequencing data with a low false discovery rate. Nucleic acids research: gks003.

25. Handsaker RE, Van Doren V, Berman JR, Genovese G, Kashin S, et al. (2015) Large multiallelic copy number variations in humans. Nature genetics 47: 296-303.

26. Claros MG, Bautista R, Guerrero-Fernández D, Benzerki H, Seoane P, et al. (2012) Why assembling plant genome sequences is so challenging. Biology 1: 439-459.

27. Warr A, Hume D, Archibald AL, Deeb N, Watson M (2015) Identification of low-confidence regions in the pig reference genome (Sscrofa10. 2). Frontiers in genetics 6: 338.

28. Kelley DR, Salzberg SL (2010) Detection and correction of false segmental duplications caused by genome mis-assembly. Genome biology 11: 1.

29. Zimin AV, Kelley DR, Roberts M, Marçais G, Salzberg SL, et al. (2012) Mis-Assembled “Segmental Duplications” in Two Versions of the <italic>Bos taurus</italic> Genome. PLoS One 7: e42680.

30. Zhang X, Wang K, Wang L, Yang Y, Ni Z, et al. (2016) Genome-wide patterns of copy number variation in the Chinese yak genome. BMC genomics 17: 1.

31. Yi G, Qu L, Liu J, Yan Y, Xu G, et al. (2014) Genome-wide patterns of copy number variation in the diversified chicken genomes using next-generation sequencing. BMC genomics 15: 962.

32. Chain FJ, Feulner PG, Panchal M, Eizaguirre C, Samonte IE, et al. (2014) Extensive copy-number variation of young genes across stickleback populations. PLoS Genet 10: e1004830.

33. Zarrei M, MacDonald JR, Merico D, Scherer SW (2015) A copy number variation map of the human genome. Nature Reviews Genetics.

34. Consortium GotN (2014) Whole-genome sequence variation, population structure and demographic history of the Dutch population. Nature genetics 46: 818-825.

35. Badr Benjelloun FJA, Streeter I, Boyer F, Coissac E, Stucki S, et al. (2015) Characterizing neutral genomic diversity and selection signatures in indigenous populations of Moroccan goats (Capra hircus) using WGS data. Frontiers in genetics 6.

36. Dong Y, Zhang X, Xie M, Arefnezhad B, Wang Z, et al. (2015) Reference genome of wild goat (capra aegagrus) and sequencing of goat breeds provide insight into genic basis of goat domestication. BMC genomics 16: 431.

37. Dong Y, Xie M, Jiang Y, Xiao N, Du X, et al. (2013) Sequencing and automated whole-genome optical mapping of the genome of a domestic goat (Capra hircus).

Nature biotechnology 31: 135-141.

38. Bickhart DM, Rosen BD, Koren S, Sayre BL, Hastie AR, et al. (2017) Single-molecule sequencing and chromatin conformation capture enable de novo reference assembly of the domestic goat genome. Nature Genetics 49: 643-650.

39. Diez CM, Meca E, Tenaillon MI, Gaut BS (2014) Three groups of transposable elements with contrasting copy number dynamics and host responses in the maize (Zea mays ssp. mays) genome. PLoS Genet 10: e1004298.

40. Li H, Ruan J, Durbin R (2008) Mapping short DNA sequencing reads and calling variants using mapping quality scores. Genome research 18: 1851-1858.

41. McKenna A, Hanna M, Banks E, Sivachenko A, Cibulskis K, et al. (2010) The Genome Analysis Toolkit: a MapReduce framework for analyzing next-generation DNA sequencing data. Genome Res 20: 1297-1303.

42. Li H, Handsaker B, Wysoker A, Fennell T, Ruan J, et al. (2009) The Sequence Alignment/Map format and SAMtools. Bioinformatics 25: 2078-2079.

43. Kent WJ (2002) BLAT—the BLAST-like alignment tool. Genome research 12: 656-664.

44. Pedregosa F, Varoquaux G, Gramfort A, Michel V, Thirion B, et al. (2011) Scikit-learn: Machine learning in Python. Journal of Machine Learning Research 12: 2825-2830.

45. Quinlan AR, Hall IM (2010) BEDTools: a flexible suite of utilities for comparing genomic features. Bioinformatics 26: 841-842.

46. Zhang X, Xu Y, Liu D, Geng J, Chen S, et al. (2015) A modified multiplex

ligation-dependent probe amplification method for the detection of 22q11. 2 copy number variations in patients with congenital heart disease. BMC genomics 16: 364.

47. Chaisson MJ, Tesler G (2012) Mapping single molecule sequencing reads using basic local alignment with successive refinement (BLASR): application and theory. BMC bioinformatics 13: 238.

48. Hach F, Hormozdiari F, Alkan C, Hormozdiari F, Birol I, et al. (2010) mrsFAST: a cache-oblivious algorithm for short-read mapping. Nature methods 7: 576-577.

49. Bickhart DM, Hou Y, Schroeder SG, Alkan C, Cardone MF, et al. (2012) Copy number variation of individual cattle genomes using next-generation sequencing. Genome research 22: 778-790.

50. Li H, Handsaker B, Wysoker A, Fennell T, Ruan J, et al. (2009) The sequence alignment/map format and SAMtools. Bioinformatics 25: 2078-2079.

51. Li R, Li Y, Zheng H, Luo R, Zhu H, et al. (2010) Building the sequence map of the human pan-genome. Nat Biotechnol 28.

52. Monat C, Pera B, Ndjiondjop M-N, Sow M, Tranchant-Dubreuil C, et al. (2016) de novo assemblies of three Oryza glaberrima accessions provide first insights about pan-genome of African rices. Genome biology and evolution: evw253.

53. Li Y-h, Zhou G, Ma J, Jiang W, Jin L-g, et al. (2014) De novo assembly of soybean wild relatives for pan-genome analysis of diversity and agronomic traits. Nature biotechnology 32: 1045-1052.

54. Jenkins GM, Goddard ME, Black MA, Brauning R, Auvray B, et al. (2016) Copy number variants in the sheep genome detected using multiple approaches. BMC

genomics 17: 1.

55. Zhu C, Fan H, Yuan Z, Hu S, Ma X, et al. (2016) Genome-wide detection of CNVs in Chinese indigenous sheep with different types of tails using ovine high-density 600K SNP arrays. Scientific reports 6.

Figure1

Click here to download Figure Figure1.pdf

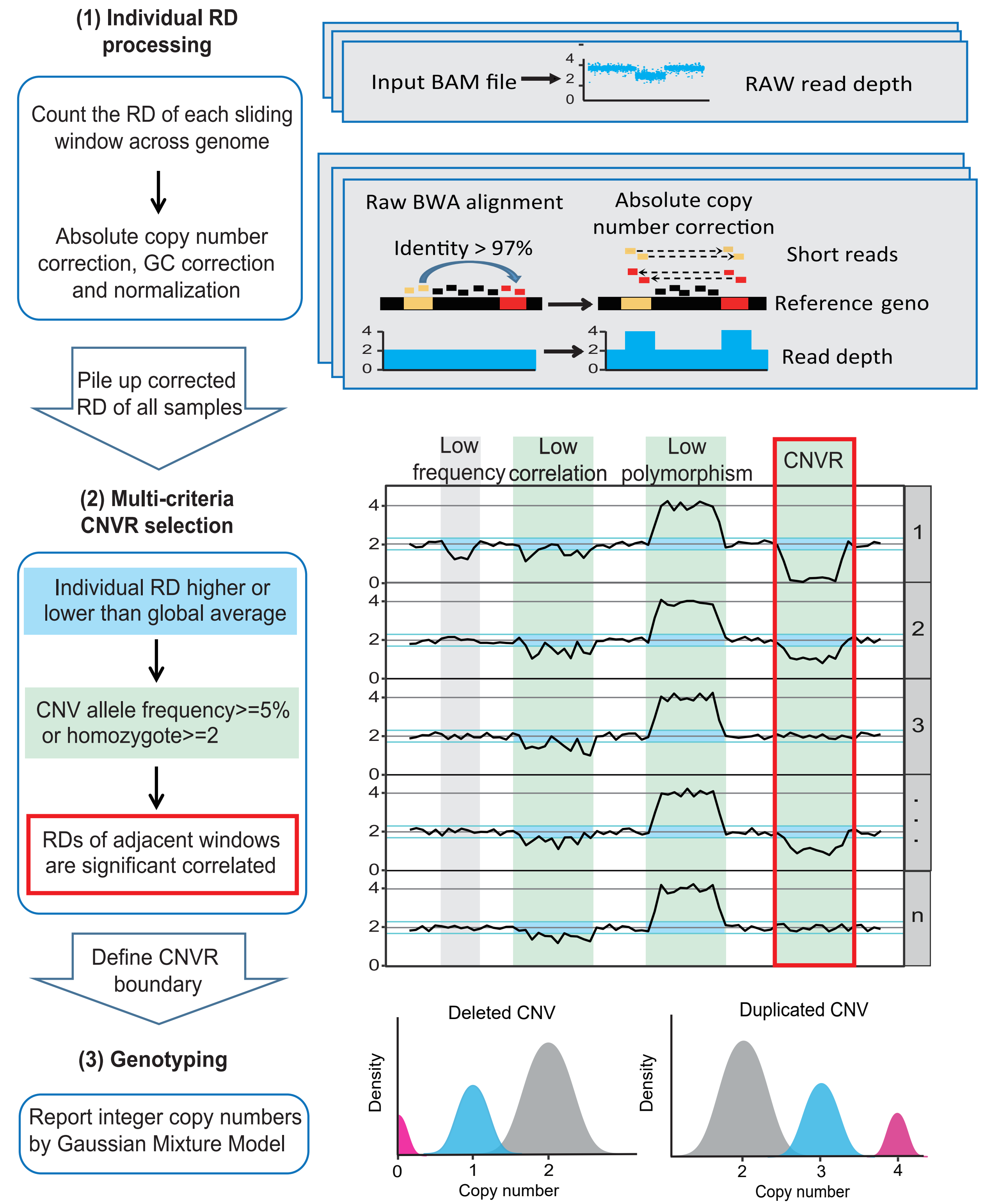

Figure2

Click here to download Figure Figure2.pdf

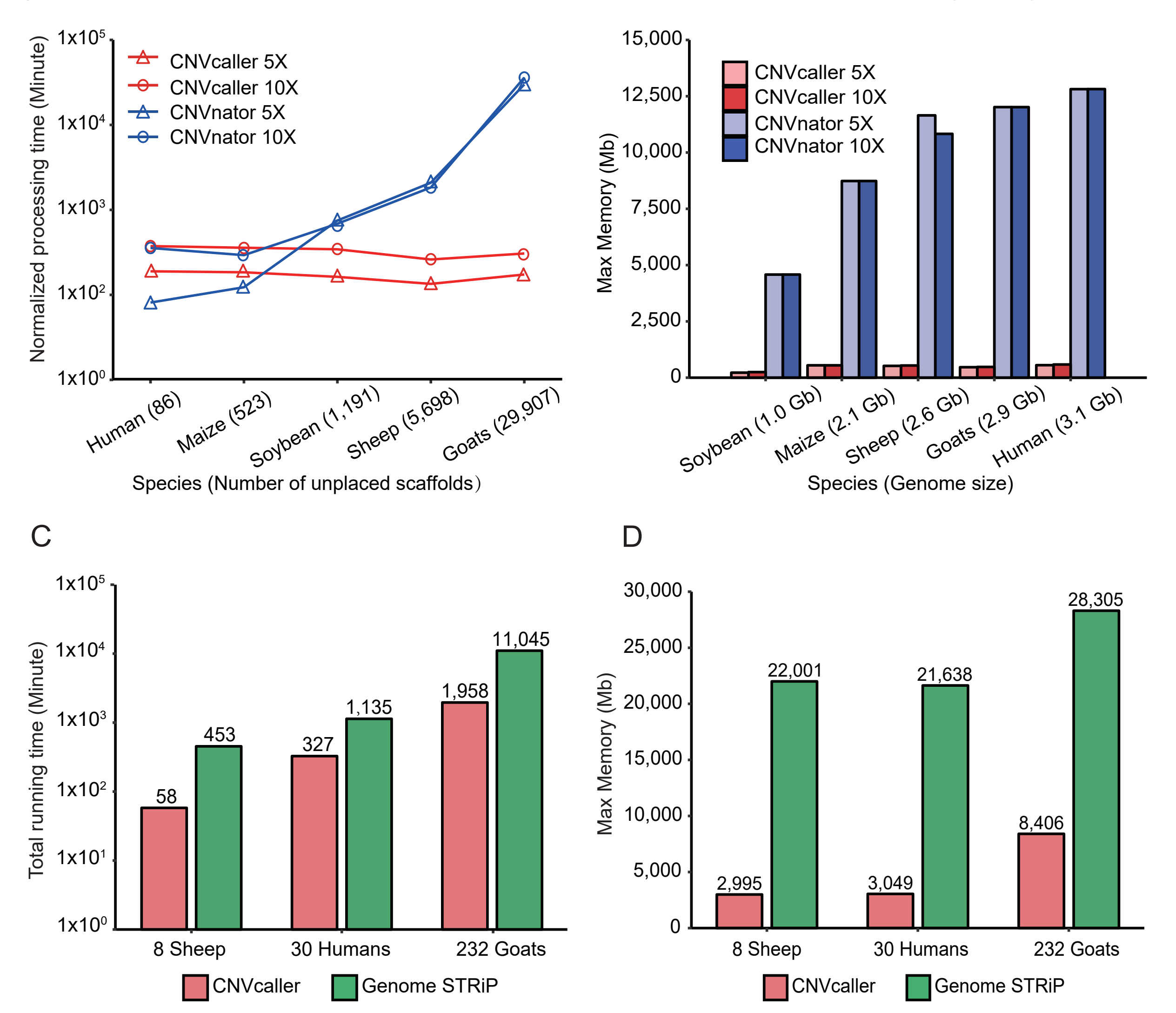

Figure3

Click here to download Figure figure3.new.pdf

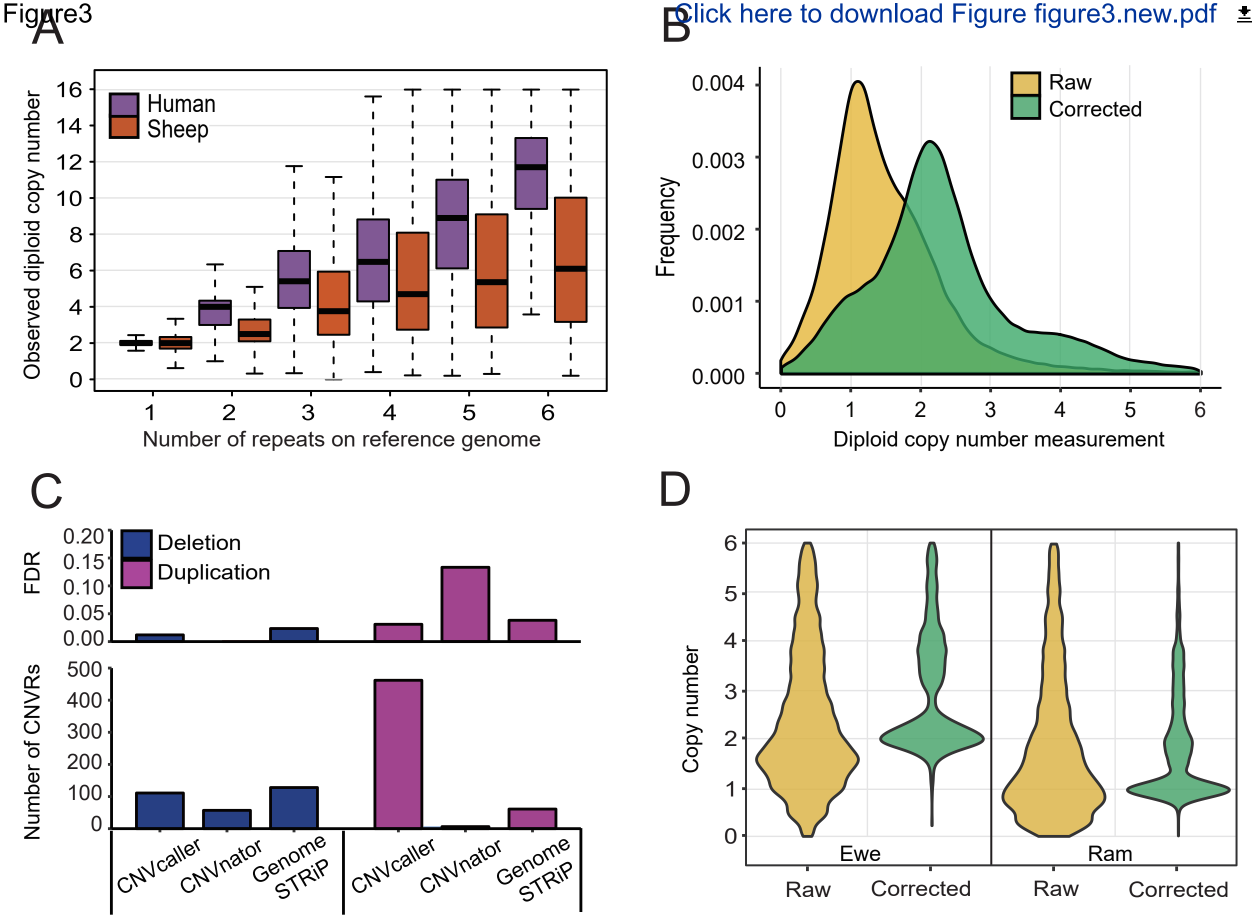

Figure4

Click here to download Figure figure4.new.pdf

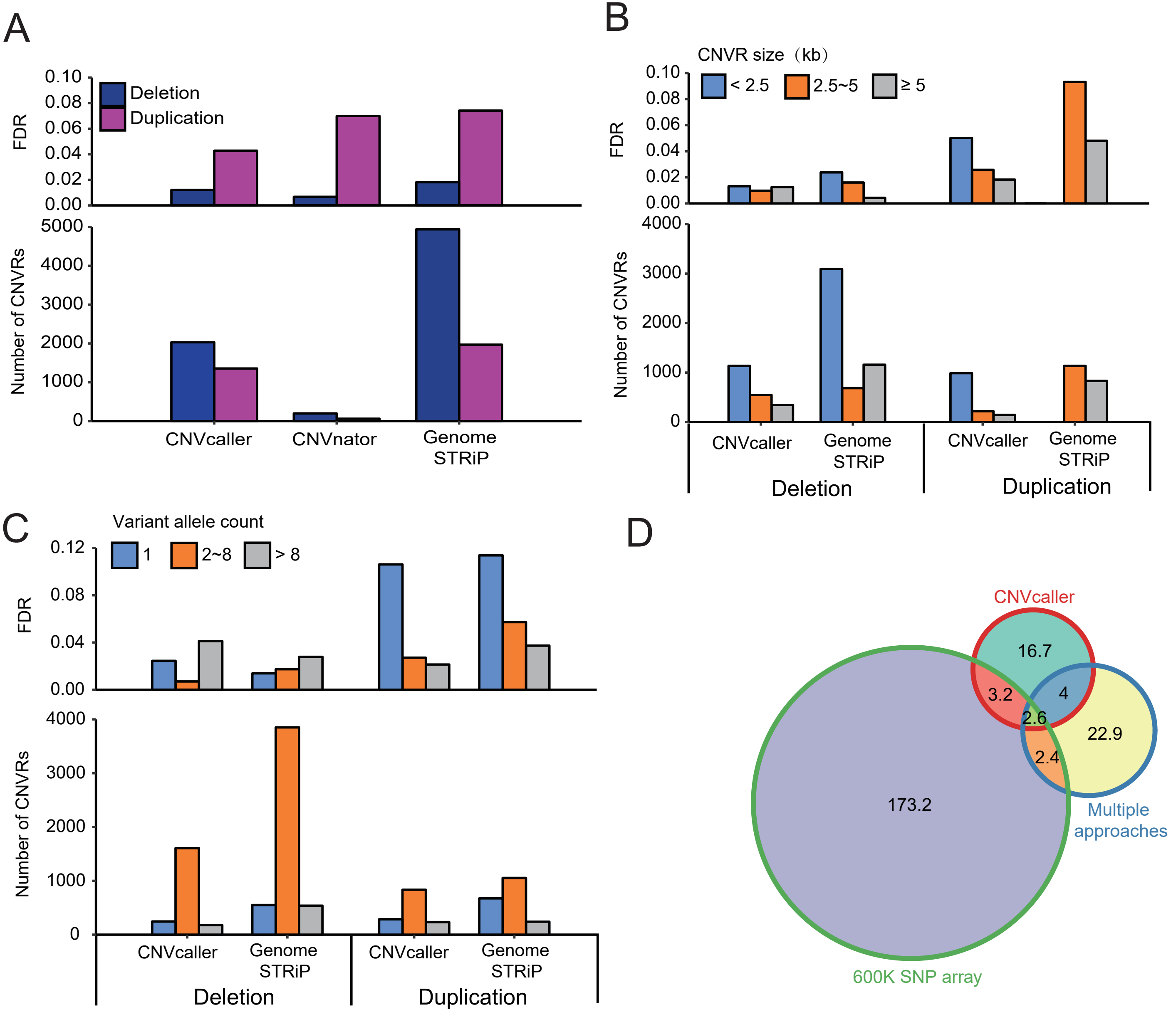

Figure5

Click here to download Figure Figure5.pdf

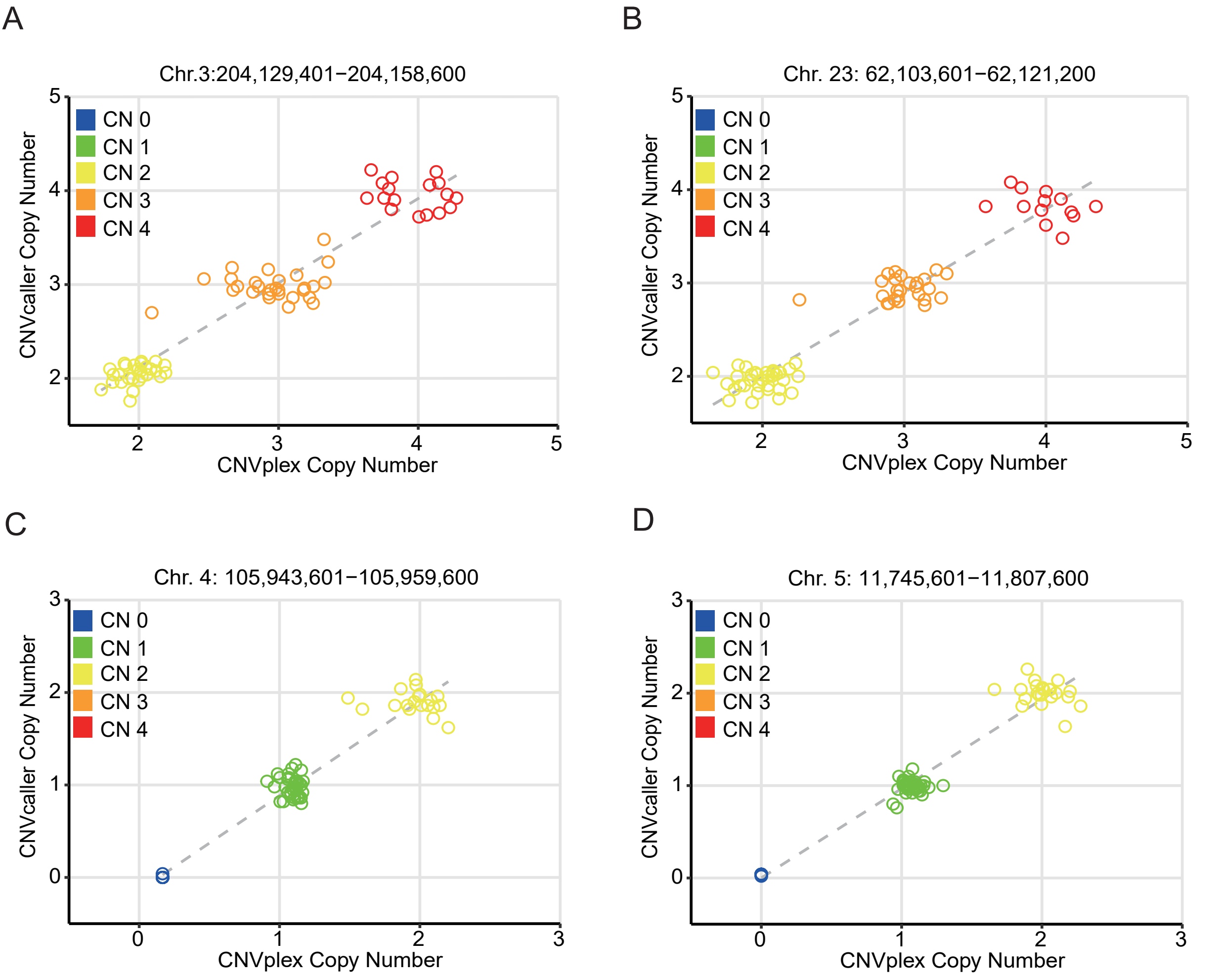

Figure6

Click here to download Figure figure6.new.pdf

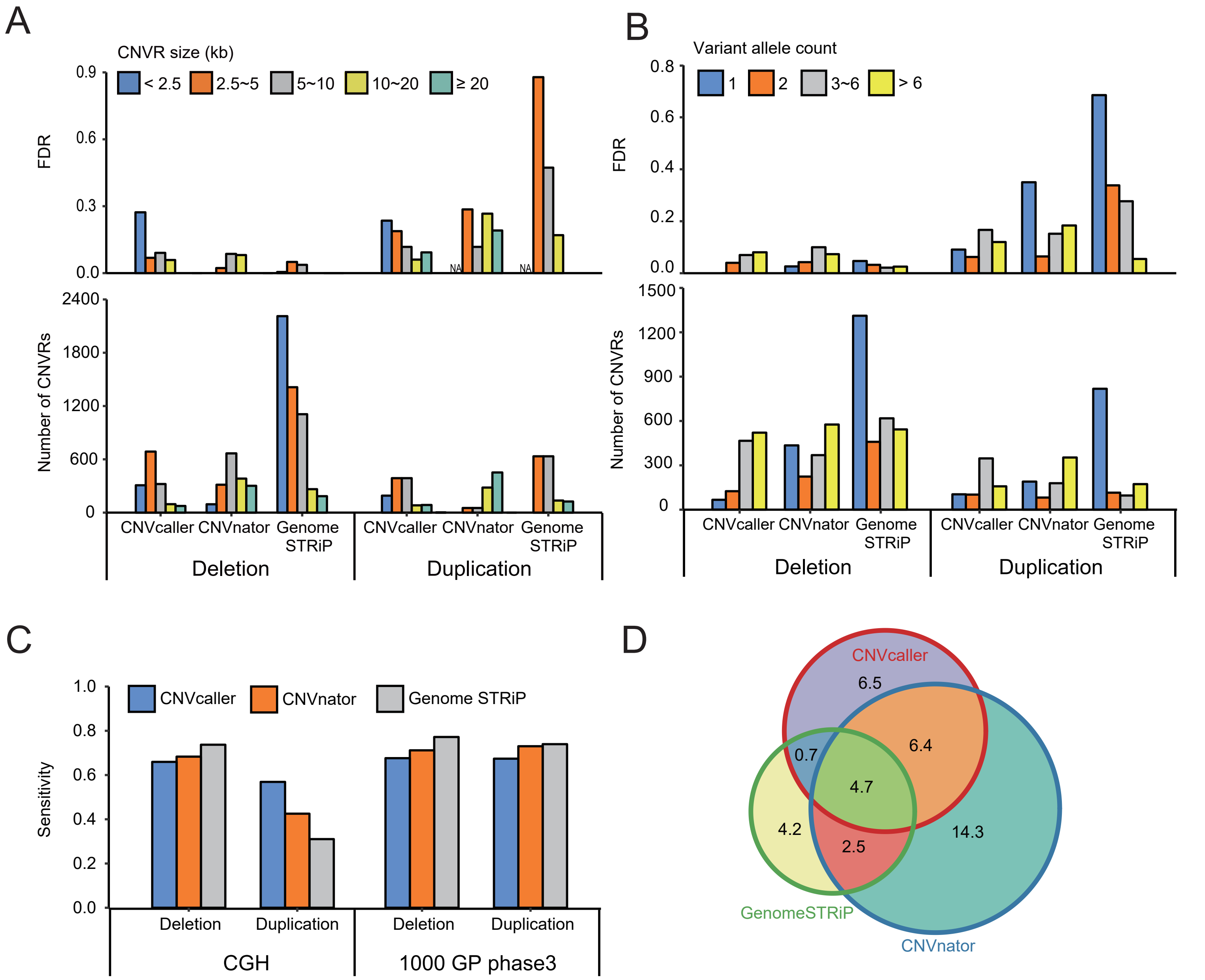

Click here to access/download
Supplementary Material
Supplementary Materials10_2_ZZQ.docx

GIGA-D-17-00119
CNVcaller: Highly Efficient and Widely Applicable Software for Detecting Copy Number Variations in Large Populations
Xihong Wang; Zhuqing Zheng; Yudong Cai; Ting Chen; Chao Li; Weiwei Fu; Yu Jiang
GigaScience

Dear Dr. Edmunds

Thank you very much for handling our manuscript "CNVcaller: Highly Efficient and Widely Applicable Software for Detecting Copy Number Variations in Large Populations " (GIGA-D-17-00119). We appreciate all the comments from the reviewers, which helped us improve our manuscript. We have now revised the manuscript according to the reviewers' comments and your instructions.

We addressed the comments and questions of the reviewers as explained below, the reviewers' text has been included and our responses are in colored italics. Revised text is indicated by quotation marks. Because several new figures have been added, we attach a list of the current figures and tables corresponding to those from last version so that the changes can easily be tracked.

Upon the suggestions of the reviewers, we modified the manuscript as follows:

1. *The newly released version of CNVcaller updated the genotyping method. The python package, scikit-learn v0.19.0, was used to decompose the reported copy numbers into several Gaussian distributions. Therefore, the accuracy of the CNVcaller in the new version was increased.*

2. *Since the reviewers required to evaluate the effects of the length and allele frequency of the discovered CNVRs. Two result sections have been added to analyze the number and FDR of the CNVRs detected by the three methods against the length and allele frequency. One section including Figure 4 was based on the sheep data, the other section including Figure 6 was based on the human data.*

3. *To answer the reviewer's question about the difference of the FDR in deletions and duplications, their FDR was evaluated respectively in the result sections.*

4. *The high-proportion mis-assembled segmental duplications in non-human*

assemblies caused misunderstanding of the reviewer. The section has been extensively redrafted, as analyzing both real and simulated data.

5. *The previous discussion sections has been merged to the result sections to reduce the length of the manuscript. The first part of the previous results has been moved to the method sections as suggested by the reviewer.*

6. *The language has been professionally edited by an English editing service agency, American Journal Experts (AJE). (Because the first version is inadequate, we are waiting for the second version.)*

Thank you again for all of your assistance.

Sincerely yours,

Yu Jiang, and other coauthors

****

REVIEWS

****

Reviewer #1:

The authors developed a new CNV caller pipeline which they called CNVcaller geared towards improved speed compared to existing CNV callers and improved accuracy for high complexity genomes. I commend the authors on their efforts to introduce improved algorithms and pipelines for an inherently difficult procedure, namely CNV calling. My comments are mostly suggestions for improvement as followss. Note, comments of the form (4:5 for example represent page 4, line 5).

Thanks for your positive comments and encouragement. We have substantially revised the manuscript upon your suggestions.

There are several grammatical errors which make the paper somewhat confusing. I would strongly recommend further extensive English editing.

We apologize for these mistakes. The manuscript has been professionally edited by an English editing service agency, American Journal Experts (AJE)..

My main criticism of the analysis is one that I have seen repeatedly of most other CNV calling publications, and that is there is no sensitivity analysis.

We are sorry for the ambiguous of the sensitivity tests, which were stuffed in previous Table1. In the revised manuscript, new figures and tests have been added. On general, CNVcaller demonstrated 57%-67% sensitivity for duplications, and 66%-68% for deletions in human data. The sensitivity in sheep data was ~73%.

The detailed descriptions were as follows:

"The sensitivity of human data was estimated as the proportion of the high-confident CNVR database that overlapped by the predicted CNVRs. Two previously published high-confident databases, including the particular samples, were the aCGH-based CNVR database and the 1000GP CNVR map. For the highly variable FDR, the sensitivity estimation removed the calls that were <=2,500 bp and had an alternative allele frequency <5%. For the aCGH database, CNVcaller demonstrated the highest sensitivity (57%) for duplication, 14% and 26% higher than Genome STRiP and CNVnator. Whereas Genome STRiP achieved the highest sensitivity (74%) in deletions, 8% and 2% higher than CNVcaller and CNVnator (Figure 6C). For the 1000 GP CNV maps, even though both Genome STRiP and CNVnator were the core methods of creating the library, the sensitivity of CNVcaller were 68% and 67% for deletions and duplications, only 4%-10% lower than Genome STRiP and CNVnator."

"Because the lack of validated sheep CNVR database, the sensitivity was validated indirectly. Based on our integrated analysis (see method), there are 138 sheep X chromosome origin scaffolds, which were not anchored onto chromosomes of OAR v3.1. Therefore, all of these scaffolds should be detected as CNV because the rams had half copy numbers of ewes. As a result, CNVcaller detected 101 out of these 138 X-origin scaffolds, with a sensitivity of 73%. Furthermore, the corrected copy numbers of these scaffolds were centralized at integer (Figure 3D), whereas the peaks of the raw copy numbers were ambiguous because of splitting the raw RDs among the putative SDs (Supplementary Figure 4). In contrast, CNVnator and Genome STRiP could not report these unmapped CNVRs."

The authors here also suggest various parameters throughout their paper for performing CNV calling, but there is no analysis of how the results change if these parameters are adjusted, i.e. no analysis of how robust your algorithm is to changes in the parameters.

Thank you for your suggestion. The FDR against the window size and allele frequency have been added in Supplementary Figure 1 and Figure 6B.

The following description was added in methods.

"The window size is an important parameter for the RD methods. CNVcaller uses half of the window size as step size. The optimal window size is 800 bp for 5-10X coverage human and livestock sequencing data (Supplementary Figure 1). The

recommended scales roughly inversely coverage, resulting in 400 bp windows for 20X coverage and 200 bp windows for 50X coverage."

As another example, Hong et al 27503473 has demonstrated that the biggest variability in calling CNVs is in terms of the CNV size. I suspect that the same can be said of CNVcaller. Please comment on what sizes of CNVs does CNV caller do well or poorly on.

Figure 4 and Figure 6 have been added to evaluate the effect of the length and frequency in sheep and human data. On general, the performance of CNVcaller was good for deletions and duplications >2.5 Kb, however poorly on < 2.5 kb.

The detailed comparisons in the manuscript are as follows:

"The detected CNVRs of CNVcaller and Genome STRiP were further analyzed against the length and alternative allele frequency (Figure 4B). CNVcaller performed better in duplication detection, it can detect duplications <2.5 Kb, and the Mendelian inconsistence of longer calls were lower than Genome STRiP (3% versus 9% for 2.5Kb ~ 5Kb calls; 2% versus 5% for > 5Kb calls). On the other hand, Genome STRiP detected 1,958 more < 2.5Kb deletions than CNVcaller. One possible reason was Genome STRiP integrating RP methods which have higher capability in detecting shorter deletions. In terms of the frequency, because the detected samples were three trios, most CNVRs were medium frequency (6%-50%). The rare duplications tended to have a higher FDR than the median and high frequency calls (Figure 4C)."

"First, CNVcaller demonstrated the highest overall accuracy for detecting duplications, and the FDR of CNVcaller are relative consistent across duplication length and frequency categories. Whereas the short or singleton duplications of other two methods have high FDR. Second, 43% duplications detected by CNVnator were >20 kb. This was not due to the merged individual CNV to the CNVR, because the average size of individual calls was 3-4 times larger than the other methods. Third, Genome STRiP also showed the highest capability for detecting deletions, especially the short and rare ones, indicating the advantage of combining RD and RP methods in deletion."

2:32 "the prevalent.." is a gross exaggeration. I think you mean "a prevalent".

Corrected as suggested.

2:35 I don't think you mean geometric. I did not comment on other grammatical/English errors as there were too many to list individually. I would highly recommend getting help with the English in this paper.

We apologize for these mistakes. The manuscript has been professionally edited by an English editing service agency, American Journal Experts (AJE).

3:53 "RD" is not defined.

We apologize for the missing. This description has been added to the introduction as follows.

"Read-depth (RD) means the depth of the coverage or the genomic region that can be calculated by the number of reads aligned [16]."

6:120 Give a brief description of how CNVnator handles GC bias. Also why 40% for the GC bias? Shouldn't this parameter be dependent on the organism of interest?

We apologize for not clearly describing the procedure. In general, the mean RD of windows with 40% percent GC is only used as the temporary standard in the GC correction step. It will be lost in the following normalization step: the GC corrected RDs of each window are divided by the global median RDs. Because the denominator is calculated from the RDs already corrected by the 40% GC windows, this parameter will be lost and is not necessarily dependent on the organism of interest.

The CG correction of CNVnator was the combination of the correction and normalization steps of CNVcaller. The equation is as follow:

$$RD^i_{corrected} = \frac{\overline{RD}_{global}}{\overline{RD}_{gc}} RD^i_{raw},$$

Where i is bin index, RD^{i}_{raw} *is raw RD signal for a bin,* $RD^{i}_{corrected}$ *is corrected RD signal for the bin,* $\overline{RD}_{global}$ *is average RD signal over all bins, and* $\overline{RD}_{gc}$ *is the average RD signal over all bins with the same GC content as in the bin.*

The commentary on certain genomes not being as complete as others is important. I suspect though that if a large percentage of the samples show a CNV in a genome that is newer or not as complete, then this observation may be more likely indicative of a problem with the reference. Can you comment?

If the detected CNVR has variation in population, which means the read depths can be separated into two or more normal distributions, this call is probably true even with high frequency. On the contrary, if all of the individuals show the same abnormal read depth, it suggests the reference individual is indeed different from the sampling population or have assembly problems.

7:145 I am not convinced Pearson's correlation is appropriate. Your data is likely to have outliers and non-normal data. A non-parametric test of correlation like Spearman's correlation (Kendall-Tau is likely too computational intensive), or performing correlation after 5 or 10% trimming may be more appropriate.

We tried to replace the Pearson's correlation with Spearman's correlation in the 30 BAM files from 1000 Genome Project data. However, after replacement the FDR doubled while the length of each calls reduced to half. A possible reason was the Spearman's correlation was calculated by sorting of the read depth. So, the diverged copy numbers of deletion or duplication individuals contribution no more than the subtle random mistakes of the normal individuals. In the low frequency CNVRs, the Spearman's correlation index was mainly contributed by the random mistakes of the normal copy individuals.

The trimming is also not recommended for similar reason. In the low frequency CNVRs, the individuals with abnormal copy number will be trimmed as outliers.

cn.MOPS (Klambauer et al, PMID: 22302147) uses a mixture of Poissons as opposed

to Gaussian Mixture Models for CNV detection. I suspect the mixture of Poissions will be superior to Gaussian Mixture Models when the read depths are low, and Gausssian mixtures may be more appropriate when read depths are high. How difficult is it to replace the Gaussian mixtures with Poisson mixtures and compare the performance? I feel that this analysis would be informative and potentially improve your algorithm.

Thank you for your suggestion. However, it is not easy to replace the distribution because the RDs after GC correction and normalization are not integer so they can not be directly treated as Poisson distributions. Basically, CNV caller recommended a proper window size to make the standard variation less than 30% of the mean RD, which will not fit the Poisson distribution for RDs. Besides, we used the RDs of 232 goats with 10X coverage to test the fitness of Gaussian distribution using omnibus test (packages). As a result, 88% windows accepted the null hypothesis at P=0.01 level. So, we believe the Gaussian Mixture Models was acceptable for the 10 X data.

The term "CNVR" is critical for understanding the algorithm, and requires more explanation of the term.

We apologies for missing this important concept. The explanation has been added to the introduction as follows.

"To compare the copy number of a particular region across the samples, the shared CNVs among individuals are needed, so the unified CNV regions (CNVRs) were merged from the individual CNVs."

It would be helpful to include some further discussion on where you see that CNVcaller works better or worse than existing CNV calling software.

Figure 2 showed the speed of CNVcaller was one to two orders of magnitudes higher than the other methods. Figure 4 and Figure 6 have been added to evaluate the effect of the length and frequency in sheep and human data. On general, the performance of CNVcaller was better for all sizes of duplications, however poorly on deletions < 2.5 kb.

9:180. The "arbitrary standards" require a citation.

Two citations were added.

1. Chain FJ, Feulner PG, Panchal M, Eizaguirre C, Samonte IE, Kalbe M, et al. Extensive copy-number variation of young genes across stickleback populations. PLoS genetics. 2014;10 12:e1004830.

2. Abyzov A, Urban AE, Snyder M and Gerstein M. CNVnator: an approach to discover, genotype, and characterize typical and atypical CNVs from family and population genome sequencing. Genome research. 2011;21 6:974-84.

Minor comment: Since speed seems to be a major selling point of the software, more details about running the software on a compute cluster or running algorithms in parallel in the documentation would be helpful.

A new part of "Parallel submission of individual RD processing" has been added to the methods with the principle and command as follows.

"CNVcaller processes the BAM file of each individual separately in the first step, so parallel submissions can be used to save the total running time. All the BAM files should be equally distributed in to N groups, and each group contains M files. The max N = the available processing cores. M = the total number of BAM files/ N. For example, the 232 goat BAM files were processed on a node with 32 processing cores and 128 GB of RAM. We distributed the 232 files into 20 groups, and each group contained 12 BAM files. The shell command for one group likes following:

```
#!/bin/sh

for i in {1..M}

  do bash Individual.Process.sh -b $i.bam -h $i -d dup -s sex_chromosome

done
```

After corrections and normalization, the comparable RDs of each sample are concentrated to an ~100 MB intermediate file and output. This design avoids the repeated calculation of the same individual in different populations."

****

Reviewer #2:

The proposed method "CNVcaller" enables the efficient discovery and genotyping of CNVs in large populations. One of the main benefits of the method is that it can handle draft genome assemblies with thousands of scaffolds. The computational benchmarks proof that the method is fast and memory efficient but the evaluation of the accuracy of the method is less convincing. Some details of the method remain vague and hinder an objective evaluation. Detailed comments of how to improve the manuscript are below:

Thank you for your affirmation. We are sorry for the ambiguous of the accuracy test, and have substantially revised the manuscript upon your suggestions. In the revised manuscript, the performance evaluation in previous Table 1 was described more detail and classify by the species in Figure 4 and Figure 6.

Comment 1 - The primary application of CNVcaller is the detection of CNVs in large populations. Population variant call sets are dominated by rare variants of rather small size. For instance, less than 20% of the 1000 Genomes structural variants have a population allele frequency >5% and almost 50% of the SVs are <2kbp in size despite the rather low coverage (~7x). CNVcaller is currently restricted to large CNVs (>2kbp) and common variants (>5% allele frequency), which is a major limitation for population genomic studies.

In the revised version, all detected calls were included in the IRS test. In fact, all three methods can report some short and rare CNVRs. However, the short and rare duplications made by Genome STRiP and CNVnator had extremely high FDR. So, we excluded these results from the previous version of manuscript as 1000 GP.

The newly added Figure 6 showed the shortest duplication reported by CNVnator and Genome STRiP was 2.8 kb and 2.5 kb, and the IRS FDR of 2.5-5kb calls are 29% and 88%, respectively. The FDR of >2.5kb singletons was 35% and 69% for CNVnator and Genome STRiP, respectively. These uncertain calls were also removed by the phase 3 extended SV release of 1000GP. After extra quality controls, the number of duplications in the released database are only 1/7 of deletions, and the median size was 36 kb, 17 times longer than deletions. Therefore, improving the accuracy of duplications on this foundation is meaningful for enrich the CNV database. The main improvement of CNVcaller is the accuracy of duplications. The FDR of 2.5 kb – 5 kb was reduced to 19%, and the >2.5kb singletons was reduced to

9%. However, the FDR were still higher than the longer and higher frequency calls.

Besides, the current main usage of CNVcaller is to detect the CNVRs related to economy traits in livestock and crops. In these populations, the target CNVs usually have a medium or high frequency after long time artificial selection. We believe the high-confident medium to high frequency reported by CNVcaller can contribute to the functional and breeding study of non-human studies.

The sensitivity increase of CNVcaller for the subset of common and large CNVs seems to be driven by an increased number of detected CNVs in SD regions (Figure 5C). SNP arrays have a low SNP density in SD regions and in the present Manuscript array SNP probes in SD regions have been removed entirely. The reported IRS FDR is therefore heavily biased against CNVs in SD regions and it thus seems mandatory to me to proof that this sensitivity increase for SD-associated CNVs is not leading to an inflated FDR.

Thanks for your suggestions. Figure 5C (New Figure 3C) was updated to show both the number and the Mendelian inconsistence of the detected CNVs in SDs. The inconsistence rate of the calls in SD regions made by CNVcaller was about 3%. The copy numbers of unique and SDs were also indirectly validated by the X-origin scaffolds of a 133-sheep population. In both validation dataset, one main reason for acceptable FDR in SDs was most SDs in sheep reference genome assembly is actually mis-assembled unique region.

The detailed description are as follows:

"In the real dataset, CNVcaller detected more duplications in the SDs of the sheep genome with only 3% Mendelian inconsistence (Figure 3C, Supplementary Figure 3). Because the lack of validated sheep CNVR database, the sensitivity was validated indirectly. Based on our integrated analysis (see method), there are 138 sheep X chromosome origin scaffolds, which were not anchored onto chromosomes of OAR v3.1. Therefore, all of these scaffolds should be detected as CNV because the rams had half copy numbers of ewes. As a result, CNVcaller detected 101 out of these 138 X-origin scaffolds, with a sensitivity of 73%. Furthermore, the corrected copy numbers of these scaffolds were centralized at integer (Figure 3D), whereas the peaks of the raw copy numbers were ambiguous because of splitting the raw RDs among the putative SDs (Supplementary Figure 4). In contrast, CNVnator and Genome STRiP could not report these unmapped CNVRs."

The Manuscript lacks a Figure that shows the size and allele frequency distribution of

the discovered CNVs in comparison to Genome STRiP and CNVnator. An estimate of breakpoint accuracy of CNVcaller would also be valuable.

Thanks for your suggestion. Figure 4 and Figure 6 have been added to evaluate the effect of the length and frequency in sheep and human data. On general, the performance of CNVcaller was better for all sizes of duplications, however poorly on deletions < 2.5 kb.

The breakpoint accuracy is an innate disadvantage of RD methods. Because the detailed situation within a window is not calculated. And the window size cannot be too small for the medium or low coverage sequencing data. We recommend the user to combine the read pair or split read methods to improve the breakpoint accuracy.

The detailed comparations in the manuscript are as follows:

"The detected CNVRs of CNVcaller and Genome STRiP were further analyzed against the length and alternative allele frequency (Figure 4B). CNVcaller performed better in duplication detection, it can detect duplications <2.5 Kb, and the Mendelian inconsistence of longer calls were lower than Genome STRiP (3% versus 9% for 2.5Kb ~ 5Kb calls; 2% versus 5% for > 5Kb calls). On the other hand, Genome STRiP detected 1958 more < 2.5Kb deletions than CNVnator. One possible reason was Genome STRiP integrating RP methods which have higher capability in detecting shorter deletions. In terms of the frequency, because the detected samples were three trios, most CNVRs were medium frequency (6%-50%). The rare duplications tended to have a higher FDR than the median and high frequency calls (Figure 4C)."

"First, CNVcaller demonstrated the highest overall accuracy for detecting duplications, and the FDR of CNVcaller are relative consistent across duplication length and frequency categories. Whereas the short or singleton duplications of other two methods have high FDR. Second, 43% duplications detected by CNVnator were >20 kb. This was not due to the merged individual CNV to the CNVR, because the average size of individual calls was 3-4 times larger than the other methods. Third, Genome STRiP also showed the highest capability for detecting deletions, especially the short and rare ones, indicating the advantage of combining RD and RP methods in deletion. Besides directly combination of the two methods into one piece of software, another option was using high-confidence RD results generated CNVcaller as the prior to improve the accuracy of the read pair/split read pipeline."

The Manuscript mentions mrsFAST for absolute copy number validation. I could not find any formal comparison of predicted copy-number by mrsFAST and CNVcaller but maybe I missed this?

Supplementary Figure 2 (Previous Supplementary Figure 1) showed the copy number calculated from mrsFAST and CNVcaller was similar. However, mrsFAST needed to realign all the multi-hit reads in BWA alignments, leading to significantly increased computational time. For example, mrsFAST needed 10 hours for a 3G genome with 10X sequencing data, whereas, CNVcaller only needed 4 minutes.

- Please add to Table 1 the number of CNV sites that could be assessed by the IRS method and what proportion of each call set could be evaluated using IRS. I also believe the IRS method reports p-values separately for deletions, duplications and multi-allelic CNVs. Was there any difference among these for CNVcaller?

The detailed information of 1000GP calls including the required information has been added to Supplementary Table 5. Overall, 28%, 30% and 60% CNVRs of CNVcaller, CNVnator and Genome STRiP covered at least one probe of Affymetrix SNP 6.0 array, therefore can be assessed by IRS test. One main reason for the diverged testable proportion was only 4% of Genome STRiP calls were overlap with SDs which have seldom probes, whereas the 34% CNVcaller calls and 28%CNVnator calls were overlap with SDs.

Two extra genome-wide evaluations can provide supplemental proofs. The Mendelian inconsistence of 10 Dutch family was added in Supplementary Figure 5, which can test both unique and SD regions. The inconsistence rate of CNVcaller, CNVnator and Genome STRiP was 1.5%, 4.4%, and 0.4%. This accuracy ranking was consistent with the genotyping discordance compared with the aCGH database, which were 2.6%, 5.5% and 2.2% for CNVcaller, CNVnator and Genome STRiP respectively.

- Some details of the method are vaguely specified and some Figures lack clarity and units.

Page 6, line 129: "... if the median RD of the homogametic sex chromosomes is about half of the median RD of autosome..."

Modified as follows:

“Most mammalian and avian genomes show XX/XY-type or ZZ/ZW-type sex-determining system. Their homogametic sex chromosomes (X or Z) constitute 5%-10% of the total genome, and show half RD of the autosomes in XY or ZW individuals. Therefore, insensitive corrections are needed. The name of the homogametic sex chromosome is required as a parameter. If the median RD of this chromosome is <0.6X of the median RD of the autosome, this individual is determined as the XY or ZW type, and the RDs of this chromosome are doubled before normalization. Otherwise, this individual is determined as XX or ZZ type, and no sex correction will be done.”

Page 8, line 154: "... and the distance between them is less than a certain percent of their own length."

Modified as follows: "The distance between the two initial calls is less than 20% of their combined length."

Page 5, line 91: "The reference genome is segmented into overlapping sliding windows." What window size and overlap was used for high-coverage genomes?

The following description was added in methods.

"The window size is an important parameter for the RD methods. CNVcaller uses half of the window size as step size. The optimal window size is 800 bp (with a 400 bp overlap) for 5-10X coverage human and livestock sequencing data (Supplementary Figure 1). The recommended scales roughly inversely coverage, resulting in 400 bp windows for 20X coverage and 200 bp windows for 50X coverage."

Page 5, line 95: "The raw RD signal is calculated for each window as the number of placed reads with centers within window boundaries." Does this imply that for paired-end data both reads are counted?

Yes, considering the uncontrollable effect of gap ratios from different genome assemblies, all of the end reads located in the window are independently added to the RD of this window, regardless of the read is from single end mapping or paired mapping.

Page 8, line 154: "Then the two adjacent initial calls are further merged if their copy numbers are highly correlated". What threshold was used?

Modified as follows: "CNVRs can be separated by gaps or poorly assembled regions, therefore, the adjacent initial calls are merged if their RDs are highly correlated. The default parameters are: the distance between the two initial calls is less than 20% of their combined length, and the Person's correlation index of the two CNVRs is significant at P = 0.01 level."

Figure 3A: CNVcaller 13.7. What is the unit? Are these 13,700 CNVs?

The unit of this figure was Mbp, because the intersection of the three methods was hard to define in number with different boundaries, so they are evaluated in length. CNVcaller covered 40% of the CNVRs detected by CNVnator, 45% of Genome STRiP and 65% of their intersection, in length.

Minor:

- I could not find a reference to the 232 goat sequencing data? Is this data publicly available?

Among the 232 goat whole genome sequencing data, 103 were acquired from NCBI (reference paper see below), and the accession numbers are provided in Supplementary Table 1. The remaining 129 samples without accession number were generated by ourselves.

Badr Benjelloun FJA, Streeter I, Boyer F, Coissac E, Stucki S, et al. (2015) Characterizing neutral genomic diversity and selection signatures in indigenous populations of Moroccan goats (Capra hircus) using WGS data. Frontiers in genetics 6.

Dong Y, Zhang X, Xie M, Arefnezhad B, Wang Z, et al. (2015) Reference genome of wild goat (capra aegagrus) and sequencing of goat breeds provide insight into genic basis of goat domestication. BMC genomics 16: 431.

Dong Y, Xie M, Jiang Y, Xiao N, Du X, et al. (2013) Sequencing and automated whole-genome optical mapping of the genome of a domestic goat (Capra hircus). Nature biotechnology 31: 135-141.

Bickhart DM, Rosen BD, Koren S, Sayre BL, Hastie AR, et al. (2017) Single-molecule sequencing and chromatin conformation capture enable de novo reference assembly of the domestic goat genome. Nature Genetics 49: 643-650.

Wang XL, Liu J, Niu YY, Li Y, Zhou SW, et al. Low incidence of SNVs and indels in trio genomes of Cas9-mediated multiplex edited sheep. BMC Genomics. Under review.

- The first Results section "Overview of CNVcaller algorithm" seems better suited for the Methods part.

Modified as suggested.

- Is the Mendelian consistency higher for the high-coverage trio: NA12878, her father (NA12891) and her mother (NA12892)?

Yes. Upon the high coverage data of all three members of the trio (NA12891, NA12892 and NA12878 are all 50 X), the inconsistent rate was 2.4%. Upon the high coverage parents (50 X NA12891 and NA12892) and low coverage child (5.3 X NA12878), the inconsistent rate was 6.1%. So, the increased sequencing depth can help to reduce the number of false positives.

- I believe the claim that read-pair/split-read algorithms are less powerful on draft assemblies of non-model organisms compared to read-depth methods is potentially true but the Manuscript lacks a proof for this or a citation that supports this claim.

Thank you for your agreement. This problem was found in our previous reference genome assembly projects for both sheep and goats. However, we did not report this result in the section of CNV/SD detection. So, we removed this comment from this manuscript. However, we found the following citations may help to support this claim:

All of these algorithms including read-pair/split-read (RP/SR) and read-depth rely on mapping sequencing reads back to reference genome. However, for many non-model organisms, the reference genome likely contains many errors, which mainly arose from repeat collapse and expansion; and rearrangement and inversion [1] . These mis-assembly sequences and the repetitive regions of the genome can result in many pair-end reads have multiple good mappings, thus it is difficult for RP/SR to uniquely identify the true CNVs boundaries[2]. However, based on read depth by considering all possible map locations for a read can address this problem[3].

1. Phillippy AM, Schatz MC, Pop M (2008) Genome assembly forensics: finding the elusive mis-assembly. Genome biology 9: R55.

2. He D, Hormozdiari F, Furlotte N, Eskin E (2011) Efficient algorithms for tandem copy number variation reconstruction in repeat-rich regions. Bioinformatics 27: 1513-1520.

3. Alkan C, Kidd JM, Marques-Bonet T, Aksay G, Antonacci F, et al. (2009) Personalized copy number and segmental duplication maps using next-generation sequencing. Nature genetics 41: 1061-1067.

- It is not clear from the Manuscript if CNVcaller reports copy-number likelihoods

based on the Gaussian mixture model. Please clarify.

Thank you for you suggest. CNVcaller reports the silhouette coefficients of the copy numbers instead of the Gaussian mixture model likelihoods as quality control. Because we found silhouette coefficients has greater correlation with IRS test result than likelihoods.

- Figure 5A: Why is the absolute copy-number correction different for Human and Sheep?

We are sorry for not clearly interpreting the high-proportioned mis-assembled segmental duplications in non-human assemblies. This part was modified as follows:

"Previous studies showed high proportion of SDs in animal genomes are mis-assembled single copy regions. So, we validated the copy numbers on human (hg19) and sheep (OAR v3.1) reference genome assembly by the sequencing copy number of a human (NA12878) and a Tan sheep sample (Figure 6A). If the SDs were correctly assembled, the sequencing diploid copy number should be two times of the copy number of SDs. For example, the average sequencing copy number of the two-copy SDs was four in NA12878. However, the corresponding sequencing copy number of sheep was only 2.4. These results indicated most two-copy SDs of hg19 were truly duplicated in NA12878 while approximately 80% of the two-copy SDs in OAR v3.1 were unique regions in the Tan sheep sample. So, the SDs in sheep genome were called "putative SDs" before validation."

- There is quite a few typing and grammatical errors. For instance:

*Figure 2B: Max mamory
*Supplementary Table 3: Memery
*Page 3, line 53: ...the number of reads aligned to of a particular region.
*Page 8, line 160: This model presets the average copy number of homozygous deletion, heterozygous deletion, normal, heterozygous deletion (duplication!), homozygous deletion (duplication!) at zero to four respectively.

We are sorry for these mistakes. We have proofread the revised manuscript and used professional English language editing to minimize the grammatical errors.

Checklist of the updated tables and figures

Current version	Last version
Fig. 3	Fig. 5
Fig. 4A-C	Table 1 and newly added
Fig. 4D	Fig. 3B
Fig. 5	Fig. 4
Fig. 6A-C	Table 1 and newly added
Fig. 6D	Fig. 3A
Supplementary Fig. 1	Newly added
Supplementary Fig. 2	Supplementary Fig. 1
Supplementary Fig. 3	Newly added
Supplementary Fig. 4	Supplementary Fig. 2
Supplementary Fig. 5	Table 1 and newly added
Supplementary Table 4	Newly added
Supplementary Table 5	Newly added
