# Supplementary material for: CNVcaller: highly efficient and widely applicable software for detecting copy number variations in large populations
Source: Gigascience. 2017 Dec 4;6(12):1–12. doi: 10.1093/gigascience/gix115 (PMC5751039; doi:10.1093/gigascience/gix115)
Supplement: GIGA-D-17-00119_Revision_2.pdf [file gix115_giga-d-17-00119_revision_2.pdf]

GigaScience

CNVcaller: Highly Efficient and Widely Applicable Software for Detecting Copy Number Variations in Large Populations

--Manuscript Draft--

**Manuscript Number:**	GIGA-D-17-00119R2
**Full Title:**	CNVcaller: Highly Efficient and Widely Applicable Software for Detecting Copy Number Variations in Large Populations
**Article Type:**	Technical Note
**Funding Information:**	National Natural Science Foundation of China (31572381) — Prof. Yu Jiang<br>National Thousand Youth Talents Plan — Prof. Yu Jiang
**Abstract:**	Background: The increasing amount of sequencing data available for a wide variety of species can be theoretically used for detecting copy number variations (CNVs) at the population level. However, the growing sample sizes and the divergent complexity of non-human genomes challenge the efficiency and robustness of current human-oriented CNV detection methods.<br><br>Results: Here, we present CNVcaller, a read-depth method for discovering CNVs in population sequencing data. The computational speed of CNVcaller was 1-2 orders of magnitude faster than CNVnator and Genome STRiP for complex genomes with thousands of unmapped scaffolds. CNV detection of 232 goats required only 1.4 days on a single compute node. Additionally, the Mendelian consistency of sheep trios indicated that CNVcaller mitigated the influence of high proportions of gaps and misassembled duplications in the non-human reference genome assembly. Furthermore, multiple evaluations using real sheep and human data indicated that CNVcaller achieved the best accuracy and sensitivity for detecting duplications.<br><br>Conclusion: The fast, generalized detection algorithms included in CNVcaller overcome prior computational barriers for detecting CNVs in large-scale sequencing data with complex genomic structures. Therefore, CNVcaller promotes population genetic analyses of functional CNVs in more species.
**Corresponding Author:**	Yu Jiang, Ph.D<br>Northwest Agriculture and Forestry University<br>Yangling, Shaanxi CHINA
**Corresponding Author Secondary Information:**	
**Corresponding Author's Institution:**	Northwest Agriculture and Forestry University
**Corresponding Author's Secondary Institution:**	
**First Author:**	Xihong Wang
**First Author Secondary Information:**	
**Order of Authors:**	Xihong Wang
	Zhuqing Zheng
	Yudong Cai
	Ting Chen
	Chao Li
	Weiwei Fu
	Yu Jiang
**Order of Authors Secondary Information:**	

Powered by Editorial Manager® and ProduXion Manager® from Aries Systems Corporation

Response to Reviewers:	Dear Dr. Edmunds,

Dear Dr. Edmunds,

Thank you very much for handling our manuscript "CNVcaller: Highly Efficient and Widely Applicable Software for Detecting Copy Number Variations in Large Populations " (GIGA-D-17-00119). We appreciate all the comments from the reviewers, which helped us improve our manuscript. We have now revised the manuscript according to the reviewers' comments and your instructions.
We addressed the comments and questions of the reviewers as explained below; the reviewers' text has been included, and our responses are in coloured italics. The revised text is indicated by quotation marks. Because several new figures have been added, we have attached a list of the current figures and tables corresponding to those in the last version so that the changes can be easily tracked.

According to the suggestions of the reviewers, we have modified the manuscript as follows:

1.The newly released version of CNVcaller updated the genotyping method. A python package, scikit-learn v0.19.0, was used to decompose the reported copy numbers into several Gaussian distributions. Therefore, the accuracy of CNVcaller in the new version was increased.

2.The reviewers requested that we evaluate the effects of the length and allele frequency of the discovered CNVRs. Therefore, two sections have been added to the results analysing the number and FDR of the CNVRs detected by the three methods against the length and allele frequency. One section (including Figure 4) was based on the sheep data, the other section (including Figure 6) was based on the human data.

3.To answer the reviewer's question about the difference between the FDRs in deletions and duplications, their FDRs were evaluated separately in the results section.

4.The high proportion of misassembled segmental duplications in non-human assemblies may have led to misunderstanding on the reviewer's part. This section has been extensively redrafted with analyses of both real and simulated data.

5.The previous discussion section has been merged with the results section to reduce the length of the manuscript. The first part of the previous results section has been moved to the methods section, as suggested by the reviewer.

6.The language has been professionally edited by an English-language editing service, American Journal Experts (AJE).

7.We have registered the software in the SciCrunch.org database. The RRID SRC_015752 was added to the 'Availability and requirements' sections.

Thank you again for all of your assistance.

Sincerely yours,
Yu Jiang and co-authors

****
REVIEWS
****
Reviewer #1:

- The authors developed a new CNV caller pipeline which they called CNVcaller geared towards improved speed compared to existing CNV callers and improved accuracy for high complexity genomes. I commend the authors on their efforts to introduce improved algorithms and pipelines for an inherently difficult procedure, namely CNV calling. My comments are mostly suggestions for improvement as follows. Note, comments of the form (4:5 for example represent page 4, line 5).

Thank you for your positive comments and encouragement. We have substantially revised the manuscript upon your suggestions.

- There are several grammatical errors which make the paper somewhat confusing. I would strongly recommend further extensive English editing.

We apologize for these mistakes. The manuscript has been professionally edited by an English-language editing service, American Journal Experts (AJE) .

- My main criticism of the analysis is one that I have seen repeatedly of most other CNV calling publications, and that is there is no sensitivity analysis.

We are sorry for the ambiguity of the sensitivity tests, which were included in the previous Table 1. In the revised manuscript, Figure 6C has been added to describe the sensitivity. In general, CNVcaller demonstrated 57%-67% sensitivity for duplications and 66%-68% for deletions in human data. The sensitivity in sheep was ~73% by indirectly evaluation. The detailed descriptions are as follows:

Human: “The sensitivity was estimated as the proportion of the high-confidence CNVR database that overlapped with the predicted CNVRs. Two previously published high-confidence databases that include our test samples are the aCGH-based CNVR database [1] and the 1000GP CNVR map [2]. For the aCGH database, CNVcaller demonstrated the highest sensitivity (57%) in duplications, whereas Genome STRiP achieved the highest sensitivity (74%) in deletions (Figure 6C). Both Genome STRiP and CNVnator were the core contributors to the 1000GP CNV maps; However, the sensitivity of CNVcaller was 68% and 67% for deletions and duplications according to this database, only 4%-10% lower than Genome STRiP and CNVnator.”

Sheep: “The sensitivity of sheep CNVRs was estimated indirectly due to the lack of a validated database. Based on our integrated analysis (see methods), there were 138 sheep X chromosome-origin scaffolds, which were not anchored onto chromosomes of OAR v3.1. Therefore, all of these scaffolds should be detected as CNVs because the rams had half the copy numbers of the ewes. As a result, CNVcaller detected 101 of these 138 X-origin scaffolds, with a sensitivity of 73%. In contrast, CNVnator and Genome STRiP did not report these unmapped CNVRs.”

- The authors here also suggest various parameters throughout their paper for performing CNV calling, but there is no analysis of how the results change if these parameters are adjusted, i.e. no analysis of how robust your algorithm is to changes in the parameters.

Thank you for your suggestion. Two important parameters of CNVcaller were window size and minimum report allele frequency. The FDRs against the window size and alternative allele frequency have been added to Supplementary Figure 1 and Figure 6B. In general, with the increasing of window size and allele frequency, the accuracy raised while the sensitivity decreased.

- As another example, Hong et al 27503473 has demonstrated that the biggest variability in calling CNVs is in terms of the CNV size. I suspect that the same can be said of CNVcaller. Please comment on what sizes of CNVs does CNV caller do well or poorly on.

Thank you for your suggestion. Figure 4 and Figure 6 have been added, which evaluate the effects of length and frequency in sheep and human data. The detailed comparisons in the manuscript are as follows:

Sheep: “The accuracy was evaluated by the Mendelian inconsistency of all the CNVRs on autosomes against the length and alternative allele frequency (Figure 4). CNVcaller achieved higher accuracy than Genome STRiP in both deletion (1% vs 2%) and duplication (4% vs 7%) (Figure 4A). Whereas Genome STRiP had greater capability to detected short (<2.5 kb) deletions (Figure 4B), indicating the RP methods integrated in Genome STRiP performed well on small deletions. Concerning the alternative allele frequency, both methods showed an increased FDR in rare duplications (Figure 4C). However, CNVcaller is primarily used to detect CNVRs related to economic traits in

livestock and crops. In these studies, the target CNVRs usually have a high frequency after long-duration breeding selection."

Human: "CNVcaller demonstrated the highest overall accuracy for detecting duplications and performed consistently across the length and frequency categories, whereas Genome STRiP and CNVnator had high FDRs on the short or singleton duplications (Figure 6A, B). Genome STRiP showed the greatest ability to detect deletions, indicating the advantage of combining RD and RP methods for deletion detection. The genotyping accuracy of the human dataset was further benchmarked against the high-confidence aCGH array-based database. The discordance rates of CNVcaller, CNVnator and Genome STRiP were 2.6%, 5.5% and 2.2%, respectively. This genotyping accuracy ranking was same with the Mendelian inconsistency of the 10 Dutch trios (Supplementary Figure 5)."

- 2:32 "the prevalent.." is a gross exaggeration. I think you mean "a prevalent".

This has been corrected as suggested.

- 2:35 I don't think you mean geometric. I did not comment on other grammatical/English errors as there were too many to list individually. I would highly recommend getting help with the English in this paper.

We apologize for these mistakes. The manuscript has been professionally edited by an English-language editing service, American Journal Experts (AJE).

- 3:53 "RD" is not defined.

We apologize for the missing definition. The following description has been added to the introduction:
"read depth (RD) refers to the depth of coverage in a genomic region that can be calculated from the number of aligned reads [14], a CNV region should have a higher or lower RD than expected [22-24]."

- 6:120 Give a brief description of how CNVnator handles GC bias. Also why 40% for the GC bias? Shouldn't this parameter be dependent on the organism of interest?

We apologize for not clearly describing the procedure. In general, the mean RD of windows with 40% percent GC is used as only a temporary standard in the GC correction step. It will be lost in the following normalization step, in which the GC-corrected RDs of each window are divided by the global median RDs. Because the denominator is calculated from the RDs already corrected by the 40% GC windows, this parameter will be lost and is not necessarily dependent on the organism of interest.

The GC corrected RD for a window is calculated by CNVnator as follows: the raw RD times the global average RD and divided by the average RD with the same GC content as in this window. Because the global average RD is calculated before the GC correction, no temporary parameter is used. The equation is showed in Personal Cover.

- The commentary on certain genomes not being as complete as others is important. I suspect though that if a large percentage of the samples show a CNV in a genome that is newer or not as complete, then this observation may be more likely indicative of a problem with the reference. Can you comment?

If the detected CNVR has variation in a population, which means the read depths can be clustered into two or more normal distributions, this CNVR is probably true even with high frequency. In contrast, if all of the individuals show the same abnormal read depth, this suggests that the reference individual is different from the sample population or some assembly problems exist.

- 7:145 I am not convinced Pearson's correlation is appropriate. Your data is likely to have outliers and non-normal data. A non-parametric test of correlation like Spearman's correlation (Kendall-Tau is likely too computational intensive), or

performing correlation after 5 or 10% trimming may be more appropriate.

We tried replacing Pearson's correlation with Spearman's correlation in the 30 BAM files from the 1000 Genome Projects data. However, the FDR doubled after the replacement, while the length of each call was reduced by half. A possible reason is that Spearman's correlation is calculated by ranking instead of the numerical value of copy numbers across samples. Therefore, the divergent copy numbers of individuals with deletions or duplications contributed no more than the subtle random mistakes of normal copy individuals, especially in the low-frequency CNVRs.

Trimming is also not recommended for a similar reason. In the low-frequency CNVRs, individuals with an abnormal copy number will be trimmed as outliers.

- cn.MOPS (Klambauer et al, PMID: 22302147) uses a mixture of Poissons as opposed to Gaussian Mixture Models for CNV detection. I suspect the mixture of Poissions will be superior to Gaussian Mixture Models when the read depths are low, and Gausssian mixtures may be more appropriate when read depths are high. How difficult is it to replace the Gaussian mixtures with Poisson mixtures and compare the performance? I feel that this analysis would be informative and potentially improve your algorithm.

Thank you for your suggestion. However, it is not easy to replace the distribution because the RDs after GC correction and normalization are not integers; thus, they cannot be directly treated as Poisson distributions. Additionally, we totally agree with your comment about the Poisson distribution will be superior for low read depths with high STDEV. However, the currently common sequencing depth are about 5-10X. Under this sequencing depth and a proper window size, the STDEV/mean RD was only 0.2-0.3, which essentially not fit the Poisson distribution. In addition, we used the RDs of 232 goats with ~10X coverage to test the fitness of Gaussian distribution using the omnibus test (scipy 0.19.0). As a result, 88% of windows accepted the null hypothesis at the P = 0.01 level. Therefore, we believe the Gaussian Mixture Model is acceptable for the current data.

- The term "CNVR" is critical for understanding the algorithm, and requires more explanation of the term.

We apologize for missing this important concept. The following explanation has been added to the introduction:
“To study the polymorphism among individuals, the overlapping CNVs need to be merged into unified regions, namely CNV regions (CNVRs)”

- It would be helpful to include some further discussion on where you see that CNVcaller works better or worse than existing CNV calling software.

Thank you for your suggestion. Figure 2 shows that the speed of CNVcaller was one to two orders of magnitude higher than the other methods. Newly added Figure 4 and Figure 6 evaluated the effects of length and frequency in sheep and human data. In general, the performance of CNVcaller was better for all sizes of duplications but was poor for deletions <2.5 kb.

- 9:180. The "arbitrary standards" require a citation.

Two citations have been added as follows:

1.Chain FJ, Feulner PG, Panchal M, Eizaguirre C, Samonte IE, Kalbe M, et al. Extensive copy-number variation of young genes across stickleback populations. PLoS genetics. 2014;10 12:e1004830.

2.Abyzov A, Urban AE, Snyder M and Gerstein M. CNVnator: an approach to discover, genotype, and characterize typical and atypical CNVs from family and population genome sequencing. Genome research. 2011;21 6:974-84.

- Minor comment: Since speed seems to be a major selling point of the software, more details about running the software on a compute cluster or running algorithms in parallel in the documentation would be helpful.

Powered by Editorial Manager® and ProduXion Manager® from Aries Systems Corporation

A new section, “Parallel submission of individual RD processing,” has been added to the methods with the principle and commands as follows:
“Parallel processing of individual RDs. The CNVcaller processes the BAM file of each individual separately in the first step, and therefore, parallel computations can be performed to reduce the total running time. All BAM files are equally distributed into N groups, and each group contains M files. The max N is the total available processing cores, and M is the total number of BAM files/N. For example, the 232 goat BAM files were processed on a node with 32 processing cores and 124 GB of RAM. We distributed the 232 files into 20 groups, and each group contained 12 BAM files. The shell command for one group is as follows:

```
#!/bin/sh
for i in {1..M}
 do bash Individual.Process.sh -b $i.bam -h $i -d dup -s sex_chromosome
done
```

After corrections and normalization, the comparable RDs of each sample are aggregated into an ~100 MB intermediate file and output, thus preventing repeated calculations for the same individual in different populations.”

****
Reviewer #2:

The proposed method "CNVcaller" enables the efficient discovery and genotyping of CNVs in large populations. One of the main benefits of the method is that it can handle draft genome assemblies with thousands of scaffolds. The computational benchmarks proof that the method is fast and memory efficient but the evaluation of the accuracy of the method is less convincing. Some details of the method remain vague and hinder an objective evaluation. Detailed comments of how to improve the manuscript are below:

Thank you for your affirmation. We are sorry for the ambiguity of the accuracy test and have substantially revised the manuscript according to your suggestions. In the revised manuscript, the performance evaluation in the previous Table 1 is described in more detail in Figure 4 and Figure 6.

Comment 1 - The primary application of CNVcaller is the detection of CNVs in large populations. Population variant call sets are dominated by rare variants of rather small size. For instance, less than 20% of the 1000 Genomes structural variants have a population allele frequency >5% and almost 50% of the SVs are <2kbp in size despite the rather low coverage (~7x). CNVcaller is currently restricted to large CNVs (>2kbp) and common variants (>5% allele frequency), which is a major limitation for population genomic studies.

We apologise for the ambiguous. Actually, the user can retain all the windows with at least one individual that shows heterozygous deletion or duplication. However, we recommend removing low-frequency windows in large populations with low sequencing coverage because of increased random mistakes. In the revised version, Figure 6 was added to evaluate the effects of length and frequency by IRS test. We found Genome STRiP showed the greatest ability to detect short and rare deletions, indicating the advantage of combining RD and RP methods for deletion detection. However, short and rare duplications still had extremely high FDR. The shortest duplications reported by CNVnator and Genome STRiP were 2.8 kb and 2.5 kb, and the IRS FDRs of 2.5-5 kb calls were 29% and 88%, respectively. The FDRs of singletons were 35% and 69% for CNVnator and Genome STRiP, respectively. The main improvement of CNVcaller is the accuracy of duplications. The FDR of 2.5 kb – 5 kb duplications was reduced to 19%, and the FDR of singleton duplications were reduced to 9%. However, the FDRs were still higher than those of the longer and higher-frequency calls. So, these calls were removed from the previous manuscript. These uncertain calls were also removed by the phase 3 extended SV release of 1000GP. After extra quality controls, the number of duplications in the released database is only 1/7 the number of deletions, and the median size is 36 kb, which is 17 times longer than deletions. Therefore, improving the accuracy of duplications on this foundation is meaningful for enriching the CNV database.

Powered by Editorial Manager® and ProduXion Manager® from Aries Systems Corporation

Additionally, the current main use of CNVcaller is the detection of CNVRs related to economic traits in livestock and crops. In these populations, the target CNVRs usually have a medium or high frequency after long-duration artificial selection. We believe that the high-confidence medium to high frequency reported by CNVcaller can contribute to functional and breeding studies of animals and plants.

The sensitivity increase of CNVcaller for the subset of common and large CNVs seems to be driven by an increased number of detected CNVs in SD regions (Figure 5C). SNP arrays have a low SNP density in SD regions and in the present Manuscript array SNP probes in SD regions have been removed entirely. The reported IRS FDR is therefore heavily biased against CNVs in SD regions and it thus seems mandatory to me to proof that this sensitivity increase for SD-associated CNVs is not leading to an inflated FDR.

Thank you for your suggestions. Figure 5C (new Figure 3C) has been updated to show both the number and the Mendelian inconsistency of the detected CNVs in SDs. The Mendelian inconsistency rate of the calls in SD regions made by CNVcaller was approximately 3%, no higher the other methods. The copy numbers of unique and SDs were also indirectly validated by the X-origin scaffolds of a 133-sheep population. All of these scaffolds should be detected as CNVs because the rams had half the copy numbers of the ewes. As a result, CNVcaller detected 101 of these 138 X-origin scaffolds. In contrast, CNVnator and Genome STRiP did not report these regions.

The Manuscript lacks a Figure that shows the size and allele frequency distribution of the discovered CNVs in comparison to Genome STRiP and CNVnator. An estimate of breakpoint accuracy of CNVcaller would also be valuable.

Thank you for your suggestion. Figure 4 and Figure 6 have been added, which evaluate the effects of length and frequency in sheep and human data. The detailed comparisons in the manuscript are as follows:

Sheep: “The accuracy was evaluated by the Mendelian inconsistency of all the CNVRs on autosomes against the length and alternative allele frequency (Figure 4). CNVcaller achieved higher accuracy than Genome STRiP in both deletion (1% vs 2%) and duplication (4% vs 7%) (Figure 4A). Whereas Genome STRiP had greater capability to detected short (<2.5 kb) deletions (Figure 4B), indicating the RP methods integrated in Genome STRiP performed well on small deletions. Concerning the alternative allele frequency, both methods showed an increased FDR in rare duplications (Figure 4C). However, CNVcaller is primarily used to detect CNVRs related to economic traits in livestock and crops. In these studies, the target CNVRs usually have a high frequency after long-duration breeding selection.”

Human: “CNVcaller demonstrated the highest overall accuracy for detecting duplications and performed consistently across the length and frequency categories, whereas Genome STRiP and CNVnator had high FDRs on the short or singleton duplications (Figure 6A, B). Genome STRiP showed the greatest ability to detect deletions, indicating the advantage of combining RD and RP methods for deletion detection. The genotyping accuracy of the human dataset was further benchmarked against the high-confidence aCGH array-based database. The discordance rates of CNVcaller, CNVnator and Genome STRiP were 2.6%, 5.5% and 2.2%, respectively. This genotyping accuracy ranking was the same with the Mendelian consistency of the 10 Dutch trios (Supplementary Figure 5).”

Thank you for your reminding of the breakpoint issue. However, unlike the PR/SP algorithm, RD can not detect breakpoints in the at base pair resolution or less than the window step size resolution. Integrating RD and RP methods can improve the breakpoint accuracy in human genome. However, precise breakpoint is more difficult to achieve in the poorly assembled genomes. Additionally, the breakpoint issue did not affect the genotyping accuracy which is the direct input of GWAS. The genotyping FDR of CNVcaller, CNVnator and Genome STRiP were 2.6%, 5.5% and 2.2%, respectively.

The Manuscript mentions mrsFAST for absolute copy number validation. I could not find any formal comparison of predicted copy-number by mrsFAST and CNVcaller but maybe I missed this?

Supplementary Figure 2 (previous Supplementary Figure 1) shows that the copy numbers calculated using mrsFAST and CNVcaller were similar. However, mrsFAST needed to realign all the multi-hit reads in BWA alignments, leading to significantly increased computational time. For example, mrsFAST required 10 hours for a 3G genome with 10X sequencing data, whereas CNVcaller needed only 4 minutes.

- Please add to Table 1 the number of CNV sites that could be assessed by the IRS method and what proportion of each call set could be evaluated using IRS. I also believe the IRS method reports p-values separately for deletions, duplications and multi-allelic CNVs. Was there any difference among these for CNVcaller?

Detailed information on 1000GP calls, including the required information, has been added to Supplementary Table 5. Overall, 28%, 30% and 60% of the CNVRs of CNVcaller, CNVnator and Genome STRiP covered at least one probe of the Affymetrix SNP 6.0 array and therefore could be assessed using the IRS test. One main reason for the divergent testable proportions was that only 4% of Genome STRiP calls overlapped with SDs, which have infrequent probes, whereas 34% of the CNVcaller calls and 28% of the CNVnator calls overlapped with SDs.

Two extra genome-wide evaluations can provide supplemental evidence. The Mendelian inconsistency of 10 Dutch families was added to Supplementary Figure 5, which was based on tests of both unique and SD regions. The inconsistency rates of CNVcaller, CNVnator and Genome STRiP were 1.5%, 4.4%, and 0.4%, respectively. This accuracy ranking was consistent with the genotyping discordance values compared with the aCGH database, which were 2.6%, 5.5% and 2.2% for CNVcaller, CNVnator and Genome STRiP, respectively.

To analysis the difference between deletions and duplications, all FDRs were evaluated separately in the revised manuscript. We found the duplications had much higher FDRs than the deletions, especially for the short and rare CNVs.

- Some details of the method are vaguely specified and some Figures lack clarity and units.
Page 6, line 129: "... if the median RD of the homogametic sex chromosomes is about half of the median RD of autosome..."

This section has been expanded in the newly added subsection “RD corrections for sex chromosomes” as follows:

“RD corrections for sex chromosomes. Most mammalian and avian genomes show an XX/XY-type or ZZ/ZW-type sex-determining system. Their homogametic sex chromosomes (X or Z) constitute 5%-10% of the total genome and show half the RD of the autosomes in XY or ZW individuals. Therefore, intensive correction for X and Z chromosomes is needed. The RD of the X or Z chromosome (the particular name provided by the user) is used to determine the sex of a particular individual. If the median RD of this chromosome is <0.6X the median RD of the autosome, the individual is considered an XY or ZW type, and the RDs of this chromosome are doubled before normalization. Otherwise, nothing is performed for individuals determined to be XX or ZZ type.”

Page 8, line 154: "... and the distance between them is less than a certain percent of their own length."

This text has been modified as follows: “As CNVRs can be separated by gaps or poorly assembled regions, the adjacent initial calls are merged if their RDs are highly correlated. The default parameters are as follows: the distance between the two initial calls is less than 20% of their combined length, and the Pearson’s correlation index of the two CNVRs is significant at the P = 0.01 level.”

Page 5, line 91: "The reference genome is segmented into overlapping sliding windows." What window size and overlap was used for high-coverage genomes?

The following description has been added to the methods.

“The window size is an important parameter for RD methods. CNVcaller uses half of the window size as the step size. The optimal window size is 800 bp (with a 400 bp overlap) for 5-10X coverage human and livestock sequencing data (Supplementary Figure 1). The recommended window sizes are inversely related with coverage, and thus, ~400 bp windows correspond to 20X coverage, and ~200 bp windows correspond to 50X coverage.”

Page 5, line 95: "The raw RD signal is calculated for each window as the number of placed reads with centers within window boundaries." Does this imply that for paired-end data both reads are counted?

We apologise for the ambiguous description. The following description has been added: “Considering the uncontrollable effect of gap ratios from different genome assemblies, all of the end reads located in the window are independently added to the RD of this window, regardless of whether the read is from single-end mapping or paired mapping.”

Page 8, line 154: "Then the two adjacent initial calls are further merged if their copy numbers are highly correlated". What threshold was used?

This text has been modified as follows: “As CNVRs can be separated by gaps or poorly assembled regions, the adjacent initial calls are merged if their RDs are highly correlated. The default parameters are as follows: the distance between the two initial calls is less than 20% of their combined length, and the Pearson’s correlation index of the two CNVRs is significant at the P = 0.01 level.”

Figure 3A: CNVcaller 13.7. What is the unit? Are these 13,700 CNVs?

The unit in this figure is Mb. Because the intersection of the three methods with different boundaries was difficult to define in numbers, they were evaluated in terms of length. CNVcaller covered 40% of the CNVRs detected by CNVnator, 45% of the CNVRs detected by Genome STRiP and 65% of their intersecting CNVRs, in terms of length.

Minor:
- I could not find a reference to the 232 goat sequencing data? Is this data publicly available?

Among the 232 goat whole-genome sequencing data files, 103 files were acquired from NCBI, the accession numbers are provided in Supplementary Table 1. The remaining 129 samples without accession numbers were generated by ourselves, and will be published soon. The reference and unpublished paper are as follows:

1.Badr Benjelloun FJA, Streeter I, Boyer F, Coissac E, Stucki S, et al. (2015) Characterizing neutral genomic diversity and selection signatures in indigenous populations of Moroccan goats (Capra hircus) using WGS data. Frontiers in genetics 6.

2.Dong Y, Zhang X, Xie M, Arefnezhad B, Wang Z, et al. (2015) Reference genome of wild goat (capra aegagrus) and sequencing of goat breeds provide insight into genic basis of goat domestication. BMC genomics 16: 431.

3.Dong Y, Xie M, Jiang Y, Xiao N, Du X, et al. (2013) Sequencing and automated whole-genome optical mapping of the genome of a domestic goat (Capra hircus). Nature biotechnology 31: 135-141.

4.Bickhart DM, Rosen BD, Koren S, Sayre BL, Hastie AR, et al. (2017) Single-molecule sequencing and chromatin conformation capture enable de novo reference assembly of the domestic goat genome. Nature Genetics 49: 643-650.

5.Wang XL, Liu J, Niu YY, Li Y, Zhou SW, et al. Low incidence of SNVs and indels in trio genomes of Cas9-mediated multiplex edited sheep. BMC Genomics. Under review.

6.Zheng ZQ, Li M, Liu J, Wang XL, Pan XY, et al. The early domestication process inferred from genome analysis of worldwide goats. In preparation.

- The first Results section "Overview of CNVcaller algorithm" seems better suited for the Methods part.

This has been modified as suggested.

- Is the Mendelian consistency higher for the high-coverage trio: NA12878, her father (NA12891) and her mother (NA12892)?

Yes. In the high-coverage data of all three members of the trio (NA12891, NA12892 and NA12878 were all 50X), the inconsistency rate was 2.4%. In the high-coverage data of the parents (50X for NA12891 and NA12892) and the low-coverage data of the child (5.3X for NA12878), the inconsistency rate was 6.1%. Thus, increased sequencing depth can help to reduce the number of false positives.

- I believe the claim that read-pair/split-read algorithms are less powerful on draft assemblies of non-model organisms compared to read-depth methods is potentially true but the Manuscript lacks a proof for this or a citation that supports this claim.

Thank you for your agreement. This problem was found in our previous reference genome assembly projects for both sheep and goats. However, we did not report this result in the section on CNV/SD detection. The review listed below has some comments about this claim, however, without direct supporting data. Therefore, we have removed this comment from this manuscript.

Bickhart DM and Liu GE. The challenges and importance of structural variation detection in livestock. Frontiers in genetics. 2014;5.

“While RP methods should provide a suitable means for detecting such events in theory, two major problems currently challenge the accuracy of this method:

(1) alignment errors resulting from the mapping of read pairs to repetitive regions of the genome…… The first problem (1) is unfortunately dependent on the reference genome assembly for the species, and is unlikely to be resolved until better reference assemblies are created for livestock.”

- It is not clear from the Manuscript if CNVcaller reports copy-number likelihoods based on the Gaussian mixture model. Please clarify.

Thank you for your suggestion. CNVcaller reports the silhouette coefficients of the copy numbers instead of the Gaussian mixture model likelihood as quality control because we found that silhouette coefficients had a greater correlation with the IRS test results than likelihood.

- Figure 5A: Why is the absolute copy-number correction different for Human and Sheep?

We are sorry for not clearly interpreting the high proportion of misassembled segmental duplications in non-human assemblies. This part of the manuscript has been modified as follows:

“Previous studies have shown that a high proportion of SDs in animal genomes are misassembled single-copy regions [27, 29]. Therefore, we detected the ratios of false SDs on the human (hg19) and sheep (OAR v3.1) reference genome assemblies by the sequencing copy number of a human (NA12878) and a Tan sheep sample (Figure 3A). If the SDs were correctly assembled, the sequencing diploid copy number should be twice the copy number of SDs. For example, the average sequencing copy number of the two-copy SDs was four in NA12878. However, the corresponding sequencing copy number in sheep was only 2.4. These results indicated that most two-copy SDs of hg19 were truly duplicated in NA12878, while approximately 80% of the two-copy SDs in OAR v3.1 were single-copy regions in the Tan sheep sample. Thus, the SDs in the sheep genome were called “putative SDs” before validation.”

- There is quite a few typing and grammatical errors. For instance:

	*Figure 2B: Max mamory<br>*Supplementary Table 3: Memery<br>*Page 3, line 53: ...the number of reads aligned to of a particular region.<br>*Page 8, line 160: This model presets the average copy number of homozygous deletion, heterozygous deletion, normal, heterozygous deletion (duplication!), homozygous deletion (duplication!) at zero to four respectively.<br><br>We are sorry for these mistakes. We have proofread the revised manuscript and used a professional English-language editing service to minimize the grammatical errors.<br><br><br>************************<br><br>Checklist of the updated tables and figures<br>Current versionLast version<br>Fig. 3Fig. 5<br>Fig. 4A-CTable 1 and newly added<br>Fig. 4DFig. 3B<br>Fig. 5Fig. 4<br>Fig. 6A-CTable 1 and newly added<br>Fig. 6DFig. 3A<br>Supplementary Fig. 1Newly added<br>Supplementary Fig. 2Supplementary Fig. 1<br>Supplementary Fig. 3Newly added<br>Supplementary Fig. 4Supplementary Fig. 2<br>Supplementary Fig. 5Table 1 and newly added<br>Supplementary Table 4Newly added<br>Supplementary Table 5Newly added
**Additional Information:**	
**Question**	**Response**
Are you submitting this manuscript to a special series or article collection?	No
**Experimental design and statistics**<br><br><br>Full details of the experimental design and statistical methods used should be given in the Methods section, as detailed in our Minimum Standards Reporting Checklist. Information essential to interpreting the data presented should be made available in the figure legends.<br><br><br>Have you included all the information requested in your manuscript?	Yes
**Resources**<br><br><br>A description of all resources used, including antibodies, cell lines, animals and software tools, with enough information to allow them to be uniquely identified, should be included in the Methods section. Authors are strongly encouraged to cite Research Resource Identifiers (RRIDs) for antibodies, model organisms and tools, where possible.	Yes

Have you included the information requested as detailed in our Minimum Standards Reporting Checklist?	
**Availability of data and materials**<br><br>All datasets and code on which the conclusions of the paper rely must be either included in your submission or deposited in publicly available repositories (where available and ethically appropriate), referencing such data using a unique identifier in the references and in the “Availability of Data and Materials” section of your manuscript.<br><br>Have you have met the above requirement as detailed in our Minimum Standards Reporting Checklist?	Yes

Powered by Editorial Manager® and ProduXion Manager® from Aries Systems Corporation

Manuscript Click here to download Manuscript 10_21.docx

CNVcaller: Highly Efficient and Widely Applicable Software for Detecting Copy Number Variations in Large Populations

Xihong Wang [1†], Zhuqing Zheng[1†], Yudong Cai[1], Ting Chen[1], Chao Li[1], Weiwei Fu[1], Yu Jiang[1]*

1 College of Animal Science and Technology, Northwest A&F University, Yangling, Shaanxi, China

† These authors contributed equally to this work.

*Correspondence should be addressed to Yu Jiang (yu.jiang@nwafu.edu.cn), ORCID: 0000-0003-4821-3585.

Abstract

Background: The increasing amount of sequencing data available for a wide variety of species can be theoretically used for detecting copy number variations (CNVs) at the population level. However, the growing sample sizes and the divergent complexity of non-human genomes challenge the efficiency and robustness of current human-oriented CNV detection methods.

Results: Here, we present CNVcaller, a read-depth method for discovering CNVs in population sequencing data. The computational speed of CNVcaller was 1-2 orders of magnitude faster than CNVnator and Genome STRiP for complex genomes with thousands of unmapped scaffolds. CNV detection of 232 goats required only 1.4 days on a single compute node. Additionally, the Mendelian consistency of sheep trios indicated that CNVcaller mitigated the influence of high proportions of gaps and misassembled duplications in the non-human reference genome assembly. Furthermore, multiple evaluations using real sheep and human data indicated that CNVcaller

achieved the best accuracy and sensitivity for detecting duplications.

Conclusion: The fast, generalized detection algorithms included in CNVcaller overcome prior computational barriers for detecting CNVs in large-scale sequencing data with complex genomic structures. Therefore, CNVcaller promotes population genetic analyses of functional CNVs in more species.

Keywords

Copy number variation (CNV), next-generation sequencing (NGS), read depth (RD), population genetics, absolute copy number.

Introduction

Copy number variations (CNVs) are defined as duplications or deletions of genomic segments that range in size from 50 base pairs (bp) to megabase pairs (Mb) and vary among individuals or species [1]. As a prevalent and important source of genetic diversity, over 50,000 CNVs have been detected in the human genome, accounting for 10% of the entire genome [2]. CNVs regulate gene expression via both gene dosage and position effects, and they have larger expression-altering effect sizes than single nucleotide polymorphisms (SNPs) and indels [3]. In the human genome, CNVs are important genetic components of numerous diseases [4, 5] and a primary driving forces of evolution [6]. Furthermore, CNVs are associated with different phenotypes and functions in animals and plants [7-11].

With the dramatic increase in sequencing capacity and the accompanying decrease in sequencing cost, whole-genome sequencing data is becoming the main source of CNV detection.

The large-scale population sequencing data also provide an unprecedented opportunity to discover the functional CNVs using genome-wide association studies (GWAS) and evolutionary analysis [11, 12]. To study the polymorphism among individuals, the overlapping CNVs need to be merged into unified regions, namely CNV regions (CNVRs) [13]. Since merging CNVs identified in each individual is inconvenience for large populations, some methods use multiple samples as input, then output the CNVRs directly [14]. More importantly, the population-based methods can improve the detection by building statistic models, such as Poisson distribution and Gaussian Mixture model [15, 16].

As the amount of data increases, the computational efficiency is becoming a rate-limiting factor in CNV analysis. In additional, most CNV detection algorithms are based on mapping the sequencing reads back to the reference genome. For example, the methods of read-pair (RP) and split-read (SR) deduce the breakpoint of CNVs from the discordant alignments [17-21]; the methods of read depth (RD) refers to the depth of coverage in a genomic region that can be calculated from the number of aligned reads [14]. A duplicated or deleted region should have a higher or lower RD than expectation [22-24]. However, the non-model genome assemblies are riddled with many gaps, unplaced scaffolds and misassembled segmental duplications (SDs) [25-28]. For example, 97% of highly similar tandem duplications in the Btau4.1 cattle genome assembly actually correspond to a single copy [29]. Therefore, more robust signal detection and noise reduction algorithms are required for detecting CNVRs from non-model species.

In this study, we introduce a super-fast, generalized method, CNVcaller, for analysing CNV sequencing data in large populations (CNVcaller, RRID: SRC_015752). Based on the RD algorithm, this software applies robust signal detection and noise deduction methods to increase

the computational efficiency in complex genomes. We applied CNVcaller to population sequencing data of humans, livestock and crops to demonstrate its utility and benchmarked it against the widely used CNV detectors.

Materials and Methods

Input data

CNVcaller requires alignment files in BAM format as the main input. The following data/samples were included in the validation. 1. Human. Thirty human BAM files from the 1000 Genomes Project (1000GP) Phase 3 [30], including 27 normal (~12X) and three deeply sequenced samples (~50X), and 30 BAM files (~20X) for 10 families from the Genomes of Netherlands (GoNL) project [31]. 2. Sheep. Seventy FASTQ files were downloaded from the NCBI BioProject: PRJNA160933 (~10X). Three Tan sheep trios (~19X, including a total of 8 individuals; one ewe was the mother of two trios) were from unpublished data. 3. Goat. A total of 103 FASTQ files were acquired from NCBI [32-35], and the remaining 129 were generated by ourselves (~12X, unpublished data). 4. Plant. Two maize [36] and two soybean [11] FASTQ files (each species containing one ~5X and one ~10X sample) were downloaded from NCBI. Details of the downloaded files are provided in **Supplementary Table 1**.

The FASTQ files were aligned to their respective reference assemblies using Burrows-Wheeler Aligner (BWA) 0.7.13(BWA, RRID:SCR_010910) [37]. The versions of the reference genomes included human GRCh37, maize B73 RefGen_v3, soybean Glycine_max_v2.0, sheep OAR_v3.1 and goat ARS1. After alignment, the PCR duplications were marked using

Picard 2.1 (https://broadinstitute.github.io/picard), and realignment was performed by GATK v3.5 (GATK, RRID:SCR_001876)[38]. The reads with a 0x504 flag (indicating unmapped, secondary mapped or PCR duplication) were removed.

Individual RD processing

RD estimation. The pipeline of CNVcaller is shown in **Figure 1**. To calculate the RD signal, we first divide the reference genome into overlapping sliding windows, which is used for all samples. Windows with >50% gaps are excluded from the database and further computation. Then, the BAM file for each individual is parsed out using SAMtools v1.3 (SAMTOOLS, RRID:SCR_002105)[39] and the RD signal is calculated for each window as the number of placed reads with centres within the window boundaries. Considering the uncontrollable effect of gap ratios from different genome assemblies, all of the end reads located in the window are independently added to the RD of this window, regardless of whether the read is from single-end mapping or paired mapping. The window size is an important parameter for RD methods. CNVcaller uses half of the window size as the step size. The optimal window size is 800 bp (with a 400 bp overlap) for 5-10X coverage human and livestock sequencing data **(Supplementary Figure 1)**. The recommended window sizes are inversely related with coverage, and thus, ~400 bp windows correspond to 20X coverage, and ~200 bp windows correspond to 50X coverage.

Absolute copy number correction. To perform absolute copy number correction, windows with >97% sequence similarity are linked together to form a duplicated window record file. This file is generated by splitting the reference genome into non-overlapping windows and aligning the

windows onto the reference genome using the precise aligner BLAT v. 36x1 [40]. Windows with more than 20 hits are excluded to remove the low-complexity regions. The record files for humans, livestock and main crops can be downloaded from the CNVcaller website (http://animal.nwsuaf.edu.cn/). Based on the duplicated window record file, the raw RDs located on similar windows are summed to generate the absolute RD for all high-similarity windows,

$$RD^{i}_{absolute} = \sum_{j=1}^{t} RD^{ij}_{raw}$$

where i is the index of the window to be corrected, t is the total number of the high-similarity windows, RD^{ij}_{raw} is the raw RD of the window that is similar to the i-th window (including the i-th window itself), which is counted directly from the BWA alignment, and $RD^{i}_{absolute}$ is the corrected RD of the i-th window, which can be used to deduce the absolute copy number.

GC correction. Considering that the population sequencing data may come from different platforms, the RD of each individual sample is counted and corrected. Because the resequencing samples may show various GC content distributions, the GC bias is corrected individually, similar to the method used in CNVnator (CNVnator, RRID:SCR_010821) [23]:

$$RD^{i}_{corrected} = \frac{\overline{RD}_{40}}{\overline{RD}_{gc}} RD^{i}_{absolute}$$

where i is the window index, $RD^{i}_{absolute}$ is the RD after absolute copy number correction, $RD^{i}_{corrected}$ is the final corrected RD for the window, $\overline{RD}_{40}$ is the mean RD of windows with 40% GC as a standard, and $\overline{RD}_{gc}$ is the mean RD over all windows that have the same GC content as the i-th window.

RD normalization. Because the samples have different sequencing depths, the corrected RD must be normalized to a single standard before population-level CNV detection. Assuming that the majority of the genome has normal copy numbers, the corrected RDs are divided by the global median RD for normalization to one,

$$RD^{i}_{normalized} = \frac{RD^{i}_{corrected}}{\overline{RD}_{global}}$$

where $\overline{RD}_{global}$ is the median of the $RD^{i}_{corrected}$ of all windows.

RD corrections for sex chromosomes. Most mammalian and avian genomes show an XX/XY-type or ZZ/ZW-type sex-determining system. Their homogametic sex chromosomes (X or Z) constitute 5%-10% of the total genome and show half the RD of the autosomes in XY or ZW individuals. Therefore, intensive correction for X and Z chromosomes is needed. The RD of the X or Z chromosome (the particular name provided by the user) is used to determine the sex of a particular individual. If the median RD of this chromosome is <0.6X the median RD of the autosome, the individual is considered an XY or ZW type, and the RDs of this chromosome are doubled before normalization. Otherwise, nothing is performed for individuals determined to be XX or ZZ type.

Parallel processing of individual RDs. The CNVcaller processes the BAM file of each individual separately in the first step, and therefore, parallel computations can be performed to reduce the total running time. All BAM files are equally distributed into N groups, and each group contains M files. The max N is the total available processing cores, and M is the total number of BAM files/N. For example, the 232 goat BAM files were processed on a node with 32 processing cores and 124 GB of RAM. We distributed the 232 files into 20 groups, and each group contained 12

BAM files. The shell command for one group is as follows:

```
#!/bin/sh
for i in {1..M}
  do bash Individual.Process.sh -b $i.bam -h $i -d dup -s sex_chromosome
done
```

After corrections and normalization, the comparable RDs of each sample are aggregated into an ~100 MB intermediate file and output, thus preventing repeated calculations for the same individual in different populations.

CNVR detection by multiple criteria

Individual candidate CNV window definition. The individual candidate CNV windows are defined using two criteria: (1) The normalized RD must be significantly higher or lower than the normalized mean RD (deletions < 1 – 2 * STDEV; duplications > 1 + 2 * STDEV). (2) Considering that the normalized RD of heterozygous deletions and duplications should be approximately 0.5 and 1.5, respectively, an empirical standard for the normalized RD (deletions < 0.65; duplications > 1.35) also must be achieved. For some strictly self-bred species, such as soybean and wheat, this empirical standard should be raised to 0.25 or 1.75 for the normalized RD of the homozygous deletions or duplications, respectively.

Population-level candidate CNV window definition. All the individual RD files are arranged according to the universal window index into a two-dimensional population RD file. Each line of this file is the multi-sample RDs of a particular window, from which the candidate CNV windows

are selected. The user can retain all the windows with at least one individual that shows heterozygous deletion or duplication. However, we recommend removing low-frequency windows in large populations with low sequencing coverage because of increased random mistakes. By default, windows with an allele frequency ≥ 0.05 or at least two homozygous duplicated/deleted individuals are selected for further validation. Then, Pearson's product-moment correlation coefficients of the multi-sample RDs are calculated between two adjacent non-overlapping windows. If the Pearson's correlation index is significant at the $P = 0.01$ level by Student's t test, the two windows are merged into one call.

CNV region definition. The initial calls are selected if more than four sequential overlapping windows are defined as population-level candidate windows. Regarding noise tolerance, a maximum of one unselected window out of four continuous candidate windows is allowed; however, their RD is not calculated in the RD of CNVR. As CNVRs can be separated by gaps or poorly assembled regions, the adjacent initial calls are merged if their RDs are highly correlated. The default parameters are as follows: the distance between the two initial calls is less than 20% of their combined length, and the Pearson's correlation index of the two CNVRs is significant at the $P = 0.01$ level.

CNVR genotyping

After merging the candidate CNV windows into a CNVR, the RDs of all samples in each CNVR are clustered, and the integer copy number of each individual is calculated, which represents the

genotyping step as used in SNP detection. The copy number of a specific sample is initially estimated as two times the median RD of all candidate windows in a given region. Then, the copy numbers of all samples of a CNVR are decomposed into several Gaussian distributions. The expectation maximization (EM) algorithm is used to estimate the model parameters, and the effective number of components is inferred by the Dirichlet Process. To ensure the quality of the genotyping, the silhouette coefficient is calculated for each CNVR. The Python package scikit-learn v0.19.0 [41] is used to implement the above algorithms. This genotyping step can be performed in sequence or in parallel, and the parameter "nproc" is used to control the number of processes. The genotyping of 232 goats took 17.49 minutes and 488 MB of memory on one node with two processors. The final output is a VCF file, which can be analysed by SNP-based population genetic software.

Performance evaluation

Competing methods. Most of the validations were based on the 30 human BAM files form 1000GP Phase 3 data, and only the autosomes were included unless otherwise noted. The performance of CNVcaller was compared with two pipelines, including CNVnator_v0.3.3 (CNVnator, RRID:SCR_010821) [23], which is widely used for CNVR detection in animal populations, and Genome STRiP (included in svtoolkit_2.00.1696) [16], which is the state-of-the-art CNV detector generated by 1000GP. The recommended parameters and quality controls were used. For Genome STRiP, both the deletion and CNV pipelines were utilized. The unplaced scaffolds were excluded, and the whole genome was separated by chromosomes as

recommended. The standard screening procedures were applied to select the passing sites and to remove duplicate calls. For CNVnator, a 400 bp window was used, as recommended. The gap regions and calls with p values less than 0.01 were removed, and the q0 filter was used to remove any predictions with q0 <0.5 (reads with multiple mapping locations), as recommended. The individual CNVs of all samples were merged into the population CNVRs based on the following arbitrary standards: two calls with >50% reciprocal overlap or one call with >90% coverage by another call [23, 42]. Then, the CNVRs were genotyped using the built-in function in CNVnator.

Sensitivity validation. Sensitivity was defined as the number of CNVs existed in both the CNV predictions and the high-confident CNVR database (>50% reciprocal intersection) divided by the total number of CNVs in the database. Calls with ≤2,500 bp and an allele frequency <5% and sex chromosomes were removed from this study. Two previously published high-confident CNVR databases, including the same samples from the test data, were used. One was the 1000GP CNVR map [44], which included 26 tested samples, and the other was array comparative genomic hybridization (aCGH)-based CNVR database [1], which included 10 tested samples. The CNVRs of the specific samples were extracted from the database and were then screened by the same length and frequency as the detected CNVRs (length >2,500 bp and alternative allele frequency ≥0.05). The intersected length of the predicted CNVRs and the high-confidence CNVR database was calculated using BEDTools v2.25 (BEDTools , RRID:SCR_006646)[43].

Accuracy validation. The intensity rank-sum (IRS) test (included in the svtoolkit_2.00.1696) was performed as previous studies [16], based on the Affymetrix SNP 6.0 array intensity data of 26 test

samples. Meanwhile, the genotyping accuracy was benchmarked against the aCGH CNVR database [1]. The predicted CNVs were subject to validation if the predicted regions had a >90% reciprocal intersection with one CNVR in the database. Only if the predicted genotyping was in exact agreement with the aCGH database, this genotyping was defined as correct. Also, the Mendelian inconsistencies were calculated from the deleted and biallelic duplicated CNVRs (maximum copy number ≤4) in the Dutch families and sheep trios.

Sheep genotyping validation by CNVplex assay. A total of 73 sheep, including Merino, Texel, Mongolia and Tibetan sheep, were used for genotyping validation. Genomic DNA was extracted from peripheral blood using a QIAamp DNA blood mini kit (Qiagen, Germany). For each sheep, whole-genome sequencing (~10X) was performed, and the CNVRs were detected by CNVcaller as described above. The copy numbers of high variant CNVRs were validated by CNVplex® (Genesky Biotechnologies Inc., Shanghai, China), which is based on double ligation and multiplex fluorescence PCR [44]. The sizes of the PCR fragments and target loci sequences used in each reaction are listed in **Supplementary Table 3.**

Absolute copy number validation

Detecting X-origin scaffolds. Unplaced scaffolds with high sequence similarity to the X chromosome were regarded as X-origin scaffolds. All the scaffolds of OAR v3.1 were mapped to the X chromosome of the sheep reference genome OAR v4.0, the goat reference genome ARS1 and the cattle reference genome UMD 3.1 using BLASR [45]. If the best hit of a scaffold had >50%

coverage with >90% identity and >3 kb length, this scaffold was defined as a putative X-origin scaffold. In theory, all of these scaffolds were expected to be detected as high-frequency CNVRs because the RDs of the unplaced scaffolds were not corrected by sex. The detection and genotyping accuracy in the SD region was estimated using the sex information from 133 sheep.

mrsFAST alignment. The paired-end reads with multiple hits indicated by the "XA" tag in the BWA alignment were selected for realignment using mrsFAST_v3.3.10 [46] as previously described [47]. Longer reads were trimmed to 40 bp to reduce the read length heterogeneity prior to sequence alignment. After alignment, the reads with more than 20 hits were excluded to remove the low-complexity regions.

Simulations of the SD. The putative SDs were modified from a randomly selected 50 Mb single-copy region of Chr1 from the sheep reference (OAR v3.1). 100 non-overlapping regions of 5,000 bp were randomly selected and artificially inserted as tandem duplications into the 50 Mb source sequence. The modified sequence with known SDs were used as reference genome in the following study. In these putative SD regions, 2-6 copies were randomly assigned to 100 individuals, and all other regions are treated as normal copy regions. The wgsim [39, 48] read simulator was used to sample the pair end reads with default parameters. The coverage of the normal regions was set to 20X.

Results and discussion

Computational cost in complex genomes from large population-based studies

Since the computational cost is one of the greatest challenges for large populations, the computational efficiency of CNVcaller was evaluated on the real sequencing data of the different genomes. The individual RD processing step was compared to CNVnator, which detects CNVs individually, and has been used in yak, chicken and fish populations [42, 49, 50]. The processing time of CNVcaller was linearly related to the genome size and sequencing coverage: 20-40 minutes for a 3 Gb genome with 10X coverage **(Supplementary Table 3)**. However, the processing time of CNVnator exponentially increased with scaffold number, which was the only time-consuming index when the scaffold number exceeded one thousand **(Figure 2A)**. Consequently, CNVcaller achieved a 145-fold increase in speed over CNVnator for goat CNV detection. Notably, the goat reference genome ARS1, which contains 29,907 scaffolds, was newly assembled by single-molecule sequencing [35]. The robustness of CNVcaller reduces the quality restrictions of the reference genome, which promotes CNV research in species with a draft assembly at the scaffold level. This feature also enables comprehensive variation discoveries based on pan-genomes, which reveal numerous functionally important genes not localized on a single reference genome [51-53].

The memory requirement of CNVcaller is mainly related to the genome size: only approximately 500 MB memory for a mammalian genome, which is less than one twentieth of the memory required by CNVnator **(Figure 2B)**. Therefore, in multi-sample CNV detection, the individual RD processing step can be run in parallel on one node to further reduce the running time. The population-level performance of CNVcaller was evaluated and benchmarked against

Genome STRiP, which also detects CNVRs at the population level, and is a main contributor of the 1000GP. After removing the unplaced scaffolds, CNVcaller was still 3.5-7.8 times faster than Genome STRiP **(Figure 2C)**, with a 70%~86% reduced memory requirement **(Figure 2D)**. For 232 goats with a mean coverage of 12X, CNVcaller can complete CNV detection in 1.4 days using a single node. The high efficiency of CNVcaller can facilitate CNV detection in large populations.

Absolute copy number correction in putative SDs of the sheep genome

Previous studies have shown that a high proportion of SDs in animal genomes are misassembled single-copy regions [27, 29]. Therefore, we detected the ratios of false SDs on the human (hg19) and sheep (OAR v3.1) reference genome assemblies by the sequencing copy number of a human (NA12878) and a Tan sheep sample **(Figure 3A)**. If the SDs were correctly assembled, the sequencing diploid copy number should be twice the copy number of SDs. For example, the average sequencing copy number of the two-copy SDs was four in NA12878. However, the corresponding sequencing copy number in sheep was only 2.4. These results indicated that most two-copy SDs of hg19 were truly duplicated in NA12878, while approximately 80% of the two-copy SDs in OAR v3.1 were single-copy regions in the Tan sheep sample. Thus, the SDs in the sheep genome were called “putative SDs” before validation.

CNV detection can be confounded by the presence of false SDs. Due to the random placement of multiple mapped reads, the RD signal in these regions is effectively smeared over all copies; thus, the raw copy number is underestimated. For example, in the putative two-copy SDs, the main peak of the copy numbers was one, the same as heterozygous deletions **(Figure 3B)**.

CNVcaller incorporates absolute copy number correction by simply adding the RD of the putative SDs to deduce the absolute copy number independent of the copy number of the genome assembly **(Figure 1)**. This target can also be achieved using mrsFAST; however, more than 10 core hours were required to realign the multi-hit reads by mrsFAST for a mammalian genome with 10X sequencing coverage. The equivalent result was achieved by CNVcaller within only 0.06 core hours **(Supplementary Figure 2)**.

In the simulate sheep sequencing data, this correction can deduce the correct genotyping in SD regions **(Supplementary Figure 3)**, also reduced the STDEV within each genotyping **(Supplementary Table 4)**. In the real individual sheep data, the corrected putative two-copy SDs clearly fall in to two categories: normal copy (the major peak, with a diploid copy number of two) and the true duplicated regions (the minor peak, with a diploid copy number of four) **(Figure 3B)**. The accuracy of the CNVRs in putative SDs was validated by the Mendelian inconsistency of three Tan sheep trios. CNVcaller detected more duplications in the putative SDs, with only 3% Mendelian inconsistency (**Figure 3C**).

The sensitivity of sheep CNVRs was estimated indirectly due to the lack of a validated database. Based on our integrated analysis (see methods), there were 138 sheep X chromosome-origin scaffolds, which were not anchored onto chromosomes of OAR v3.1. Therefore, all of these scaffolds should be detected as CNVs because the rams had half the copy numbers of the ewes. As a result, CNVcaller detected 101 of these 138 X-origin scaffolds, with a sensitivity of 73%. In contrast, CNVnator and Genome STRiP did not report these regions. Furthermore, the copy numbers of single-copy and duplicated X-origin scaffolds were centred at integers, namely one and two in rams and doubled in ewes, whereas the peaks of the raw copy numbers were

ambiguous **(Figure 3D)**. Further examination of the duplicated regions showed that this result was caused by splitting the raw RDs among the putative SDs **(Supplementary Figure 4)**.

Performance evaluations on sheep data

To evaluate the robustness and false discovery rate (FDR) in sheep, we used CNVcaller, CNVnator and Genome STRiP to detect CNVRs from three sheep trios. CNVnator detected less than 1/10 the number of CNVRs of the other methods, with only 260 CNVRs reported. One main reason was that assembly gaps without reads were detected as homozygous deletions by CNVnator in the initial calls. Thus, more than 90% of the initial calls were removed in the recommended gap filtering step because the sheep reference genome OAR v3.1 has ~125,000 gaps. By comparison, the human reference genome hg19 has only 354 gaps. To address the gap problem in non-human genomes, CNVcaller removed the sliding windows with gaps at the first step, and adjacent CNVRs were merged into one call if their RDs were ultimately highly correlated. These optimizations avoided the artefacts caused by assembly errors and retained the adjacent CNVRs as well.

The accuracy was evaluated by the Mendelian inconsistency of all the CNVRs on autosomes against the length and alternative allele frequency **(Figure 4)**. CNVcaller achieved higher accuracy than Genome STRiP in both deletion (1% vs 2%) and duplication (4% vs 7%) (**Figure 4A**). Whereas Genome STRiP had greater capability to detected short (<2.5 kb) deletions **(Figure 4B)**, indicating the RP methods integrated in Genome STRiP performed well on small deletions. Concerning the alternative allele frequency, both methods showed an increased FDR in rare duplications (**Figure 4C)**. However, CNVcaller is primarily used to detect CNVRs related to

economic traits in livestock and crops. In these studies, the target CNVRs usually have a high frequency after long-duration breeding selection.

To investigate the reproducibility of CNVcaller, the CNVRs identified by CNVcaller from 133 sheep of 44 worldwide breeds were compared with two other recently released large-scale sheep CNVR datasets. One dataset was derived from allied breeds using multiple platforms, including aCGH, SNP chip and whole-genome sequencing [54], and the other dataset was based on three Chinese sheep breeds using a 600K SNP array [55]. The samples and platforms had major influences on the results; therefore, the overall intersection ratio was low. However, CNVcaller covered 51% of the cross-validated regions of the other dataset **(Figure 4D)**.

The genotyping accuracy of 73 sheep was validated by a recently developed molecular biology technique, CNVplex. This method reports the copy number of a genomic sequence based on the multiplex ligation-dependent probe amplification (MLPA) method [44]. When the copy numbers of sequencing data predicted by CNVcaller and CNVplex were compared, the Pearson's product–moment correlation coefficients were greater than 0.95, and the genotyping concordance was 98% **(Figure 5).**

Performance evaluations on 1000 Genomes Project data

Although CNVcaller was mainly designed for complex genomes, the performance was also evaluated on 30 human BAM files from 1000GP. Because the SNP array data and high-confidence CNVR databases were only available in human, which can be used to evaluate the accuracy from population-level (IRS test) and sensitivity. CNVcaller demonstrated the highest overall accuracy for detecting duplications and performed consistently across the length and

frequency categories, whereas Genome STRiP and CNVnator had high FDRs on the short or singleton duplications **(Figure 6A, B)**. Genome STRiP showed the greatest ability to detect deletions, indicating the advantage of combining RD and RP methods for deletion detection. The genotyping accuracy of the human dataset was further benchmarked against the high-confidence aCGH array-based database. The discordance rates of CNVcaller, CNVnator and Genome STRiP were 2.6%, 5.5% and 2.2%, respectively. This genotyping accuracy ranking was the same with the Mendelian consistency of the 10 Dutch trios **(Supplementary Figure 5)**.

The sensitivity was estimated as the proportion of the high-confidence CNVR database that overlapped with the predicted CNVRs. Two previously published high-confidence databases that include our test samples are the aCGH-based CNVR database [1] and the 1000GP CNVR map [2]. For the aCGH database, CNVcaller demonstrated the highest sensitivity (57%) in duplications, whereas Genome STRiP achieved the highest sensitivity (74%) in deletions **(Figure 6C)**. Both Genome STRiP and CNVnator were the core contributors to the 1000GP CNV maps; However, the sensitivity of CNVcaller was 68% and 67% for deletions and duplications according to this database, only 4%-10% lower than Genome STRiP and CNVnator.

The three methods had a high degree of intersection with each other. The number of overlapping (>50%) calls was 429 (CNVcaller vs CNVnator), 502 (CNVcaller vs Genome STRiP) and 513 (CNVnator vs Genome STRiP). CNVcaller covered 40% of the CNVRs detected by CNVnator, 45% of the CNVRs detected by Genome STRiP and 65% of their intersecting CNVRs by length **(Figure 6D)**.

Conclusion

CNVcaller was designed to detect CNVRs from large-scale resequencing data from all types of genomes. This generalized detection and correction algorithms employed in CNVcaller greatly increase the computational efficiency of analysing complex genomes. The validation performed using sheep data showed that the absolute copy number correction increased the detection efficiency of the misassembled SDs, greatly reduced the running time, and deduced more reasonable copy numbers. Both the evaluations using sheep and human data indicated that CNVcaller achieved the best accuracy and sensitivity for detecting duplications. Therefore, this rapid and reliable population-level CNV detection method can promote the discovery of the missing heritability of complex traits and the accurate determination of causative mutations in more species.

Availability and requirements

Project name: CNVcaller

RRID: SRC_015752

Project home page: http://animal.nwsuaf.edu.cn/software

https://github.com/JiangYuLab/CNVcaller

Operating system(s): platform independent

Programming language: Perl, Python

Other requirements: SAMtools 1.3 (using htslib 1.3), scikit-learn v0.19.0

License: GNU General Public License, version 3.0 (GPL-3.0)

Availability of data

Snapshots of the supporting code and materials are hosted in the GigaScience GigaDB repository[56].

Conflict of interest

The authors declare that they have no competing interests.

Author contributions

WXH and JY designed the software; ZZQ and CT wrote the code; WXH and ZZQ improved the pipeline structures; ZZQ and CYD tested the software prototype; LC and FWW contributed to the data organization; and WXH and JY drafted the manuscript. All authors read and approved the final manuscript.

Acknowledgements

This work was supported by grants from the National Natural Science Foundation of China (31572381) and the National Thousand Youth Talents Plan. We thank the International Sheep Genomics Consortium (ISGC) for access to the unpublished sheep sequencing data provided under the Toronto guidelines for data users. We thank the NextGen Project for the goat sequencing data. We thank the Genomes of Netherlands (GoNL) project for the human family data.

Figure Legends

Figure 1 CNVcaller algorithm flowchart (left) and the key algorithms of each step (right). (1)

Individual RD processing. In the absolute copy number correction, the RDs of highly similar windows were added together to deduce the absolute copy number. (2) Multi-criteria CNVR selection. The curves show the copy numbers in a specific region for multiple samples. The blue transverse boxes mark the windows with a significant distinguishing copy number from the average (individual criterion). The green vertical boxes indicate that these regions meet the frequency conditions, and the red frame indicates that the RDs between the two adjacent windows are significantly correlated (population criteria). The forth bar from the left, satisfying all the above conditions, is selected as the CNVR. (3) Genotyping: The copy numbers in each CNVR are clustered by a mixture Gaussian model to distinguish the normal, heterozygous and homozygous samples.

Figure 2 Computational performance of CNVcaller, CNVnator and Genome STRiP. All the programs were executed on a single node with two 2.40-GHz Intel Xeon E5-2620 v3 processors. (A, B) Log plots of the processing time (A) and the max memory (B) for one individual. The numbers of unplaced scaffolds of the reference genome are indicated in brackets. The processing time was normalized by the genome size and sequencing coverage to simulate a 3 Gb genome with 5X or 10X sequencing coverage. (C, D) Log plots of the total running time (C) and the max memory (D) of the population CNVR detection. The test cohorts are as follows: 8 sheep, 30 humans and 232 goats with 19X, 16X and 12X average sequencing coverage, respectively. In Genome STRiP, the unplaced scaffolds were excluded.

Figure 3 Absolute copy number correction in the sheep genome. (A) The copy numbers of all the

windows with no more than six repeats were plotted against the repeat numbers in the reference genome. Compared with humans, the sheep sample had much lower copy numbers in the putative duplicated regions than expected. (B) The distribution of copy numbers of the putative two-copy regions in the sheep genome before and after absolute copy number correction. After correction, the main peak of the copy number shifted to two (normal diploid copy number). The smaller peaks at four, after correction, indicated the 20% real SDs. (C) The number and FDR (Mendelian inconsistency) of detected CNVRs residing in the SD regions. The sheep SD regions include the regions longer than 2 kb with >97% identity. The CNVRs residing in the SD regions were defined if more than 50% of a given CNVR overlapped with the SD regions. (D) The raw and corrected copy numbers of all the X-linked scaffolds of 133 sheep.

Figure 4 Performance evaluations on the sheep data. (A) CNVR number and FDR (Mendelian inconsistency) of three sheep trios. (B) The number of calls and FDR partitioned by CNV length. (C) The number of calls and FDR partitioned by allele frequency. The frequency was showed by the alternative allele number. (D) The length of overlapping CNVRs (in Mb) detected by CNVcaller and two other large-scale sheep studies with different approaches and platforms.

Figure 5 Evaluation of the sheep CNV genotypes by CNVplex. Two duplicated (A, B) and two deleted (C, D) CNVRs with a high variation frequency were typed in CNVplex using 73 sheep samples. The copy number genotypes predicted by CNVcaller from the sequencing data were plotted against the CNVplex measurements of the same animal.

Figure 6 Performance evaluations on the 1000GP data. (A, B) The number of calls (A) and the IRS FDR (B) partitioned by CNV length. All the calls on the autosomes were included. (C, D) The number of calls (C) and the IRS FDR (D) partitioned by allele frequency. To eliminate the huge FDR diversity of the short CNVs, the effect of the allele frequency was evaluated using the >2,500 bp calls. (C) The sensitivity (the proportion of high-confidence CNV database overlapped by the predicted CNVs) of the three methods. For the highly variable FDRs, the sensitivity estimation removed the calls less than 2,500 bp or had an alternative allele frequency less than 5%. (D) Comparison of CNVR results identified by CNVcaller, CNVnator and Genome STRiP based on the same 30 BAM files from the 1000GP Phase3. The length of overlapping CNVRs was indicated in Mb.

References

1. Conrad DF, Pinto D, Redon R, Feuk L, Gokcumen O, Zhang Y, et al. Origins and functional impact of copy number variation in the human genome. Nature. 2010;464 7289:704-12.
2. Sudmant PH, Rausch T, Gardner EJ, Handsaker RE, Abyzov A, Huddleston J, et al. An integrated map of structural variation in 2,504 human genomes. Nature. 2015;526 7571:75-81.
3. Chiang C, Scott AJ, Davis JR, Tsang EK, Li X, Kim Y, et al. The impact of structural variation on human gene expression. Nature genetics. 2017.
4. Khurana E, Fu Y, Colonna V, Mu XJ, Kang HM, Lappalainen T, et al. Integrative annotation of variants from 1092 humans: application to cancer genomics. Science.

2013;342 6154:1235587.

5. Stefansson H, Meyer-Lindenberg A, Steinberg S, Magnusdottir B, Morgen K, Arnarsdottir S, et al. CNVs conferring risk of autism or schizophrenia affect cognition in controls. Nature. 2014;505 7483:361-6.

6. Sudmant PH, Mallick S, Nelson BJ, Hormozdiari F, Krumm N, Huddleston J, et al. Global diversity, population stratification, and selection of human copy-number variation. Science. 2015;349 6253:aab3761.

7. Norris BJ and Whan VA. A gene duplication affecting expression of the ovine ASIP gene is responsible for white and black sheep. Genome research. 2008;18 8:1282-93.

8. Giuffra E, Törnsten A, Marklund S, Bongcam-Rudloff E, Chardon P, Kijas JM, et al. A large duplication associated with dominant white color in pigs originated by homologous recombination between LINE elements flanking KIT. Mammalian Genome. 2002;13 10:569-77.

9. Wright D, Boije H, Meadows JR, Bed'Hom B, Gourichon D, Vieaud A, et al. Copy number variation in intron 1 of SOX5 causes the Pea-comb phenotype in chickens. PLoS Genet. 2009;5 6:e1000512.

10. Seo B-Y, Park E-W, Ahn S-J, Lee S-H, Kim J-H, Im H-T, et al. An accurate method for quantifying and analyzing copy number variation in porcine KIT by an oligonucleotide ligation assay. BMC genetics. 2007;8 1:81.

11. Zhou Z, Jiang Y, Wang Z, Gou Z, Lyu J, Li W, et al. Resequencing 302 wild and cultivated accessions identifies genes related to domestication and improvement in

soybean. Nature biotechnology. 2015;33 4:408-14.

12. Zhao P, Li J, Kang H, Wang H, Fan Z, Yin Z, et al. Structural Variant Detection by Large-scale Sequencing Reveals New Evolutionary Evidence on Breed Divergence between Chinese and European Pigs. Scientific reports. 2016;6.

13. Redon R, Ishikawa S, Fitch KR, Feuk L, Perry GH, Andrews TD, et al. Global variation in copy number in the human genome. Nature. 2006;444 7118:444-54.

14. Zhao M, Wang Q, Wang Q, Jia P and Zhao Z. Computational tools for copy number variation (CNV) detection using next-generation sequencing data: features and perspectives. BMC bioinformatics. 2013;14 11:S1.

15. Klambauer G, Schwarzbauer K, Mayr A, Clevert D-A, Mitterecker A, Bodenhofer U, et al. cn. MOPS: mixture of Poissons for discovering copy number variations in next-generation sequencing data with a low false discovery rate. Nucleic acids research. 2012:gks003.

16. Handsaker RE, Van Doren V, Berman JR, Genovese G, Kashin S, Boettger LM, et al. Large multiallelic copy number variations in humans. Nature genetics. 2015;47 3:296-303.

17. Chen K, Wallis JW, McLellan MD, Larson DE, Kalicki JM, Pohl CS, et al. BreakDancer: an algorithm for high-resolution mapping of genomic structural variation. Nat Meth. 2009;6 9:677-81.

18. Hormozdiari F, Hajirasouliha I, Dao P, Hach F, Yorukoglu D, Alkan C, et al. Next-generation VariationHunter: combinatorial algorithms for transposon insertion discovery. Bioinformatics. 2010;26 12:i350-i7. doi:10.1093/bioinformatics/btq216.

19. Ye K, Schulz MH, Long Q, Apweiler R and Ning Z. Pindel: a pattern growth approach to detect break points of large deletions and medium sized insertions from paired-end short reads. Bioinformatics. 2009;25 21:2865-71. doi:10.1093/bioinformatics/btp394.

20. Rausch T, Zichner T, Schlattl A, Stütz AM, Benes V and Korbel JO. DELLY: structural variant discovery by integrated paired-end and split-read analysis. Bioinformatics. 2012;28 18:i333-i9.

21. Layer RM, Chiang C, Quinlan AR and Hall IM. LUMPY: a probabilistic framework for structural variant discovery. Genome biology. 2014;15 6:R84.

22. Xie C and Tammi MT. CNV-seq, a new method to detect copy number variation using high-throughput sequencing. BMC bioinformatics. 2009;10 1:80.

23. Abyzov A, Urban AE, Snyder M and Gerstein M. CNVnator: an approach to discover, genotype, and characterize typical and atypical CNVs from family and population genome sequencing. Genome research. 2011;21 6:974-84.

24. Szatkiewicz JP, Wang W, Sullivan PF, Wang W and Sun W. Improving detection of copy-number variation by simultaneous bias correction and read-depth segmentation. Nucleic acids research. 2013;41 3:1519-32.

25. Claros MG, Bautista R, Guerrero-Fernández D, Benzerki H, Seoane P and Fernández-Pozo N. Why assembling plant genome sequences is so challenging. Biology. 2012;1 2:439-59.

26. Warr A, Hume D, Archibald AL, Deeb N and Watson M. Identification of low-confidence regions in the pig reference genome (Sscrofa10. 2). Frontiers in

genetics. 2015;6:338.

27. Kelley DR and Salzberg SL. Detection and correction of false segmental duplications caused by genome mis-assembly. Genome biology. 2010;11 3:1.

28. He D, Hormozdiari F, Furlotte N and Eskin E. Efficient algorithms for tandem copy number variation reconstruction in repeat-rich regions. Bioinformatics. 2011;27 11:1513-20.

29. Zimin AV, Kelley DR, Roberts M, Marçais G, Salzberg SL and Yorke JA. Mis-Assembled “Segmental Duplications” in Two Versions of the <italic>Bos taurus</italic> Genome. PLoS One. 2012;7 8:e42680. doi:10.1371/journal.pone.0042680.

30. Zarrei M, MacDonald JR, Merico D and Scherer SW. A copy number variation map of the human genome. Nature Reviews Genetics. 2015.

31. Consortium GotN. Whole-genome sequence variation, population structure and demographic history of the Dutch population. Nature genetics. 2014;46 8:818-25.

32. Badr Benjelloun FJA, Streeter I, Boyer F, Coissac E, Stucki S, BenBati M, et al. Characterizing neutral genomic diversity and selection signatures in indigenous populations of Moroccan goats (Capra hircus) using WGS data. Frontiers in genetics. 2015;6.

33. Dong Y, Zhang X, Xie M, Arefnezhad B, Wang Z, Wang W, et al. Reference genome of wild goat (capra aegagrus) and sequencing of goat breeds provide insight into genic basis of goat domestication. BMC genomics. 2015;16 1:431.

34. Dong Y, Xie M, Jiang Y, Xiao N, Du X, Zhang W, et al. Sequencing and

automated whole-genome optical mapping of the genome of a domestic goat (Capra hircus). Nature biotechnology. 2013;31 2:135-41.

35. Bickhart DM, Rosen BD, Koren S, Sayre BL, Hastie AR, Chan S, et al. Single-molecule sequencing and chromatin conformation capture enable de novo reference assembly of the domestic goat genome. Nature Genetics. 2017;49 4:643-50.

36. Diez CM, Meca E, Tenaillon MI and Gaut BS. Three groups of transposable elements with contrasting copy number dynamics and host responses in the maize (Zea mays ssp. mays) genome. PLoS Genet. 2014;10 4:e1004298.

37. Li H, Ruan J and Durbin R. Mapping short DNA sequencing reads and calling variants using mapping quality scores. Genome research. 2008;18 11:1851-8.

38. McKenna A, Hanna M, Banks E, Sivachenko A, Cibulskis K, Kernytsky A, et al. The Genome Analysis Toolkit: a MapReduce framework for analyzing next-generation DNA sequencing data. Genome Res. 2010;20 9:1297-303. doi:10.1101/gr.107524.110.

39. Li H, Handsaker B, Wysoker A, Fennell T, Ruan J, Homer N, et al. The Sequence Alignment/Map format and SAMtools. Bioinformatics. 2009;25 16:2078-9. doi:10.1093/bioinformatics/btp352.

40. Kent WJ. BLAT—the BLAST-like alignment tool. Genome research. 2002;12 4:656-64.

41. Pedregosa F, Varoquaux G, Gramfort A, Michel V, Thirion B, Grisel O, et al. Scikit-learn: Machine learning in Python. Journal of Machine Learning Research.

2011;12 Oct:2825-30.

42. Chain FJ, Feulner PG, Panchal M, Eizaguirre C, Samonte IE, Kalbe M, et al. Extensive copy-number variation of young genes across stickleback populations. PLoS Genet. 2014;10 12:e1004830. doi:10.1371/journal.pgen.1004830.

43. Quinlan AR and Hall IM. BEDTools: a flexible suite of utilities for comparing genomic features. Bioinformatics. 2010;26 6:841-2.

44. Zhang X, Xu Y, Liu D, Geng J, Chen S, Jiang Z, et al. A modified multiplex ligation-dependent probe amplification method for the detection of 22q11. 2 copy number variations in patients with congenital heart disease. BMC genomics. 2015;16 1:364.

45. Chaisson MJ and Tesler G. Mapping single molecule sequencing reads using basic local alignment with successive refinement (BLASR): application and theory. BMC bioinformatics. 2012;13 1:238.

46. Hach F, Hormozdiari F, Alkan C, Hormozdiari F, Birol I, Eichler EE, et al. mrsFAST: a cache-oblivious algorithm for short-read mapping. Nature methods. 2010;7 8:576-7.

47. Bickhart DM, Hou Y, Schroeder SG, Alkan C, Cardone MF, Matukumalli LK, et al. Copy number variation of individual cattle genomes using next-generation sequencing. Genome research. 2012;22 4:778-90.

48. Li H, Handsaker B, Wysoker A, Fennell T, Ruan J, Homer N, et al. The sequence alignment/map format and SAMtools. Bioinformatics. 2009;25 16:2078-9.

49. Zhang X, Wang K, Wang L, Yang Y, Ni Z, Xie X, et al. Genome-wide patterns of

copy number variation in the Chinese yak genome. BMC genomics. 2016;17 1:1.

50. Yi G, Qu L, Liu J, Yan Y, Xu G and Yang N. Genome-wide patterns of copy number variation in the diversified chicken genomes using next-generation sequencing. BMC genomics. 2014;15:962. doi:10.1186/1471-2164-15-962.

51. Li R, Li Y, Zheng H, Luo R, Zhu H, Li Q, et al. Building the sequence map of the human pan-genome. Nat Biotechnol. 2010;28 doi:10.1038/nbt.1596.

52. Monat C, Pera B, Ndjiondjop M-N, Sow M, Tranchant-Dubreuil C, Bastianelli L, et al. de novo assemblies of three Oryza glaberrima accessions provide first insights about pan-genome of African rices. Genome biology and evolution. 2016:evw253.

53. Li Y-h, Zhou G, Ma J, Jiang W, Jin L-g, Zhang Z, et al. De novo assembly of soybean wild relatives for pan-genome analysis of diversity and agronomic traits. Nature biotechnology. 2014;32 10:1045-52.

54. Jenkins GM, Goddard ME, Black MA, Brauning R, Auvray B, Dodds KG, et al. Copy number variants in the sheep genome detected using multiple approaches. BMC genomics. 2016;17 1:1.

55. Zhu C, Fan H, Yuan Z, Hu S, Ma X, Xuan J, et al. Genome-wide detection of CNVs in Chinese indigenous sheep with different types of tails using ovine high-density 600K SNP arrays. Scientific reports. 2016;6.

56. Wang, X; Zheng, Z; Cai, Y; Chen, T; Li, C; Fu, W; Jiang, Y (2017): Supporting data for "CNVcaller: High efficient and Widely Applicable Software for Detecting Copy Number Variations in large Populations" GigaScience Database. http://dx.doi.org/10.5524/100380

Figure1

Click here to download Figure Figure1.pdf

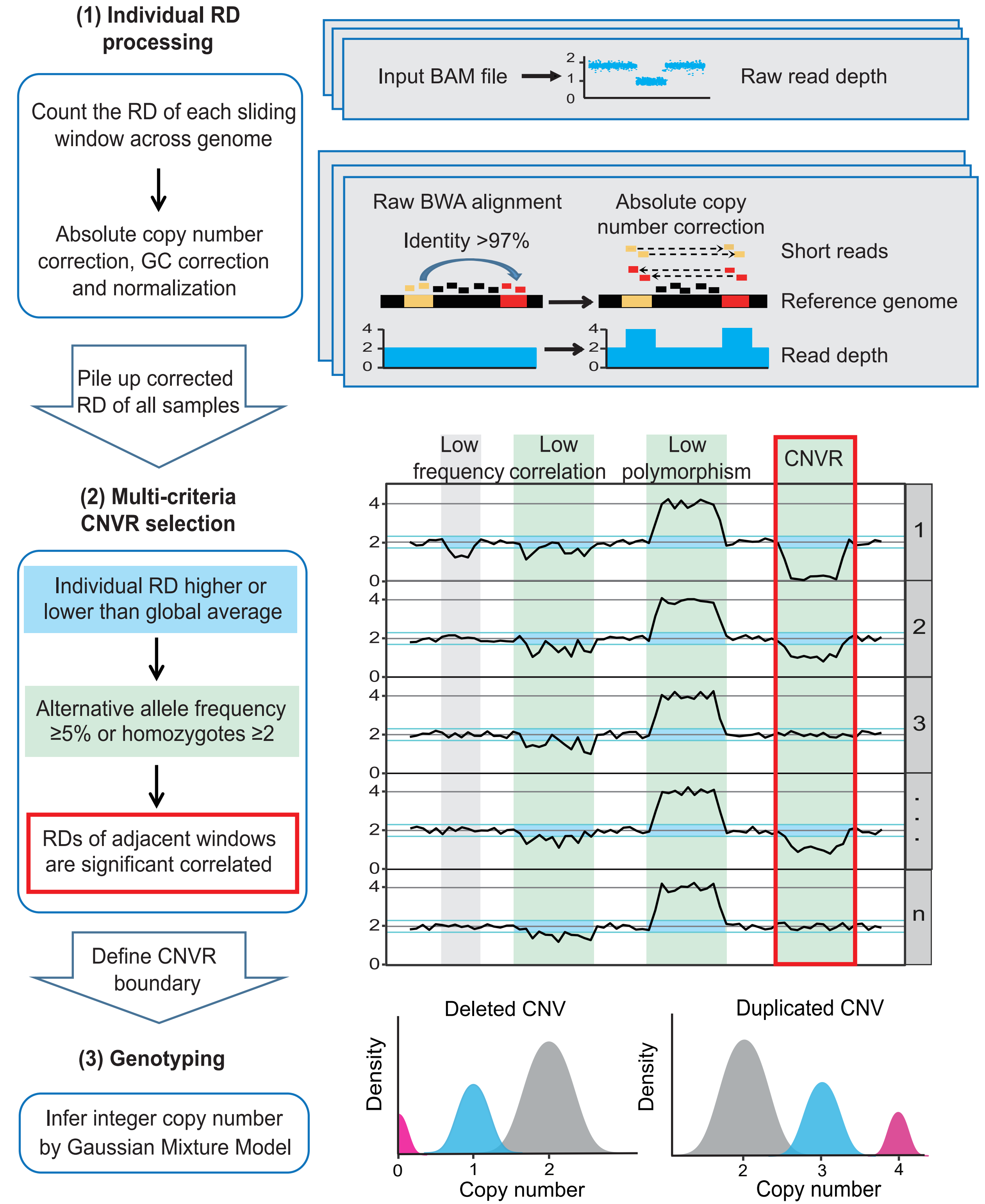

Figure2

Click here to download Figure Figure2.pdf

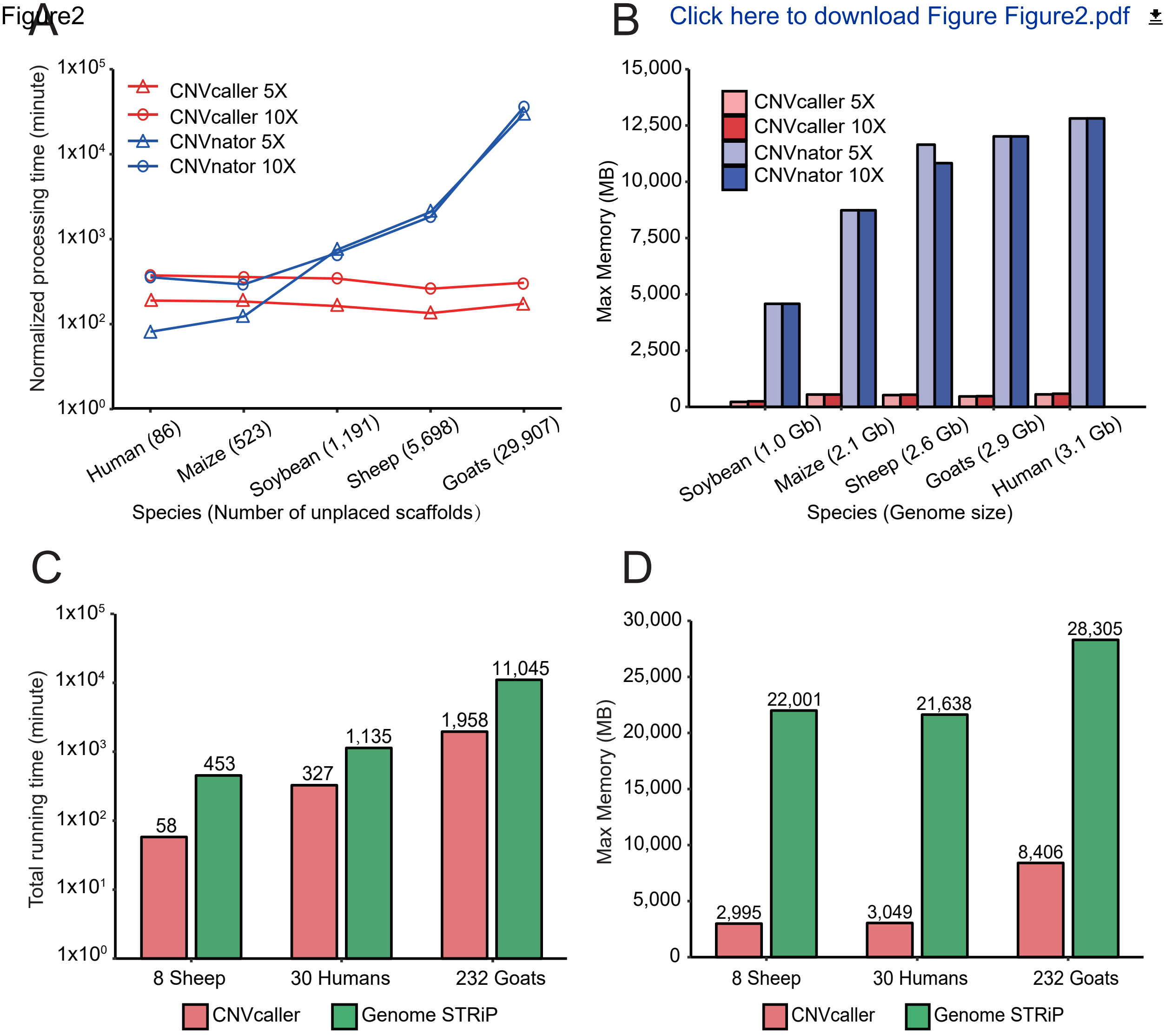

Figure3

Click here to download Figure figure3.new.pdf

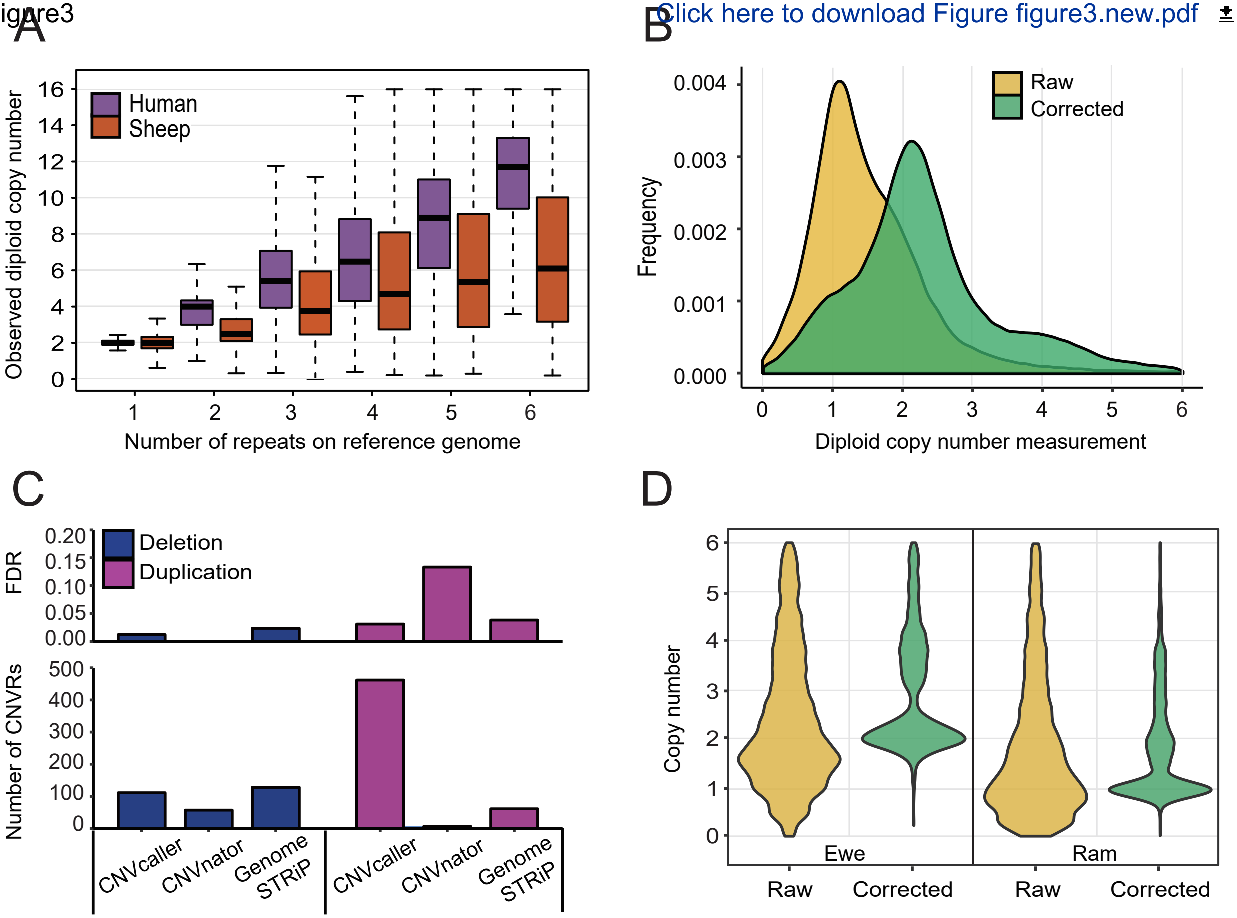

Figure4

Click here to download Figure figure4.new.pdf

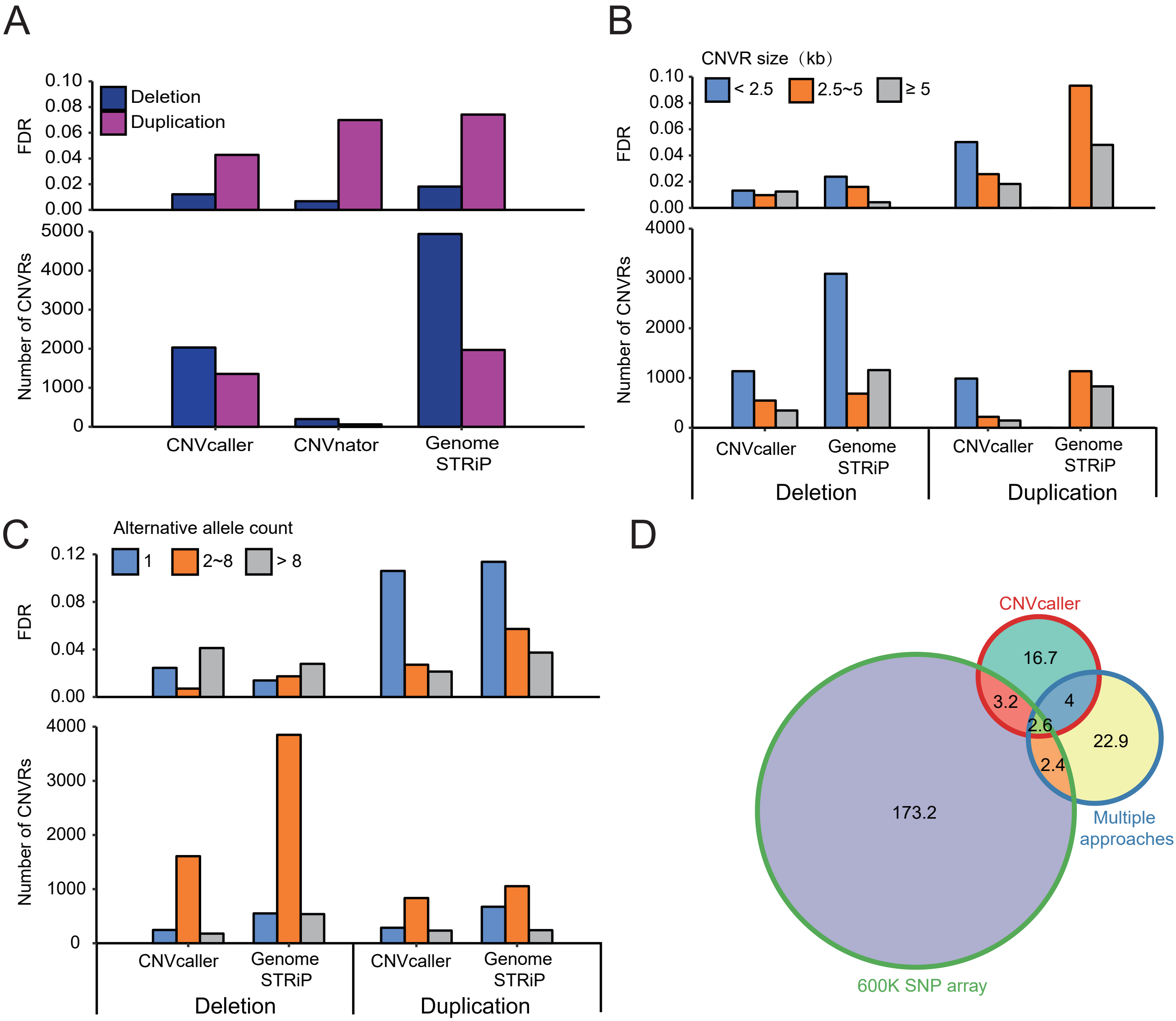

Figure5

Click here to download Figure Figure5.pdf

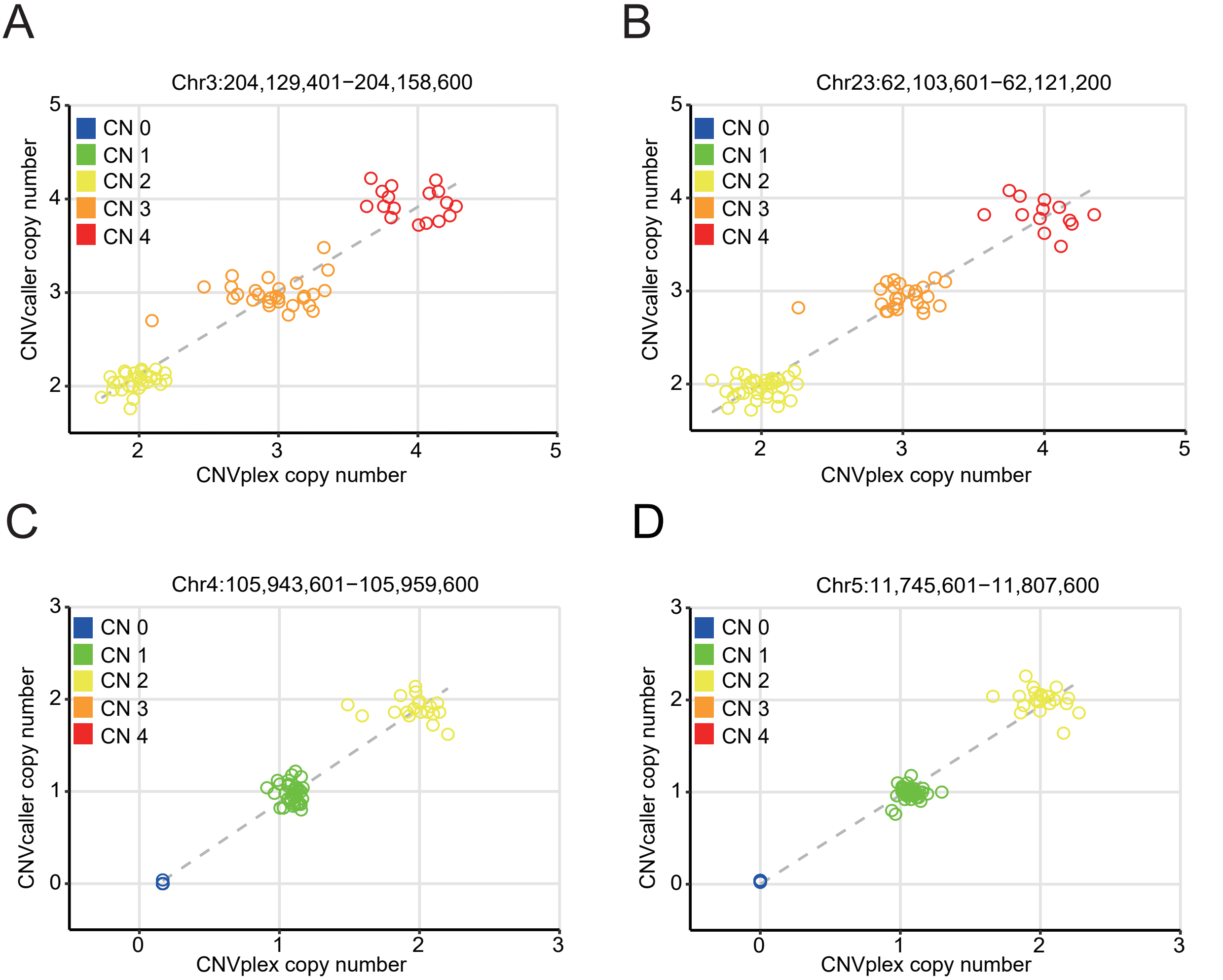

Figure6

Click here to download Figure figure6.new.pdf

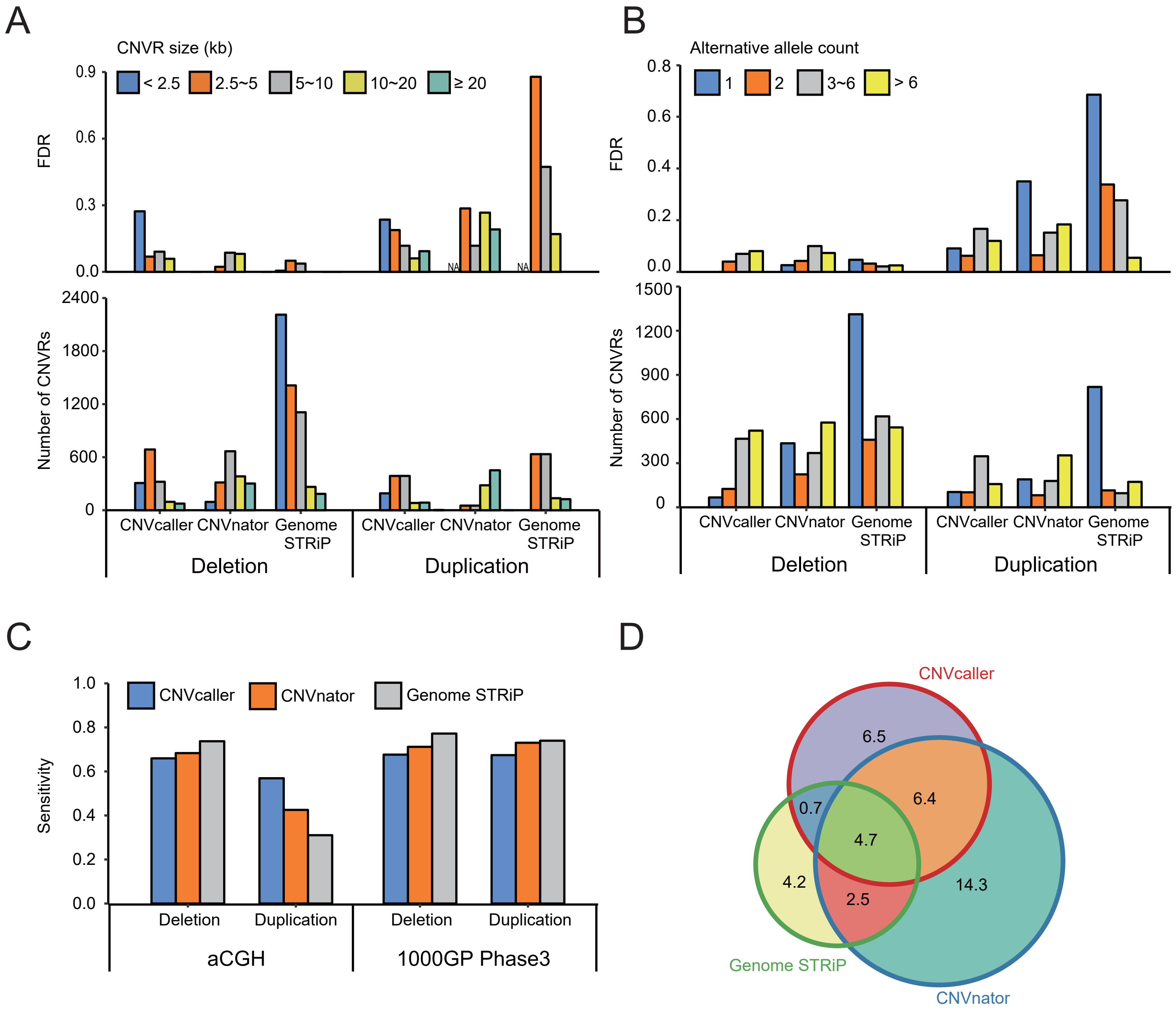

Click here to access/download
Supplementary Material
Supplementary Materials10_14.docx

GIGA-D-17-00119
CNVcaller: Highly Efficient and Widely Applicable Software for Detecting Copy Number Variations in Large Populations
Xihong Wang; Zhuqing Zheng; Yudong Cai; Ting Chen; Chao Li; Weiwei Fu; Yu Jiang
GigaScience

Dear Dr. Edmunds,

Thank you very much for handling our manuscript "CNVcaller: Highly Efficient and Widely Applicable Software for Detecting Copy Number Variations in Large Populations " (GIGA-D-17-00119). We appreciate all the comments from the reviewers, which helped us improve our manuscript. We have now revised the manuscript according to the reviewers' comments and your instructions.

We addressed the comments and questions of the reviewers as explained below; the reviewers' text has been included, and our responses are in coloured italics. The revised text is indicated by quotation marks. Because several new figures have been added, we have attached a list of the current figures and tables corresponding to those in the last version so that the changes can be easily tracked.

According to the suggestions of the reviewers, we have modified the manuscript as follows:

1. *The newly released version of CNVcaller updated the genotyping method. A python package, scikit-learn v0.19.0, was used to decompose the reported copy numbers into several Gaussian distributions. Therefore, the accuracy of CNVcaller in the new version was increased.*

2. *The reviewers requested that we evaluate the effects of the length and allele frequency of the discovered CNVRs. Therefore, two sections have been added to the results analysing the number and FDR of the CNVRs detected by the three methods against the length and allele frequency. One section (including Figure 4) was based on the sheep data, the other section (including Figure 6) was based on the human data.*

3. *To answer the reviewer's question about the difference between the FDRs in deletions and duplications, their FDRs were evaluated separately in the results section.*

4. *The high proportion of misassembled segmental duplications in non-human assemblies may have led to misunderstanding on the reviewer's part. This section has been extensively redrafted with analyses of both real and simulated data.*

5. *The previous discussion section has been merged with the results section to reduce the length of the manuscript. The first part of the previous results section has been moved to the methods section, as suggested by the reviewer.*

6. *The language has been professionally edited by an English-language editing service, American Journal Experts (AJE).*

7. *We have registered the software in the SciCrunch.org database. The RRID SRC_015752 was added to the 'Availability and requirements' sections.*

Thank you again for all of your assistance.

Sincerely yours,

Yu Jiang and co-authors

****

REVIEWS

****

Reviewer #1:

The authors developed a new CNV caller pipeline which they called CNVcaller geared towards improved speed compared to existing CNV callers and improved accuracy for high complexity genomes. I commend the authors on their efforts to introduce improved algorithms and pipelines for an inherently difficult procedure, namely CNV calling. My comments are mostly suggestions for improvement as follows. Note, comments of the form (4:5 for example represent page 4, line 5).

Thank you for your positive comments and encouragement. We have substantially revised the manuscript upon your suggestions.

There are several grammatical errors which make the paper somewhat confusing. I would strongly recommend further extensive English editing.

We apologize for these mistakes. The manuscript has been professionally edited by an English-language editing service, American Journal Experts (AJE).

My main criticism of the analysis is one that I have seen repeatedly of most other CNV calling publications, and that is there is no sensitivity analysis.

We are sorry for the ambiguity of the sensitivity tests, which were included in the previous Table 1. In the revised manuscript, Figure 6C has been added to describe the sensitivity. In general, CNVcaller demonstrated 57%-67% sensitivity for duplications and 66%-68% for deletions in human data. The sensitivity in sheep was ~73% by indirectly evaluation. The detailed descriptions are as follows:

Human: "The sensitivity was estimated as the proportion of the high-confidence

CNVR database that overlapped with the predicted CNVRs. Two previously published high-confidence databases that include our test samples are the aCGH-based CNVR database [1] and the 1000GP CNVR map [2]. For the aCGH database, CNVcaller demonstrated the highest sensitivity (57%) in duplications, whereas Genome STRiP achieved the highest sensitivity (74%) in deletions (Figure 6C). Both Genome STRiP and CNVnator were the core contributors to the 1000GP CNV maps; However, the sensitivity of CNVcaller was 68% and 67% for deletions and duplications according to this database, only 4%-10% lower than Genome STRiP and CNVnator."

Sheep: "The sensitivity of sheep CNVRs was estimated indirectly due to the lack of a validated database. Based on our integrated analysis (see methods), there were 138 sheep X chromosome-origin scaffolds, which were not anchored onto chromosomes of OAR v3.1. Therefore, all of these scaffolds should be detected as CNVs because the rams had half the copy numbers of the ewes. As a result, CNVcaller detected 101 of these 138 X-origin scaffolds, with a sensitivity of 73%. In contrast, CNVnator and Genome STRiP did not report these unmapped CNVRs."

The authors here also suggest various parameters throughout their paper for performing CNV calling, but there is no analysis of how the results change if these parameters are adjusted, i.e. no analysis of how robust your algorithm is to changes in the parameters.

Thank you for your suggestion. Two important parameters of CNVcaller were window size and minimum report allele frequency. The FDRs against the window size and alternative allele frequency have been added to Supplementary Figure 1 and Figure 6B. In general, with the increasing of window size and allele frequency, the accuracy raised while the sensitivity decreased.

As another example, Hong et al 27503473 has demonstrated that the biggest variability in calling CNVs is in terms of the CNV size. I suspect that the same can be said of CNVcaller. Please comment on what sizes of CNVs does CNV caller do well or poorly on.

Thank you for your suggestion. Figure 4 and Figure 6 have been added, which evaluate the effects of length and frequency in sheep and human data. The detailed

comparisons in the manuscript are as follows:

Sheep: "The accuracy was evaluated by the Mendelian inconsistency of all the CNVRs on autosomes against the length and alternative allele frequency (Figure 4). CNVcaller achieved higher accuracy than Genome STRiP in both deletion (1% vs 2%) and duplication (4% vs 7%) (Figure 4A). Whereas Genome STRiP had greater capability to detected short (<2.5 kb) deletions (Figure 4B), indicating the RP methods integrated in Genome STRiP performed well on small deletions. Concerning the alternative allele frequency, both methods showed an increased FDR in rare duplications (Figure 4C). However, CNVcaller is primarily used to detect CNVRs related to economic traits in livestock and crops. In these studies, the target CNVRs usually have a high frequency after long-duration breeding selection."

Human: "CNVcaller demonstrated the highest overall accuracy for detecting duplications and performed consistently across the length and frequency categories, whereas Genome STRiP and CNVnator had high FDRs on the short or singleton duplications (Figure 6A, B). Genome STRiP showed the greatest ability to detect deletions, indicating the advantage of combining RD and RP methods for deletion detection. The genotyping accuracy of the human dataset was further benchmarked against the high-confidence aCGH array-based database. The discordance rates of CNVcaller, CNVnator and Genome STRiP were 2.6%, 5.5% and 2.2%, respectively. This genotyping accuracy ranking was same with the Mendelian inconsistency of the 10 Dutch trios (Supplementary Figure 5)."

2:32 "the prevalent.." is a gross exaggeration. I think you mean "a prevalent".

This has been corrected as suggested.

2:35 I don't think you mean geometric. I did not comment on other grammatical/English errors as there were too many to list individually. I would highly recommend getting help with the English in this paper.

We apologize for these mistakes. The manuscript has been professionally edited by an English-language editing service, American Journal Experts (AJE).

3:53 "RD" is not defined.

We apologize for the missing definition. The following description has been added to the introduction:

"read depth (RD) refers to the depth of coverage in a genomic region that can be calculated from the number of aligned reads [14], a CNV region should have a higher or lower RD than expected [22-24]."

6:120 Give a brief description of how CNVnator handles GC bias. Also why 40% for the GC bias? Shouldn't this parameter be dependent on the organism of interest?

We apologize for not clearly describing the procedure. In general, the mean RD of windows with 40% percent GC is used as only a temporary standard in the GC correction step. It will be lost in the following normalization step, in which the GC-corrected RDs of each window are divided by the global median RDs. Because the denominator is calculated from the RDs already corrected by the 40% GC windows, this parameter will be lost and is not necessarily dependent on the organism of interest.

The GC corrected RD for a window is calculated by CNVnator as follows: the raw RD times the global average RD and divided by the average RD with the same GC content as in this window. Because the global average RD is calculated before the GC correction, no temporary parameter is used. The equation is as follows:

$$RD^i_{corrected} = \frac{\overline{RD}_{global}}{\overline{RD}_{gc}} RD^i_{raw},$$

where i is the bin index, RD^i_{raw} *is the raw RD signal for a bin,* $RD^i_{corrected}$ *is the corrected RD signal for the bin,* $\overline{RD}_{global}$ *is the average RD signal over all bins, and* $\overline{RD}_{gc}$ *is the average RD signal over all bins with the same GC content as the bin.*

The commentary on certain genomes not being as complete as others is important. I

suspect though that if a large percentage of the samples show a CNV in a genome that is newer or not as complete, then this observation may be more likely indicative of a problem with the reference. Can you comment?

If the detected CNVR has variation in a population, which means the read depths can be clustered into two or more normal distributions, this CNVR is probably true even with high frequency. In contrast, if all of the individuals show the same abnormal read depth, this suggests that the reference individual is different from the sample population or some assembly problems exist.

7:145 I am not convinced Pearson's correlation is appropriate. Your data is likely to have outliers and non-normal data. A non-parametric test of correlation like Spearman's correlation (Kendall-Tau is likely too computational intensive), or performing correlation after 5 or 10% trimming may be more appropriate.

We tried replacing Pearson's correlation with Spearman's correlation in the 30 BAM files from the 1000 Genome Projects data. However, the FDR doubled after the replacement, while the length of each call was reduced by half. A possible reason is that Spearman's correlation is calculated by ranking instead of the numerical value of copy numbers across samples. Therefore, the divergent copy numbers of individuals with deletions or duplications contributed no more than the subtle random mistakes of normal copy individuals, especially in the low-frequency CNVRs.

Trimming is also not recommended for a similar reason. In the low-frequency CNVRs, individuals with an abnormal copy number will be trimmed as outliers.

cn.MOPS (Klambauer et al, PMID: 22302147) uses a mixture of Poissons as opposed to Gaussian Mixture Models for CNV detection. I suspect the mixture of Poissions will be superior to Gaussian Mixture Models when the read depths are low, and Gausssian mixtures may be more appropriate when read depths are high. How difficult is it to replace the Gaussian mixtures with Poisson mixtures and compare the performance? I feel that this analysis would be informative and potentially improve your algorithm.

Thank you for your suggestion. However, it is not easy to replace the distribution because the RDs after GC correction and normalization are not integers; thus, they

cannot be directly treated as Poisson distributions. Additionally, we totally agree with your comment about the Poisson distribution will be superior for low read depths with high STDEV. However, the currently common sequencing depth are about 5-10X. Under this sequencing depth and a proper window size, the STDEV/mean RD was only 0.2-0.3, which essentially not fit the Poisson distribution. In addition, we used the RDs of 232 goats with ~10X coverage to test the fitness of Gaussian distribution using the omnibus test (scipy 0.19.0). As a result, 88% of windows accepted the null hypothesis at the P = 0.01 level. Therefore, we believe the Gaussian Mixture Model is acceptable for the current data.

The term "CNVR" is critical for understanding the algorithm, and requires more explanation of the term.

We apologize for missing this important concept. The following explanation has been added to the introduction:

"To study the polymorphism among individuals, the overlapping CNVs need to be merged into unified regions, namely CNV regions (CNVRs)"

It would be helpful to include some further discussion on where you see that CNVcaller works better or worse than existing CNV calling software.

Thank you for your suggestion. Figure 2 shows that the speed of CNVcaller was one to two orders of magnitude higher than the other methods. Newly added Figure 4 and Figure 6 evaluated the effects of length and frequency in sheep and human data. In general, the performance of CNVcaller was better for all sizes of duplications but was poor for deletions <2.5 kb.

9:180. The "arbitrary standards" require a citation.

Two citations have been added as follows:

1. *Chain FJ, Feulner PG, Panchal M, Eizaguirre C, Samonte IE, Kalbe M, et al. Extensive copy-number variation of young genes across stickleback populations. PLoS genetics. 2014;10 12:e1004830.*

2. *Abyzov A, Urban AE, Snyder M and Gerstein M. CNVnator: an approach to discover, genotype, and characterize typical and atypical CNVs from family and*

population genome sequencing. Genome research. 2011;21 6:974-84.

Minor comment: Since speed seems to be a major selling point of the software, more details about running the software on a compute cluster or running algorithms in parallel in the documentation would be helpful.

A new section, "Parallel submission of individual RD processing," has been added to the methods with the principle and commands as follows:

"Parallel processing of individual RDs. The CNVcaller processes the BAM file of each individual separately in the first step, and therefore, parallel computations can be performed to reduce the total running time. All BAM files are equally distributed into N groups, and each group contains M files. The max N is the total available processing cores, and M is the total number of BAM files/N. For example, the 232 goat BAM files were processed on a node with 32 processing cores and 124 GB of RAM. We distributed the 232 files into 20 groups, and each group contained 12 BAM files. The shell command for one group is as follows:

```
#!/bin/sh

for i in {1..M}

  do bash Individual.Process.sh -b $i.bam -h $i -d dup -s sex_chromosome

done
```

After corrections and normalization, the comparable RDs of each sample are aggregated into an ~100 MB intermediate file and output, thus preventing repeated calculations for the same individual in different populations."

****

Reviewer #2:

The proposed method "CNVcaller" enables the efficient discovery and genotyping of CNVs in large populations. One of the main benefits of the method is that it can handle draft genome assemblies with thousands of scaffolds. The computational benchmarks proof that the method is fast and memory efficient but the evaluation of the accuracy of the method is less convincing. Some details of the method remain vague and hinder an objective evaluation. Detailed comments of how to improve the manuscript are below:

Thank you for your affirmation. We are sorry for the ambiguity of the accuracy test and have substantially revised the manuscript according to your suggestions. In the revised manuscript, the performance evaluation in the previous Table 1 is described in more detail in Figure 4 and Figure 6.

Comment 1 - The primary application of CNVcaller is the detection of CNVs in large populations. Population variant call sets are dominated by rare variants of rather small size. For instance, less than 20% of the 1000 Genomes structural variants have a population allele frequency >5% and almost 50% of the SVs are <2kbp in size despite the rather low coverage (~7x). CNVcaller is currently restricted to large CNVs (>2kbp) and common variants (>5% allele frequency), which is a major limitation for population genomic studies.

We apologise for the ambiguous. Actually, the user can retain all the windows with at least one individual that shows heterozygous deletion or duplication. However, we recommend removing low-frequency windows in large populations with low sequencing coverage because of increased random mistakes. In the revised version, Figure 6 was added to evaluate the effects of length and frequency by IRS test. We found Genome STRiP showed the greatest ability to detect short and rare deletions, indicating the advantage of combining RD and RP methods for deletion detection. However, short and rare duplications still had extremely high FDR. The shortest duplications reported by CNVnator and Genome STRiP were 2.8 kb and 2.5 kb, and the IRS FDRs of 2.5-5 kb calls were 29% and 88%, respectively. The FDRs of singletons were 35% and 69% for CNVnator and Genome STRiP, respectively. The main improvement of CNVcaller is the accuracy of duplications. The FDR of 2.5 kb – 5 kb duplications was reduced to 19%, and the FDR of singleton duplications were reduced to 9%. However, the FDRs were still higher than those of the longer and higher-frequency calls. So, these calls were removed from the previous manuscript.

These uncertain calls were also removed by the phase 3 extended SV release of 1000GP. After extra quality controls, the number of duplications in the released database is only 1/7 the number of deletions, and the median size is 36 kb, which is 17 times longer than deletions. Therefore, improving the accuracy of duplications on this foundation is meaningful for enriching the CNV database.

Additionally, the current main use of CNVcaller is the detection of CNVRs related to economic traits in livestock and crops. In these populations, the target CNVRs usually have a medium or high frequency after long-duration artificial selection. We believe that the high-confidence medium to high frequency reported by CNVcaller can contribute to functional and breeding studies of animals and plants.

The sensitivity increase of CNVcaller for the subset of common and large CNVs seems to be driven by an increased number of detected CNVs in SD regions (Figure 5C). SNP arrays have a low SNP density in SD regions and in the present Manuscript array SNP probes in SD regions have been removed entirely. The reported IRS FDR is therefore heavily biased against CNVs in SD regions and it thus seems mandatory to me to proof that this sensitivity increase for SD-associated CNVs is not leading to an inflated FDR.

Thank you for your suggestions. Figure 5C (new Figure 3C) has been updated to show both the number and the Mendelian inconsistency of the detected CNVs in SDs. The Mendelian inconsistency rate of the calls in SD regions made by CNVcaller was approximately 3%, no higher the other methods. The copy numbers of unique and SDs were also indirectly validated by the X-origin scaffolds of a 133-sheep population. All of these scaffolds should be detected as CNVs because the rams had half the copy numbers of the ewes. As a result, CNVcaller detected 101 of these 138 X-origin scaffolds. In contrast, CNVnator and Genome STRiP did not report these regions.

The Manuscript lacks a Figure that shows the size and allele frequency distribution of the discovered CNVs in comparison to Genome STRiP and CNVnator. An estimate of breakpoint accuracy of CNVcaller would also be valuable.

Thank you for your suggestion. Figure 4 and Figure 6 have been added, which evaluate the effects of length and frequency in sheep and human data. The detailed comparisons in the manuscript are as follows:

Sheep: "The accuracy was evaluated by the Mendelian inconsistency of all the CNVRs on autosomes against the length and alternative allele frequency (Figure 4). CNVcaller achieved higher accuracy than Genome STRiP in both deletion (1% vs

2%) and duplication (4% vs 7%) (Figure 4A). Whereas Genome STRiP had greater capability to detected short (<2.5 kb) deletions (Figure 4B), indicating the RP methods integrated in Genome STRiP performed well on small deletions. Concerning the alternative allele frequency, both methods showed an increased FDR in rare duplications (Figure 4C). However, CNVcaller is primarily used to detect CNVRs related to economic traits in livestock and crops. In these studies, the target CNVRs usually have a high frequency after long-duration breeding selection."

Human: "CNVcaller demonstrated the highest overall accuracy for detecting duplications and performed consistently across the length and frequency categories, whereas Genome STRiP and CNVnator had high FDRs on the short or singleton duplications (Figure 6A, B). Genome STRiP showed the greatest ability to detect deletions, indicating the advantage of combining RD and RP methods for deletion detection. The genotyping accuracy of the human dataset was further benchmarked against the high-confidence aCGH array-based database. The discordance rates of CNVcaller, CNVnator and Genome STRiP were 2.6%, 5.5% and 2.2%, respectively. This genotyping accuracy ranking was the same with the Mendelian consistency of the 10 Dutch trios (Supplementary Figure 5)."

Thank you for your reminding of the breakpoint issue. However, unlike the PR/SP algorithm, RD can not detect breakpoints in the at base pair resolution or less than the window step size resolution. Integrating RD and RP methods can improve the breakpoint accuracy in human genome. However, precise breakpoint is more difficult to achieve in the poorly assembled genomes. Additionally, the breakpoint issue did not affect the genotyping accuracy which is the direct input of GWAS. The genotyping FDR of CNVcaller, CNVnator and Genome STRiP were 2.6%, 5.5% and 2.2%, respectively.

The Manuscript mentions mrsFAST for absolute copy number validation. I could not find any formal comparison of predicted copy-number by mrsFAST and CNVcaller but maybe I missed this?

Supplementary Figure 2 (previous Supplementary Figure 1) shows that the copy numbers calculated using mrsFAST and CNVcaller were similar. However, mrsFAST needed to realign all the multi-hit reads in BWA alignments, leading to significantly increased computational time. For example, mrsFAST required 10 hours for a 3G genome with 10X sequencing data, whereas CNVcaller needed only 4 minutes.

- Please add to Table 1 the number of CNV sites that could be assessed by the IRS method and what proportion of each call set could be evaluated using IRS. I also believe the IRS method reports p-values separately for deletions, duplications and multi-allelic CNVs. Was there any difference among these for CNVcaller?

Detailed information on 1000GP calls, including the required information, has been

added to Supplementary Table 5. Overall, 28%, 30% and 60% of the CNVRs of CNVcaller, CNVnator and Genome STRiP covered at least one probe of the Affymetrix SNP 6.0 array and therefore could be assessed using the IRS test. One main reason for the divergent testable proportions was that only 4% of Genome STRiP calls overlapped with SDs, which have infrequent probes, whereas 34% of the CNVcaller calls and 28% of the CNVnator calls overlapped with SDs.

Two extra genome-wide evaluations can provide supplemental evidence. The Mendelian inconsistency of 10 Dutch families was added to Supplementary Figure 5, which was based on tests of both unique and SD regions. The inconsistency rates of CNVcaller, CNVnator and Genome STRiP were 1.5%, 4.4%, and 0.4%, respectively. This accuracy ranking was consistent with the genotyping discordance values compared with the aCGH database, which were 2.6%, 5.5% and 2.2% for CNVcaller, CNVnator and Genome STRiP, respectively.

To analysis the difference between deletions and duplications, all FDRs were evaluated separately in the revised manuscript. We found the duplications had much higher FDRs than the deletions, especially for the short and rare CNVs.

- Some details of the method are vaguely specified and some Figures lack clarity and units.

Page 6, line 129: "... if the median RD of the homogametic sex chromosomes is about half of the median RD of autosome..."

This section has been expanded in the newly added subsection "RD corrections for sex chromosomes" as follows:

"RD corrections for sex chromosomes. Most mammalian and avian genomes show an XX/XY-type or ZZ/ZW-type sex-determining system. Their homogametic sex chromosomes (X or Z) constitute 5%-10% of the total genome and show half the RD of the autosomes in XY or ZW individuals. Therefore, intensive correction for X and Z chromosomes is needed. The RD of the X or Z chromosome (the particular name provided by the user) is used to determine the sex of a particular individual. If the median RD of this chromosome is <0.6X the median RD of the autosome, the individual is considered an XY or ZW type, and the RDs of this chromosome are doubled before normalization. Otherwise, nothing is performed for individuals determined to be XX or ZZ type."

Page 8, line 154: "... and the distance between them is less than a certain percent of their own length."

This text has been modified as follows: "As CNVRs can be separated by gaps or poorly assembled regions, the adjacent initial calls are merged if their RDs are highly correlated. The default parameters are as follows: the distance between the two initial calls is less than 20% of their combined length, and the Pearson's correlation index of the two CNVRs is significant at the P = 0.01 level."

Page 5, line 91: "The reference genome is segmented into overlapping sliding windows." What window size and overlap was used for high-coverage genomes?

The following description has been added to the methods.

"The window size is an important parameter for RD methods. CNVcaller uses half of the window size as the step size. The optimal window size is 800 bp (with a 400 bp overlap) for 5-10X coverage human and livestock sequencing data (Supplementary Figure 1). The recommended window sizes are inversely related with coverage, and thus, ~400 bp windows correspond to 20X coverage, and ~200 bp windows correspond to 50X coverage."

Page 5, line 95: "The raw RD signal is calculated for each window as the number of placed reads with centers within window boundaries." Does this imply that for paired-end data both reads are counted?

We apologise for the ambiguous description. The following description has been added: "Considering the uncontrollable effect of gap ratios from different genome assemblies, all of the end reads located in the window are independently added to the RD of this window, regardless of whether the read is from single-end mapping or paired mapping."

Page 8, line 154: "Then the two adjacent initial calls are further merged if their copy numbers are highly correlated". What threshold was used?

This text has been modified as follows: "As CNVRs can be separated by gaps or poorly assembled regions, the adjacent initial calls are merged if their RDs are highly correlated. The default parameters are as follows: the distance between the two initial calls is less than 20% of their combined length, and the Pearson's correlation index of the two CNVRs is significant at the P = 0.01 level."

Figure 3A: CNVcaller 13.7. What is the unit? Are these 13,700 CNVs?

The unit in this figure is Mb. Because the intersection of the three methods with different boundaries was difficult to define in numbers, they were evaluated in terms of length. CNVcaller covered 40% of the CNVRs detected by CNVnator, 45% of the CNVRs detected by Genome STRiP and 65% of their intersecting CNVRs, in terms of length.

Minor:

- I could not find a reference to the 232 goat sequencing data? Is this data publicly available?

Among the 232 goat whole-genome sequencing data files, 103 files were acquired from NCBI, the accession numbers are provided in Supplementary Table 1. The remaining 129 samples without accession numbers were generated by ourselves, and will be published soon. The reference and unpublished paper are as follows:

1.Badr Benjelloun FJA, Streeter I, Boyer F, Coissac E, Stucki S, et al. (2015) Characterizing neutral genomic diversity and selection signatures in indigenous populations of Moroccan goats (Capra hircus) using WGS data. Frontiers in genetics 6.

2.Dong Y, Zhang X, Xie M, Arefnezhad B, Wang Z, et al. (2015) Reference genome of wild goat (capra aegagrus) and sequencing of goat breeds provide insight into genic basis of goat domestication. BMC genomics 16: 431.

3.Dong Y, Xie M, Jiang Y, Xiao N, Du X, et al. (2013) Sequencing and automated whole-genome optical mapping of the genome of a domestic goat (Capra hircus). Nature biotechnology 31: 135-141.

4.Bickhart DM, Rosen BD, Koren S, Sayre BL, Hastie AR, et al. (2017) Single-molecule sequencing and chromatin conformation capture enable de novo reference assembly of the domestic goat genome. Nature Genetics 49: 643-650.

5.Wang XL, Liu J, Niu YY, Li Y, Zhou SW, et al. Low incidence of SNVs and indels in trio genomes of Cas9-mediated multiplex edited sheep. BMC Genomics. Under review.

6.Zheng ZQ, Li M, Liu J, Wang XL, Pan XY, et al. The early domestication process inferred from genome analysis of worldwide goats. In preparation.

- The first Results section "Overview of CNVcaller algorithm" seems better suited for

the Methods part.
This has been modified as suggested.

- Is the Mendelian consistency higher for the high-coverage trio: NA12878, her father (NA12891) and her mother (NA12892)?

Yes. In the high-coverage data of all three members of the trio (NA12891, NA12892 and NA12878 were all 50X), the inconsistency rate was 2.4%. In the high-coverage data of the parents (50X for NA12891 and NA12892) and the low-coverage data of the child (5.3X for NA12878), the inconsistency rate was 6.1%. Thus, increased sequencing depth can help to reduce the number of false positives.

- I believe the claim that read-pair/split-read algorithms are less powerful on draft assemblies of non-model organisms compared to read-depth methods is potentially true but the Manuscript lacks a proof for this or a citation that supports this claim.

Thank you for your agreement. This problem was found in our previous reference genome assembly projects for both sheep and goats. However, we did not report this result in the section on CNV/SD detection. The review listed below has some comments about this claim, however, without direct supporting data. Therefore, we have removed this comment from this manuscript.

Bickhart DM and Liu GE. The challenges and importance of structural variation detection in livestock. Frontiers in genetics. 2014;5.

"While RP methods should provide a suitable means for detecting such events in theory, two major problems currently challenge the accuracy of this method: (1) alignment errors resulting from the mapping of read pairs to repetitive regions of the genome…… The first problem (1) is unfortunately dependent on the reference genome assembly for the species, and is unlikely to be resolved until better reference assemblies are created for livestock."

- It is not clear from the Manuscript if CNVcaller reports copy-number likelihoods based on the Gaussian mixture model. Please clarify.
Thank you for your suggestion. CNVcaller reports the silhouette coefficients of the copy numbers instead of the Gaussian mixture model likelihood as quality control because we found that silhouette coefficients had a greater correlation with the IRS test results than likelihood.

- Figure 5A: Why is the absolute copy-number correction different for Human and Sheep?

We are sorry for not clearly interpreting the high proportion of misassembled segmental duplications in non-human assemblies. This part of the manuscript has been modified as follows:

"Previous studies have shown that a high proportion of SDs in animal genomes are misassembled single-copy regions [27, 29]. Therefore, we detected the ratios of false SDs on the human (hg19) and sheep (OAR v3.1) reference genome assemblies by the sequencing copy number of a human (NA12878) and a Tan sheep sample (Figure 3A). If the SDs were correctly assembled, the sequencing diploid copy number should be twice the copy number of SDs. For example, the average sequencing copy number of the two-copy SDs was four in NA12878. However, the corresponding sequencing copy number in sheep was only 2.4. These results indicated that most two-copy SDs of hg19 were truly duplicated in NA12878, while approximately 80% of the two-copy SDs in OAR v3.1 were single-copy regions in the Tan sheep sample. Thus, the SDs in the sheep genome were called "putative SDs" before validation."

- There is quite a few typing and grammatical errors. For instance:
*Figure 2B: Max mamory
*Supplementary Table 3: Memery
*Page 3, line 53: ...the number of reads aligned to of a particular region.
*Page 8, line 160: This model presets the average copy number of homozygous deletion, heterozygous deletion, normal, heterozygous deletion (duplication!), homozygous deletion (duplication!) at zero to four respectively.

We are sorry for these mistakes. We have proofread the revised manuscript and used a professional English-language editing service to minimize the grammatical errors.

Checklist of the updated tables and figures

Current version	Last version
Fig. 3	Fig. 5
Fig. 4A-C	Table 1 and newly added
Fig. 4D	Fig. 3B
Fig. 5	Fig. 4

Fig. 6A-C	Table 1 and newly added
Fig. 6D	Fig. 3A
Supplementary Fig. 1	Newly added
Supplementary Fig. 2	Supplementary Fig. 1
Supplementary Fig. 3	Newly added
Supplementary Fig. 4	Supplementary Fig. 2
Supplementary Fig. 5	Table 1 and newly added
Supplementary Table 4	Newly added
Supplementary Table 5	Newly added
