# Supplementary material for: CNVcaller: highly efficient and widely applicable software for detecting copy number variations in large populations
Source: Gigascience. 2017 Dec 4;6(12):1–12. doi: 10.1093/gigascience/gix115 (PMC5751039; doi:10.1093/gigascience/gix115)
Supplement: Supplemental material [file gix115_supp.docx]

**Supplementary Materials**

**Supplementary Figure 1** Number of CNVRs (A) and corresponding IRS FDR (B) were plotted against window size. 30 human BAM files of 1000GP Phase3 were used as input.


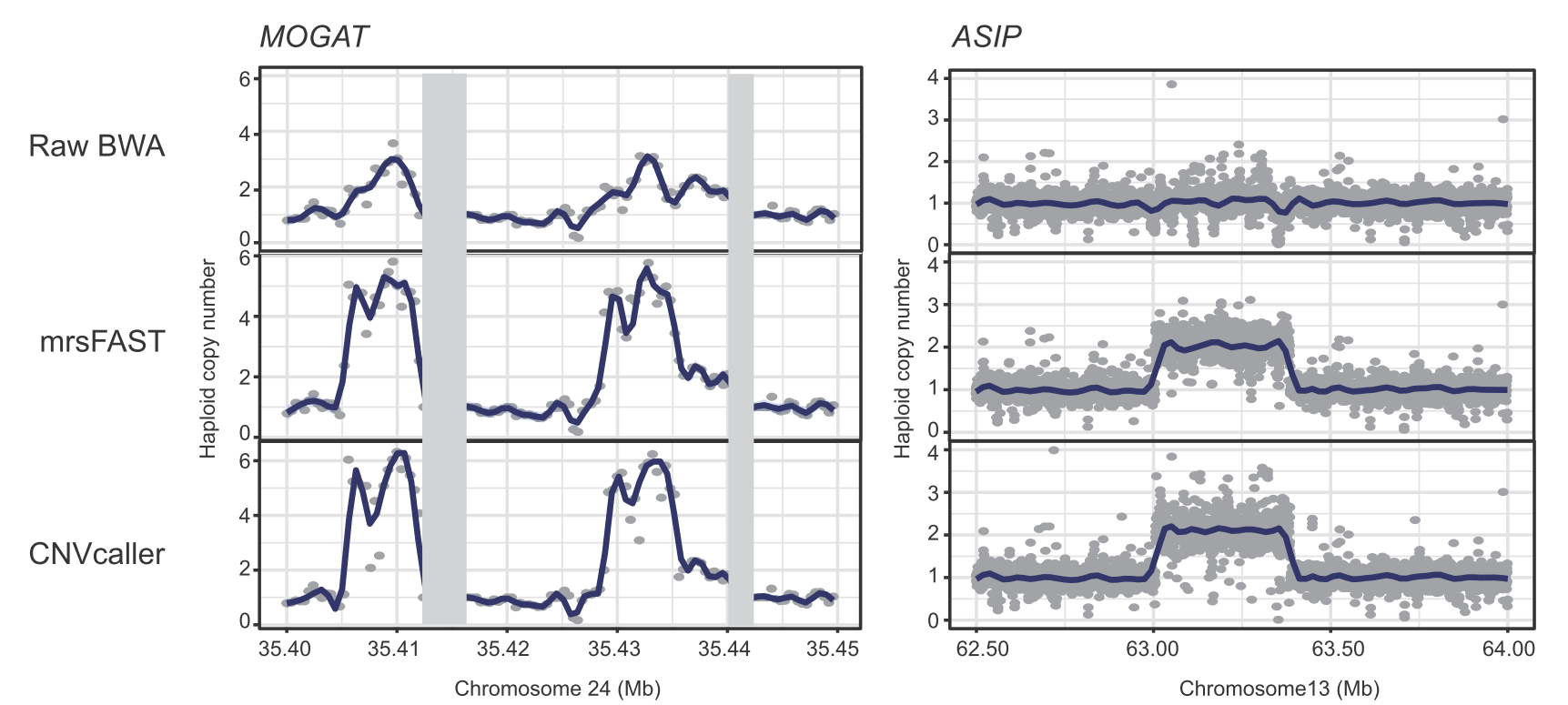


**Supplementary Figure 2** The haploid copy number distributions of a sheep sample at *ASIP* and *MOGAT* gene loci. The haploid copy number from top to bottom was counted from: raw BWA alignment; mrsFAST alignment; BWA alignment corrected by CNVcaller. The signals were normalized to one through divided by the global mean read depth of the sequencing data. The gray regions indicate the gaps in the reference genome.


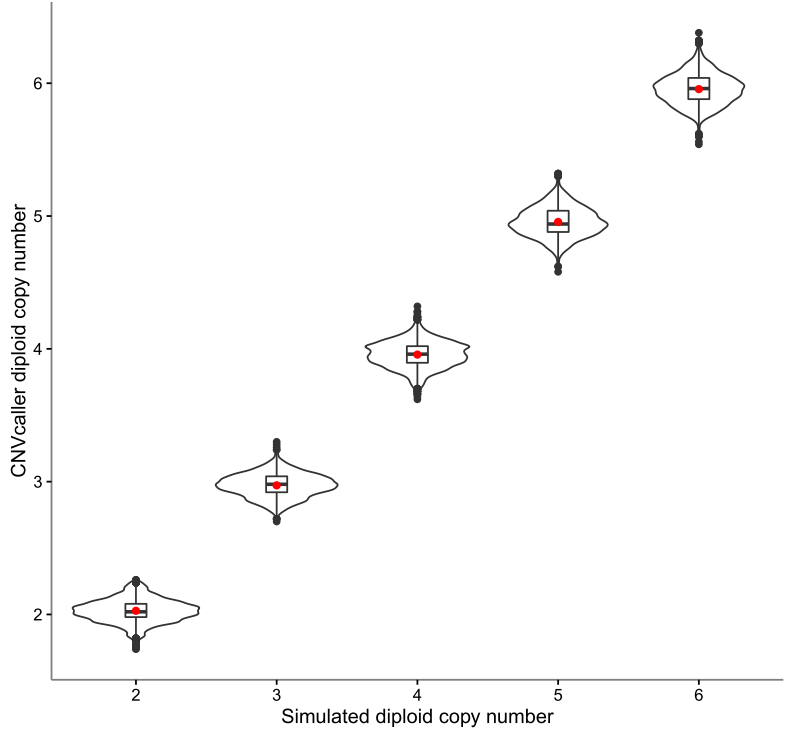


**Supplementary Figure 3** The absolute copy number of SD regions deduced by CNVcaller against the simulated copy number: 2, 3, 4, 5, 6. The red points indicate the mean values.


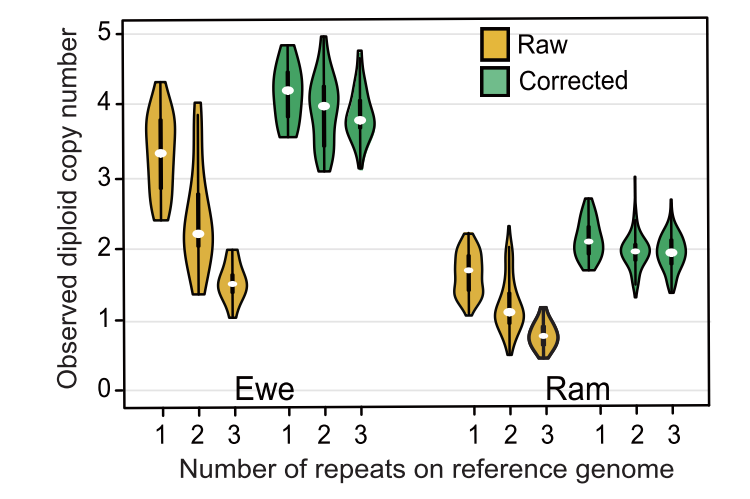


**Supplementary Figure 4** The raw and corrected copy number of 36 duplicated X-origin scaffolds grouped by the number of repeats on reference genome. Noteworthy, the raw copy numbers were split among the putative SDs.

**Supplementary Figure 5** FDR via the rate of Mendelian inconsistency and the number of detected CNVRs in the 10 Dutch families using CNVcaller, CNVnator and Genome STRiP.

**Supplementary Table 1** Validation sequencing data information

| Species (Data source) | Sample name |
| --- | --- |
| Human (1000 Genome Project, BAM files) | HG00133, HG00258, HG01886, HG02024, HG02025, HG02026, HG02922, HG02943, HG02974, HG03007, HG03099, HG03114, HG03121, HG03123, HG03132, HG03135, HG03616, HG03796, HG03802, NA11918, NA11920, NA12006, NA12044, NA12342, NA12760, NA12872, NA12878*, NA12891*, NA12982* |
| Human family (Genomes of Netherlands, GoNL project, BAM files) | gonl11, gonl118, gonl119, gonl120, gonl121, gonl122, gonl123, gonl124, gonl125, gonl126 |
| Goat (NCBI, FASTQ files) | ERR340328, ERR340340, ERR340341, ERR340342, ERR340343, ERR340344, ERR340345, ERR340347, ERR340348, ERR340426, ERR470100, ERR340329, ERR470104, ERR470106, ERP001584, SRP047212, ERR340330, ERR340331, ERR340333, ERR340334, ERR340335, ERR340336, ERR340338, SRP069238, ERR219543, ERR229485, ERR229487, ERR232492, ERR234304, ERR234305, ERR234315, ERR246143, ERR246152, ERR246153, ERR248926, ERR219546, ERR248928, ERR248929, ERR248933, ERR299283, ERR313257, ERR313258, ERR313259, ERR313264, ERR313266, ERR313272, ERR229471, ERR315498, ERR315500, ERR315503, ERR315505, ERR315508, ERR315510, ERR315512, ERR315515, ERR315516, ERR315795, ERR229474, ERR318768, ERR332581, ERR332592, ERR340428, ERR345973, ERR229476, ERR229478, ERR229479, ERR229481, ERR229484, ERR470101, ERR470102, ERR470103, ERR470105, ERR405774, ERR405775, ERR405776, ERR405777, ERR405778, ERR318225, ERR318226, ERR318227, ERR318228, ERR318229, ERR297229, ERR313206, ERR313207, ERR313209, ERR313210, ERR313211, ERR313212, ERR313213, ERR313215, ERR340332, ERR340337, ERR299449, ERR340339, ERR299456, ERR313197, ERR313198, ERR313199, ERR313200, ERR313202, ERR313204, SRP012150 |
| Sheep (International Sheep Genomics Consortium, FASTQ files) | OJA5, PD454, CHU2, SWAN3, NQA11, CHVC1, GCN5, CAS1, GAR4, GAR14, TEX454, OJA4, GCN4, CHVA1, CHA05, AWT1, AWD1, AWD3, AW454, RDA4, NDZ1, MERA1, KRS3, GUR5, SWAA27, CAS3, TWM1, WHSF1, SUM2, FIN4, SKZ4, SALA1, DWM1, CC50, SWAA29, BGE4, AWT2, AFS33, RDA2, NDZ4, MERC1, KR4, GUR4, EMZ1, SMS2, ZB08, SWAN4, SUM7, VBS2, ROM454, CHA02, FIN1, BCS3, BGE2, AFS32, MER454, LAC84, LAC1, KRS5, CHU1, BCS1, ZD11, SBF454, SKZ1, SALA2, SALC1, BSI3, BMN4, BSI4, BMN3 |
| Maize (FASTQ files) | F7, HP301 |
| Soybean (FASTQ files) | IGDB-TZX-270, IGDB-TZX-611 |

*Deep sequencing coverage (~50X) BAM files of NA12878, NA12891 and NA12892 were used, the normal (~5.3X) coverage BAM file of NA12878 were also used in the test.

**Supplementary Table 2** PCR fragments sizes and target loci sequences in CNVplex

| Chr | Start | End | Product Size (bp) | Target Genomic Sequences |
| --- | --- | --- | --- | --- |
| 3 | 204,143,305 | 204,143,363 | 59 | cagaatacgttccaggaagttaatgattggcagaagagaatcaaggttgataagaaaga |
| 4 | 105,951,521 | 105,951,567 | 47 | gagaaccctcagctcttccctggttctcccatttgcatggtagatgc |
| 5 | 11779195 | 11,779,235 | 41 | atggtgaatgcagggtggaggggctgcaacagttgaggggc |
| 23 | 62,103,998 | 62,104,043 | 46 | gctgtctgcaatggtcagggtcaacgtctcagctgagcttaggctt |

**Supplementary Table 3** Individual processing time and memory footprint of genomes with different genome size and unplaced scaffold number

| Species | Reference genome information | | Sequencing coverage (X) | Time (minutes) | | Memory (MB) | |
| --- | --- | --- | --- | --- | --- | --- | --- |
|  | Length (Mb) | Scaffold number |  | CNVcaller | CNVnator | CNVcaller | CNVnator |
| human | 3,137 | 86 | 5.3 | 21 | 9 | 556 | 12,815 |
|  |  |  | 10.2 | 40 | 38 | 585 | 12,817 |
| maize | 2,068 | 523 | 5.9 | 15 | 10 | 549 | 8,735 |
|  |  |  | 8.9 | 22 | 18 | 581 | 8,735 |
| soybean | 979 | 1,191 | 4.7 | 5 | 23 | 222 | 4,579 |
|  |  |  | 9.8 | 11 | 22 | 248 | 4,580 |
| sheep | 2,619 | 5,698 | 5.1 | 12 | 186 | 524 | 11,650 |
|  |  |  | 10.1 | 23 | 162 | 541 | 10,829 |
| goat | 2,923 | 29,907 | 6.5 | 18 | 3796 | 465 | 12,017 |
|  |  |  | 10.7 | 26 | 3765 | 477 | 12,016 |

**Supplementary Table 4** RD and STDEV of the simulate data before and after absolute copy number correction

| Simulated copy number | Total window number | Raw RD | Raw STDEV | corrected RD | corrected STDEV |
| --- | --- | --- | --- | --- | --- |
| 2 | 46,571 | 88.41 | 19.33 | 176.05 | 15.83 |
| 3 | 52,746 | 131.04 | 24.4 | 260.51 | 19.7 |
| 4 | 54,414 | 174.12 | 27.09 | 347.53 | 20.88 |
| 5 | 43,021 | 217.28 | 24.58 | 434.4 | 21.69 |
| 6 | 36,594 | 260.63 | 20.46 | 521.36 | 21.56 |

**Supplementary Table 5** Detailed information of the CNVRs detected from 1000GP Phase3 data

| CNVR size  (kb) | Deletion | | | |  | Duplication | | | |
| --- | --- | --- | --- | --- | --- | --- | --- | --- | --- |
|  | Number of CNVRs | IRS verifiable^1^ | FDR | Overlap with SDs^2^ |  | Number of CNVRs | IRS verifiable | FDR | Overlap with SDs |
| **CNVcaller** |  |  |  |  |  |  |  |  |  |
| < 2.5 | 308 | 66 | 0.27 | 67 |  | 191 | 34 | 0.24 | 64 |
| 2.5-5 | 687 | 204 | 0.07 | 161 |  | 388 | 85 | 0.19 | 163 |
| 5-10 | 322 | 88 | 0.09 | 92 |  | 155 | 51 | 0.12 | 90 |
| 10-20 | 95 | 34 | 0.06 | 39 |  | 82 | 33 | 0.06 | 52 |
| >= 20 | 75 | 35 | 0.00 | 27 |  | 86 | 43 | 0.09 | 61 |
| **CNVnator** |  |  |  |  |  |  |  |  |  |
| < 2.5 | 94 | 21 | 0.00 | 1 |  | 2 | 0 | NA | 0 |
| 2.5-5 | 314 | 88 | 0.02 | 14 |  | 52 | 7 | 0.29 | 11 |
| 5-10 | 667 | 232 | 0.09 | 53 |  | 263 | 51 | 0.12 | 95 |
| 10-20 | 383 | 148 | 0.08 | 59 |  | 282 | 75 | 0.27 | 151 |
| >= 20 | 302 | 90 | 0.00 | 84 |  | 452 | 136 | 0.19 | 307 |
| **Genome STRiP** |  |  |  |  |  |  |  |  |  |
| < 2.5 | 2,212 | 571 | 0.01 | 25 |  | 0 | 0 | NA | 0 |
| 2.5-5 | 1,412 | 996 | 0.05 | 28 |  | 634 | 346 | 0.88 | 41 |
| 5-10 | 1,108 | 913 | 0.04 | 40 |  | 355 | 203 | 0.47 | 60 |
| 10-20 | 264 | 255 | 0.00 | 18 |  | 136 | 94 | 0.17 | 31 |
| >= 20 | 185 | 178 | 0.00 | 9 |  | 125 | 108 | 0.02 | 34 |

^1^IRS verifiable means the number of CNVRs covering at least one probe of Affymetrix SNP 6.0 array, therefore can be verified by IRS rest.

^2^Overlap with SDs means the number of CNVRs have >50% intersection with the human SD database (similarity >97%).
