# Supplementary material for: CNVcaller: highly efficient and widely applicable software for detecting copy number variations in large populations
Source: Gigascience. 2017 Dec 4;6(12):1–12. doi: 10.1093/gigascience/gix115 (PMC5751039; doi:10.1093/gigascience/gix115)
Supplement: Response_to_Reviewer_Comments_Original_Submission.pdf [file gix115_response_to_reviewer_comments_original_submission.pdf]

Author's Response To Reviewer Comments

GIGA-D-17-00119
CNVcaller: Highly Efficient and Widely Applicable Software for Detecting Copy Number Variations in Large Populations
Xihong Wang; Zhuqing Zheng; Yudong Cai; Ting Chen; Chao Li; Weiwei Fu; Yu Jiang
GigaScience

Dear Dr. Edmunds

Thank you very much for handling our manuscript "CNVcaller: Highly Efficient and Widely Applicable Software for Detecting Copy Number Variations in Large Populations " (GIGA-D-17-00119). We appreciate all the comments from the reviewers, which helped us improve our manuscript. We have now revised the manuscript according to the reviewers' comments and your instructions.

We addressed the comments and questions of the reviewers as explained below, the reviewers' text has been included and our responses are in colored italics. Revised text is indicated by quotation marks. Because several new figures have been added, we attach a list of the current figures and tables corresponding to those from last version so that the changes can easily be tracked.

Upon the suggestions of the reviewers, we modified the manuscript as follows:

1. The newly released version of CNVcaller updated the genotyping method. The python package, scikit-learn v0.19.0, was used to decompose the reported copy numbers into several Gaussian distributions. Therefore, the accuracy of the CNVcaller in the new version was increased.

2. Since the reviewers required to evaluate the effects of the length and allele frequency of the discovered CNVRs. Two result sections have been added to analyze the number and FDR of the CNVRs detected by the three methods against the length and allele frequency. One section including Figure 4 was based on the sheep data, the other section including Figure 6 was based on the human data.

3. To answer the reviewer's question about the difference of the FDR in deletions and duplications, their FDR was evaluated respectively in the result sections.

4. The high-proportion mis-assembled segmental duplications in non-human assemblies caused misunderstanding of the reviewer. The section has been extensively redrafted, as analyzing both real and simulated data.

5. The previous discussion sections has been merged to the result sections to reduce the length of the manuscript. The first part of the previous results has been moved to the method sections as suggested by the reviewer.

6. The language has been professionally edited by an English editing service agency, American Journal Experts (AJE). (Because the first version is inadequate, we are waiting for the second version.)

Thank you again for all of your assistance.
Sincerely yours,
Yu Jiang, and other coauthors

****
REVIEWS
****
Reviewer #1:

The authors developed a new CNV caller pipeline which they called CNVcaller geared towards improved speed compared to existing CNV callers and improved accuracy for high complexity genomes. I commend the authors on their efforts to introduce improved algorithms and pipelines for an inherently difficult procedure, namely CNV calling. My comments are mostly suggestions for improvement as followss. Note, comments of the form (4:5 for example represent page 4, line 5).
Thanks for your positive comments and encouragement. We have substantially revised the manuscript upon your suggestions.

There are several grammatical errors which make the paper somewhat confusing. I would strongly recommend further extensive English editing.
We apologize for these mistakes. The manuscript has been professionally edited by an English editing service agency, American Journal Experts (AJE) .

My main criticism of the analysis is one that I have seen repeatedly of most other CNV calling publications, and that is there is no sensitivity analysis.
We are sorry for the ambiguous of the sensitivity tests, which were stuffed in previous Table1. In the revised manuscript, new figures and tests have been added. On general, CNVcaller demonstrated 57%-67% sensitivity for duplications, and 66%-68% for deletions in human data. The sensitivity in sheep data was ~73%.
The detailed descriptions were as follows:
"The sensitivity of human data was estimated as the proportion of the high-confident CNVR database that overlapped by the predicted CNVRs. Two previously published high-confident databases, including the particular samples, were the aCGH-based CNVR database and the 1000GP CNVR map. For the highly variable FDR, the sensitivity estimation removed the calls that were <=2,500 bp and had an alternative allele frequency <5%. For the aCGH database, CNVcaller demonstrated the highest sensitivity (57%) for duplication, 14% and 26% higher than Genome STRiP and CNVnator. Whereas Genome STRiP achieved the highest sensitivity (74%) in deletions, 8% and 2% higher than CNVcaller and CNVnator (Figure 6C). For the 1000 GP CNV maps, even though both Genome STRiP and CNVnator were the core methods of creating the library, the sensitivity of CNVcaller were 68% and 67% for deletions and duplications, only 4%-10% lower than Genome STRiP and CNVnator."

“Because the lack of validated sheep CNVR database, the sensitivity was validated indirectly. Based on our integrated analysis (see method), there are 138 sheep X chromosome origin scaffolds, which were not anchored onto chromosomes of OAR v3.1. Therefore, all of these scaffolds should be detected as CNV because the rams had half copy numbers of ewes. As a result, CNVcaller detected 101 out of these 138 X-origin scaffolds, with a sensitivity of 73%. Furthermore, the corrected copy numbers of these scaffolds were centralized at integer (Figure 3D), whereas the peaks of the raw copy numbers were ambiguous because of splitting the raw RDs among the putative SDs (Supplementary Figure 4). In contrast, CNVnator and Genome STRiP could not report these unmapped CNVRs.”

The authors here also suggest various parameters throughout their paper for performing CNV calling, but there is no analysis of how the results change if these parameters are adjusted, i.e. no analysis of how robust your algorithm is to changes in the parameters.
Thank you for your suggestion. The FDR against the window size and allele frequency have been added in Supplementary Figure 1 and Figure 6B.
The following description was added in methods.
“The window size is an important parameter for the RD methods. CNVcaller uses half of the window size as step size. The optimal window size is 800 bp for 5-10X coverage human and livestock sequencing data (Supplementary Figure 1). The recommended scales roughly inversely coverage, resulting in 400 bp windows for 20X coverage and 200 bp windows for 50X coverage.”

As another example, Hong et al 27503473 has demonstrated that the biggest variability in calling CNVs is in terms of the CNV size. I suspect that the same can be said of CNVcaller. Please comment on what sizes of CNVs does CNV caller do well or poorly on.
Figure 4 and Figure 6 have been added to evaluate the effect of the length and frequency in sheep and human data. On general, the performance of CNVcaller was good for deletions and duplications >2.5 Kb, however poorly on < 2.5 kb.
The detailed comparisons in the manuscript are as follows:
“The detected CNVRs of CNVcaller and Genome STRiP were further analyzed against the length and alternative allele frequency (Figure 4B). CNVcaller performed better in duplication detection, it can detect duplications <2.5 Kb, and the Mendelian inconsistence of longer calls were lower than Genome STRiP (3% versus 9% for 2.5Kb ~ 5Kb calls; 2% versus 5% for > 5Kb calls). On the other hand, Genome STRiP detected 1,958 more < 2.5Kb deletions than CNVcaller. One possible reason was Genome STRiP integrating RP methods which have higher capability in detecting shorter deletions. In terms of the frequency, because the detected samples were three trios, most CNVRs were medium frequency (6%-50%). The rare duplications tended to have a higher FDR than the median and high frequency calls (Figure 4C).”
“First, CNVcaller demonstrated the highest overall accuracy for detecting duplications, and the FDR of CNVcaller are relative consistent across duplication length and frequency categories. Whereas the short or singleton duplications of other two methods have high FDR. Second, 43% duplications detected by CNVnator were >20 kb. This was not due to the merged individual CNV to the CNVR, because the average size of individual calls was 3-4 times larger than the other methods. Third, Genome STRiP also showed the highest capability for detecting deletions, especially the short and rare ones, indicating the advantage of combining RD and RP methods in deletion.”

2:32 "the prevalent.." is a gross exaggeration. I think you mean "a prevalent".
Corrected as suggested.

2:35 I don't think you mean geometric. I did not comment on other grammatical/English errors as there were too many to list individually. I would highly recommend getting help with the English in this paper.
We apologize for these mistakes. The manuscript has been professionally edited by an English editing service agency, American Journal Experts (AJE).

3:53 "RD" is not defined.
We apologize for the missing. This description has been added to the introduction as follows. "Read-depth (RD) means the depth of the coverage or the genomic region that can be calculated by the number of reads aligned [16]."

6:120 Give a brief description of how CNVnator handles GC bias. Also why 40% for the GC bias? Shouldn't this parameter be dependent on the organism of interest?
We apologize for not clearly describing the procedure. In general, the mean RD of windows with 40% percent GC is only used as the temporary standard in the GC correction step. It will be lost in the following normalization step: the GC corrected RDs of each window are divided by the global median RDs. Because the denominator is calculated from the RDs already corrected by the 40% GC windows, this parameter will be lost and is not necessarily dependent on the organism of interest.
The CG correction of CNVnator was the combination of the correction and normalization steps of CNVcaller. The equation is as follow:

Where i is bin index, is raw RD signal for a bin, is corrected RD signal for the bin, is average RD signal over all bins, and is the average RD signal over all bins with the same GC content as in the bin.

The commentary on certain genomes not being as complete as others is important. I suspect though that if a large percentage of the samples show a CNV in a genome that is newer or not as complete, then this observation may be more likely indicative of a problem with the reference. Can you comment?
If the detected CNVR has variation in population, which means the read depths can be separated into two or more normal distributions, this call is probably true even with high frequency. On the contrary, if all of the individuals show the same abnormal read depth, it suggests the reference individual is indeed different from the sampling population or have assembly problems.

7:145 I am not convinced Pearson's correlation is appropriate. Your data is likely to have outliers and non-normal data. A non-parametric test of correlation like Spearman's correlation (Kendall-Tau is likely too computational intensive), or performing correlation after 5 or 10% trimming may be more appropriate.
We tried to replace the Pearson's correlation with Spearman's correlation in the 30 BAM files from 1000 Genome Project data. However, after replacement the FDR doubled while the length of each calls reduced to half. A possible reason was the Spearman's correlation was calculated by

sorting of the read depth. So, the diverged copy numbers of deletion or duplication individuals contribution no more than the subtle random mistakes of the normal individuals. In the low frequency CNVRs, the Spearman's correlation index was mainly contributed by the random mistakes of the normal copy individuals.
The trimming is also not recommended for similar reason. In the low frequency CNVRs, the individuals with abnormal copy number will be trimmed as outliers.

cn.MOPS (Klambauer et al, PMID: 22302147) uses a mixture of Poissons as opposed to Gaussian Mixture Models for CNV detection. I suspect the mixture of Poissions will be superior to Gaussian Mixture Models when the read depths are low, and Gausssian mixtures may be more appropriate when read depths are high. How difficult is it to replace the Gaussian mixtures with Poisson mixtures and compare the performance? I feel that this analysis would be informative and potentially improve your algorithm.
Thank you for your suggestion. However, it is not easy to replace the distribution because the RDs after GC correction and normalization are not integer so they can not be directly treated as Poisson distributions. Basically, CNV caller recommended a proper window size to make the standard variation less than 30% of the mean RD, which will not fit the Poisson distribution for RDs. Besides, we used the RDs of 232 goats with 10X coverage to test the fitness of Gaussian distribution using omnibus test (packages). As a result, 88% windows accepted the null hypothesis at P=0.01 level. So, we believe the Gaussian Mixture Models was acceptable for the 10 X data.

The term "CNVR" is critical for understanding the algorithm, and requires more explanation of the term.
We apologies for missing this important concept. The explanation has been added to the introduction as follows.
“To compare the copy number of a particular region across the samples, the shared CNVs among individuals are needed, so the unified CNV regions (CNVRs) were merged from the individual CNVs.”
It would be helpful to include some further discussion on where you see that CNVcaller works better or worse than existing CNV calling software.
Figure 2 showed the speed of CNVcaller was one to two orders of magnitudes higher than the other methods. Figure 4 and Figure 6 have been added to evaluate the effect of the length and frequency in sheep and human data. On general, the performance of CNVcaller was better for all sizes of duplications, however poorly on deletions < 2.5 kb.

9:180. The "arbitrary standards" require a citation.
Two citations were added.
1. Chain FJ, Feulner PG, Panchal M, Eizaguirre C, Samonte IE, Kalbe M, et al. Extensive copy-number variation of young genes across stickleback populations. PLoS genetics. 2014;10 12:e1004830.
2. Abyzov A, Urban AE, Snyder M and Gerstein M. CNVnator: an approach to discover, genotype, and characterize typical and atypical CNVs from family and population genome sequencing. Genome research. 2011;21 6:974-84.

Minor comment: Since speed seems to be a major selling point of the software, more details

about running the software on a compute cluster or running algorithms in parallel in the documentation would be helpful.
A new part of "Parallel submission of individual RD processing" has been added to the methods with the principle and command as follows.
"CNVcaller processes the BAM file of each individual separately in the first step, so parallel submissions can be used to save the total running time. All the BAM files should be equally distributed in to N groups, and each group contains M files. The max N = the available processing cores. M = the total number of BAM files/ N. For example, the 232 goat BAM files were processed on a node with 32 processing cores and 128 GB of RAM. We distributed the 232 files into 20 groups, and each group contained 12 BAM files. The shell command for one group likes following:

```
#!/bin/sh
for i in {1..M}
do bash Individual.Process.sh -b $i.bam -h $i -d dup -s sex_chromosome
done
```

After corrections and normalization, the comparable RDs of each sample are concentrated to an ~100 MB intermediate file and output. This design avoids the repeated calculation of the same individual in different populations."

****
Reviewer #2:

The proposed method "CNVcaller" enables the efficient discovery and genotyping of CNVs in large populations. One of the main benefits of the method is that it can handle draft genome assemblies with thousands of scaffolds. The computational benchmarks proof that the method is fast and memory efficient but the evaluation of the accuracy of the method is less convincing. Some details of the method remain vague and hinder an objective evaluation. Detailed comments of how to improve the manuscript are below:
Thank you for your affirmation. We are sorry for the ambiguous of the accuracy test, and have substantially revised the manuscript upon your suggestions. In the revised manuscript, the performance evaluation in previous Table 1 was described more detail and classify by the species in Figure 4 and Figure 6.

Comment 1 - The primary application of CNVcaller is the detection of CNVs in large populations. Population variant call sets are dominated by rare variants of rather small size. For instance, less than 20% of the 1000 Genomes structural variants have a population allele frequency >5% and almost 50% of the SVs are <2kbp in size despite the rather low coverage (~7x). CNVcaller is currently restricted to large CNVs (>2kbp) and common variants (>5% allele frequency), which is a major limitation for population genomic studies.
In the revised version, all detected calls were included in the IRS test. In fact, all three methods can report some short and rare CNVRs. However, the short and rare duplications made by Genome STRiP and CNVnator had extremely high FDR. So, we excluded these results from the previous version of manuscript as 1000 GP.
The newly added Figure 6 showed the shortest duplication reported by CNVnator and Genome STRiP was 2.8 kb and 2.5 kb, and the IRS FDR of 2.5-5kb calls are 29% and 88%, respectively. The FDR of >2.5kb singletons was 35% and 69% for CNVnator and Genome STRiP,

respectively. These uncertain calls were also removed by the phase 3 extended SV release of 1000GP. After extra quality controls, the number of duplications in the released database are only 1/7 of deletions, and the median size was 36 kb, 17 times longer than deletions. Therefore, improving the accuracy of duplications on this foundation is meaningful for enrich the CNV database. The main improvement of CNVcaller is the accuracy of duplications. The FDR of 2.5 kb – 5 kb was reduced to 19%, and the >2.5kb singletons was reduced to 9%. However, the FDR were still higher than the longer and higher frequency calls.

Besides, the current main usage of CNVcaller is to detect the CNVRs related to economy traits in livestock and crops. In these populations, the target CNVs usually have a medium or high frequency after long time artificial selection. We believe the high-confident medium to high frequency reported by CNVcaller can contribute to the functional and breeding study of non-human studies.

The sensitivity increase of CNVcaller for the subset of common and large CNVs seems to be driven by an increased number of detected CNVs in SD regions (Figure 5C). SNP arrays have a low SNP density in SD regions and in the present Manuscript array SNP probes in SD regions have been removed entirely. The reported IRS FDR is therefore heavily biased against CNVs in SD regions and it thus seems mandatory to me to proof that this sensitivity increase for SD-associated CNVs is not leading to an inflated FDR.

Thanks for your suggestions. Figure 5C (New Figure 3C) was updated to show both the number and the Mendelian inconsistence of the detected CNVs in SDs. The inconsistence rate of the calls in SD regions made by CNVcaller was about 3%. The copy numbers of unique and SDs were also indirectly validated by the X-origin scaffolds of a 133-sheep population. In both validation dataset, one main reason for acceptable FDR in SDs was most SDs in sheep reference genome assembly is actually mis-assembled unique region.

The detailed description are as follows:

“In the real dataset, CNVcaller detected more duplications in the SDs of the sheep genome with only 3% Mendelian inconsistence (Figure 3C, Supplementary Figure 3). Because the lack of validated sheep CNVR database, the sensitivity was validated indirectly. Based on our integrated analysis (see method), there are 138 sheep X chromosome origin scaffolds, which were not anchored onto chromosomes of OAR v3.1. Therefore, all of these scaffolds should be detected as CNV because the rams had half copy numbers of ewes. As a result, CNVcaller detected 101 out of these 138 X-origin scaffolds, with a sensitivity of 73%. Furthermore, the corrected copy numbers of these scaffolds were centralized at integer (Figure 3D), whereas the peaks of the raw copy numbers were ambiguous because of splitting the raw RDs among the putative SDs (Supplementary Figure 4). In contrast, CNVnator and Genome STRiP could not report these unmapped CNVRs.”

The Manuscript lacks a Figure that shows the size and allele frequency distribution of the discovered CNVs in comparison to Genome STRiP and CNVnator. An estimate of breakpoint accuracy of CNVcaller would also be valuable.

Thanks for your suggestion. Figure 4 and Figure 6 have been added to evaluate the effect of the length and frequency in sheep and human data. On general, the performance of CNVcaller was better for all sizes of duplications, however poorly on deletions < 2.5 kb.

The breakpoint accuracy is an innate disadvantage of RD methods. Because the detailed situation within a window is not calculated. And the window size cannot be too small for the medium or

low coverage sequencing data. We recommend the user to combine the read pair or split read methods to improve the breakpoint accuracy.
The detailed comparations in the manuscript are as follows:
“The detected CNVRs of CNVcaller and Genome STRiP were further analyzed against the length and alternative allele frequency (Figure 4B). CNVcaller performed better in duplication detection, it can detect duplications <2.5 Kb, and the Mendelian inconsistence of longer calls were lower than Genome STRiP (3% versus 9% for 2.5Kb ~ 5Kb calls; 2% versus 5% for > 5Kb calls). On the other hand, Genome STRiP detected 1958 more < 2.5Kb deletions than CNVnator. One possible reason was Genome STRiP integrating RP methods which have higher capability in detecting shorter deletions. In terms of the frequency, because the detected samples were three trios, most CNVRs were medium frequency (6%-50%). The rare duplications tended to have a higher FDR than the median and high frequency calls (Figure 4C).”
“First, CNVcaller demonstrated the highest overall accuracy for detecting duplications, and the FDR of CNVcaller are relative consistent across duplication length and frequency categories. Whereas the short or singleton duplications of other two methods have high FDR. Second, 43% duplications detected by CNVnator were >20 kb. This was not due to the merged individual CNV to the CNVR, because the average size of individual calls was 3-4 times larger than the other methods. Third, Genome STRiP also showed the highest capability for detecting deletions, especially the short and rare ones, indicating the advantage of combining RD and RP methods in deletion. Besides directly combination of the two methods into one piece of software, another option was using high-confidence RD results generated CNVcaller as the prior to improve the accuracy of the read pair/split read pipeline.”

The Manuscript mentions mrsFAST for absolute copy number validation. I could not find any formal comparison of predicted copy-number by mrsFAST and CNVcaller but maybe I missed this?
Supplementary Figure 2 (Previous Supplementary Figure 1) showed the copy number calculated from mrsFAST and CNVcaller was similar. However, mrsFAST needed to realign all the multi-hit reads in BWA alignments, leading to significantly increased computational time. For example, mrsFAST needed 10 hours for a 3G genome with 10X sequencing data, whereas, CNVcaller only needed 4 minutes.
- Please add to Table 1 the number of CNV sites that could be assessed by the IRS method and what proportion of each call set could be evaluated using IRS. I also believe the IRS method reports p-values separately for deletions, duplications and multi-allelic CNVs. Was there any difference among these for CNVcaller?
The detailed information of 1000GP calls including the required information has been added to Supplementary Table 5. Overall, 28%, 30% and 60% CNVRs of CNVcaller, CNVnator and Genome STRiP covered at least one probe of Affymetrix SNP 6.0 array, therefore can be assessed by IRS test. One main reason for the diverged testable proportion was only 4% of Genome STRiP calls were overlap with SDs which have seldom probes, whereas the 34% CNVcaller calls and 28%CNVnator calls were overlap with SDs.
Two extra genome-wide evaluations can provide supplemental proofs. The Mendelian inconsistence of 10 Dutch family was added in Supplementary Figure 5, which can test both unique and SD regions. The inconsistence rate of CNVcaller, CNVnator and Genome STRiP was 1.5%, 4.4%, and 0.4%. This accuracy ranking was consistent with the genotyping discordance compared with the aCGH database, which were 2.6%, 5.5% and 2.2% for CNVcaller, CNVnator

and Genome STRiP respectively.

- Some details of the method are vaguely specified and some Figures lack clarity and units.
Page 6, line 129: "... if the median RD of the homogametic sex chromosomes is about half of the median RD of autosome..."
Modified as follows:
“Most mammalian and avian genomes show XX/XY-type or ZZ/ZW-type sex-determining system. Their homogametic sex chromosomes (X or Z) constitute 5%-10% of the total genome, and show half RD of the autosomes in XY or ZW individuals. Therefore, insensitive corrections are needed. The name of the homogametic sex chromosome is required as a parameter. If the median RD of this chromosome is <0.6X of the median RD of the autosome, this individual is determined as the XY or ZW type, and the RDs of this chromosome are doubled before normalization. Otherwise, this individual is determined as XX or ZZ type, and no sex correction will be done.”

Page 8, line 154: "... and the distance between them is less than a certain percent of their own length."
Modified as follows: “The distance between the two initial calls is less than 20% of their combined length.”

Page 5, line 91: "The reference genome is segmented into overlapping sliding windows." What window size and overlap was used for high-coverage genomes?
The following description was added in methods.
“The window size is an important parameter for the RD methods. CNVcaller uses half of the window size as step size. The optimal window size is 800 bp (with a 400 bp overlap) for 5-10X coverage human and livestock sequencing data (Supplementary Figure 1). The recommended scales roughly inversely coverage, resulting in 400 bp windows for 20X coverage and 200 bp windows for 50X coverage.”

Page 5, line 95: "The raw RD signal is calculated for each window as the number of placed reads with centers within window boundaries." Does this imply that for paired-end data both reads are counted?
Yes, considering the uncontrollable effect of gap ratios from different genome assemblies, all of the end reads located in the window are independently added to the RD of this window, regardless of the read is from single end mapping or paired mapping.

Page 8, line 154: "Then the two adjacent initial calls are further merged if their copy numbers are highly correlated". What threshold was used?
Modified as follows: “CNVRs can be separated by gaps or poorly assembled regions, therefore, the adjacent initial calls are merged if their RDs are highly correlated. The default parameters are: the distance between the two initial calls is less than 20% of their combined length, and the Person’s correlation index of the two CNVRs is significant at P = 0.01 level.”

Figure 3A: CNVcaller 13.7. What is the unit? Are these 13,700 CNVs?
The unit of this figure was Mbp, because the intersection of the three methods was hard to define in number with different boundaries, so they are evaluated in length. CNVcaller covered 40% of

the CNVRs detected by CNVnator, 45% of Genome STRiP and 65% of their intersection, in length.

Minor:
- I could not find a reference to the 232 goat sequencing data? Is this data publicly available?
Among the 232 goat whole genome sequencing data, 103 were acquired from NCBI (reference paper see below), and the accession numbers are provided in Supplementary Table 1. The remaining 129 samples without accession number were generated by ourselves.
Badr Benjelloun FJA, Streeter I, Boyer F, Coissac E, Stucki S, et al. (2015) Characterizing neutral genomic diversity and selection signatures in indigenous populations of Moroccan goats (Capra hircus) using WGS data. Frontiers in genetics 6.
Dong Y, Zhang X, Xie M, Arefnezhad B, Wang Z, et al. (2015) Reference genome of wild goat (capra aegagrus) and sequencing of goat breeds provide insight into genic basis of goat domestication. BMC genomics 16: 431.
Dong Y, Xie M, Jiang Y, Xiao N, Du X, et al. (2013) Sequencing and automated whole-genome optical mapping of the genome of a domestic goat (Capra hircus). Nature biotechnology 31: 135-141.
Bickhart DM, Rosen BD, Koren S, Sayre BL, Hastie AR, et al. (2017) Single-molecule sequencing and chromatin conformation capture enable de novo reference assembly of the domestic goat genome. Nature Genetics 49: 643-650.
Wang XL, Liu J, Niu YY, Li Y, Zhou SW, et al. Low incidence of SNVs and indels in trio genomes of Cas9-mediated multiplex edited sheep. BMC Genomics. Under review.

- The first Results section "Overview of CNVcaller algorithm" seems better suited for the Methods part.
Modified as suggested.

- Is the Mendelian consistency higher for the high-coverage trio: NA12878, her father (NA12891) and her mother (NA12892)?
Yes. Upon the high coverage data of all three members of the trio (NA12891, NA12892 and NA12878 are all 50 X), the inconsistent rate was 2.4%. Upon the high coverage parents (50 X NA12891 and NA12892) and low coverage child (5.3 X NA12878), the inconsistent rate was 6.1%. So, the increased sequencing depth can help to reduce the number of false positives.

- I believe the claim that read-pair/split-read algorithms are less powerful on draft assemblies of non-model organisms compared to read-depth methods is potentially true but the Manuscript lacks a proof for this or a citation that supports this claim.
Thank you for your agreement. This problem was found in our previous reference genome assembly projects for both sheep and goats. However, we did not report this result in the section of CNV/SD detection. So, we removed this comment from this manuscript. However, we found the following citations may help to support this claim:
All of these algorithms including read-pair/split-read (RP/SR) and read-depth rely on mapping sequencing reads back to reference genome. However, for many non-model organisms, the reference genome likely contains many errors, which mainly arose from repeat collapse and expansion; and rearrangement and inversion [1] . These mis-assembly sequences and the repetitive regions of the genome can result in many pair-end reads have multiple good mappings,

thus it is difficult for RP/SR to uniquely identify the true CNVs boundaries[2]. However, based on read depth by considering all possible map locations for a read can address this problem[3].

1. Phillippy AM, Schatz MC, Pop M (2008) Genome assembly forensics: finding the elusive mis-assembly. Genome biology 9: R55.
2. He D, Hormozdiari F, Furlotte N, Eskin E (2011) Efficient algorithms for tandem copy number variation reconstruction in repeat-rich regions. Bioinformatics 27: 1513-1520.
3. Alkan C, Kidd JM, Marques-Bonet T, Aksay G, Antonacci F, et al. (2009) Personalized copy number and segmental duplication maps using next-generation sequencing. Nature genetics 41: 1061-1067.

- It is not clear from the Manuscript if CNVcaller reports copy-number likelihoods based on the Gaussian mixture model. Please clarify.

Thank you for you suggest. CNVcaller reports the silhouette coefficients of the copy numbers instead of the Gaussian mixture model likelihoods as quality control. Because we found silhouette coefficients has greater correlation with IRS test result than likelihoods.

- Figure 5A: Why is the absolute copy-number correction different for Human and Sheep?

We are sorry for not clearly interpreting the high-proportioned mis-assembled segmental duplications in non-human assemblies. This part was modified as follows:

“Previous studies showed high proportion of SDs in animal genomes are mis-assembled single copy regions. So, we validated the copy numbers on human (hg19) and sheep (OAR v3.1) reference genome assembly by the sequencing copy number of a human (NA12878) and a Tan sheep sample (Figure 6A). If the SDs were correctly assembled, the sequencing diploid copy number should be two times of the copy number of SDs. For example, the average sequencing copy number of the two-copy SDs was four in NA12878. However, the corresponding sequencing copy number of sheep was only 2.4. These results indicated most two-copy SDs of hg19 were truly duplicated in NA12878 while approximately 80% of the two-copy SDs in OAR v3.1 were unique regions in the Tan sheep sample. So, the SDs in sheep genome were called “putative SDs” before validation.”

- There is quite a few typing and grammatical errors. For instance:

*Figure 2B: Max mamory
*Supplementary Table 3: Memery
*Page 3, line 53: ...the number of reads aligned to of a particular region.
*Page 8, line 160: This model presets the average copy number of homozygous deletion, heterozygous deletion, normal, heterozygous deletion (duplication!), homozygous deletion (duplication!) at zero to four respectively.

We are sorry for these mistakes. We have proofread the revised manuscript and used professional English language editing to minimize the grammatical errors.

****

Checklist of the updated tables and figures

Current version Last version
Fig. 3 Fig. 5
Fig. 4A-C Table 1 and newly added
Fig. 4D Fig. 3B
Fig. 5 Fig. 4
Fig. 6A-C Table 1 and newly added
Fig. 6D Fig. 3A
Supplementary Fig. 1 Newly added
Supplementary Fig. 2 Supplementary Fig. 1
Supplementary Fig. 3 Newly added
Supplementary Fig. 4 Supplementary Fig. 2
Supplementary Fig. 5 Table 1 and newly added
Supplementary Table 4 Newly added
Supplementary Table 5 Newly added
