# Supplementary material for: CNVcaller: highly efficient and widely applicable software for detecting copy number variations in large populations
Source: Gigascience. 2017 Dec 4;6(12):1–12. doi: 10.1093/gigascience/gix115 (PMC5751039; doi:10.1093/gigascience/gix115)
Supplement: Response_to_Reviewer_Comments_Revision_1.pdf [file gix115_response_to_reviewer_comments_revision_1.pdf]

Author's Response To Reviewer Comments

Dear Dr. Edmunds,

Thank you very much for handling our manuscript "CNVcaller: Highly Efficient and Widely Applicable Software for Detecting Copy Number Variations in Large Populations " (GIGA-D-17-00119). We appreciate all the comments from the reviewers, which helped us improve our manuscript. We have now revised the manuscript according to the reviewers' comments and your instructions.
We addressed the comments and questions of the reviewers as explained below; the reviewers' text has been included, and our responses are in coloured italics. The revised text is indicated by quotation marks. Because several new figures have been added, we have attached a list of the current figures and tables corresponding to those in the last version so that the changes can be easily tracked.

According to the suggestions of the reviewers, we have modified the manuscript as follows:

1. The newly released version of CNVcaller updated the genotyping method. A python package, scikit-learn v0.19.0, was used to decompose the reported copy numbers into several Gaussian distributions. Therefore, the accuracy of CNVcaller in the new version was increased.

2. The reviewers requested that we evaluate the effects of the length and allele frequency of the discovered CNVRs. Therefore, two sections have been added to the results analysing the number and FDR of the CNVRs detected by the three methods against the length and allele frequency. One section (including Figure 4) was based on the sheep data, the other section (including Figure 6) was based on the human data.

3. To answer the reviewer's question about the difference between the FDRs in deletions and duplications, their FDRs were evaluated separately in the results section.

4. The high proportion of misassembled segmental duplications in non-human assemblies may have led to misunderstanding on the reviewer's part. This section has been extensively redrafted with analyses of both real and simulated data.

5. The previous discussion section has been merged with the results section to reduce the length of the manuscript. The first part of the previous results section has been moved to the methods section, as suggested by the reviewer.

6. The language has been professionally edited by an English-language editing service, American Journal Experts (AJE).

7. We have registered the software in the SciCrunch.org database. The RRID SRC_015752 was added to the 'Availability and requirements' sections.

Thank you again for all of your assistance.

Sincerely yours,
Yu Jiang and co-authors

****
REVIEWS
****
Reviewer #1:

- The authors developed a new CNV caller pipeline which they called CNVcaller geared towards improved speed compared to existing CNV callers and improved accuracy for high complexity genomes. I commend the authors on their efforts to introduce improved algorithms and pipelines for an inherently difficult procedure, namely CNV calling. My comments are mostly suggestions for improvement as follows. Note, comments of the form (4:5 for example represent page 4, line 5).

Thank you for your positive comments and encouragement. We have substantially revised the manuscript upon your suggestions.

- There are several grammatical errors which make the paper somewhat confusing. I would strongly recommend further extensive English editing.

We apologize for these mistakes. The manuscript has been professionally edited by an English-language editing service, American Journal Experts (AJE) .

- My main criticism of the analysis is one that I have seen repeatedly of most other CNV calling publications, and that is there is no sensitivity analysis.

We are sorry for the ambiguity of the sensitivity tests, which were included in the previous Table 1. In the revised manuscript, Figure 6C has been added to describe the sensitivity. In general, CNVcaller demonstrated 57%-67% sensitivity for duplications and 66%-68% for deletions in human data. The sensitivity in sheep was ~73% by indirectly evaluation. The detailed descriptions are as follows:

Human: “The sensitivity was estimated as the proportion of the high-confidence CNVR database that overlapped with the predicted CNVRs. Two previously published high-confidence databases that include our test samples are the aCGH-based CNVR database [1] and the 1000GP CNVR map [2]. For the aCGH database, CNVcaller demonstrated the highest sensitivity (57%) in duplications, whereas Genome STRiP achieved the highest sensitivity (74%) in deletions (Figure 6C). Both Genome STRiP and CNVnator were the core contributors to the 1000GP CNV maps; However, the sensitivity of CNVcaller was 68% and 67% for deletions and duplications according to this database, only 4%-10% lower than Genome STRiP and CNVnator.”

Sheep: “The sensitivity of sheep CNVRs was estimated indirectly due to the lack of a validated

database. Based on our integrated analysis (see methods), there were 138 sheep X chromosome-origin scaffolds, which were not anchored onto chromosomes of OAR v3.1. Therefore, all of these scaffolds should be detected as CNVs because the rams had half the copy numbers of the ewes. As a result, CNVcaller detected 101 of these 138 X-origin scaffolds, with a sensitivity of 73%. In contrast, CNVnator and Genome STRiP did not report these unmapped CNVRs."

- The authors here also suggest various parameters throughout their paper for performing CNV calling, but there is no analysis of how the results change if these parameters are adjusted, i.e. no analysis of how robust your algorithm is to changes in the parameters.

Thank you for your suggestion. Two important parameters of CNVcaller were window size and minimum report allele frequency. The FDRs against the window size and alternative allele frequency have been added to Supplementary Figure 1 and Figure 6B. In general, with the increasing of window size and allele frequency, the accuracy raised while the sensitivity decreased.

- As another example, Hong et al 27503473 has demonstrated that the biggest variability in calling CNVs is in terms of the CNV size. I suspect that the same can be said of CNVcaller. Please comment on what sizes of CNVs does CNV caller do well or poorly on.

Thank you for your suggestion. Figure 4 and Figure 6 have been added, which evaluate the effects of length and frequency in sheep and human data. The detailed comparisons in the manuscript are as follows:

Sheep: "The accuracy was evaluated by the Mendelian inconsistency of all the CNVRs on autosomes against the length and alternative allele frequency (Figure 4). CNVcaller achieved higher accuracy than Genome STRiP in both deletion (1% vs 2%) and duplication (4% vs 7%) (Figure 4A). Whereas Genome STRiP had greater capability to detected short (<2.5 kb) deletions (Figure 4B), indicating the RP methods integrated in Genome STRiP performed well on small deletions. Concerning the alternative allele frequency, both methods showed an increased FDR in rare duplications (Figure 4C). However, CNVcaller is primarily used to detect CNVRs related to economic traits in livestock and crops. In these studies, the target CNVRs usually have a high frequency after long-duration breeding selection."

Human: "CNVcaller demonstrated the highest overall accuracy for detecting duplications and performed consistently across the length and frequency categories, whereas Genome STRiP and CNVnator had high FDRs on the short or singleton duplications (Figure 6A, B). Genome STRiP showed the greatest ability to detect deletions, indicating the advantage of combining RD and RP methods for deletion detection. The genotyping accuracy of the human dataset was further benchmarked against the high-confidence aCGH array-based database. The discordance rates of CNVcaller, CNVnator and Genome STRiP were 2.6%, 5.5% and 2.2%, respectively. This genotyping accuracy ranking was same with the Mendelian inconsistency of the 10 Dutch trios (Supplementary Figure 5)."

- 2:32 "the prevalent.." is a gross exaggeration. I think you mean "a prevalent".

This has been corrected as suggested.

- 2:35 I don't think you mean geometric. I did not comment on other grammatical/English errors as there were too many to list individually. I would highly recommend getting help with the English in this paper.

We apologize for these mistakes. The manuscript has been professionally edited by an English-language editing service, American Journal Experts (AJE).

- 3:53 "RD" is not defined.

We apologize for the missing definition. The following description has been added to the introduction:
“read depth (RD) refers to the depth of coverage in a genomic region that can be calculated from the number of aligned reads [14], a CNV region should have a higher or lower RD than expected [22-24].”

- 6:120 Give a brief description of how CNVnator handles GC bias. Also why 40% for the GC bias? Shouldn't this parameter be dependent on the organism of interest?

We apologize for not clearly describing the procedure. In general, the mean RD of windows with 40% percent GC is used as only a temporary standard in the GC correction step. It will be lost in the following normalization step, in which the GC-corrected RDs of each window are divided by the global median RDs. Because the denominator is calculated from the RDs already corrected by the 40% GC windows, this parameter will be lost and is not necessarily dependent on the organism of interest.

The GC corrected RD for a window is calculated by CNVnator as follows: the raw RD times the global average RD and divided by the average RD with the same GC content as in this window. Because the global average RD is calculated before the GC correction, no temporary parameter is used. The equation is showed in Personal Cover.

- The commentary on certain genomes not being as complete as others is important. I suspect though that if a large percentage of the samples show a CNV in a genome that is newer or not as complete, then this observation may be more likely indicative of a problem with the reference. Can you comment?

If the detected CNVR has variation in a population, which means the read depths can be clustered into two or more normal distributions, this CNVR is probably true even with high frequency. In contrast, if all of the individuals show the same abnormal read depth, this suggests that the reference individual is different from the sample population or some assembly problems exist.

- 7:145 I am not convinced Pearson's correlation is appropriate. Your data is likely to have outliers and non-normal data. A non-parametric test of correlation like Spearman's correlation

(Kendall-Tau is likely too computational intensive), or performing correlation after 5 or 10% trimming may be more appropriate.

We tried replacing Pearson's correlation with Spearman's correlation in the 30 BAM files from the 1000 Genome Projects data. However, the FDR doubled after the replacement, while the length of each call was reduced by half. A possible reason is that Spearman's correlation is calculated by ranking instead of the numerical value of copy numbers across samples. Therefore, the divergent copy numbers of individuals with deletions or duplications contributed no more than the subtle random mistakes of normal copy individuals, especially in the low-frequency CNVRs.

Trimming is also not recommended for a similar reason. In the low-frequency CNVRs, individuals with an abnormal copy number will be trimmed as outliers.

- cn.MOPS (Klambauer et al, PMID: 22302147) uses a mixture of Poissons as opposed to Gaussian Mixture Models for CNV detection. I suspect the mixture of Poissions will be superior to Gaussian Mixture Models when the read depths are low, and Gausssian mixtures may be more appropriate when read depths are high. How difficult is it to replace the Gaussian mixtures with Poisson mixtures and compare the performance? I feel that this analysis would be informative and potentially improve your algorithm.

Thank you for your suggestion. However, it is not easy to replace the distribution because the RDs after GC correction and normalization are not integers; thus, they cannot be directly treated as Poisson distributions. Additionally, we totally agree with your comment about the Poisson distribution will be superior for low read depths with high STDEV. However, the currently common sequencing depth are about 5-10X. Under this sequencing depth and a proper window size, the STDEV/mean RD was only 0.2-0.3, which essentially not fit the Poisson distribution. In addition, we used the RDs of 232 goats with ~10X coverage to test the fitness of Gaussian distribution using the omnibus test (scipy 0.19.0). As a result, 88% of windows accepted the null hypothesis at the P = 0.01 level. Therefore, we believe the Gaussian Mixture Model is acceptable for the current data.

- The term "CNVR" is critical for understanding the algorithm, and requires more explanation of the term.

We apologize for missing this important concept. The following explanation has been added to the introduction:
“To study the polymorphism among individuals, the overlapping CNVs need to be merged into unified regions, namely CNV regions (CNVRs)”

- It would be helpful to include some further discussion on where you see that CNVcaller works better or worse than existing CNV calling software.

Thank you for your suggestion. Figure 2 shows that the speed of CNVcaller was one to two orders of magnitude higher than the other methods. Newly added Figure 4 and Figure 6 evaluated the effects of length and frequency in sheep and human data. In general, the

performance of CNVcaller was better for all sizes of duplications but was poor for deletions <2.5 kb.

- 9:180. The "arbitrary standards" require a citation.

Two citations have been added as follows:

1. Chain FJ, Feulner PG, Panchal M, Eizaguirre C, Samonte IE, Kalbe M, et al. Extensive copy-number variation of young genes across stickleback populations. PLoS genetics. 2014;10 12:e1004830.

2. Abyzov A, Urban AE, Snyder M and Gerstein M. CNVnator: an approach to discover, genotype, and characterize typical and atypical CNVs from family and population genome sequencing. Genome research. 2011;21 6:974-84.

- Minor comment: Since speed seems to be a major selling point of the software, more details about running the software on a compute cluster or running algorithms in parallel in the documentation would be helpful.

A new section, “Parallel submission of individual RD processing,” has been added to the methods with the principle and commands as follows:
“Parallel processing of individual RDs. The CNVcaller processes the BAM file of each individual separately in the first step, and therefore, parallel computations can be performed to reduce the total running time. All BAM files are equally distributed into N groups, and each group contains M files. The max N is the total available processing cores, and M is the total number of BAM files/N. For example, the 232 goat BAM files were processed on a node with 32 processing cores and 124 GB of RAM. We distributed the 232 files into 20 groups, and each group contained 12 BAM files. The shell command for one group is as follows:

```
#!/bin/sh
for i in {1..M}
do bash Individual.Process.sh -b $i.bam -h $i -d dup -s sex_chromosome
done
```

After corrections and normalization, the comparable RDs of each sample are aggregated into an ~100 MB intermediate file and output, thus preventing repeated calculations for the same individual in different populations.”

****
Reviewer #2:

The proposed method "CNVcaller" enables the efficient discovery and genotyping of CNVs in large populations. One of the main benefits of the method is that it can handle draft genome assemblies with thousands of scaffolds. The computational benchmarks proof that the method is fast and memory efficient but the evaluation of the accuracy of the method is less convincing. Some details of the method remain vague and hinder an objective evaluation. Detailed comments

of how to improve the manuscript are below:

Thank you for your affirmation. We are sorry for the ambiguity of the accuracy test and have substantially revised the manuscript according to your suggestions. In the revised manuscript, the performance evaluation in the previous Table 1 is described in more detail in Figure 4 and Figure 6.

Comment 1 - The primary application of CNVcaller is the detection of CNVs in large populations. Population variant call sets are dominated by rare variants of rather small size. For instance, less than 20% of the 1000 Genomes structural variants have a population allele frequency >5% and almost 50% of the SVs are <2kbp in size despite the rather low coverage (~7x). CNVcaller is currently restricted to large CNVs (>2kbp) and common variants (>5% allele frequency), which is a major limitation for population genomic studies.

We apologise for the ambiguous. Actually, the user can retain all the windows with at least one individual that shows heterozygous deletion or duplication. However, we recommend removing low-frequency windows in large populations with low sequencing coverage because of increased random mistakes. In the revised version, Figure 6 was added to evaluate the effects of length and frequency by IRS test. We found Genome STRiP showed the greatest ability to detect short and rare deletions, indicating the advantage of combining RD and RP methods for deletion detection. However, short and rare duplications still had extremely high FDR. The shortest duplications reported by CNVnator and Genome STRiP were 2.8 kb and 2.5 kb, and the IRS FDRs of 2.5-5 kb calls were 29% and 88%, respectively. The FDRs of singletons were 35% and 69% for CNVnator and Genome STRiP, respectively. The main improvement of CNVcaller is the accuracy of duplications. The FDR of 2.5 kb – 5 kb duplications was reduced to 19%, and the FDR of singleton duplications were reduced to 9%. However, the FDRs were still higher than those of the longer and higher-frequency calls. So, these calls were removed from the previous manuscript. These uncertain calls were also removed by the phase 3 extended SV release of 1000GP. After extra quality controls, the number of duplications in the released database is only 1/7 the number of deletions, and the median size is 36 kb, which is 17 times longer than deletions. Therefore, improving the accuracy of duplications on this foundation is meaningful for enriching the CNV database.

Additionally, the current main use of CNVcaller is the detection of CNVRs related to economic traits in livestock and crops. In these populations, the target CNVRs usually have a medium or high frequency after long-duration artificial selection. We believe that the high-confidence medium to high frequency reported by CNVcaller can contribute to functional and breeding studies of animals and plants.

The sensitivity increase of CNVcaller for the subset of common and large CNVs seems to be driven by an increased number of detected CNVs in SD regions (Figure 5C). SNP arrays have a low SNP density in SD regions and in the present Manuscript array SNP probes in SD regions have been removed entirely. The reported IRS FDR is therefore heavily biased against CNVs in SD regions and it thus seems mandatory to me to proof that this sensitivity increase for SD-associated CNVs is not leading to an inflated FDR.

Thank you for your suggestions. Figure 5C (new Figure 3C) has been updated to show both the number and the Mendelian inconsistency of the detected CNVs in SDs. The Mendelian inconsistency rate of the calls in SD regions made by CNVcaller was approximately 3%, no higher the other methods. The copy numbers of unique and SDs were also indirectly validated by the X-origin scaffolds of a 133-sheep population. All of these scaffolds should be detected as CNVs because the rams had half the copy numbers of the ewes. As a result, CNVcaller detected 101 of these 138 X-origin scaffolds. In contrast, CNVnator and Genome STRiP did not report these regions.

The Manuscript lacks a Figure that shows the size and allele frequency distribution of the discovered CNVs in comparison to Genome STRiP and CNVnator. An estimate of breakpoint accuracy of CNVcaller would also be valuable.

Thank you for your suggestion. Figure 4 and Figure 6 have been added, which evaluate the effects of length and frequency in sheep and human data. The detailed comparisons in the manuscript are as follows:

Sheep: "The accuracy was evaluated by the Mendelian inconsistency of all the CNVRs on autosomes against the length and alternative allele frequency (Figure 4). CNVcaller achieved higher accuracy than Genome STRiP in both deletion (1% vs 2%) and duplication (4% vs 7%) (Figure 4A). Whereas Genome STRiP had greater capability to detected short (<2.5 kb) deletions (Figure 4B), indicating the RP methods integrated in Genome STRiP performed well on small deletions. Concerning the alternative allele frequency, both methods showed an increased FDR in rare duplications (Figure 4C). However, CNVcaller is primarily used to detect CNVRs related to economic traits in livestock and crops. In these studies, the target CNVRs usually have a high frequency after long-duration breeding selection."

Human: "CNVcaller demonstrated the highest overall accuracy for detecting duplications and performed consistently across the length and frequency categories, whereas Genome STRiP and CNVnator had high FDRs on the short or singleton duplications (Figure 6A, B). Genome STRiP showed the greatest ability to detect deletions, indicating the advantage of combining RD and RP methods for deletion detection. The genotyping accuracy of the human dataset was further benchmarked against the high-confidence aCGH array-based database. The discordance rates of CNVcaller, CNVnator and Genome STRiP were 2.6%, 5.5% and 2.2%, respectively. This genotyping accuracy ranking was the same with the Mendelian consistency of the 10 Dutch trios (Supplementary Figure 5)."

Thank you for your reminding of the breakpoint issue. However, unlike the PR/SP algorithm, RD can not detect breakpoints in the at base pair resolution or less than the window step size resolution. Integrating RD and RP methods can improve the breakpoint accuracy in human genome. However, precise breakpoint is more difficult to achieve in the poorly assembled genomes. Additionally, the breakpoint issue did not affect the genotyping accuracy which is the direct input of GWAS. The genotyping FDR of CNVcaller, CNVnator and Genome STRiP were 2.6%, 5.5% and 2.2%, respectively.

The Manuscript mentions mrsFAST for absolute copy number validation. I could not find any

formal comparison of predicted copy-number by mrsFAST and CNVcaller but maybe I missed this?

Supplementary Figure 2 (previous Supplementary Figure 1) shows that the copy numbers calculated using mrsFAST and CNVcaller were similar. However, mrsFAST needed to realign all the multi-hit reads in BWA alignments, leading to significantly increased computational time. For example, mrsFAST required 10 hours for a 3G genome with 10X sequencing data, whereas CNVcaller needed only 4 minutes.

- Please add to Table 1 the number of CNV sites that could be assessed by the IRS method and what proportion of each call set could be evaluated using IRS. I also believe the IRS method reports p-values separately for deletions, duplications and multi-allelic CNVs. Was there any difference among these for CNVcaller?

Detailed information on 1000GP calls, including the required information, has been added to Supplementary Table 5. Overall, 28%, 30% and 60% of the CNVRs of CNVcaller, CNVnator and Genome STRiP covered at least one probe of the Affymetrix SNP 6.0 array and therefore could be assessed using the IRS test. One main reason for the divergent testable proportions was that only 4% of Genome STRiP calls overlapped with SDs, which have infrequent probes, whereas 34% of the CNVcaller calls and 28% of the CNVnator calls overlapped with SDs.

Two extra genome-wide evaluations can provide supplemental evidence. The Mendelian inconsistency of 10 Dutch families was added to Supplementary Figure 5, which was based on tests of both unique and SD regions. The inconsistency rates of CNVcaller, CNVnator and Genome STRiP were 1.5%, 4.4%, and 0.4%, respectively. This accuracy ranking was consistent with the genotyping discordance values compared with the aCGH database, which were 2.6%, 5.5% and 2.2% for CNVcaller, CNVnator and Genome STRiP, respectively.

To analysis the difference between deletions and duplications, all FDRs were evaluated separately in the revised manuscript. We found the duplications had much higher FDRs than the deletions, especially for the short and rare CNVs.

- Some details of the method are vaguely specified and some Figures lack clarity and units. Page 6, line 129: "... if the median RD of the homogametic sex chromosomes is about half of the median RD of autosome..."

This section has been expanded in the newly added subsection “RD corrections for sex chromosomes” as follows:

“RD corrections for sex chromosomes. Most mammalian and avian genomes show an XX/XY-type or ZZ/ZW-type sex-determining system. Their homogametic sex chromosomes (X or Z) constitute 5%-10% of the total genome and show half the RD of the autosomes in XY or ZW individuals. Therefore, intensive correction for X and Z chromosomes is needed. The RD of the X or Z chromosome (the particular name provided by the user) is used to determine the sex of a particular individual. If the median RD of this chromosome is <0.6X the median RD of the autosome, the individual is considered an XY or ZW type, and the RDs of this chromosome are

doubled before normalization. Otherwise, nothing is performed for individuals determined to be XX or ZZ type."

Page 8, line 154: "... and the distance between them is less than a certain percent of their own length."

This text has been modified as follows: "As CNVRs can be separated by gaps or poorly assembled regions, the adjacent initial calls are merged if their RDs are highly correlated. The default parameters are as follows: the distance between the two initial calls is less than 20% of their combined length, and the Pearson's correlation index of the two CNVRs is significant at the P = 0.01 level."

Page 5, line 91: "The reference genome is segmented into overlapping sliding windows." What window size and overlap was used for high-coverage genomes?

The following description has been added to the methods.
"The window size is an important parameter for RD methods. CNVcaller uses half of the window size as the step size. The optimal window size is 800 bp (with a 400 bp overlap) for 5-10X coverage human and livestock sequencing data (Supplementary Figure 1). The recommended window sizes are inversely related with coverage, and thus, ~400 bp windows correspond to 20X coverage, and ~200 bp windows correspond to 50X coverage."

Page 5, line 95: "The raw RD signal is calculated for each window as the number of placed reads with centers within window boundaries." Does this imply that for paired-end data both reads are counted?

We apologise for the ambiguous description. The following description has been added:
"Considering the uncontrollable effect of gap ratios from different genome assemblies, all of the end reads located in the window are independently added to the RD of this window, regardless of whether the read is from single-end mapping or paired mapping."

Page 8, line 154: "Then the two adjacent initial calls are further merged if their copy numbers are highly correlated". What threshold was used?

This text has been modified as follows: "As CNVRs can be separated by gaps or poorly assembled regions, the adjacent initial calls are merged if their RDs are highly correlated. The default parameters are as follows: the distance between the two initial calls is less than 20% of their combined length, and the Pearson's correlation index of the two CNVRs is significant at the P = 0.01 level."

Figure 3A: CNVcaller 13.7. What is the unit? Are these 13,700 CNVs?

The unit in this figure is Mb. Because the intersection of the three methods with different boundaries was difficult to define in numbers, they were evaluated in terms of length. CNVcaller covered 40% of the CNVRs detected by CNVnator, 45% of the CNVRs detected by Genome STRiP and 65% of their intersecting CNVRs, in terms of length.

Minor:
- I could not find a reference to the 232 goat sequencing data? Is this data publicly available?

Among the 232 goat whole-genome sequencing data files, 103 files were acquired from NCBI, the accession numbers are provided in Supplementary Table 1. The remaining 129 samples without accession numbers were generated by ourselves, and will be published soon. The reference and unpublished paper are as follows:

1.Badr Benjelloun FJA, Streeter I, Boyer F, Coissac E, Stucki S, et al. (2015) Characterizing neutral genomic diversity and selection signatures in indigenous populations of Moroccan goats (Capra hircus) using WGS data. Frontiers in genetics 6.

2.Dong Y, Zhang X, Xie M, Arefnezhad B, Wang Z, et al. (2015) Reference genome of wild goat (capra aegagrus) and sequencing of goat breeds provide insight into genic basis of goat domestication. BMC genomics 16: 431.

3.Dong Y, Xie M, Jiang Y, Xiao N, Du X, et al. (2013) Sequencing and automated whole-genome optical mapping of the genome of a domestic goat (Capra hircus). Nature biotechnology 31: 135-141.

4.Bickhart DM, Rosen BD, Koren S, Sayre BL, Hastie AR, et al. (2017) Single-molecule sequencing and chromatin conformation capture enable de novo reference assembly of the domestic goat genome. Nature Genetics 49: 643-650.

5.Wang XL, Liu J, Niu YY, Li Y, Zhou SW, et al. Low incidence of SNVs and indels in trio genomes of Cas9-mediated multiplex edited sheep. BMC Genomics. Under review.

6.Zheng ZQ, Li M, Liu J, Wang XL, Pan XY, et al. The early domestication process inferred from genome analysis of worldwide goats. In preparation.

- The first Results section "Overview of CNVcaller algorithm" seems better suited for the Methods part.

This has been modified as suggested.

- Is the Mendelian consistency higher for the high-coverage trio: NA12878, her father (NA12891) and her mother (NA12892)?

Yes. In the high-coverage data of all three members of the trio (NA12891, NA12892 and NA12878 were all 50X), the inconsistency rate was 2.4%. In the high-coverage data of the parents (50X for NA12891 and NA12892) and the low-coverage data of the child (5.3X for NA12878), the inconsistency rate was 6.1%. Thus, increased sequencing depth can help to reduce the number of false positives.

- I believe the claim that read-pair/split-read algorithms are less powerful on draft assemblies of

non-model organisms compared to read-depth methods is potentially true but the Manuscript lacks a proof for this or a citation that supports this claim.

Thank you for your agreement. This problem was found in our previous reference genome assembly projects for both sheep and goats. However, we did not report this result in the section on CNV/SD detection. The review listed below has some comments about this claim, however, without direct supporting data. Therefore, we have removed this comment from this manuscript.

Bickhart DM and Liu GE. The challenges and importance of structural variation detection in livestock. Frontiers in genetics. 2014;5.

“While RP methods should provide a suitable means for detecting such events in theory, two major problems currently challenge the accuracy of this method:

(1) alignment errors resulting from the mapping of read pairs to repetitive regions of the genome…… The first problem (1) is unfortunately dependent on the reference genome assembly for the species, and is unlikely to be resolved until better reference assemblies are created for livestock.”

- It is not clear from the Manuscript if CNVcaller reports copy-number likelihoods based on the Gaussian mixture model. Please clarify.

Thank you for your suggestion. CNVcaller reports the silhouette coefficients of the copy numbers instead of the Gaussian mixture model likelihood as quality control because we found that silhouette coefficients had a greater correlation with the IRS test results than likelihood.

- Figure 5A: Why is the absolute copy-number correction different for Human and Sheep?

We are sorry for not clearly interpreting the high proportion of misassembled segmental duplications in non-human assemblies. This part of the manuscript has been modified as follows:

“Previous studies have shown that a high proportion of SDs in animal genomes are misassembled single-copy regions [27, 29]. Therefore, we detected the ratios of false SDs on the human (hg19) and sheep (OAR v3.1) reference genome assemblies by the sequencing copy number of a human (NA12878) and a Tan sheep sample (Figure 3A). If the SDs were correctly assembled, the sequencing diploid copy number should be twice the copy number of SDs. For example, the average sequencing copy number of the two-copy SDs was four in NA12878. However, the corresponding sequencing copy number in sheep was only 2.4. These results indicated that most two-copy SDs of hg19 were truly duplicated in NA12878, while approximately 80% of the two-copy SDs in OAR v3.1 were single-copy regions in the Tan sheep sample. Thus, the SDs in the sheep genome were called “putative SDs” before validation.”

- There is quite a few typing and grammatical errors. For instance:
*Figure 2B: Max mamory
*Supplementary Table 3: Memery
*Page 3, line 53: ...the number of reads aligned to of a particular region.

*Page 8, line 160: This model presets the average copy number of homozygous deletion, heterozygous deletion, normal, heterozygous deletion (duplication!), homozygous deletion (duplication!) at zero to four respectively.

We are sorry for these mistakes. We have proofread the revised manuscript and used a professional English-language editing service to minimize the grammatical errors.

****

Checklist of the updated tables and figures

Current version	Last version
Fig. 3	Fig. 5
Fig. 4A-C	Table 1 and newly added
Fig. 4D	Fig. 3B
Fig. 5	Fig. 4
Fig. 6A-C	Table 1 and newly added
Fig. 6D	Fig. 3A
Supplementary Fig. 1	Newly added
Supplementary Fig. 2	Supplementary Fig. 1
Supplementary Fig. 3	Newly added
Supplementary Fig. 4	Supplementary Fig. 2
Supplementary Fig. 5	Table 1 and newly added
Supplementary Table 4	Newly added
Supplementary Table 5	Newly added
