# Supplementary material for: CNVcaller: highly efficient and widely applicable software for detecting copy number variations in large populations
Source: Gigascience. 2017 Dec 4;6(12):1–12. doi: 10.1093/gigascience/gix115 (PMC5751039; doi:10.1093/gigascience/gix115)
Supplement: Reviewer_1_Report_(Original_Submission).pdf [file gix115_reviewer_1_report_(original_submission).pdf]

Reviewer Report

Title: CNVcaller: Highly Efficient and Widely Applicable Software for Detecting Copy Number Variations in Large Populations

Version: Original Submission **Date:** 6/12/2017

Reviewer name: Larry Singh

Reviewer Comments to Author:

The authors developed a new CNV caller pipeline which they called CNVcaller geared towards improved speed compared to existing CNV callers and improved accuracy for high complexity genomes. I commend the authors on their efforts to introduce improved algorithms and pipelines for an inherently difficult procedure, namely CNV calling. My comments are mostly suggestions for improvement as follows. Note, comments of the form (4:5 for example represent page 4, line 5).

There are several grammatical errors which make the paper somewhat confusing. I would strongly recommend further extensive English editing.

My main criticism of the analysis is one that I have seen repeatedly of most other CNV calling publications, and that is there is no sensitivity analysis. The authors here also suggest various parameters throughout their paper for performing CNV calling, but there is no analysis of how the results change if these parameters are adjusted, i.e. no analysis of how robust your algorithm is to changes in the parameters. As another example, Hong et al 27503473 has demonstrated that the biggest variability in calling CNVs is in terms of the CNV size. I suspect that the same can be said of CNVcaller. Please comment on what sizes of CNVs does CNV caller do well or poorly on.

Other comments:
* 2:32 "the prevalent.." is a gross exaggeration. I think you mean "a prevalent".

* 2:35 I don't think you mean geometric. I did not comment on other grammatical/English errors as there were too many to list individually. I would highly recommend getting help with the English in this paper.

* 3:53 "RD" is not defined.

* 6:120 Give a brief description of how CNVator handles GC bias. Also why 40% for the GC bias? Shouldn't this parameter be dependent on the organism of interest?

*. The commentary on certain genomes not being as complete as others is important. I suspect though that if a large percentage of the samples show a CNV in a genome that is newer or not as complete, then this observation may be more likely indicative of a problem with the reference. Can you comment?

* 7:145 I am not convinced Pearson's correlation is appropriate. Your data is likely to have outliers and non-normal data. A non-parametric test of correlation like Spearman's correlation (Kendall-Tau is likely too computational intensive), or performing correlation after 5 or 10% trimming may be more appropriate.

* cn.MOPS (Klambauer et al, PMID: 22302147) uses a mixture of Poissons as opposed to Gaussian Mixture Models for CNV detection. I suspect the mixture of Poissions will be superior to Gaussian Mixture Models when the read depths are low, and Gausssian mixtures may be more appropriate when read depths are high. How difficult is it to replace the Gaussian mixtures with Poisson mixtures and compare the performance? I feel that this analysis would be informative and potentially improve your algorithm.

* The term "CNVR" is critical for understanding the algorithm, and requires more explanation of the term.

* It would be helpful to include some further discussion on where you see that CNVcaller works better or worse than existing CNV calling software.

* 9:180. The "arbitrary standards" require a citation.

* Minor comment: Since speed seems to be a major selling point of the software, more details about running the software on a compute cluster or running algorithms in parallel in the documentation would be helpful.

Methods

Are the methods appropriate to the aims of the study, are they well described, and are necessary controls included? Yes

Conclusions

Are the conclusions adequately supported by the data shown? Yes

Reporting Standards

Does the manuscript adhere to the journal's guidelines on minimum standards of reporting? YesChoose an item.

Statistics

Are you able to assess all statistics in the manuscript, including the appropriateness of statistical tests used? Yes, and I have assessed the statistics in my report.

Quality of Written English

Please indicate the quality of language in the manuscript: Not suitable for publication unless extensively edited

Declaration of Competing Interests

Please complete a declaration of competing interests, considering the following questions:

- Have you in the past five years received reimbursements, fees, funding, or salary from an organisation that may in any way gain or lose financially from the publication of this manuscript, either now or in the future?
- Do you hold any stocks or shares in an organisation that may in any way gain or lose financially from the publication of this manuscript, either now or in the future?
- Do you hold or are you currently applying for any patents relating to the content of the manuscript?
- Have you received reimbursements, fees, funding, or salary from an organization that holds or has applied for patents relating to the content of the manuscript?
- Do you have any other financial competing interests?
- Do you have any non-financial competing interests in relation to this paper?

If you can answer no to all of the above, write 'I declare that I have no competing interests' below. If your reply is yes to any, please give details below.

I declare that I have no competing interests.

I agree to the open peer review policy of the journal. I understand that my name will be included on my report to the authors and, if the manuscript is accepted for publication, my named report including any attachments I upload will be posted on the website along with the authors' responses. I agree for my report to be made available under an Open Access Creative Commons CC-BY license (http://creativecommons.org/licenses/by/4.0/). I understand that any comments which I do not wish to be included in my named report can be included as confidential comments to the editors, which will not be published.

I agree to the open peer review policy of the journal

To further support our reviewers, we have joined with Publons, where you can gain additional credit to further highlight your hard work (see: https://publons.com/journal/530/gigascience). On publication of this paper, your review will be automatically added to Publons, you can then choose whether or not to claim your Publons credit. I understand this statement.

Yes
