# Supplementary material for: CNVcaller: highly efficient and widely applicable software for detecting copy number variations in large populations
Source: Gigascience. 2017 Dec 4;6(12):1–12. doi: 10.1093/gigascience/gix115 (PMC5751039; doi:10.1093/gigascience/gix115)
Supplement: Reviewer_2_Report_(Original_Submission).pdf [file gix115_reviewer_2_report_(original_submission).pdf]

Reviewer Report

Title: CNVcaller: Highly Efficient and Widely Applicable Software for Detecting Copy Number Variations in Large Populations

Version: Original Submission **Date:** 6/19/2017

Reviewer name: Tobias Rausch

Reviewer Comments to Author:

The proposed method "CNVcaller" enables the efficient discovery and genotyping of CNVs in large populations. One of the main benefits of the method is that it can handle draft genome assemblies with thousands of scaffolds. The computational benchmarks proof that the method is fast and memory efficient but the evaluation of the accuracy of the method is less convincing. Some details of the method remain vague and hinder an objective evaluation. Detailed comments of how to improve the manuscript are below:

- The primary application of CNVcaller is the detection of CNVs in large populations. Population variant call sets are dominated by rare variants of rather small size. For instance, less than 20% of the 1000 Genomes structural variants have a population allele frequency >5% and almost 50% of the SVs are <2kbp in size despite the rather low coverage (~7x). CNVcaller is currently restricted to large CNVs (>2kbp) and common variants (>5% allele frequency), which is a major limitation for population genomic studies.

- The sensitivity increase of CNVcaller for the subset of common and large CNVs seems to be driven by an increased number of detected CNVs in SD regions (Figure 5C). SNP arrays have a low SNP density in SD regions and in the present Manuscript array SNP probes in SD regions have been removed entirely. The reported IRS FDR is therefore heavily biased against CNVs in SD regions and it thus seems mandatory to me to proof that this sensitivity increase for SD-associated CNVs is not leading to an inflated FDR.

- The Manuscript lacks a Figure that shows the size and allele frequency distribution of the discovered CNVs in comparison to GenomeSTRiP and CNVnator. An estimate of breakpoint accuracy of CNVcaller would also be valuable.

- The Manuscript mentions mrsFAST for absolute copy number validation. I could not find any formal comparison of predicted copy-number by mrsFAST and CNVcaller but maybe I missed this?

- Please add to Table 1 the number of CNV sites that could be assessed by the IRS method and what proportion of each call set could be evaluated using IRS. I also believe the IRS method reports p-values separately for deletions, duplications and multi-allelic CNVs. Was there any difference among these for CNVcaller?

- Some details of the method are vaguely specified and some Figures lack clarity and units.
Page 6, line 129: "... if the median RD of the homogametic sex chromosomes is about half of the median RD of autosome..."
Page 8, line 154: "... and the distance between them is less than a certain percent of their own length."
Page 5, line 91: "The reference genome is segmented into overlapping sliding windows." What window size and overlap was used for high-coverage genomes?
Page 5, line 95: "The raw RD signal is calculated for each window as the number of placed reads with centers within window boundaries." Does this imply that for paired-end data both reads are counted?
Page 8, line 154: "Then the two adjacent initial calls are further merged if their copy numbers are highly correlated". What threshold was used?
Figure 3A: CNVcaller 13.7. What is the unit? Are these 13,700 CNVs?

Minor:
- I could not find a reference to the 232 goat sequencing data? Is this data publicly available?

- The first Results section "Overview of CNVcaller algorithm" seems better suited for the Methods part.

- Is the Mendelian consistency higher for the high-coverage trio: NA12878, her father (NA12891) and her mother (NA12892)?

- I believe the claim that read-pair/split-read algorithms are less powerful on draft assemblies of non-model organisms compared to read-depth methods is potentially true but the Manuscript lacks a proof for this or a citation that supports this claim.

- It is not clear from the Manuscript if CNVcaller reports copy-number likelihoods based on the Gaussian mixture model. Please clarify.

- Figure 5A: Why is the absolute copy-number correction different for Human and Sheep?

- There is quite a few typing and grammatical errors. For instance:
*Figure 2B: Max mamory
*Supplementary Table 3: Memery
*Page 3, line 53: ...the number of reads aligned to of a particular region.
*Page 8, line 160: This model presets the average copy number of homozygous deletion, heterozygous deletion, normal, heterozygous deletion (duplication!), homozygous deletion (duplication!) at zero to four respectively.

Methods

Are the methods appropriate to the aims of the study, are they well described, and are necessary controls included? Yes

Conclusions

Are the conclusions adequately supported by the data shown? No

Reporting Standards

Does the manuscript adhere to the journal's guidelines on [minimum standards of reporting?](#) YesChoose an item.

Statistics

Are you able to assess all statistics in the manuscript, including the appropriateness of statistical tests used? No, and I do not feel adequately qualified to assess the statistics.

Quality of Written English

Please indicate the quality of language in the manuscript: Needs some language corrections before being published

Declaration of Competing Interests

Please complete a declaration of competing interests, considering the following questions:

- Have you in the past five years received reimbursements, fees, funding, or salary from an organisation that may in any way gain or lose financially from the publication of this manuscript, either now or in the future?
- Do you hold any stocks or shares in an organisation that may in any way gain or lose financially from the publication of this manuscript, either now or in the future?
- Do you hold or are you currently applying for any patents relating to the content of the manuscript?
- Have you received reimbursements, fees, funding, or salary from an organization that holds or has applied for patents relating to the content of the manuscript?
- Do you have any other financial competing interests?
- Do you have any non-financial competing interests in relation to this paper?

If you can answer no to all of the above, write 'I declare that I have no competing interests' below. If your reply is yes to any, please give details below.

I declare that I have no competing interests.

I agree to the open peer review policy of the journal. I understand that my name will be included on my report to the authors and, if the manuscript is accepted for publication, my named report including any attachments I upload will be posted on the website along with the authors' responses. I agree for my

report to be made available under an Open Access Creative Commons CC-BY license (http://creativecommons.org/licenses/by/4.0/). I understand that any comments which I do not wish to be included in my named report can be included as confidential comments to the editors, which will not be published.

I agree to the open peer review policy of the journal

To further support our reviewers, we have joined with Publons, where you can gain additional credit to further highlight your hard work (see: https://publons.com/journal/530/gigascience). On publication of this paper, your review will be automatically added to Publons, you can then choose whether or not to claim your Publons credit. I understand this statement.

Yes
